# Effectiveness of physiotherapist-led exercise interventions for burn rehabilitation: A systematic review and meta-analysis

**DOI:** 10.1371/journal.pone.0316658

**Published:** 2024-12-31

**Authors:** Ulric Sena Abonie, Martin Ackah, Tapfuma Mudawarima, Alberta Rockson

**Affiliations:** 1 Department of Sport, Exercise & Rehabilitation, Northumbria University, Coach Lane Campus, Newcastle upon Tyne, United Kingdom; 2 Department of Physiotherapy, DDT College of Medicine, Gaborone, Botswana; 3 Physiotherapy Department, Korle bu Teaching Hospital, Accra, Ghana; Transilvania University of Brasov: Universitatea Transilvania din Brasov, ROMANIA

## Abstract

**Background:**

Exercise is utilised by physiotherapists to prevent complications and improve overall function and quality of life post-burn. However, the effect of physiotherapist-led exercise has not been comprehensively reviewed. Consequently, this study aimed to investigate the effectiveness of physiotherapy exercises for persons’ post-burn.

**Methods:**

PubMed, CINAHL, Cochrane Library, and Web of Science were searched from database inception to September 27, 2024, to identify relevant studies. Two independent reviewers screened and selected the articles. Studies were included if they were randomised controlled trials of physiotherapy exercises to improve functional outcomes in persons with post burn injuries. Extracted data included author’s surname and year, country, population type, sample size, age, and total body surface area, mode, frequency and duration of exercise. The quality of the evidence was assessed with the Cochrane risk of bias (RoB 2.0) tool. Narrative synthesis and meta-analysis were conducted to examine exercise effect on physical, physiological and psychological outcomes.

**Results:**

Out of 3610 records screened, eight articles involving 393 participants were deemed eligible for inclusion. Physiotherapy exercises significantly improved lean body mass and pulmonary function but did not improve quality of life. Meta-analysis showed significant effects for aerobic capacity (Hedge’s g = 1.13, 95% confidence interval: 0.44–1.83, p = 0.00) and muscle strength (Hedge’s g = 2.27, 95% confidence interval: 0.42–4.13, p = 0.02).

**Conclusion:**

Physiotherapy exercises have positive impacts on physical, physiological and psychological outcomes particularly aerobic capacity and muscle strength in individuals’ post burns. The heterogeneity in effects for all outcomes highlights the need for further research.

## Introduction

Burns are one of the most devastating forms of injury and a major global public health crisis that severely impacts not only on the affected individual and their quality of life but also on their family and society [1, 2]. Burn injuries are the fourth most common type of trauma worldwide [1, 3], and leads to chronic sequelae characterised by loss of lean body mass, reduced aerobic capacity and muscle weakness [4–7]. Burns are one of the leading causes of death globally, with an estimated 180, 000 deaths every year caused by burns [1], and a leading cause of morbidity including prolonged hospitalisation, post-traumatic stress disorder, depression, lifelong disability and dysfunction [8–10].

Interventions to improve outcomes are of utmost importance due to impact of burns on overall health and health related quality of life [11]. Physiotherapy is vital in the rehabilitation of person’s post-burn, and the ultimate objective is for persons to return to full independence post-burn. Exercise prescription is utilised in rehabilitation by physiotherapists to encourage persons post burn to take steps to prevent complication and improve overall function and quality of life [12, 13]. Physiotherapists encourage, advice and progress exercise programme for persons post burn as part of their rehabilitation, due to their increased risk of bed immobility [8].

Exercise (mainly aerobic and resistance exercise) improves and maintain health, weight, self-confidence and social skills in persons with burn injuries [11–15]. However, the results of previous reviews of exercise effect post burn on physical, physiological or psychological outcomes have been conflicting [16, 17]. While reviews reported a lack of significant effect of resistance exercise, aerobic exercise and combined aerobic with resistance exercise on aerobic capacity and muscle strength, and beneficial effect on health-rated quality of life [16, 17], one review reported positive effect on lean body mass [16], while another reported no effect on lean body mass [17]. The divergence in exercise effects on post-burn outcomes may be due to intervention characteristics such as intervention types, duration, and delivery. It is worth noting that these previous reviews did not assess potential effect of intervention component and thus a possible reason for the varied results in literature may be the intervention component such as intervention delivery (i.e. who provided).

As exercise may not always involve physiotherapists, it is unclear if intervention provided by physiotherapists who are trained in evidence-based approach is associated with greater efficacy. Understanding the core elements for success in exercise interventions for post burn injuries is critical to identify the suitable context for implementation and scaling up. Conversely, the effectiveness of physiotherapist-led exercise on physical, physiological or psychological outcomes in persons post-burn has not been systematically reviewed, necessitating a review to comprehensively evaluate the effect of exercise on functional outcomes in persons post burn. This is critical to help guide treatment efforts to improve functional outcomes in persons post burn. Consequently, the aim of this present study was to conduct a systematic review and meta-analysis of literature on the efficacy of physiotherapy exercise interventions to improve physical, physiological and psychological outcomes. We hypothesised that physiotherapist-led exercise interventions would lead to improved physical, physiological and psychological outcomes in persons with post-burn injuries.

## Materials and methods

### Best practices and study protocol registration

The Preferred Reporting Items for Systematic Reviews and Meta- Analyses (PRISMA) [18] was used to guide the conduct of this systematic review and meta-analysis to enhance the quality of the report. The study protocol was prospectively registered with the International Prospective Register of Systematic Reviews (PROSPERO) database (registration number: CRD42024550635).

### Eligibility criteria

The study included persons of all ages, both children and adults with post-burn injuries. There were no restrictions on sex, severity or the duration of burn injuries. The study only considered randomised controlled trials evaluating the effects of physiotherapist-led exercise interventions on functional outcomes in persons with post-burn injuries, with no restriction on time of publications. Any form of exercise, including resistance and aerobic training, with no restrictions on the frequency, intensity, or duration of the interventions was considered. Exercise interventions could be either standalone or in combination with other supplements (provided at least one group received an exercise only intervention). The control measures could be standard care without supervised exercises. Non-randomised controlled trials, observational studies, and review studies were excluded. The outcomes were aerobic capacity, muscle strength, lean body mass, quality of life, and pulmonary function.

### Data sources and search strategies

The search was conducted in PubMed, Cumulative Index to Nursing and Allied Health Literature (CINAHL), Cochrane Library, and Web of Science using Medical Subject Heading (MeSH) and relevant key words related to “burns,” “exercise,” “physiotherapy” and “functional capacity.” The searching period was from database inception to May 15, 2024, and updated (date last searched) on September 27, 2024. The search terms, along with their variants, were initially created in PubMed and subsequently modified for other databases. The search was limited to randomised controlled studies in humans, published in English. The full search strategy for PubMed is available as S1 File. Additionally, references of retrieved studies were manually searched to ensure relevant papers were not overlooked. Finally, grey literature was searched for and accessed through Google Scholar.

### Study selection and data extraction

All the articles retrieved from the databases were exported into Mendeley referencing manager where duplicates were removed. MA screened the titles and abstracts of the studies and USA cross checked and confirmed the eligibility of the studies. Subsequently, the full text of the articles was retrieved and screened by MA, with a secondary check by USA. Any disagreements on inclusion decisions were double checked and resolved through discussion consensus by USA and MA. Data from the eligible studies were extracted by MA using a standardised form and secondary check by USA. The extracted data included author’s surname and year, country, population type, sample size, age, and Total Body Surface Area (%). Mode, frequency and duration of exercise as well as control measures and outcome variables were also extracted.

### Risk of bias and quality assessment

The risk of bias and quality of studies was independently assessed by USA and MA, and disagreements were solved by consensus. The Cochrane risk of bias (ROB 2.0) [19] tool was used to assess the risk of bias in the included studies. The tool assessed five domains: sequence generation, allocation concealment, blinding, incomplete outcome reporting, and selective outcome reporting. Within each domain, the risk of bias was rated as ’low,’ ’high,’ ’some concerns,’ or ’no information.’ Additionally, an overall risk of bias was assigned a rating of ’low,’ ’high,’ ’some concerns,’ or ’no information.

Assessment of the certainty of the evidence across studies was assessed with the Grades of Recommendation, Assessment, Development, and Evaluation (GRADE) approach [20] independently by USA and MA, and disagreements were solved by consensus discussion. The GRADE tool assessed components related to study design, risk of bias, inconsistency, indirectness, imprecision and other considerations. A checklist, designed to aid consistency and reproducibility of GRADE assessments [21], was used to evaluate the evidence. The evidence was rated as high, moderate, low and very low quality.

### Data synthesis

The selection procedure was summarised using the PRISMA flow chart [18]. Initially, a systematic review of the outcomes was performed. Then, a meta-analysis was carried out if at least two studies reported the outcome data within the same population [i.e., children or adults]. For studies that reported standard error instead of standard deviation, the formula [SD = se √n] (SD = standard deviation, se = standard error, n = sample size) was used to calculate the standard deviation. The Random effects modelling method (to account for potential variability between participants or interventions) proposed by DerSimonian and Laird [22] was used to pool the results of homogenous studies. Pool effects were estimated using Hedges’ g statistic [23, 24] with corresponding 95% Confidence Interval (CI). Heterogeneity (I^2^) amongst the studies were quantified and interpreted according to Higgins and Thompson classification where 25%, 50% and ≥75% were considered as low, moderate and high heterogeneity, respectively [25]. As a result of the high heterogeneity (I^2^ = 88.23%), sub-group analyses were conducted (where applicable) to investigate potential variations in effects according to intervention types, duration, and participant characteristics. Microsoft Office 2013 and Stata (Stata Statistical Tools: Release 16; College Station, TX; Stata Corp LP) software were used for all statistical analyses.

## Results

**Fig 1:**
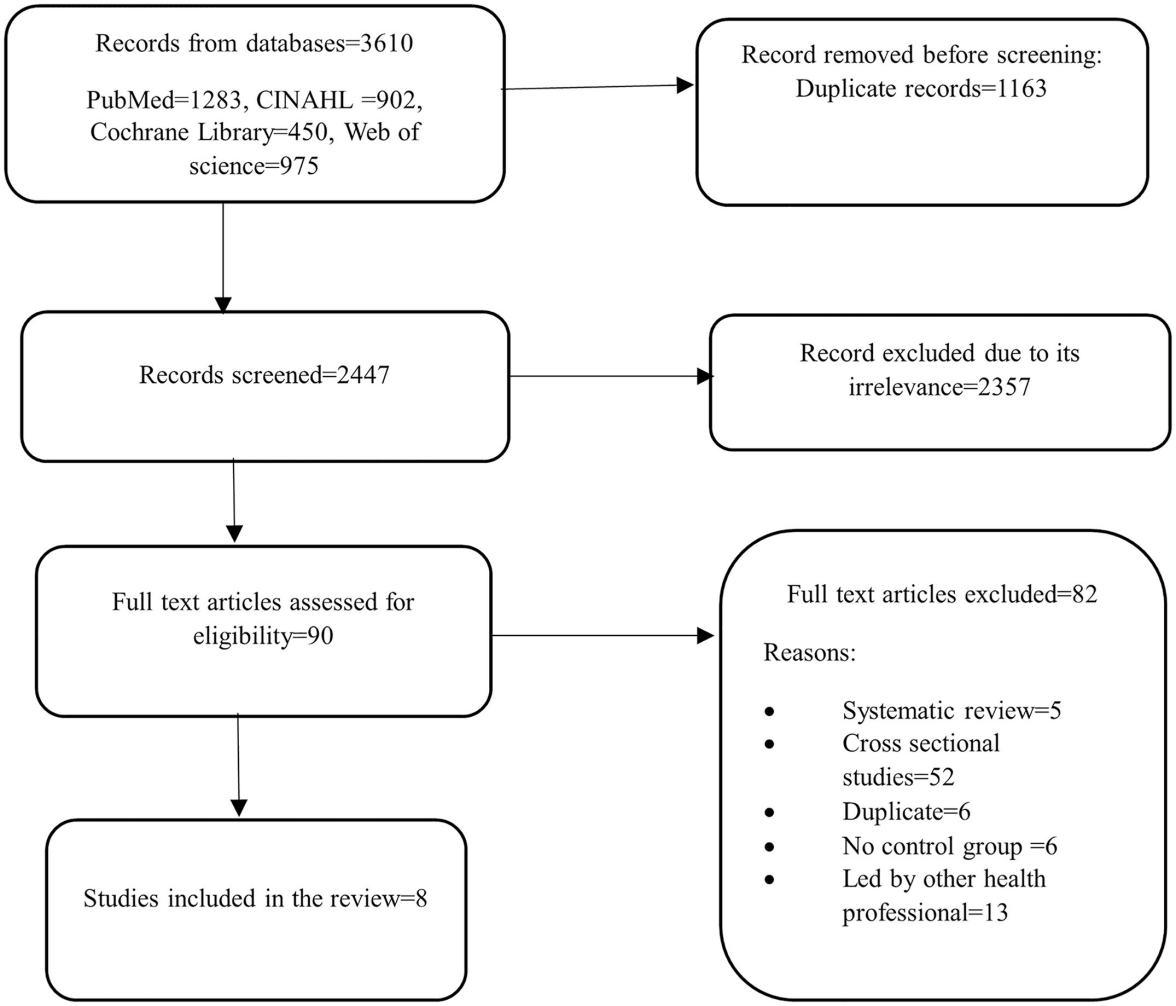
Flowchart of study screening and selection.

The search found 3,610 articles across the databases (see S2 File). Of these, 1,163 were removed as duplicates. During the title and abstract screening, 2,357 articles were excluded. Ninety studies were assessed for full text and potential eligibility. Consequently, eighty-two studies were excluded for being systematic reviews, cross-sectional studies, duplicates, lacking controls, or led by health professionals other than physiotherapists (see S3 File). Finally, eight studies [26–33] were included. The results are presented in Fig 1.

### Characteristics of the included studies and interventions

The study included 393 participants (198 intervention vs.195 control). Additionally, 75% (n = 6) of the studies were conducted in adults’ population [26–28, 30, 32, 33]. The sample size ranged from 25 to 110 participants. The intervention duration ranged from 6 to 12 weeks and sessions varied from 2 to 5 times per week. The Total Burns Surface Area (TBSA) ranged from 12% [30] to 59% [31] in the interventions group and 14% [30] to 60% [31] in the control group. Two studies were conducted in the United States of America [26, 31], three studies in Europe [27, 30, 32], two studies in Asia [29, 33], and one study in Africa [28]. Characteristics of the included studies are detailed in Table 1.

**Table 1:** Characteristics of the included studies.

Authors	Country	Population	Sample Size	Age (mean + SD or (range)) years	TBSA (mean + SD or (range)) %	Mode	Intensity; Frequency	Duration	Control Description	Outcome
Badawy et al. [26]	United States of America	Adults	Total = 30 Intervention = 15 Control = 15	Intervention = 35.86 ± 3.66 Control = 33.73 ± 4.13	Intervention = 34.73 ± 3.53 Control = 35.06 ± 3.28	Resistance and aerobic exercises	3 sets of 8–12 reps at 60% 3RM; 3 days a week	12 weeks	Standard care	Lean body mass, muscle strength
Çınar et al. [27]	Turkiye	Adults	Total = 25 Intervention = 16 Control = 9	Intervention = 19–56 Control = 19–56	Intervention = 25–50 Control = 25–50	Resistance and aerobic exercises	20 minutes bicycle ergometer at 10–12 RPE + resistance exercise; 5 days a week	Not reported	Standard care	Aerobic capacity
Ebid et al. [28]	Egypt	Adults	Total = 40 Intervention = 20 Control = 20	Intervention = 24.60 ± 5.33 Control = 27.30 ± 8.56	Intervention = 46.50 ± 3.14 Control = 44.50 ± 6.50	Resistance exercise	1–5 sets of 5 reps in first five sessions, 6 sets in the next 19 sessions and 10 set in the rest 12 sessions at 60% peak torque; 3 days a week	12 weeks	Standard care	Muscle strength
Elnaggar et al. [29]	Saudi Arabia	Children	Total = 36 Intervention = 18 Control = 18	Intervention = 14.10 ± 2.70 Control = 13.80 ± 2.00	Intervention = 24.9 ± 5.3 Control = 23.5 ± 4.7	Aerobic exercise	50% HR_max_ for 30min + 5% HR_max_ increase every 5min; biweekly	12 weeks	Standard care	Aerobic capacity, quality of life
Gittings et al. [30]	Austrilia	Adults	Total = 48 Intervention = 23 Control = 25	Intervention = 30 (25–33) Control = 33 (24–43)	Intervention = 12 (10–20) Control = 14 (9–20)	Resistance Training	3 sets of 8–12 reps at 70% 1RM; 3 days a week	6 weeks	Standard care	Muscle strength, quality of life
Hardee et al., [31]	United States of America	Children	Total = 47 Intervention = 24 Control = 23	Intervention = 13.00 ± 1.00 Control = 13.00 ± 1.00	Intervention = 59.00 ± 2.00 Control = 60.00 ± 3.00	Resistance and aerobic exercises	3 sets of 8–12 reps at 50–85% 3RM + 20–40 min 70–85% VO_2peak_; 3–5 days a week	12 weeks	Standard care	Lean body mass, aerobic capacity
Schieffelers et al. [32]	Belgium	Adults	Total = 57 Intervention = 28 Control = 29	Intervention = 48 (43–55) Control = 52 (47–58)	Intervention = 17 (10–60) Control = 18 (10–70)	Resistance and aerobic exercises	3 sets of 8–12 reps at 60% peak force, 3 days a week + 24min of 3-min intervals of 50% & 70% peak watts steep ramp test; 2 days a week	12 weeks	Standard care	Muscle strength, quality of life
Won et al. [33]	South Korea	Adults	Total = 110 Intervention = 54 Control = 56	Intervention = 43.60 ± 13.10 Control = 46.80 ± 13.90	Intervention = 36.93 ± 21.18 Control = 40.29 ± 19.18	Resistance, aerobic and deep breathing exercises	8–12 reps at 50–80% maximum strength + 4 and 6 on the Borg scale; 30-min 5 days a week	12 weeks	Standard care	Pulmonary function

HR_max_ = heart rate maximum; reps = repetitions; RPE = rate of perceived exertion; RM = repetition measurement; min = minute; SD = Standard deviation; TBSA = Total body surface area; VO_2peak_ = maximal oxygen uptake.

Regarding exercise mode, three studies used combined resistance and aerobic training [26, 27, 30, 32], two studies used resistance training [28, 30], another used grade aerobic exercise [29] and the other used circuit training consisting of resistive exercises, aerobic exercise, and deep breathing exercises [33]. The average frequency of the exercises was three times per week. The duration of the exercise program ranged from 6 weeks to 12 weeks. The primary outcomes of the studies were lean body mass [26, 31], muscle strength [26–28, 30, 32], aerobic capacity [27, 29, 31], quality of life [29, 30, 32] and pulmonary function [33].

Lean body mass was measured using dual-energy X-ray absorptiometry and a densitometry system in adults [26] and children [31], respectively. Muscle strength was measured using a handheld dynamometer, muscular ultrasound and Medical Research Council muscle-strength measurement scale in adults [27, 30, 32]. Additionally, studies measured muscle strength using a Biodex system dynamometer in children [26] and adults [28]. Aerobic capacity was assessed with 6-minute walk test and physiological cost index in adults [27], McMaster all-out progressive continuous cycling protocol and a modified Bruce treadmill protocol in children [29, 31]. Pulmonary function was measured with spirometry which included forced vital capacity in adults [33]. Quality of life was measured with the paediatric quality of life inventory in children [29] and the burn specific health scale-brief in adults [30, 32].

### Risk of bias and quality assessment

**Fig 2:**
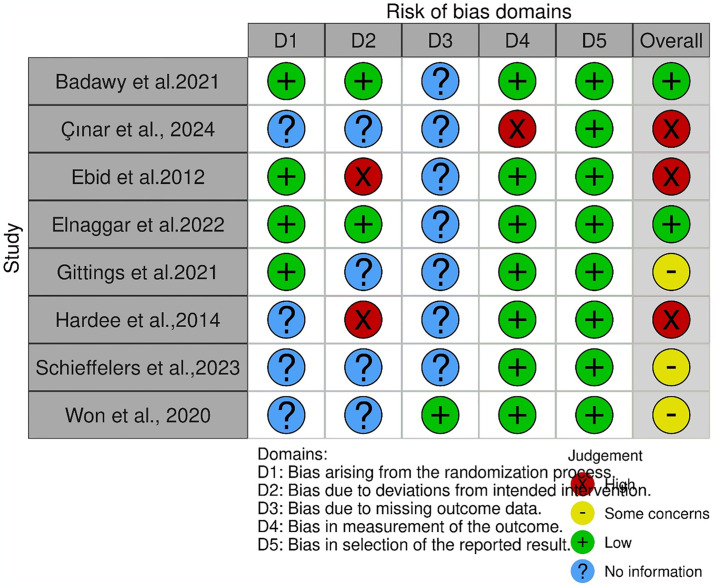
Risk of bias assessment.

Fig 2 shows the risk of bias and methodological quality of the included studies. Four studies [26, 28–30] reported on the randomisation process and four studies did not provide information on this domain [27, 31–33]. Seven studies did not provide information on missing outcomes [26–32]. Overall, two studies were adjudged high quality [26, 29], two low quality [28, 31], three studies had some concerns [30, 32, 33] and one was low quality [27].

Overall, quality of evidence for outcomes was assessed as moderate for aerobic capacity (VO_2peak_) and muscle mass, and low for lean body mass, pulmonary function and quality of life (S1 Table).

### Effects of exercise

#### Lean body mass

Lean body mass was assessed in two studies [26, 31]. Badawy and colleagues evaluated the efficacy of resistance exercises on lean body mass among adult patients with burn injuries and found that the exercise intervention significantly increased lean body mass compared to standard care at post-treatment [26]. Similarly, in a sample of children with burns, Hardee et al.,[31] found significant effects of resistance exercise on lean body mass at post-treatment.

#### Quality of life

Effects of exercise on quality of life post-assessment was assessed in three studies [29, 30, 32]. Elnaggar and colleagues reported that graded aerobic exercise significantly improved quality of life in children with post-burn injuries [29]. However, Gettings et al.,[30] and Schieffelers et al.,[32] reported that quality of life was not significantly different between the exercise and control groups in adults’ post-burn.

#### Pulmonary function

Only one study reported on the effects of exercise on pulmonary function in adults with post-burn injuries [33]. In a sample of 54 participants in the exercise group and 56 in the control group, Won et al.,[33] found significant improvements in diaphragmatic mobility, maximal inspiratory pressure and peak cough flow with exercise compared to the control group.

#### Muscle strength

**Fig 3:**
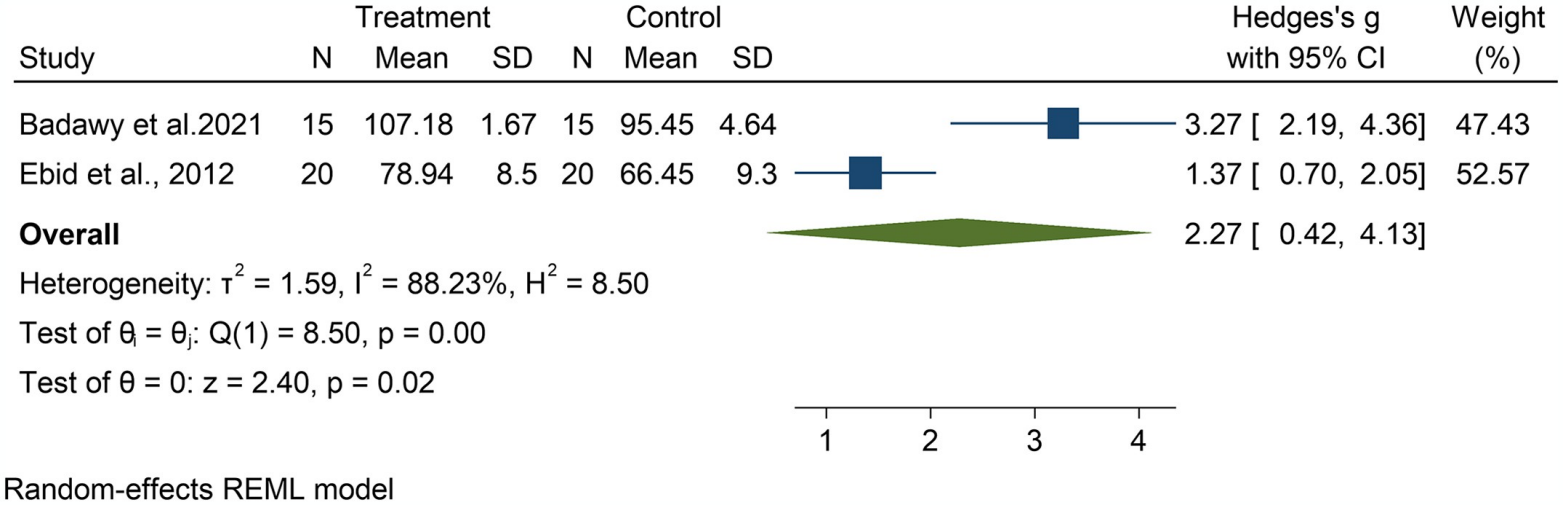
Effect of exercise on muscle strength in adults with burned injuries.

Five studies reported exercise effect on muscle strength post-treatment in adults [26–28, 30, 32], with four studies reporting statistical significance favouring the exercise group [26–28, 32]. The other study found no significant effects [30]. Data were pooled from two homogeneous studies [26, 28] using a random effects model. A significant difference favouring the exercise group was found [Hedge’s g = 2.27, 95%CI: 0.42; 4.13, p = 0.02]. However, the studies showed high heterogeneity [I^2^ = 88.23%]. The limited number of studies presenting data for muscle strength prevented the conduction of further potential moderator analyses. The forest plots of effect sizes comprising the main effects of exercise on muscle strength at post-treatment are illustrated in Fig 3.

#### Aerobic capacity

**Fig 4:**
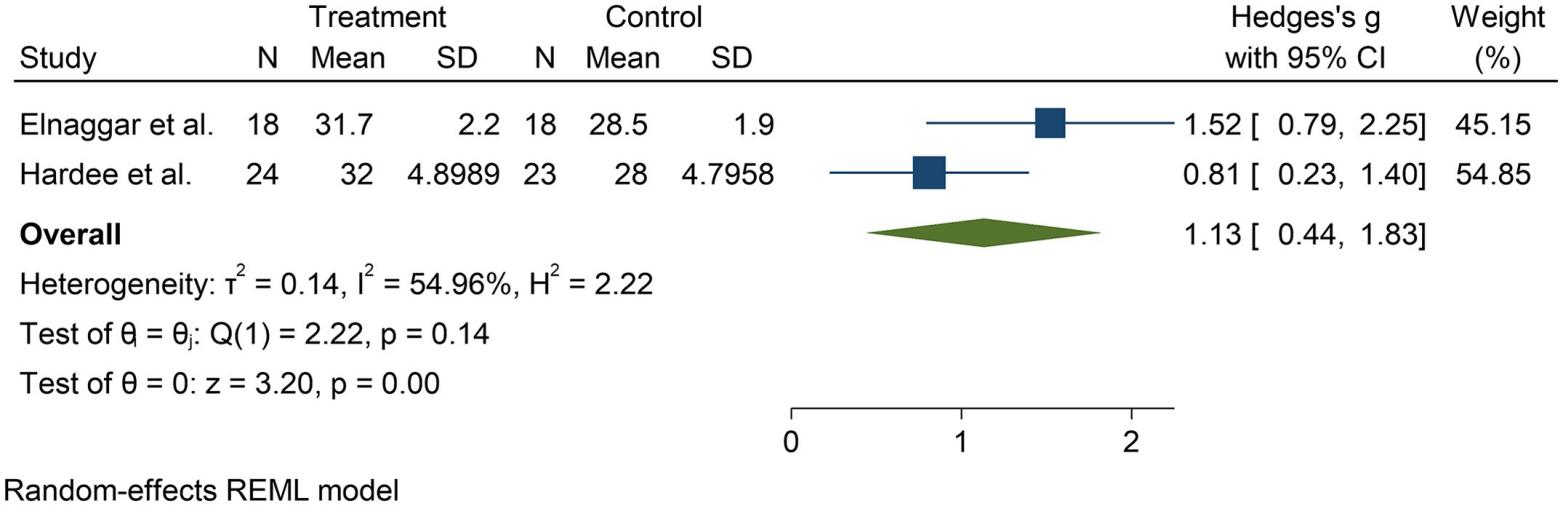
Effect of exercise on aerobic capacity in children with burned injuries.

Three studies reported exercise effect on aerobic capacity at post-treatment in children [29, 31] and adults [27], with all studies reporting statistical significance favouring the exercise group [27, 29, 31]. The pooled meta-analysis of the two studies in children was significant [Hedge’s g = 1.13, 95%CI: 0.44; 1.83, p = 0.00], favouring the exercise group. However, the studies showed high heterogeneity [I^2^ = 54.96%]. The limited number of studies presenting data for aerobic capacity prevented the conduction of further potential moderator analyses. The forest plots of effect sizes comprising the main effects of exercise on aerobic capacity at post-treatment are illustrated in Fig 4.

## Discussion

This systematic review and meta-analysis investigated the effects of physiotherapy exercises on muscle strength, aerobic capacity, lean body mass, health-related quality of life and pulmonary function in people with a burn injury. We identified eight studies with baseline assessment and post-treatment assessment of the study variables. To the best of our knowledge, this is the first study to comprehensively evaluate the impact of physiotherapist-led exercise interventions on functional outcomes in persons post burns to address a gap in literature in the context of whether intervention delivery has relevance on effect. Synthesis of studies revealed significant improvements in aerobic capacity, muscle strength, lean body mass and health-related quality of life.

Muscle strength was reported in four adult studies, that utilised aerobic and resistance exercise modes. According to the findings reported in the studies, significant improvements were observed in favour of the exercise intervention, and pooled analysis of these two studies confirmed the beneficial effect of exercise on muscle strength. The small number of studies that reported effects on muscle strength limited further analyses of potential sources of the heterogeneity observed in the pooled analysis. This indicates that there may be differences in the effect on muscle strength across participant and/or intervention characteristics. More research is needed to analyse the effects of potential moderators. The study findings are however somewhat similar to that of previous reviews assessing the effectiveness of exercise-based interventions post-burns on muscle strength that reported significant improvement in muscle strength following exercise training [16, 34]. This finding provides valuable insights, as muscle strength is a key indicator of physical function, affecting engagement in daily activities [34].

Aerobic capacity assessed with maximal oxygen uptake (VO_2peak_) was reported in two paediatric studies that utilised aerobic and resistance exercises. According to the findings reported in the studies, significant improvements were observed in favour of the exercise intervention, and pooled analysis of these two studies confirmed the beneficial effect of exercise on aerobic capacity. The small number of studies that reported effects on aerobic capacity limited further analyses of potential sources of the heterogeneity observed in the meta-analysis. This suggests that factors such as differences in intervention types, duration, and participant characteristics may influence exercise effect on aerobic capacity. Further research is needed to analyse the effects of potential moderators. Conversely, no studies assessed the effect of physiotherapist-led exercise on aerobic capacity in adult with burns, therefore further research is needed in this population. It is worth noting that previous reviews assessing the effects of exercise interventions on aerobic capacity in adults did not access effect of potential moderating factors such as intervention delivery, further justifying the need for the current study [16].

Lean body mass was reported in one paediatric study, and another adult study. Effect sizes favoured the exercise intervention. These findings are similar to that reported in previous systematic reviews and meta-analyses [16, 35]. Nedelec et al.,[35] in their review of practice guidelines for cardiovascular fitness and strengthening exercise prescription after burn injury concluded that exercise training leads to significant improvement in lean body mass.

Quality of life was reported in two adult studies, and one paediatric study. The narrative synthesis of physiotherapist-led exercise effect presented inconsistencies in the available evidence. While majority of the studies found no significant improvements in quality of life [30, 32], another study found no significant difference [29] between the intervention group and control group. This was contrary to the previous reviews finding of Flores et al.,[16] and Gittings et al.,[17] that included the same single study that assessed effectiveness of exercise on quality of life and found beneficial effect. A possible reason for the inconsistencies in effect for quality of life found in this review may be the time needed for a health behaviour change to take place, as literature suggest that it takes approximately 66 days (range: 18–254 days) to form a health behavioural habit [36]. Quality of life assessment point in the studies included in this current review varied from 56 days to 90 days, indicating that longer follow-up duration may be necessary to evaluate physiotherapist-led exercise effect on quality of life.

According to the finding of the single study in adults, physiotherapist-led exercise had a positive effect on pulmonary function reflected by the significant improvement in diaphragmatic mobility, maximal inspiratory pressure and peak cough flow in the exercise group. This finding was comparable to that reported in the systematic review and meta-analysis of Flores et al.,[16] which assessed effectiveness of exercise training on post-burn outcomes and found improvements in pulmonary function were related to maximal voluntary ventilation. Taken together, our systematic review suggested that physiotherapist-led exercise had a positive effect on muscle strength, aerobic capacity, and lean body mass.

### Limitations and recommendations

There was heterogeneity across populations, intervention types and protocols, and outcomes. The limited number of eligible and included studies, coupled with the uneven distribution of studies in subgroups limited the analyses of subgroups effect sizes of physiotherapist-led exercise on muscle strength and aerobic capacity as well as the conduction of meta-analysis on lean body mass, health-related quality of life and pulmonary function. The evaluation of evidence indicated that there is a lack of high-quality evidence for all outcomes. Additionally, the inclusion of only full-text studies which were published and available in English likely limits the generalisability of the review findings. However, after our thorough search of the literature, no study was excluded for this reason. Readers should therefore be cautious when interpreting the pooled effect sizes [37, 38].

The finding of this review indicates the importance of physiotherapist-led exercise intervention in burn rehabilitation. However, the wide variation observed across studies underscores the need for further exploration of potential moderator factors on burn outcomes. Among these are participant characteristics (such as age, gender, burn severity, duration, previous exercise experience) and intervention features (type, duration, number of sessions, setting). Additionally, the incorporation of patient centred outcomes such as general health, quality of life and functional abilities (including return to recreation and work) is crucial as these outcomes have been identified as key factors in rehabilitation.

Future randomised controlled trials should provide detailed description of the content of the intervention and present effect sizes and raw data (means and standard deviations) for all outcomes to enable future assessment of potential moderator effect of intervention characteristics. Furthermore, studies which adhere to CONSORT guidance [39], are adequately powered, where allocation is transparently randomised and concealed, and where blinded assessment can be truly undertaken are needed to improve the quality of research outcomes.

## Conclusion

This review examined the current evidence on physiotherapy exercises effect on functional outcomes and found positive effects on muscle strength, aerobic capacity and lean body mass in persons with burns. However, the limited number of included studies coupled with the wide variance currently available for these outcomes warrants the need for further exploratory studies in this context. Nonetheless, this study findings provides valuable insight that physiotherapists working in burn rehabilitation settings can influence burn outcome, and highlights the importance of physiotherapists in standard care of person’s post-burns. Notwithstanding the beneficial effects of physiotherapist-led exercise intervention reported in this review and the valuable implication for burn rehabilitation, high quality better-designed randomised controlled trials that are adequately powered, provided detailed description of the content and adheres to the CONSORT guidance are needed to identify optimal features of intervention. Conversely, findings of the study highlight the need to further explore moderators such as intervention and participant characteristics of exercise effect on all outcomes.

## Supporting information

S1 Checklist PRISMA 2020 checklist. (DOCX)

S1 Data (XLSX)

S1 File Full search strategy for PubMed. (DOCX)

S2 File Studies identified in the literature search. (DOCX)

S3 File Excluded studies with reasons. (DOCX)

S1 Table Quality of evidence using the GRADE approach. (DOCX)
