# Supplementary material for: Effectiveness of physiotherapist-led exercise interventions for burn rehabilitation: A systematic review and meta-analysis
Source: PLoS One. 2024 Dec 31;19(12):e0316658. doi: 10.1371/journal.pone.0316658 (PMC11687864; doi:10.1371/journal.pone.0316658)
Supplement: S2 File — (DOCX) [file pone.0316658.s004.docx]

**S2 File. Studies identified in the literature search**

**Total articles screened=2447**

1. A LG, AT B, AJ T, et al. Maximal oxygen consumption is best predicted by measures of cardiac size rather than function in healthy adults. *Eur J Appl Physiol*. 2012;112(6):2139-2147. doi:10.1007/s00421-011-2184-9

2. A LG, AT B, D’Hooge J, et al. Exercise strain rate imaging demonstrates normal right ventricular contractile reserve and clarifies ambiguous resting measures in endurance athletes. *J Am Soc Echocardiogr*. 2012;25(3):253-262.e1. doi:10.1016/j.echo.2011.11.023

3. Aaron SE, Tomoto T, Zhang R, et al. Statin contribution to middle cerebral artery blood flow velocity in older adults at risk for dementia. *Eur J Appl Physiol*. 2022;122(11):2417-2426. doi:10.1007/s00421-022-05022-1

4. Abazarnejad E, Froutan R, Ahmadabadi A, Mazlom SR. Improving respiratory muscle strength and health status in burn patients: a randomized controlled trial. *Qual life Res*. 2022;31(3):769‐776. doi:10.1007/s11136-021-02996-x

5. Abbas MI, Bamberger HB, Gebhart RW. Home treadmill injuries in infants and children aged to 5 years: a review of Consumer Product Safety Commission data and an illustrative report of case. *J Am Osteopath Assoc*. 2004;104(9):372-376.

6. Abbasi J. For Fat Burning, Interval Training Beats Continuous Exercise. *JAMA-JOURNAL Am Med Assoc*. 2019;321(22):2151-2152. doi:10.1001/jama.2019.4607

7. Abbass RA, Kumar P, El-Gendy A. Fine particulate matter exposure in four transport modes of Greater Cairo. *Sci Total Environ*. 2021;791:148104. doi:10.1016/j.scitotenv.2021.148104

8. Abdel-Aal NM, Allam NM, Eladl HM. Efficacy of whole-body vibration on balance control, postural stability, and mobility after thermal burn injuries: A prospective randomized controlled trial. *Clin Rehabil*. 2021;35(11):1555-1565. doi:10.1177/02692155211020861

9. Abdelbasset WK, Elsayed SH, Nambi G, et al. Optimization of pulmonary function, functional capacity, and quality of life in adolescents with thoracic burns after a 2-month arm cycling exercise programme: A randomized controlled study. *BURNS*. 2022;48(1):78-84. doi:10.1016/j.burns.2021.03.010

10. Abdelbasset WK, Elsayed SH, Nambi G, et al. Response to Letter to the Editor on “Potential efficacy of sensorimotor exercise program on pain, proprioception, mobility, and quality of life in diabetic patients with foot burns: a 12-week randomized control study.” *Burns*. 2021;47(5):1204‐1205. doi:10.1016/j.burns.2021.01.002

11. Abdelbasset WK, Abdelhalim NM. Assessing the effects of 6 weeks of intermittent aerobic exercise on aerobic capacity, muscle fatigability, and quality of life in diabetic burned patients: Randomized control study. *Burns*. 2020;46(5):1193-1200. doi:10.1016/j.burns.2019.12.013

12. Abdelbasset WK, Elsayed SH, Nambi G, et al. Response to Letter to the Editor on “Potential efficacy of sensorimotor exercise program on pain, proprioception, mobility, and quality of life in diabetic patients with foot burns: A 12-week randomized control study”. *Burns*. 2021;47(5):1204-1205. doi:10.1016/j.burns.2021.01.002

13. Abd-Elsayed A, Rupp A, D’Souza RS, et al. Interventional Pain Physician Burnout During the COVID-19 Pandemic: A Survey from the American Society of Pain and Neuroscience. *Curr Pain Headache Rep*. 2023;27(8):259-267. doi:10.1007/s11916-023-01121-6

14. Aberumand B, Jeimy S. The complexities of insulin allergy: a case and approach. *ALLERGY ASTHMA Clin Immunol*. 2021;17(1). doi:10.1186/s13223-021-00554-1

15. Agha RA, Pidgeon TE, Borrelli MR, et al. Validated Outcomes in the Grafting of Autologous Fat to the Breast: The VOGUE Study. Development of a Core Outcome Set for Research and Audit. *Plast Reconstr Surg*. 2018;141(5):633E-638E. doi:10.1097/PRS.0000000000004273

16. Agha RA, Fowler AJ, Pidgeon TE, Wellstead G, Orgill DP. Protocol for the development of a core outcome set for autologous fat grafting to the breast. *Int J Surg*. 2016;31:104-106. doi:10.1016/j.ijsu.2016.05.067

17. Aghajanzade M, Momeni M, Niazi M, et al. Effectiveness of incorporating occupational therapy in rehabilitation of hand burn patients. *Ann Burns Fire Disasters*. 2019;32(2):147-152.

18. Agrawal CS, Yadav V, Nikhade D. Physiotherapy to Alleviate Chest Complications in Acute Pancreatitis With Comorbidities: A Rare Case of Young Female. *Cureus*. 2024;16(6):e62000. doi:10.7759/cureus.62000

19. Ahmed ET, Abdel-aziem AA, Ebid AA. Effect of isokinetic training on quadriceps peak torgue in healthy subjects and patients with burn injury. *J Rehabil Med (Stiftelsen Rehabiliteringsinformation)*. 2011;43(10):930-934. doi:10.2340/16501977-0862

20. Ahmed S, Banwell P, Tiernan E. Exercise caution!--a hazard of treadmills. *Burns*. 2004;30(4):407-408. doi:10.1016/j.burns.2003.09.033

21. Aisbett B, Phillips M, Sargeant M, Gilbert B, Nichols D. Fighting with fire--how bushfire suppression can impact on fire fighters’ health. *Aust Fam Physician*. 2007;36(12):994-997.

22. Akkerman M, Mouton LJ, Disseldorp LM, et al. Physical activity and sedentary behavior following pediatric burns - a preliminary investigation using objective activity monitoring. *BMC Sport Sci Med Rehabil*. 2018;10. doi:10.1186/s13102-018-0093-5

23. Akkerman M, Mouton LJ, de Groot S, et al. Predictability of exercise capacity following pediatric burns: a preliminary investigation. *Disabil Rehabil*. 2021;43(5):703-712. doi:10.1080/09638288.2019.1641846

24. Akkerman M, Mouton LJ, de Groot S, et al. Predictability of exercise capacity following pediatric burns: a preliminary investigation. *Disabil Rehabil*. 2021;43(5):703-712. doi:10.1080/09638288.2019.1641846

25. Akkerman M, Mouton LJ, Dijkstra F, et al. Perceived fatigue following pediatric burns. *Burns*. 2017;43(8):1792-1801. doi:10.1016/j.burns.2017.05.007

26. Albadawi H, Tzika AA, Rask-Madsen C, et al. Revascularization and muscle adaptation to limb demand ischemia in diet-induced obese mice. *J Surg Res*. 2016;205(1):49-58. doi:10.1016/j.jss.2016.06.001

27. Albert M V, Deeny S, McCarthy C, Valentin J, Jayaraman A. Monitoring daily function in persons with transfemoral amputations using a commercial activity monitor: a feasibility study. *PM R*. 2014;6(12):1120-1127. doi:10.1016/j.pmrj.2014.06.006

28. Aldred S, Rohalu M, Edwards K, Burns V. Altered DHEA and DHEAS response to exercise in healthy older adults. *J Aging Phys Act*. 2009;17(1):77-88. doi:10.1123/japa.17.1.77

29. Aldrete JA, Ghaly R. Delayed sympathetically maintained pain caused by electrical burn at the current’s entry and exit sites. *J Pain Symptom Manage*. 1994;9(8):541-543. doi:10.1016/0885-3924(94)90118-x

30. Aleksovska K, Puggina A, Giraldi L, et al. Biological determinants of physical activity across the life course: a “Determinants of Diet and Physical Activity” (DEDIPAC) umbrella systematic literature review. *Sport Med - Open*. 2019;5(1):1. doi:10.1186/s40798-018-0173-9

31. Aleksovska K, Puggina A, Giraldi L, et al. Correction to: Biological determinants of physical activity across the life course: a “Determinants of Diet and Physical Activity” (DEDIPAC) umbrella systematic literature review. *Sport Med - open*. 2020;6(1):60. doi:10.1186/s40798-020-00291-6

32. Alfakir A, Arrowsmith C, Burns D, Razmjou H, Hardisty M, Whyne C. Detection of Low Back Physiotherapy Exercises With Inertial Sensors and Machine Learning: Algorithm Development and Validation. *JMIR Rehabil Assist Technol*. 2022;9(3):e38689. doi:10.2196/38689

33. Al-Ghabeesh SH, Mahmoud MM. Mindfulness and its Positive Effect on Quality of Life among Chronic Burn Survivors: A descriptive Correlational Study. *Burns*. 2022;48(5):1130-1138. doi:10.1016/j.burns.2021.09.022

34. Ali RR, Selim AO, Abdel Ghafar MA, Abdelraouf OR, Ali OI. Virtual reality as a pain distractor during physical rehabilitation in pediatric burns. *Burns*. 2022;48(2):303‐308. doi:10.1016/j.burns.2021.04.031

35. Ali ZA, Eladl HM, Abdelbasset WK, Eid MM, Mosa HE, Elsayeh SM. Inhalation injury in adult males: Evaluation of the short-term efficacy of transcutaneous electrical acupoint stimulation on pulmonary functions and diaphragmatic mobility after burn: A double-blind randomized controlled study. *Burns*. 2022;48(8):1933-1939. doi:10.1016/j.burns.2022.01.015

36. Ali ZMI, El-Refay BH, Ali RR. Aerobic exercise training in modulation of aerobic physical fitness and balance of burned patients. *J Phys Ther Sci*. 2015;27(3):585-589. doi:10.1589/jpts.27.585

37. Alirezaei P, Ahmadpanah M, Rezanejad A, Soltanian A, Bahmani DS, Brand S. Compared to Controls, Individuals with Lichen Planopilaris Have More Depression, a Lower Self-Esteem, and a Lower Quality of Life. *Neuropsychobiology*. 2019;78(2):95-103. doi:10.1159/000499135

38. Allahham A, Cooper MN, Fear MW, Martin L, Wood FM. Quality of life in paediatric burn patients with non-severe burns. *Burns*. 2023;49(1):220-232. doi:10.1016/j.burns.2022.03.012

39. Allam NM, Badawy MM. Does High-Frequency Chest Wall Oscillation Have an Impact on Improving Pulmonary Function in Patients With Smoke Inhalation Injury? *J Burn care Res*. 2021;42(2):300‐304. doi:10.1093/jbcr/iraa147

40. Allam NM, Badawy MM, Elimy DA. Effect of Pilates exercises on pulmonary function, respiratory muscle strength, and functional capacity in patients with inhalation injury after flame thermal burn: A prospective randomized controlled trial. *Burns*. 2024;50(9):107284. doi:10.1016/j.burns.2024.10.005

41. Allan J, Sadko K, Bell C, Johnston D. How many calories do nurses burn at work? A real-time study of nurses’ energy expenditure. *J Res Nurs*. 2019;24(7):488-497. doi:10.1177/1744987119837586

42. Allemann TS, Dhamrait GK, Fleury NJ, et al. Low-dose UV radiation before running wheel access activates brown adipose tissue. *J Endocrinol*. 2020;244(3):473-486. doi:10.1530/JOE-19-0470

43. Allen BA, Hannon JC, Burns RD, Williams SM. Effect of a core conditioning intervention on tests of trunk muscular endurance in school-aged children. *J strength Cond Res*. 2014;28(7):2063-2070. doi:10.1519/JSC.0000000000000352

44. Almutairi N, Burns S, Portsmouth L. Physical Activity Knowledge, Attitude, and Behaviours Among Adolescents in the Kingdom of Saudi Arabia Prior to and during COVID-19 Restrictions. *J Obes*. 2022;2022:1892017. doi:10.1155/2022/1892017

45. Al-Rawas OA, Al-Maniri AA, Al-Riyami BM. Home exposure to Arabian incense (bakhour) and asthma symptoms in children: a community survey in two regions in Oman. *BMC Pulm Med*. 2009;9:23. doi:10.1186/1471-2466-9-23

46. Alschuler KN, Krabak BJ, Kratz AL, et al. Pain Is Inevitable But Suffering Is Optional: Relationship of Pain Coping Strategies to Performance in Multistage Ultramarathon Runners. *WILDERNESS Environ Med*. 2020;31(1):23-30. doi:10.1016/j.wem.2019.10.007

47. Alschuler KN, Kratz AL, Lipman GS, et al. How variability in pain and pain coping relate to pain interference during multistage ultramarathons. *Pain*. 2019;160(1):257-262. doi:10.1097/j.pain.0000000000001397

48. Althunayan AM, Elkoushy MA, Elhilali MM, Andonian S. Adverse events resulting from lasers used in urology. *J Endourol*. 2014;28(2):256-260. doi:10.1089/end.2013.0451

49. Altin C, Kvist Lindholm S, Wejdmark M, Lättman-Masch R, Boldemann C. Upgrading Preschool Environment in a Swedish Municipality: Evaluation of an Implementation Process. *Health Promot Pract*. 2015;16(4):583-591. doi:10.1177/1524839914566273

50. Alwatban MR, Liu YM, Perdomo SJ, et al. TCD Cerebral Hemodynamic Changes during Moderate-Intensity Exercise in Older Adults. *J NEUROIMAGING*. 2020;30(1):76-81. doi:10.1111/jon.12675

51. AM AM, FN W, RP M, et al. Effects of exercise training on resting energy expenditure and lean mass during pediatric burn rehabilitation. *J Burn Care Res*. 2010;31(3):400-408. doi:10.1097/BCR.0b013e3181db5317

52. Amin Yousif PH, Mirlashari J, Nasrabadi AN, Jahanbani S. Coping with suicide’ burning scar’s disfigurement among Kurdish women in Iraq (Kurdistan). *Burns*. 2022;48(2):448-455. doi:10.1016/j.burns.2021.05.009

53. Amin M, Lee EB, Bhutani T, Wu JJ. Do psoriasis patients engage in vigorous physical activity? *Cutis*. 2018;101(3):198-200.

54. Ammitzbøll G, Lanng C, Kroman N, et al. Progressive strength training to prevent LYmphoedema in the first year after breast CAncer–the LYCA feasibility study. *Acta Oncol (Madr)*. 2017;56(2):360-366. doi:10.1080/0284186X.2016.1268266

55. Anane LH, Edwards KM, Burns VE, et al. Mobilization of gammadelta T lymphocytes in response to psychological stress, exercise, and beta-agonist infusion. *Brain Behav Immun*. 2009;23(6):823-829. doi:10.1016/j.bbi.2009.03.003

56. Anane LH, Edwards KM, Burns VE, Zanten JJCSV van, Drayson MT, Bosch JA. Phenotypic characterization of gammadelta T cells mobilized in response to acute psychological stress. *Brain Behav Immun*. 2010;24(4):608-614. doi:10.1016/j.bbi.2010.01.002

57. Andersen Hammond E, Pitz M, Shay B. Neuropathic Pain in Taxane-Induced Peripheral Neuropathy: Evidence for Exercise in Treatment. *Neurorehabil Neural Repair*. 2019;33(10):792-799. doi:10.1177/1545968319860486

58. Anderson HS, Kluding PM, Gajewski BJ, Donnelly JE, Burns JM. Reliability of peak treadmill exercise tests in mild Alzheimer disease. *Int J Neurosci*. 2011;121(8):450-456. doi:10.3109/00207454.2011.574762

59. Anderson JC, Fritz ML, Benson JM, Tracy BL. Nerve Decompression and Restless Legs Syndrome: A Retrospective Analysis. *Front Neurol*. 2017;8:287. doi:10.3389/fneur.2017.00287

60. Anderson T, Adams WM, Burns GT, et al. Addressing Circadian Disruptions in Visually Impaired Paralympic Athletes. *Int J Sports Physiol Perform*. 2024;19(2):212-218. doi:10.1123/ijspp.2023-0267

61. Andrews SK, Carek PJ. Osteitis pubis: a diagnosis for the family physician. *J Am Board Fam Pract*. 1998;11(4):291-295. doi:10.3122/jabfm.11.4.291

62. Andy UU, Jelovsek JE, Carper B, et al. Impact of treatment for fecal incontinence on constipation symptoms. *Am J Obstet Gynecol*. 2020;222(6):590.e1-590.e8. doi:10.1016/j.ajog.2019.11.1256

63. Angelou IK. Factors that influence the recovery of physical function of adult patients with major burn injuries. *WiredspaceWitsAcZa*. Published online 2020. https://wiredspace.wits.ac.za/server/api/core/bitstreams/3c6c663a-d2dd-48a4-b43d-9052ae7730fd/content

64. Anthonissen M, Daly D, Janssens T, Van den Kerckhove E. The effects of conservative treatments on burn scars: A systematic review. *Burns*. 2016;42(3):508-518. doi:10.1016/j.burns.2015.12.006

65. Ao M, Wu J, Chen J. [Investigation of burn rehabilitation development of China in 2014]. *Zhonghua Shao Shang Za Zhi*. 2017;33(5):260-266. doi:10.3760/cma.j.issn.1009-2587.2017.05.002

66. Aquino RK, Perez M, Sil P, Shintani T, Harrigan R, Rodriguez B. The Relationship of 5-Aminolevulinic Acid on Mood and Coping Ability in Prediabetic Middle Aged and Older Adults. *Geriatrics*. 2018;3(2). doi:10.3390/geriatrics3020017

67. AR E, Parutis V, Hart G, et al. The sexual attitudes and lifestyles of London’s Eastern Europeans (SALLEE Project): design and methods. *BMC Public Health*. 2009;9(1):399. doi:10.1186/1471-2458-9-399

68. Aralbaev TA. [Cicatricial contracture after burns of the arm and trunk]. *Sov Zdravookhr Kirg*. 1973;4:63-64.

69. Armstrong M, Hume E, McNeillie L, et al. Cognitive behavioural therapy combined with physical activity behavioural modification strategies during pulmonary rehabilitation in patients with COPD. *ERJ open Res*. 2023;9(5). doi:10.1183/23120541.00074-2023

70. Armstrong M, Hume E, McNeillie L, et al. Behavioural modification interventions alongside pulmonary rehabilitation improve COPD patients’ experiences of physical activity. *Respir Med*. 2021;180:106353. doi:10.1016/j.rmed.2021.106353

71. Arnaoutis G, Kavouras SA, Angelopoulou A, et al. Fluid Balance During Training in Elite Young Athletes of Different Sports. *J strength Cond Res*. 2015;29(12):3447-3452. doi:10.1519/JSC.0000000000000400

72. Arnold BF, Schiff KC, Griffith JF, et al. Swimmer illness associated with marine water exposure and water quality indicators: impact of widely used assumptions. *Epidemiology*. 2013;24(6):845-853. doi:10.1097/01.ede.0000434431.06765.4a

73. Arnson Y, Rozanski A, Gransar H, et al. Impact of Exercise on the Relationship Between CAC Scores and All-Cause Mortality. *JACC Cardiovasc Imaging*. 2017;10(12):1461-1468. doi:10.1016/j.jcmg.2016.12.030

74. Arrowsmith C, Burns D, Mak T, Hardisty M, Whyne C. Physiotherapy Exercise Classification with Single-Camera Pose Detection and Machine Learning. *Sensors (Basel)*. 2022;23(1). doi:10.3390/s23010363

75. Arshad Z, Rehan M, Iqbal T, et al. Treadmill versus overground gait training in patients with lower limb burn injury: a comparative study. *J Burn care Res*. 2023;44(5):1150‐1153. doi:10.1093/jbcr/irad043

76. Arshad Z, Rehan M, Iqbal T, Waheed U. Reply to Letter to the Editor Concerning the Article: “Treadmill Versus Overground Gait Training in Patients with Lower Limb Burn Injury: A Comparative Study”. *J Burn care Res Off Publ Am Burn Assoc*. 2023;44(5):1259. doi:10.1093/jbcr/irad093

77. Arzbaecher R, Jenkins JM. A review of the theoretical and experimental bases of transesophageal atrial pacing. *J Electrocardiol*. 2002;35 Suppl:137-141. doi:10.1054/jelc.2002.37171

78. Asghar AA, Faiq A, Shafique S, et al. Prevalence and Predictors of the Burnout Syndrome in Medical Students of Karachi, Pakistan. *CUREUS J Med Sci*. 2019;11(6). doi:10.7759/cureus.4879

79. Ashe MC, Azim FT, Ariza‐Vega P, et al. Determinants of implementing reablement into research or practice: A concept mapping study. *Physiother Res Int*. 2022;27(3):1-10. doi:10.1002/pri.1949

80. Ashraf B, Tasnim N, Saaiq M, Khaleeq-Uz-Zaman. Informed consent for surgery: do our current practices conform to the accepted standards? *J Coll Physicians Surg Pak*. 2014;24(10):775-777. doi:10.2014/JCPSP.775777

81. Asiva Noor Rachmayani. No 主観的健康感を中心とした在宅高齢者における 健康関連指標に関する共分散構造分析Title. Published online 2015:6.

82. Aspinall B, Burns C, Robins D. Measles--a fire-fighting exercise. *Community Med*. 1988;10(4):341-343. doi:10.1093/oxfordjournals.pubmed.a042428

83. Assiri A, Hamzi SA, Hamzi YA, et al. Knowledge, Attitude, Reception, and Preventive Practices Towards Skin Photoaging Among the General Population in Jazan, Saudi Arabia. *Cureus*. 2024;16(3):e55710. doi:10.7759/cureus.55710

84. Astorino TA, Martin BJ, Wong K, Schachtsiek L. Effect of acute caffeine ingestion on EPOC after intense resistance training. *J Sports Med Phys Fitness*. 2011;51(1):11-17.

85. AT B, A LG, AI M, et al. Augmentation of left ventricular torsion with exercise is attenuated with age. *J Am Soc Echocardiogr*. 2008;21(4):315-320. https://search.ebscohost.com/login.aspx?direct=true&AuthType=cookie,ip,shib,uid&db=cul&AN=105725646&site=ehost-live&scope=site

86. Atchley AE, Kitzman DW, Whellan DJ, et al. Myocardial perfusion, function, and dyssynchrony in patients with heart failure: baseline results from the single-photon emission computed tomography imaging ancillary study of the Heart Failure and A Controlled Trial Investigating Outcomes of Exercise T. *Am Heart J*. 2009;158(4 Suppl):S53-63. doi:10.1016/j.ahj.2009.07.009

87. Atkins WC, Foster J, McKenna ZJ, et al. Exercise and Heat Stress in Well-Healed Burn Survivors: Effects of Cooling Modalities on Thermal and Perceptual Responses. *Med Sci Sports Exerc*. Published online September 2024. doi:10.1249/MSS.0000000000003557

88. Atkins WC, Romero SA, Moralez G, et al. Attrition of Well-Healed Burn Survivors to a 6-Month Community-Based Exercise Program: A Retrospective Evaluation. *J Burn care Res Off Publ Am Burn Assoc*. 2023;44(6):1478-1484. doi:10.1093/jbcr/irad063

89. Atkins WC, Romero SA, Moralez G, et al. Attrition of Well-Healed Burn Survivors to a 6-Month Community-Based Exercise Program: A Retrospective Evaluation. *J Burn CARE Res*. 2023;44(6):1478-1484. doi:10.1093/jbcr/irad063

90. Attalla MF, al-Baker AA, al-Ekiabi SA. Friction burns of the hand caused by jogging machines: a potential hazard to children. *Burns*. 1991;17(2):170-171. doi:10.1016/0305-4179(91)90146-8

91. Attias L, Bucchi AR, Maranghi F, Holt S, Marcello I, Zapponi GA. Crude oil spill in sea water: an assessment of the risk for bathers correlated to benzo(a)pyrene exposure. *Cent Eur J Public Health*. 1995;3(3):142-145.

92. Atz AM, Zak V, Mahony L, et al. Longitudinal Outcomes of Patients With Single Ventricle After the Fontan Procedure. *J Am Coll Cardiol*. 2017;69(22):2735-2744. doi:10.1016/j.jacc.2017.03.582

93. Austin KG, Hansbrough JF, Dore C, Noordenbos J, Buono MJ. Thermoregulation in burn patients during exercise. *J Burn Care Rehabil*. 2003;24(1):9-14. doi:10.1097/00004630-200301000-00004

94. Aydin Y, Hassa H, Oge T, Yalcin OT, Mutlu FS. Frequency and determinants of urogenital symptoms in postmenopausal Islamic women. *Menopause*. 2014;21(2):182-187. doi:10.1097/GME.0b013e3182937966

95. Azizi E, Kushelevsky AP, Schewach-Millet M. Efficacy of topical sunscreen preparations on the human skin: combined indoor-outdoor study. *Isr J Med Sci*. 1984;20(7):569-577.

96. Babar SA, Ahmad I, Mughal IS. Sliding-mode-based controllers for automation of blood glucose concentration for type 1 diabetes. *IET Syst Biol*. 2021;15(2):72-82. doi:10.1049/syb2.12015

97. Bach J, Draslov B, Jørgensen B. Positioning, splinting and pressure management of the burned hand: a method. *Scand J Plast Reconstr Surg*. 1984;18(1):145-147. doi:10.3109/02844318409057417

98. Bacher-Stier C, Sharir T, Kavanagh PB, et al. Postexercise lung uptake of 99mTc-sestamibi determined by a new automatic technique: validation and application in detection of severe and extensive coronary artery disease and reduced left ventricular function. *J Nucl Med*. 2000;41(7):1190-1197.

99. Badawy MM, Allam NM. Impact of Adding Protein Supplementation to Exercise Training on Lean Body Mass and Muscle Strength in Burn Patients. *J Burn care Res*. 2021;42(5):968‐974. doi:10.1093/jbcr/irab007

100. Bai Y, Burns R, Gell N, Byun W. A randomized trial to promote physical activity in adult pre-hypertensive and hypertensive patients. *J Sports Sci*. 2022;40(14):1648-1657. doi:10.1080/02640414.2022.2099179

101. Bai Y, Copeland WE, Burns R, et al. Ecological Momentary Assessment of Physical Activity and Wellness Behaviors in College Students Throughout a School Year: Longitudinal Naturalistic Study. *JMIR PUBLIC Heal Surveill*. 2022;8(1). doi:10.2196/25375

102. Bai Y, Burns R, Ma C, Curl A, Hudziak J, Copeland WE. Tracking Well-Being: A Comprehensive Analysis of Physical Activity and Mental Health in College Students Across COVID-19 Phases Using Ecological Momentary Assessment. *Scand J Med Sci Sports*. 2024;34(10):e14738. doi:10.1111/sms.14738

103. Baker CSR. Learning on the Web. Case 3: acute chest pain. *Heart*. 2004;90(1):112. doi:10.1136/heart.90.1.112

104. Baker MS. Creating order from chaos: part I: triage, initial care, and tactical considerations in mass casualty and disaster response. *Mil Med*. 2007;172(3):232-236. doi:10.7205/milmed.172.3.232

105. Baldwin JN, McKay MJ, Hiller CE, et al. Forming norms: informing diagnosis and management in sports medicine. *Br J Sports Med*. 2015;49(19):1226-1227. doi:10.1136/bjsports-2014-094489

106. Baldwin JN, McKay MJ, Hiller CE, et al. Defining health and disease: setting the boundaries for physiotherapy. Are we undertreating or overtreating? How can we tell? *Br J Sports Med*. 2015;49(19):1225-1226. doi:10.1136/bjsports-2014-094488

107. Baldwin J, Li F. Exercise behaviors and barriers to exercise in adult burn survivors: A questionnaire survey. *Burn trauma*. 2013;1(3):134-139. doi:10.4103/2321-3868.123075

108. Baldwin J, Li F. Exercise behaviors after burn injury. *J Burn Care Res*. 2013;34(5):529-536. doi:10.1097/BCR.0b013e31827a2bcd

109. Banach M, Juranek JK, Zygulska AL. Chemotherapy-induced neuropathies-a growing problem for patients and health care providers. *Brain Behav*. 2017;7(1):e00558. doi:10.1002/brb3.558

110. Banever GT, Moriarty KP, Sachs BF, Courtney RA, Konefal SHJ, Barbeau L. Pediatric hand treadmill injuries. *J Craniofac Surg*. 2003;14(4):482-487. doi:10.1097/00001665-200307000-00017

111. Banzett RB, Guz A, Paydarfar D, Shea SA, Schachter SC, Lansing RW. Cardiorespiratory variables and sensation during stimulation of the left vagus in patients with epilepsy. *Epilepsy Res*. 1999;35(1):1-11. doi:10.1016/s0920-1211(98)00126-0

112. Baptist J, Shakya S, Ongole R. Rebamipide to Manage Stomatopyrosis in Oral Submucous Fibrosis. *J Contemp Dent Pract*. 2016;17(12 CC-Oral Health):1009‐1012. https://www.cochranelibrary.com/central/doi/10.1002/central/CN-01419696/full

113. Barbosa CMG, Terra-Filho M, de Albuquerque ALP, et al. Burnt sugarcane harvesting - cardiovascular effects on a group of healthy workers, Brazil. *PLoS One*. 2012;7(9):e46142. doi:10.1371/journal.pone.0046142

114. Barillo DJ, Harvey KD, Hobbs CL, Mozingo DW, Cioffi WG, Pruitt BAJ. Prospective outcome analysis of a protocol for the surgical and rehabilitative management of burns to the hands. *Plast Reconstr Surg*. 1997;100(6):1442-1451. doi:10.1097/00006534-199711000-00010

115. Bar-Meir E, Yaffe B, Winkler E, Sher N, Berenstein M, Schindler A. Combined Iliazarov and free flap for severe recurrent flexion-contracture release. *J Burn care Res Off Publ Am Burn Assoc*. 2006;27(4):529-534. doi:10.1097/01.BCR.0000226033.45478.2D

116. Barnes A, Sullivan J, Pappas E, Adams R, Burns J. Clinical and Functional Characteristics of People With Chronic and Recent-Onset Plantar Heel Pain. *PM R J Inj Funct Rehabil*. 2017;9(11):1128-1134. doi:10.1016/j.pmrj.2017.04.009

117. Barnes H, Yeoh HL, Fothergill T, Burns A, Humbert M, Williams T. Prostacyclin for pulmonary arterial hypertension. *Cochrane database Syst Rev*. 2019;5(5):CD012785. doi:10.1002/14651858.CD012785.pub2

118. Barnes JN, Burns JM, Bamman MM, et al. Proceedings from the Albert Charitable Trust Inaugural Workshop on “Understanding the Acute Effects of Exercise on the Brain”. *Brain Plast (Amsterdam, Netherlands)*. 2022;8(2):153-168. doi:10.3233/BPL-220146

119. Barnes TA, Catino ME, Burns EC, et al. Comparison of an oxygen-powered flow-limited resuscitator to manual ventilation with an adult 1,000-mL self-inflating bag. *Respir Care*. 2005;50(11):1445-1450.

120. Barnett SS, Smolinski P, Vorp DA. A Three-Dimensional Finite Element Analysis of Heat Transfer in the Forearm. *Comput Methods Biomech Biomed Engin*. 2000;3(4):287-296. doi:10.1080/10255840008915272

121. Barsch F, Mamilos A, Schmitt VH, et al. In Vivo Comparison of Synthetic Macroporous Filamentous and Sponge-like Skin Substitute Matrices Reveals Morphometric Features of the Foreign Body Reaction According to 3D Biomaterial Designs. *CELLS*. 2022;11(18). doi:10.3390/cells11182834

122. Basha MA, Abdel-Aal NM, Kamel FAH. Effects of Wii Fit Rehabilitation on Lower Extremity Functional Status in Adults With Severe Burns: a Randomized Controlled Trial. *Arch Phys Med Rehabil*. 2022;103(2):289‐296. doi:10.1016/j.apmr.2021.08.020

123. Basha MA, Aboelnour NH, Aly SM, Kamel FAH. Impact of Kinect-based virtual reality training on physical fitness and quality of life in severely burned children: a monocentric randomized controlled trial. *Ann Phys Rehabil Med*. 2022;65(1):101471. doi:10.1016/j.rehab.2020.101471

124. Basha MA, Azab AR, Elnaggar RK, et al. Inspiratory muscle training impact on respiratory muscle strength, pulmonary function, and quality of life in children with chest burn: A randomized controlled trial. *Burns*. 2024;50(7):1916-1924. doi:10.1016/j.burns.2024.05.007

125. Bassareo PP, Mercuro G. Pediatric hypertension: An update on a burning problem. *World J Cardiol*. 2014;6(5):253-259. doi:10.4330/wjc.v6.i5.253

126. Bassler TJ. Marathon running and immunity to atherosclerosis. *Ann N Y Acad Sci*. 1977;301:579-592. doi:10.1111/j.1749-6632.1977.tb38231.x

127. Basti S, Mathur U. Unusual intermediate-term outcome in three cases of limbal autograft transplantation. *Ophthalmology*. 1999;106(5):958-963. doi:10.1016/S0161-6420(99)00516-3

128. Bastide GMGBH, Remund AL, Oosthuizen DN, Derron N, Gerber PA, Weber IC. Handheld device quantifies breath acetone for real-life metabolic health monitoring. *Sensors & diagnostics*. 2023;2(4):918-928. doi:10.1039/d3sd00079f

129. Battistini A. [The pediatrician facing the problem of polluted air]. *Acta bio-medica L’Ateneo Parm organo della Soc di Med e Sci Nat di Parma*. 2000;71(3-4):27-36.

130. Bauer C, Graf C, Platschek AM, Struder HK, Ferrari N. Reasons, Motivational Factors, and Perceived Personal Barriers to Engagement in Physical Activity During Pregnancy Vary Within the BMI Classes: the Prenatal Prevention Project Germany. *J Phys Act Health*. 2018;15(3):204‐211. doi:10.1123/jpah.2016-0563

131. Baux S. [Burn sequelae]. *Rev Prat*. 1980;30(9):577-580,585-588.

132. Beach C, Billstrom G, Steeves ETA, Flynn JI, Steeves JA. The Physical Activity Patterns of Greenway Users Playing Pokemon Go: A Natural Experiment. *Games Health J*. 2019;8(1):7-14. doi:10.1089/g4h.2017.0168

133. Beaulieu LD, Blanchette AK, Mercier C, Bernard-Larocque V, Milot MH. Efficacy, safety, and tolerability of bilateral transcranial direct current stimulation combined to a resistance training program in chronic stroke survivors: a double-blind, randomized, placebo-controlled pilot study. *Restor Neurol Neurosci*. 2019;37(4 CC-Stroke):333‐346. doi:10.3233/RNN-190908

134. Bebbington E, Miles J, Peck M, Singer Y, Dunn K, Young A. Exploring the similarities and differences of variables collected by burn registers globally: protocol for a data dictionary review study. *BMJ Open*. 2023;13(2):e066512. doi:10.1136/bmjopen-2022-066512

135. Becker FC. Acute low back pain: diagnostics and treatment. *Clin Excell Nurse Pract*. 2001;5(2):80-84. doi:10.1054/xc.2001.19730

136. Becker JM, Iskandrian S, Conkling J. Fatal and near-fatal asthma in children exposed to fireworks. *Ann allergy, asthma Immunol Off Publ Am Coll Allergy, Asthma, Immunol*. 2000;85(6 Pt 1):512-513. doi:10.1016/S1081-1206(10)62581-1

137. Beebe LH, Harris RF. Description of physical activity in outpatients with schizophrenia spectrum disorders. *Int J Ment Health Nurs*. 2013;22(5):430-436. doi:10.1111/inm.12008

138. Begg L, McLaughlin P, Vicaretti M, Fletcher J, Burns J. Total contact cast wall load in patients with a plantar forefoot ulcer and diabetes. *J Foot Ankle Res*. 2016;9:2. doi:10.1186/s13047-015-0119-0

139. Bell CL, LaCroix A, Masaki K, et al. Prestroke factors associated with poststroke mortality and recovery in older women in the Women’s Health Initiative. *J Am Geriatr Soc*. 2013;61(8):1324-1330. doi:10.1111/jgs.12361

140. Belur J, Tilley N, Osrin D, Daruwalla N, Kumar M, Tiwari V. Police investigations: discretion denied yet undeniably exercised. *Polic Soc*. 2014;25(5):439-462. doi:10.1080/10439463.2013.878343

141. Belval LN, Cramer MN, Moralez G, et al. Interaction of Exercise Intensity and Simulated Burn Injury Size on Thermoregulation. *Med Sci Sports Exerc*. 2021;53(2):367-374. doi:10.1249/MSS.0000000000002480

142. Belval LN, Cramer MN, Moralez G, et al. Burn size and environmental conditions modify thermoregulatory responses to exercise in burn survivors. *J Burn care Res Off Publ Am Burn Assoc*. 2024;45(1):227-233. doi:10.1093/jbcr/irad128

143. Benjamin NC, Andersen CR, Herndon DN, Suman OE. The effect of lower body burns on physical function. *Burns*. 2015;41(8):1653‐1659. doi:10.1016/j.burns.2015.05.020

144. Benjamin NC, Andersen CR, Herndon DN, Suman OE. The effect of lower body burns on physical function. *Burns*. 2015;41(8):1653-1659. doi:10.1016/j.burns.2015.05.020

145. Benjamin NC, Andersen CR, Herndon DN, Suman OE. The effect of lower body burns on physical function. *Burns*. 2018;41(8):1653-1659. doi:10.1016/j.burns.2015.05.020

146. Benka-Coker ML, Clark ML, Rajkumar S, et al. Exposure to Household Air Pollution from Biomass Cookstoves and Levels of Fractional Exhaled Nitric Oxide (FeNO) among Honduran Women. *Int J Environ Res Public Health*. 2018;15(11). doi:10.3390/ijerph15112544

147. Benka-Coker ML, Clark ML, Rajkumar S, et al. Household air pollution from wood-burning cookstoves and C-reactive protein among women in rural Honduras. *Int J Hyg Environ Health*. 2022;241. doi:10.1016/j.ijheh.2022.113949

148. Bennett FE. An exercise in professional criticism. *J Am Pharm Assoc*. 1968;8(6):300-301. doi:10.1016/s0003-0465(16)30638-3

149. Bennett GB, Helm P, Purdue GF, Hunt JL. Serial casting: a method for treating burn contractures. *J Burn Care Rehabil*. 1989;10(6):543-545.

150. Bennett HG, Dahl LA, Furness J, Kemp-Smith K, Climstein M. Skin cancer and sun protective behaviours in water-based sports: A scoping review. *Photodermatol Photoimmunol Photomed*. 2022;38(3):197-214. doi:10.1111/phpp.12737

151. Bennett T, Birmingham AT, Fentem PH, Fitton D, Goldsmith R, Mason KD. Physiological and medical aspects of a relay cross-Channel swim. *J Sports Med Phys Fitness*. 1973;13(4):253-265.

152. Ben-Shlomo A, Sheppard MC, Stephens JM, Pulgar S, Melmed S. Clinical, quality of life, and economic value of acromegaly disease control. *Pituitary*. 2011;14(3):284-294. doi:10.1007/s11102-011-0310-7

153. Benson LN, Burns R, Schwaiger M, et al. Radionuclide angiographic evaluation of ventricular function in isolated congenitally corrected transposition of the great arteries. *Am J Cardiol*. 1986;58(3):319-324. doi:10.1016/0002-9149(86)90070-6

154. Bentley CL, Powell L, Potter S, et al. The Use of a Smartphone App and an Activity Tracker to Promote Physical Activity in the Management of Chronic Obstructive Pulmonary Disease: Randomized Controlled Feasibility Study. *JMIR mHealth uHealth*. 2020;8(6):e16203. doi:10.2196/16203

155. Bentley C, Hazeldine J, Greig C, Lord J, Foster M. Dehydroepiandrosterone: a potential therapeutic agent in the treatment and rehabilitation of the traumatically injured patient. *Burn trauma*. 2019;7:26. doi:10.1186/s41038-019-0158-z

156. Berarducci A, PA B, CA L, Sellers E. Health-promoting educational practices related to osteoporosis. *Appl Nurs Res*. 2000;13(4):173-180. https://search.ebscohost.com/login.aspx?direct=true&AuthType=cookie,ip,shib,uid&db=cul&AN=106995524&site=ehost-live&scope=site

157. Bergen G, Stevens MR, Burns ER. Falls and Fall Injuries Among Adults Aged ≥65 Years - United States, 2014. *MMWR Morb Mortal Wkly Rep*. 2016;65(37):993-998. doi:10.15585/mmwr.mm6537a2

158. Bergkamp D, Lenk J, Reynolds M, et al. Effectiveness of a burn rehabilitation workshop addressing confidence in therapy providers. *J Burn Care Res*. 2013;34(1):e10-4. doi:10.1097/BCR.0b013e3182644e82

159. Bergman SB, Yarkony GM, Stiens SA. Spinal cord injury rehabilitation. 2. Medical complications. *Arch Phys Med Rehabil*. 1997;78(3 Suppl):S53-8. doi:10.1016/s0003-9993(97)90410-x

160. Berketa J, James H, Langlois N, Richards L. The use of incinerated pig head in dental identification simulation. *J Forensic Odontostomatol*. 2015;33(2):1-8.

161. Berman DS, Kang X, Schisterman EF, et al. Serial changes on quantitative myocardial perfusion SPECT in patients undergoing revascularization or conservative therapy. *J Nucl Cardiol Off Publ Am Soc Nucl Cardiol*. 2001;8(4):428-437. doi:10.1067/mnc.2001.113991

162. Berman DS, Hachamovitch R, Shaw LJ, et al. Roles of nuclear cardiology, cardiac computed tomography, and cardiac magnetic resonance: Noninvasive risk stratification and a conceptual framework for the selection of noninvasive imaging tests in patients with known or suspected coronary artery diseas. *J Nucl Med*. 2006;47(7):1107-1118.

163. Berman DS, Kang X, Hayes SW, et al. Adenosine myocardial perfusion single-photon emission computed tomography in women compared with men. Impact of diabetes mellitus on incremental prognostic value and effect on patient management. *J Am Coll Cardiol*. 2003;41(7):1125-1133. doi:10.1016/s0735-1097(03)00085-8

164. Berman DS, Kang X, Nishina H, et al. Diagnostic accuracy of gated Tc-99m sestamibi stress myocardial perfusion SPECT with combined supine and prone acquisitions to detect coronary artery disease in obese and nonobese patients. *J Nucl Cardiol Off Publ Am Soc Nucl Cardiol*. 2006;13(2):191-201. doi:10.1007/BF02971243

165. Berman DS, Shaw LJ, Hachamovitch R, et al. Comparative use of radionuclide stress testing, coronary artery calcium scanning, and noninvasive coronary angiography for diagnostic and prognostic cardiac assessment. *Semin Nucl Med*. 2007;37(1):2-16. doi:10.1053/j.semnuclmed.2006.08.002

166. Berman DS, Wong ND, Gransar H, et al. Relationship between stress-induced myocardial ischemia and atherosclerosis measured by coronary calcium tomography. *J Am Coll Cardiol*. 2004;44(4):923-930. doi:10.1016/j.jacc.2004.06.042

167. Berman D, Kang X, Van Train K, et al. Comparative prognostic value of automatic quantitative analysis versus semiquantitative visual analysis of exercise myocardial perfusion single-photon emission computed tomography. *J Am Coll Cardiol*. 1998;32(7):1987-1995. doi:10.1016/S0735-1097(98)00501-4

168. Bernardes RA, Caldeira S, Parreira P, et al. Foot and Ankle Disorders in Nurses Exposed to Prolonged Standing Environments: A Scoping Review. *Workplace Health Saf*. 2023;71(3):101-116. doi:10.1177/21650799221137646

169. Bertozzi G, Salerno M, Pomara C, Sessa F. Neuropsychiatric and Behavioral Involvement in AAS Abusers. A Literature Review. *Medicina (Kaunas)*. 2019;55(7). doi:10.3390/medicina55070396

170. Bhadage CJ, Umarji HR, Shah K, Välimaa H. Vasodilator isoxsuprine alleviates symptoms of oral submucous fibrosis. *Clin Oral Investig*. 2013;17(5 CC-Oral Health):1375‐1382. doi:10.1007/s00784-012-0824-z

171. Bhat SG, Nagaraj M, Balentine C, et al. Assessing a Structured Mental Fitness Program for Academic Acute Care Surgeons: A Pilot Study. *J Surg Res*. 2024;295:9-18. doi:10.1016/j.jss.2023.09.052

172. Bi YX, Liu XJ, Liu Y, et al. Molecular and biochemical investigations of the anti-fatigue effects of tea polyphenols and fruit extracts of *Lycium ruthenicum* Murr*.* on mice with exercise-induced fatigue. *Front Mol Biosci*. 2023;10. doi:10.3389/fmolb.2023.1223411

173. Bilic A, Burns RD, Bai Y, Brusseau TA, Lucero JE, Jensen JLK. Preliminary Efficacy of a Multi-Behavioral Zoom-Based Peer Health Coaching Intervention in Young Adults: A Stepped Wedge Randomized Controlled Trial. *CYBERPSYCHOLOGY Behav Soc Netw*. 2023;26(9):698-705. doi:10.1089/cyber.2022.0365

174. Billinger SA, Vidoni ED, Greer CS, Graves RS, Mattlage AE, Burns JM. Cardiopulmonary exercise testing is well tolerated in people with Alzheimer-related cognitive impairment. *Arch Phys Med Rehabil*. 2014;95(9):1714-1718. doi:10.1016/j.apmr.2014.04.007

175. Billinger SA, Vidoni ED, Honea RA, Burns JM. Cardiorespiratory Response to Exercise Testing in Individuals With Alzheimer’s Disease. *Arch Phys Med Rehabil*. 2011;92(12):2000-2005. doi:10.1016/j.apmr.2011.07.194

176. Billinger SA, Vidoni ED, Morris JK, Thyfault JP, Burns JM. Exercise Test Performance Reveals Evidence of the Cardiorespiratory Fitness Hypothesis. *J Aging Phys Act*. 2017;25(2):240-246. doi:10.1123/japa.2015-0321

177. Bina S, Pacey V, Barnes EH, Burns J, Gray K. Interventions for congenital talipes equinovarus (clubfoot). *Cochrane database Syst Rev*. 2020;5(5):CD008602. doi:10.1002/14651858.CD008602.pub4

178. Binder R. Malpractice--in dermatology. *Cutis*. 1979;23(5):663-666.

179. Biolo G, Tipton KD, Klein S, Wolfe RR. An abundant supply of amino acids enhances the metabolic effect of exercise on muscle protein. *Am J Physiol*. 1997;273(1 Pt 1):E122-9. doi:10.1152/ajpendo.1997.273.1.E122

180. Biolo G, Williams BD, Fleming RYD, et al. Insulin action on muscle protein kinetics and amino acid transport during recovery after resistance exercise. *Diabetes*. 1999;48(5):949-957. doi:10.2337/diabetes.48.5.949

181. Birch JR, Eakins B, Gosen J, Green S, Morton M. Musculoskeletal management of the severely burned child. *Can Med Assoc J*. 1976;115(6):533-536.

182. Birch L, English CA, Burns M, Keer JT. Generic scheme for independent performance assessment in the molecular biology laboratory. *Clin Chem*. 2004;50(9):1553-1559. doi:10.1373/clinchem.2003.029454

183. Bird JD, Leacy JK, Foster GE, et al. Time course and magnitude of ventilatory and renal acid-base acclimatization following rapid ascent to and residence at 3,800 m over nine days. *J Appl Physiol*. 2021;130(6):1705-1715. doi:10.1152/japplphysiol.00973.2020

184. Biring MS, Fournier M, Ross DJ, Lewis MI. Cellular adaptations of skeletal muscles to cyclosporine. *J Appl Physiol*. 1998;84(6):1967-1975. doi:10.1152/jappl.1998.84.6.1967

185. Björnhagen V, Schüldt Ekholm K, Larsen F, Ekholm J. BURN SURVIVORS’ PULMONARY AND MUSCULAR IMPAIRMENT, EXERCISE TOLERANCE AND RETURN-TO-WORK FOLLOWING MEDICAL-VOCATIONAL REHABILITATION: A LONG-TERM FOLLOW-UP. *J Rehabil Med*. 2018;50(5):465-471. doi:10.2340/16501977-2337

186. Black S, Carter GM, Nitz AJ, Worthington JA. Oxygen consumption for lower extremity exercises in normal subjects and burned patients. *Phys Ther*. 1980;60(10):1255-1258. doi:10.1093/ptj/60.10.1255

187. Blake RL, Burns DP, Colson JP. Etiology of atraumatic medial knee pain. *J Am Podiatry Assoc*. 1981;71(10):580-583. doi:10.7547/87507315-71-10-580

188. Blashill AJ, Williams A, Grogan S, Clark-Carter D. Negative appearance evaluation is associated with skin cancer risk behaviors among American men and women. *Heal Psychol Off J Div Heal Psychol Am Psychol Assoc*. 2015;34(1):93-96. doi:10.1037/hea0000100

189. Blassingame WM, Bennett GB, Helm PA, Purdue GF, Hunt JL. Range of motion of the shoulder performed while patient is anesthetized. *J Burn Care Rehabil*. 1989;10(6):539-542. doi:10.1097/00004630-198911000-00016

190. Bleecker ML. Carbon monoxide intoxication. *Handb Clin Neurol*. 2015;131:191-203. doi:10.1016/B978-0-444-62627-1.00024-X

191. Bléher M, Meshko B, Cacciapuoti I, et al. *Egr1* loss-of-function promotes beige adipocyte differentiation and activation specifically in inguinal subcutaneous white adipose tissue. *Sci Rep*. 2020;10(1). doi:10.1038/s41598-020-72698-w

192. Blocker EM, Fry AC, Luebbers PE, et al. Promoting Alzheimer’s Risk-Reduction through Community-Based Lifestyle Education and Exercise in Rural America: A Pilot Intervention. *Kansas J Med*. 2020;13:179-185.

193. Blokstra A, Burns CM, Seidell JC. Perception of weight status and dieting behaviour in Dutch men and women. *Int J Obes Relat Metab Disord J Int Assoc Study Obes*. 1999;23(1):7-17. doi:10.1038/sj.ijo.0800803

194. Blyton F, Chuter V, Walter KEL, Burns J. Non-drug therapies for lower limb muscle cramps. *Cochrane database Syst Rev*. 2012;1(1):CD008496. doi:10.1002/14651858.CD008496.pub2

195. Boczar D, Seu M, O’Connell A, et al. Hand Therapy Regimen for Functional Recovery Following Combined Face and Bilateral Hand Transplantation. *Hand (N Y)*. 2023;18(3):NP7-NP15. doi:10.1177/15589447221124250

196. Bodekaer M, Faurschou A, Philipsen PA, Wulf HC. Sun protection factor persistence during a day with physical activity and bathing. *Photodermatol Photoimmunol Photomed*. 2008;24(6):296-300. doi:10.1111/j.1600-0781.2008.00379.x

197. Boeselt T, Spielmanns M, Nell C, et al. Validity and Usability of Physical Activity Monitoring in Patients with Chronic Obstructive Pulmonary Disease (COPD). *PLoS One*. 2016;11(6):e0157229. doi:10.1371/journal.pone.0157229

198. Bolger LA, Bolger LE, O’Neill C, et al. Fundamental Movement Skill Proficiency and Health Among a Cohort of Irish Primary School Children. *Res Q Exerc Sport*. 2019;90(1):24-35. doi:10.1080/02701367.2018.1563271

199. Bolger LE, Bolger LA, O’Neill C, et al. Global levels of fundamental motor skills in children: A systematic review. *J Sports Sci*. 2021;39(7):717-753. doi:10.1080/02640414.2020.1841405

200. Bombardier CH, Dyer JR, Burns P, et al. A tele-health intervention to increase physical fitness in people with spinal cord injury and cardiometabolic disease or risk factors: a pilot randomized controlled trial. *Spinal Cord*. 2021;59(1):63-73. doi:10.1038/s41393-020-0523-6

201. Bombardier CH, Fann JR, Ehde DM, et al. Collaborative Care Versus Usual Care to Improve Quality of Life, Pain, Depression, and Physical Activity in Outpatients With Spinal Cord Injury: The SCI-CARE Randomized Controlled Clinical Trial. *J Neurotrauma*. 2023;40(23-24):2667-2679. doi:10.1089/neu.2023.0200

202. Bonetti G, Herbst KL, Dhuli K, et al. Dietary supplements for lipedema. *J Prev Med Hyg*. 2022;63(2 Suppl 3):E169-E173. doi:10.15167/2421-4248/jpmh2022.63.2S3.2758

203. Bonnecaze AK, Reynolds P, Burns CA. Stress-Dosed Glucocorticoids and Mineralocorticoids Before Intensive Endurance Exercise in Primary Adrenal Insufficiency. *Clin J Sport Med*. 2019;29(6):E73-E75. doi:10.1097/JSM.0000000000000540

204. Booth B, Furzeland J. An unusual rash for Royal: a case series. *J R Nav Med Serv*. 2016;102(1):19-21. doi:10.1136/jrnms-102-19

205. Borgerding MF, Hicks RD, Bodnar JE, et al. Cigarette smoke composition. Part 1. Limitations of FTC method when applied to cigarettes that heat instead of burn tobacco. *J Assoc Off Anal Chem*. 1990;73(4):605-609.

206. Borisova AI. [Exercise therapy for the prevention and complex treatment of neck contractures after burns in children]. *Vopr Kurortol Fizioter Lech Fiz Kult*. 1976;(5):46-48.

207. Borisova AI. [Role of exercise therapy and its methods at different periods of burn diseases]. *Klin Khir*. 1976;(6):20-22.

208. Borisova AI. [Medical gymnastics in the rehabilitative period in children who have had burn disease]. *Vopr Kurortol Fizioter Lech Fiz Kult*. 1980;(3):37-39.

209. Börzsei D, Szabó R, Hoffmann A, et al. Multiple Applications of Different Exercise Modalities with Rodents. *Oxid Med Cell Longev*. 2021;2021:3898710. doi:10.1155/2021/3898710

210. Boschetti L, Stehman S V, Roy DP. A stratified random sampling design in space and time for regional to global scale burned area product validation. *Remote Sens Environ*. 2016;186:465-478. doi:10.1016/j.rse.2016.09.016

211. Bösner S, Haasenritter J, Becker A, et al. Heartburn or angina? Differentiating gastrointestinal disease in primary care patients presenting with chest pain: a cross sectional diagnostic study. *Int Arch Med*. 2009;2:40. doi:10.1186/1755-7682-2-40

212. Boswick JAJ. Comprehensive rehabilitation after burn injury. *Surg Clin North Am*. 1987;67(1):159-166. doi:10.1016/s0039-6109(16)44139-3

213. Boswick JAJ. Rehabilitation of the burned hand. *Clin Orthop Relat Res*. 1974;(104):162-174. doi:10.1097/00003086-197410000-00017

214. Boswick JAJ. Rehabilitation after burn injury. *Ann Acad Med Singapore*. 1983;12(3):443-448.

215. Bottoni U, Tamburrini S, Scali E, et al. Neurodystrophic Hand Dermatitis: Sannino Barduagni Syndrome. *Acta Dermatovenerol Croat*. 2017;25(4):312-313.

216. Boyd J, Paratz J, Tronstad O, Caruana L, Walsh J. Exercise is feasible in patients receiving vasoactive medication in a cardiac surgical intensive care unit: A prospective observational study. *Aust Crit CARE*. 2020;33(3):244-249. doi:10.1016/j.aucc.2020.02.004

217. Boyd J, Paratz J, Tronstad O, Caruana L, McCormack P, Walsh J. When is it safe to exercise mechanically ventilated patients in the intensive care unit? An evaluation of consensus recommendations in a cardiothoracic setting. *Hear LUNG*. 2018;47(2):81-86. doi:10.1016/j.hrtlng.2017.11.006

218. Boyer MI, Gelberman RH, Burns ME, Dinopoulos H, Hofem R, Silva MJ. Intrasynovial flexor tendon repair. An experimental study comparing low and high levels of in vivo force during rehabilitation in canines. *J Bone Joint Surg Am*. 2001;83(6):891-899.

219. Boyer P, Burns D, Whyne C. Out-of-Distribution Detection of Human Activity Recognition with Smartwatch Inertial Sensors. *Sensors (Basel)*. 2021;21(5). doi:10.3390/s21051669

220. Boyer P, Burns D, Whyne C. Evaluation of at-home physiotherapy. *Bone Joint Res*. 2023;12(3):165-177. doi:10.1302/2046-3758.123.BJR-2022-0126.R1

221. Brant JM. Breathlessness with pulmonary metastases: a multimodal approach. *J Adv Pract Oncol*. 2013;4(6):415-422.

222. Brauer SG, Burns YR, Galley P. A prospective study of laboratory and clinical measures of postural stability to predict community-dwelling fallers. *J Gerontol A Biol Sci Med Sci*. 2000;55(8):M469-76. doi:10.1093/gerona/55.8.m469

223. Bray R, Forrester K, Leonard C, McArthur R, Tulip J, Lindsay R. Laser Doppler imaging of burn scars: a comparison of wavelength and scanning methods. *Burns*. 2003;29(3):199-206. doi:10.1016/s0305-4179(02)00307-8

224. Breisblatt WM, Weiland FL, McLain JR, Tomlinson GC, Burns MJ, Spaccavento LJ. Usefulness of ambulatory radionuclide monitoring of left ventricular function early after acute myocardial infarction for predicting residual myocardial ischemia. *Am J Cardiol*. 1988;62(16):1005-1010. doi:10.1016/0002-9149(88)90538-3

225. Breland JY, McAndrew LM, Burns E, Leventhal EA, Leventhal H. Using the Common Sense Model of Self-regulation to review the effects of self-monitoring of blood glucose on glycemic control for non-insulin-treated adults with type 2 diabetes. *Diabetes Educ*. 2013;39(4):541-559. doi:10.1177/0145721713490079

226. Brema I, Hatunic M, Finucane F, et al. Plasma visfatin is reduced after aerobic exercise in early onset type 2 diabetes mellitus. *Diabetes Obes Metab*. 2008;10(7):600-602. doi:10.1111/j.1463-1326.2008.00872.x

227. Breuninger H, Veihelmann D, Domres B. [Indications for percutaneous fracture fixation in bone surgery]. *Fortschr Med*. 1977;95(25):1653-1658.

228. Brewster LP, Bennett BK, Gamelli RL. Application of rehabilitation ethics to a selected burn patient population’s perspective. *J Am Coll Surg*. 2006;203(5):766-771. doi:10.1016/j.jamcollsurg.2006.06.024

229. Briggs AL, Katzer R, Gonzalez IA, Boysen-Osborn M. Two-Screen Virtual Board Game Didactic for Teaching Wilderness and Environmental Medicine Topics to Emergency Medicine Residents. *J Educ Teach Emerg Med*. 2021;6(4):L1-L6. doi:10.21980/J8J343

230. Brink C, Isaacs Q, Scriba MF, Nathire MEH, Rode H, Martinez R. Infant burns: A single institution retrospective review. *Burns*. 2019;45(7):1518-1527. doi:10.1016/j.burns.2018.11.005

231. Brink Y, Brooker H, Carstens E, Gissing CA, Langtree C. Effectiveness of resistance strength training in children and adolescents with ≥30% total body surface area: A systematic review. *South African J Physiother*. 2016;72(1):1-8. doi:10.4102/sajp.v72i1.303

232. Brodaty H, Burns K. Nonpharmacological management of apathy in dementia: a systematic review. *Am J Geriatr Psychiatry*. 2012;20(7):549-564. doi:10.1097/JGP.0b013e31822be242

233. Brodfuehrer PD, Burns A. Neuronal factors influencing the decision to swim in the medicinal leech. *Neurobiol Learn Mem*. 1995;63(2):192-199. doi:10.1006/nlme.1995.1020

234. Brodfuehrer PD, Parker HJ, Burns A, Berg M. Regulation of the segmental swim-generating system by a pair of identified interneurons in the leech head ganglion. *J Neurophysiol*. 1995;73(3):983-992. doi:10.1152/jn.1995.73.3.983

235. Broemeling LD, Wolfe RR. Measuring intrasubject variability: use of the jacknife in doubly labeled water experiments. *J Appl Physiol*. 1993;75(4):1507-1512. doi:10.1152/jappl.1993.75.4.1507

236. Broom DR, Stensel DJ, Bishop NC, Burns SF, Miyashita M. Exercise-induced suppression of acylated ghrelin in humans. *J Appl Physiol*. 2007;102(6):2165-2171. doi:10.1152/japplphysiol.00759.2006

237. Brown AC. An overview of herb and dietary supplement efficacy, safety and government regulations in the United States with suggested improvements. Part 1 of 5 series. *Food Chem Toxicol an Int J Publ Br Ind Biol Res Assoc*. 2017;107(Pt A):449-471. doi:10.1016/j.fct.2016.11.001

238. Brown MC, Araújo-Soares V, Skinner R, et al. Using qualitative and co-design methods to inform the development of an intervention to support and improve physical activity in childhood cancer survivors: a study protocol for BEing Active after ChildhOod caNcer (BEACON). *BMJ Open*. 2020;10(12). doi:10.1136/bmjopen-2020-041073

239. Brownjb. The closure of surface defects with free skin grafts and with pedicle flaps. *Woman Physician*. 1971;26(2):75-77.

240. Bruce J, Lall R, Withers EJ, et al. A cluster randomised controlled trial of advice, exercise or multifactorial assessment to prevent falls and fractures in community-dwelling older adults: protocol for the prevention of falls injury trial (PreFIT). *BMJ Open*. 2016;6(1):e009362. doi:10.1136/bmjopen-2015-009362

241. Bruce-Hickman D, Jiang X, Thia JJP, Kansal A. Stevens-Johnson Syndrome complicated by obstructive uropathy, pneumothorax, and pneumomediastinum: a case report and literature review. *Burn trauma*. 2019;7:14. doi:10.1186/s41038-019-0153-4

242. Bruehl S, Burns JW, Koltyn K, et al. Are endogenous opioid mechanisms involved in the effects of aerobic exercise training on chronic low back pain? A randomized controlled trial. *Pain*. 2020;161(12):2887-2897. doi:10.1097/j.pain.0000000000001969

243. Bruehl S, Burns JW, Koltyn K, et al. Does aerobic exercise training alter responses to opioid analgesics in individuals with chronic low back pain? A randomized controlled trial. *Pain*. 2021;162(8):2204-2213. doi:10.1097/j.pain.0000000000002165

244. Bruehl S, Burns JW, Morgan A, et al. The association between endogenous opioid function and morphine responsiveness: a moderating role for endocannabinoids. *Pain*. 2019;160(3):676-687. doi:10.1097/j.pain.0000000000001447

245. Brüggen MC, Le ST, Walsh S, et al. Supportive care in the acute phase of Stevens-Johnson syndrome and toxic epidermal necrolysis: an international, multidisciplinary Delphi-based consensus. *Br J Dermatol*. 2021;185(3):616-626. doi:10.1111/bjd.19893

246. Brunner J, Singh AK, Rocha T, Havens J, Goralnick E, Sodickson A. Terrorist bombings: foreign bodies from the Boston Marathon bombing. *Semin Ultrasound CT MR*. 2015;36(1):68-72. doi:10.1053/j.sult.2014.10.006

247. Brusseau TA, Hannon JC, Fu Y, et al. Trends in physical activity, health-related fitness, and gross motor skills in children during a two-year comprehensive school physical activity program. *J Sci Med Sport*. 2018;21(8):828-832. doi:10.1016/j.jsams.2017.12.015

248. Brusseau TA, Burns RD. Associations of Physical Activity, School Safety, and Non-Prescription Steroid Use in Adolescents: A Structural Equation Modeling Approach. *Int J Environ Res Public Health*. 2021;19(1). doi:10.3390/ijerph19010087

249. Brusseau TA, Burns RD. Children’s Weight Gain and Cardiovascular Fitness Loss over the Summer. *Int J Environ Res Public Health*. 2018;15(12). doi:10.3390/ijerph15122770

250. Brusseau TA, Burns RD. Physical Activity, Health-Related Fitness, and Classroom Behavior in Children: A Discriminant Function Analysis. *Res Q Exerc Sport*. 2018;89(4):411-417. doi:10.1080/02701367.2018.1519521

251. Brusseau TA, Burns RD, Fu Y. Contextual factors related to physical activity during daily middle school physical education. *J Sci Med Sport*. 2016;19(9):733-737. doi:10.1016/j.jsams.2015.10.001

252. Brusseau TA, Burns RD, Fu Y, Weaver RG. Impact of Year-Round and Traditional School Schedules on Summer Weight Gain and Fitness Loss. *Child Obes*. 2019;15(8):541-547. doi:10.1089/chi.2019.0070

253. Brusseau TA, Burns RD, Hannon JC. Physical Activity and Health-Related Fitness of Adolescents within the Juvenile Justice System. *Biomed Res Int*. 2018;2018:9710714. doi:10.1155/2018/9710714

254. Brusseau TA, Burns RD, Hannon JC. Trends in Sedentary and Physical Activity Behaviors in Incarcerated Adolescent Boys During a Sports, Play, and Recreation for Kids Program. *Am J Heal Promot*. 2019;33(5):760-763. doi:10.1177/0890117118812666

255. Brusseau TA, Hannon J, Burns R. The Effect of a Comprehensive School Physical Activity Program on Physical Activity and Health-Related Fitness in Children From Low-Income Families. *J Phys Act Health*. 2016;13(8):888-894. doi:10.1123/jpah.2016-0028

256. Buchthal OV, Doff AL, Hsu LA, Silbanuz A, Heinrich KM, Maddock JE. Avoiding a knowledge gap in a multiethnic statewide social marketing campaign: is cultural tailoring sufficient? *J Health Commun*. 2011;16(3):314-327. doi:10.1080/10810730.2010.535111

257. Bunney PE, Zink AN, Holm AA, Billington CJ, Kotz CM. Orexin activation counteracts decreases in nonexercise activity thermogenesis (NEAT) caused by high-fat diet. *Physiol Behav*. 2017;176:139-148. doi:10.1016/j.physbeh.2017.03.040

258. Burd A, KW C, WS H, et al. Before the paradigm shift: concepts and communication between doctors and nurses in a burns team. *Burn*. 2002;28(7):691-695. doi:10.1016/s0305-4179(02)00095-5

259. Burd A, Kwok CH, Hung SC, et al. A comparative study of the cytotoxicity of silver-based dressings in monolayer cell, tissue explant, and animal models. *Wound repair Regen Off Publ Wound Heal Soc [and] Eur Tissue Repair Soc*. 2007;15(1):94-104. doi:10.1111/j.1524-475X.2006.00190.x

260. Burgess HJ, Bahl S, Wilensky K, et al. A 4-week morning light treatment with stable sleep timing for individuals with fibromyalgia: a randomized controlled trial. *Pain Med*. 2023;24(7):787-795. doi:10.1093/pm/pnad007

261. BURGESS SG, BURNS D, TIDY CW. Investigations into swimming bath water treatment. *J R Sanit Inst*. 1953;73(2):123-142. doi:10.1177/146642405307300208

262. Burini RC, Borges-Santos MD, Moreto F, Yu YM. Comparative effects of acute-methionine loading on the plasma sulfur-amino acids in NAC-supplemented HIV plus patients and healthy controls. *Amino Acids*. 2018;50(5):569-576. doi:10.1007/s00726-018-2538-2

263. Burke LM. Re-Examining High-Fat Diets for Sports Performance: Did We Call the “Nail in the Coffin” Too Soon? *Sports Med*. 2015;45 Suppl 1(Suppl 1):S33-49. doi:10.1007/s40279-015-0393-9

264. Burkle FMJ, McGrady KA, Newett SL, et al. Complex, humanitarian emergencies: III. Measures of effectiveness. *Prehosp Disaster Med*. 1995;10(1):48-56. doi:10.1017/s1049023x00041662

265. Burnet G, Platnick C, Krishnan P, et al. Muffins and Meditation: Combatting Burnout in Surgical Residents. *J Surg Educ*. 2023;80(2):185-193. doi:10.1016/j.jsurg.2022.09.005

266. Burnett D, Smith K, Smeltzer C, Young K, Burns S. Perceived Muscle Soreness in Recreational Female Runners. *Int J Exerc Sci*. 2010;3(3):108-116.

267. Burnett DM, Burns S, Merritt S, Wick J, Sharpe M. Prevalence of Exercise-Induced Bronchoconstriction Measured by Standardized Testing in Healthy College Athletes. *Respir Care*. 2016;61(5):571-576. doi:10.4187/respcare.04493

268. Burns AM, Nixon A, Mallinson J, Cordon SM, Stephens FB, Greenhaff PL. Immobilisation induces sizeable and sustained reductions in forearm glucose uptake in just 24 h but does not change lipid uptake in healthy men. *J Physiol*. 2021;599(8):2197-2210. doi:10.1113/JP281021

269. Burns A, Gatlin DM. Effects of sustained swimming exercise on growth and body composition responses of Nile tilapia (*Oreochromis niloticus*), red drum (*Sciaenops ocellatus*), and hybrid striped bass (*Morone chrysops* x *M. saxatilis*). *FISH Physiol Biochem*. 2022;48(5):1401-1411. doi:10.1007/s10695-022-01129-6

270. Burns A, Iliffe S. Alzheimer’s disease. *BMJ*. 2009;338:b158. doi:10.1136/bmj.b158

271. Burns AMN, Erickson DH. Adding Cognitive Remediation to Employment Support Services: A Randomized Controlled Trial. *Psychiatr Serv*. 2023;74(3):222-228. doi:10.1176/appi.ps.202100249

272. Burns AS, Boyce VS, Tessler A, Lemay MA. Fibrillation potentials following spinal cord injury: improvement with neurotrophins and exercise. *Muscle Nerve*. 2007;35(5):607-613. doi:10.1002/mus.20738

273. Burns AS, Delparte JJ, Ballantyne EC, Boschen KA. Evaluation of an interdisciplinary program for chronic pain after spinal cord injury. *PM R J Inj Funct Rehabil*. 2013;5(10):832-838. doi:10.1016/j.pmrj.2013.05.004

274. Burns AS, Delparte JJ, Patrick M, Marino RJ, Ditunno JF. The reproducibility and convergent validity of the walking index for spinal cord injury (WISCI) in chronic spinal cord injury. *Neurorehabil Neural Repair*. 2011;25(2):149-157. doi:10.1177/1545968310376756

275. Burns AS, Jawaid S, Zhong H, et al. Paralysis elicited by spinal cord injury evokes selective disassembly of neuromuscular synapses with and without terminal sprouting in ankle flexors of the adult rat. *J Comp Neurol*. 2007;500(1):116-133. doi:10.1002/cne.21143

276. Burns CA, Sperry RE, Arrowood JA, Wood MA, Nixon J V, Ellenbogen KA. Doppler echocardiographic assessment of an impedance-based dual-chamber rate-responsive pacemaker. *Am J Cardiol*. 1993;71(7):569-574. doi:10.1016/0002-9149(93)90513-c

277. Burns CJ, LaKind JS, Naiman J, et al. Research on COVID-19 and air pollution: A path towards advancing exposure science. *Environ Res*. 2022;212. doi:10.1016/j.envres.2022.113240

278. Burns CM, Inglis AD. Measuring food access in Melbourne: access to healthy and fast foods by car, bus and foot in an urban municipality in Melbourne. *Health Place*. 2007;13(4):877-885. doi:10.1016/j.healthplace.2007.02.005

279. Burns CM, Richman R, Caterson ID. Nutrition knowledge in the obese and overweight. *Int J Obes*. 1987;11(5):485-492.

280. Burns CJ, LaKind JS. Elements to increase translation in pyrethroid epidemiology research: A review. *Sci Total Environ*. 2022;813:152568. doi:10.1016/j.scitotenv.2021.152568

281. Burns DC, Gollnick PD. An inexpensive floating-mesh electrode for EKG recording during exercise. *J Appl Physiol*. 1966;21(6):1889-1891. doi:10.1152/jappl.1966.21.6.1889

282. Burns DS, Clay KA, Bailey MS. Leptospirosis in a British soldier after travel to Borneo. *J R Army Med Corps*. 2016;162(6):473-475. doi:10.1136/jramc-2015-000533

283. Burns DS, Porter CK, Gutierrez RL, et al. Diarrhea and associated illness characteristics and risk factors among British active duty service members at Askari Storm training exercise, Nanyuki, Kenya, January-June 2014. *MSMR*. 2020;27(8):4-7.

284. Burns DM, Leung N, Hardisty M, Whyne CM, Henry P, McLachlin S. Shoulder physiotherapy exercise recognition: machine learning the inertial signals from a smartwatch. *Physiol Meas*. 2018;39(7):75007. doi:10.1088/1361-6579/aacfd9

285. Burns D, Boyer P, Razmjou H, Richards R, Whyne C. Adherence Patterns and Dose Response of Physiotherapy for Rotator Cuff Pathology: Longitudinal Cohort Study. *JMIR Rehabil Assist Technol*. 2021;8(1):e21374. doi:10.2196/21374

286. Burns D, Razmjou H, Shaw J, et al. Adherence Tracking With Smart Watches for Shoulder Physiotherapy in Rotator Cuff Pathology: Protocol for a Longitudinal Cohort Study. *JMIR Res Protoc*. 2020;9(7):e17841. doi:10.2196/17841

287. Burns EM, Quyn A, UKPEN LC, subcomm AAC. The “Pelvic exenteration lexicon”: Creating a common language for complex pelvic cancer surgery. *Color Dis*. 2023;25(5):888-896. doi:10.1111/codi.16476

288. Burns E, Nair S. New horizons in care home medicine. *Age Ageing*. 2014;43(1):2-7. doi:10.1093/ageing/aft186

289. Burns EM, Enns RM, Garrick DJ. The effect of simulated censored data on estimates of heritability of longevity in the Thoroughbred racing industry. *Genet Mol Res*. 2006;5(1):7-15.

290. Burns ER, Haddad YK, Parker EM. Primary care providers’ discussion of fall prevention approaches with their older adult patients-DocStyles, 2014. *Prev Med reports*. 2018;9:149-152. doi:10.1016/j.pmedr.2018.01.016

291. Burns EE, Carter LJ, Snape J, Thomas-Oates J, Boxall ABA. Application of prioritization approaches to optimize environmental monitoring and testing of pharmaceuticals. *J Toxicol Environ Health B Crit Rev*. 2018;21(3):115-141. doi:10.1080/10937404.2018.1465873

292. Burns F, Stevens PA, Pyne NJ. The identification of apparently novel cyclic AMP and cyclic GMP phosphodiesterase activities in guinea-pig tracheal smooth muscle. *Br J Pharmacol*. 1994;113(1):3-4. doi:10.1111/j.1476-5381.1994.tb16164.x

293. Burns FR. Protecting elders: regulating intergenerationally transmitted debt in Australia. *Int J Law Psychiatry*. 2005;28(3):300-319. doi:10.1016/j.ijlp.2004.02.001

294. Burns GP, Schenk WGJ. Effect of digestion and exercise on intestinal blood flow and cardiac output. An experimental study in the conscious dog. *Arch Surg*. 1969;98(6):790-794. doi:10.1001/archsurg.1969.01340120138026

295. Burns GT, Tam N, Santos-Concejero J, Tucker R, Zernicke RF. Assessing spring-mass similarity in elite and recreational runners. *Front Physiol*. 2023;14. doi:10.3389/fphys.2023.1224459

296. Burns GT, Zernicke RF. A simple computational method to estimate stance velocity in running. *J Exp Biol*. 2021;224(18). doi:10.1242/jeb.242787

297. Burns GT, Deneweth Zendler J, Zernicke RF. Validation of a wireless shoe insole for ground reaction force measurement. *J Sports Sci*. 2019;37(10):1129-1138. doi:10.1080/02640414.2018.1545515

298. Burns GT, Gonzalez R, Zendler JM, Zernicke RF. Bouncing behavior of sub-four minute milers. *Sci Rep*. 2021;11(1):10501. doi:10.1038/s41598-021-89858-1

299. Burns GT, Gonzalez R, Zernicke RF. Improving spring-mass parameter estimation in running using nonlinear regression methods. *J Exp Biol*. 2021;224(Pt 6). doi:10.1242/jeb.232850

300. Burns GT, Joubert DP. Running Shoes of the Postmodern Footwear Era: A Narrative Overview of Advanced Footwear Technology. *Int J Sports Physiol Perform*. 2024;19(10):975-986. doi:10.1123/ijspp.2023-0446

301. Burns GT, Tam N. Is it the shoes? A simple proposal for regulating footwear in road running. *Br J Sports Med*. 2020;54(8):439-440. doi:10.1136/bjsports-2018-100480

302. Burns GT, Tam N, Langerak NG, Zernicke RF, Lamberts RP. Spring-Mass Characteristics in Runners Before and After a 56-km Road Ultramarathon. *J Appl Biomech*. Published online November 2024:1-9. doi:10.1123/jab.2023-0281

303. Burns GT, Zendler JM, Zernicke RF. Step frequency patterns of elite ultramarathon runners during a 100-km road race. *J Appl Physiol*. 2019;126(2):462-468. doi:10.1152/japplphysiol.00374.2018

304. Burns G, Wilson CM. Rehabilitation for Rhabdomyolysis Associated With Breast Cancer Treatment. *Cureus*. 2020;12(6):e8625. doi:10.7759/cureus.8625

305. Burns G, Bianchi S. Chronic obstructive pulmonary disease: time to ditch the old mindset. *Br J Hosp Med (Lond)*. 2006;67(2):62-63. doi:10.12968/hmed.2006.67.2.20462

306. Burns J. Fitness campaign or fraud scheme? Postal inspector, state of California investigating promotion that may have cost hospitals $1 million. *Mod Healthc*. 1992;22(31):51-52,54,56.

307. Burns JJ, MacMillan KM, John E. Prevalence of Exercise-Induced Pulmonary Hemorrhage, Tracheal Mucus and Recurrent Laryngeal Neuropathy in Competitive Draft Pulling Horses. *J EQUINE Vet Sci*. 2023;129. doi:10.1016/j.jevs.2023.104895

308. Burns JM, Lestyk K, Freistroffer D, Hammill MO. Preparing Muscles for Diving: Age-Related Changes in Muscle Metabolic Profiles in Harp (Pagophilus groenlandicus) and Hooded (Cystophora cristata) Seals. *Physiol Biochem Zool*. 2015;88(2):167-182. doi:10.1086/680015

309. Burns JW, Johnson BJ, Devine J, Mahoney N, Pawl R. Anger management style and the prediction of treatment outcome among male and female chronic pain patients. *Behav Res Ther*. 1998;36(11):1051-1062. doi:10.1016/s0005-7967(98)00080-1

310. Burns JW, Mullen JT, Higdon LJ, Wei JM, Lansky D. Validity of the pain anxiety symptoms scale (PASS): prediction of physical capacity variables. *Pain*. 2000;84(2-3):247-252. doi:10.1016/S0304-3959(99)00218-3

311. Burns JW, Werchan PM, Fanton JW, Dollins AB. Performance recovery following +Gz-induced loss of consciousness. *Aviat Space Environ Med*. 1991;62(7):615-617.

312. Burns J, Begg L. Optimizing the offloading properties of the total contact cast for plantar foot ulceration. *Diabet Med*. 2011;28(2):179-185. doi:10.1111/j.1464-5491.2010.03135.x

313. Burns J, Wegener C, Begg L, Vicaretti M, Fletcher J. Randomized trial of custom orthoses and footwear on foot pain and plantar pressure in diabetic peripheral arterial disease. *Diabet Med*. 2009;26(9):893-899. doi:10.1111/j.1464-5491.2009.02799.x

314. Burns JM, Peiffer JJ, Abbiss CR, Watson G, Burnett A, Laursen PB. Effects of Short-Term Training With Uncoupled Cranks in Trained Cyclists. *Int J Sport Physiol Perform*. 2012;7(2):113-120. https://search.ebscohost.com/login.aspx?direct=true&AuthType=cookie,ip,shib,uid&db=cul&AN=104461146&site=ehost-live&scope=site

315. Burns J, Boogaard H, Polus S, et al. Interventions to reduce ambient particulate matter air pollution and their effect on health. *Cochrane database Syst Rev*. 2019;5(5):CD010919. doi:10.1002/14651858.CD010919.pub2

316. Burns J, Mason C, Mueller N, et al. Asthma prevalence in Olympic summer athletes and the general population: An analysis of three European countries. *Respir Med*. 2015;109(7):813-820. doi:10.1016/j.rmed.2015.05.002

317. Burns J, Carter A, Draper S, Foad A. Engaging and sustaining people with intellectual disabilities in physical activity: a narrative review of existing evidence. *Int J Dev Disabil*. 2024;70(5):803-813. doi:10.1080/20473869.2022.2149096

318. Burns JC, Shike H, Gordon JB, et al. Sequelae of Kawasaki disease in adolescents and young adults. *J Am Coll Cardiol*. 1996;28(1):253-257. doi:10.1016/0735-1097(96)00099-X

319. Burns JM, Mayo MS, Anderson HS, Smith HJ, Donnelly JE. Cardiorespiratory fitness in early-stage Alzheimer disease. *Alzheimer Dis Assoc Disord*. 2008;22(1):39-46. doi:10.1097/WAD.0b013e31815a9ddc

320. Burns J, Dunning J. Is the preservation of the phrenic nerve important after pneumonectomy? *Interact Cardiovasc Thorac Surg*. 2011;12(1):47-50. doi:10.1510/icvts.2010.248583

321. Burns JW, Evon D. Common and specific process factors in cardiac rehabilitation: independent and interactive effects of the working alliance and self-efficacy. *Heal Psychol Off J Div Heal Psychol Am Psychol Assoc*. 2007;26(6):684-692. doi:10.1037/0278-6133.26.6.684

322. Burns JW, Nielson WR, Jensen MP, Heapy A, Czlapinski R, Kerns RD. Does Change Occur for the Reasons We Think It Does? A Test of Specific Therapeutic Operations During Cognitive-Behavioral Treatment of Chronic Pain. *Clin J Pain*. 2015;31(7):603-611. doi:10.1097/AJP.0000000000000141

323. Burns JK. Mental health and inequity: a human rights approach to inequality, discrimination, and mental disability. *Health Hum Rights*. 2009;11(2):19-31.

324. Burns J, Keenan AM, Redmond A. Foot type and overuse injury in triathletes. *J Am Podiatr Med Assoc*. 2005;95(3):235-241. doi:10.7547/0950235

325. Burns J, Keenan AM, Redmond AC. Factors associated with triathlon-related overuse injuries. *J Orthop Sports Phys Ther*. 2003;33(4):177-184. doi:10.2519/jospt.2003.33.4.177

326. Burns J, Raymond J, Ouvrier R. Feasibility of foot and ankle strength training in childhood Charcot-Marie-Tooth disease. *Neuromuscul Disord*. 2009;19(12):818-821. doi:10.1016/j.nmd.2009.09.007

327. Burns J, Ryan MM, Ouvrier RA. Evolution of foot and ankle manifestations in children with CMT1A. *Muscle Nerve*. 2009;39(2):158-166. doi:10.1002/mus.21140

328. Burns J, Sman AD, Cornett KMD, et al. Safety and efficacy of progressive resistance exercise for Charcot-Marie-Tooth disease in children: a randomised, double-blind, sham-controlled trial. *Lancet Child Adolesc Heal*. 2017;1(2):106-113. doi:10.1016/S2352-4642(17)30013-5

329. Burns KE, Dwyer JJM, Coe JB, Tam GCY, Wong SNR. Qualitative Pilot Study of Veterinarians’ Perceptions of and Experiences with Counseling about Dog Walking in Companion-Animal Practice in Southern Ontario. *J Vet Med Educ*. 2018;45(4):502-513. doi:10.3138/jvme.0117-011r1

330. Burns KE, Ferguson KA, Spouge A, Brown JE. Massive congenital coronary arteriovenous malformation presenting with exertional dyspnea and desaturation in an adult: a case report and review of the literature. *Can J Cardiol*. 2001;17(1):85-89.

331. Burns KJ. A new recommendation for physical activity as a means of health promotion. *Nurse Pract*. 1996;21(9):18,21-22,26-28.

332. Burns KJ. Beyond classical reliability: using generalizability theory to assess dependability. *Res Nurs Health*. 1998;21(1):83-90. doi:10.1002/(sici)1098-240x(199802)21:1<83::aid-nur9>3.0.co;2-p

333. Burns KJ, Camaione DN, Chatterton CT. Prescription of physical activity by adult nurse practitioners: a national survey. *Nurs Outlook*. 2000;48(1):28-33. doi:10.1067/mno.2000.99101

334. Burns KJ, Camaione DN, Froman RD, et al. Predictors of referral to cardiac rehabilitation and cardiac exercise self-efficacy. *Clin Nurs Res*. 1998;7(2):147-163. doi:10.1177/105477389800700205

335. Burns KJ, Froman RD. Refinement of the Habitual Physical Activity Index for use with American adults. *J Nurs Meas*. 1997;5(1):17-32.

336. Burns K, Heslin J, Crowley B, et al. Nosocomial outbreak of hepatitis B virus infection involving two hospitals in the Republic of Ireland. *J Hosp Infect*. 2011;78(4):279-283. doi:10.1016/j.jhin.2011.02.016

337. Burns KJ, Robinson K, Lowe EG. Evaluation of responses of an air medical helicopter program during a comprehensive emergency response drill. *Air Med J*. 2007;26(3):139-143. doi:10.1016/j.amj.2006.08.009

338. Burns KE, Chaurasia A, Carson V, Leatherdale ST. Examining If Changes in the Type of School-Based Intramural Programs Affect Youth Physical Activity over Time: A Natural Experiment Evaluation. *Int J Environ Res Public Health*. 2021;18(5). doi:10.3390/ijerph18052752

339. Burns KE, Chaurasia A, Carson V, Leatherdale ST. Examining if changes in gender-specific and co-ed intramural programs affect youth physical activity over time: a natural experiment evaluation using school- and student-level data from the COMPASS study. *BMC Public Health*. 2021;21(1):1-11. doi:10.1186/s12889-021-12090-z

340. Burns KE, Vermeer J, Battista K, Leatherdale ST. A School-Level Examination of the Association between Programs and Policies and Physical Activity Outcomes among Females from the COMPASS Study. *Int J Environ Res Public Health*. 2021;18(6). doi:10.3390/ijerph18063314

341. Burns K, Draghici AE, Taylor JA. Responses to Valsalva’s maneuver in spinal cord injury do not broadly relate to vasoconstrictor capacity. *Clin Auton Res Off J Clin Auton Res Soc*. 2024;34(6):571-581. doi:10.1007/s10286-024-01060-1

342. Burns K. Obesity. *J Am Vet Med Assoc*. 2008;232(3):341-342. doi:10.2460/javma.232.3.340

343. Burns KJ, Martin JC, Elmer SJ, McDaniel J. Response to Letter to the Editor: a counterweight is not necessary to implement simple, natural and comfortable single-leg cycle training. *Eur J Appl Physiol*. 2014;114(11):2457-2458. doi:10.1007/s00421-014-2965-z

344. Burns KJ, Pollock BS, Lascola P, McDaniel J. Cardiovascular responses to counterweighted single-leg cycling: implications for rehabilitation. *Eur J Appl Physiol*. 2014;114(5):961-968. doi:10.1007/s00421-014-2830-0

345. Burns KJ, Pollock BS, McDaniel J. The cardiovascular response to passive movement is joint dependent. *Physiol Rep*. 2016;4(5). doi:10.14814/phy2.12721

346. Burns KJ, Pollock BS, Stavres J, Kilbane M, Brochetti A, McDaniel J. Passive limb movement intervals results in repeated hyperemic responses in those with paraplegia. *Spinal Cord*. 2018;56(10):940-948. doi:10.1038/s41393-018-0099-6

347. Burns K, Bechara A. Decision making and free will: a neuroscience perspective. *Behav Sci Law*. 2007;25(2):263-280. doi:10.1002/bsl.751

348. Burns KM, Rey M, Baker CAH, Schriemer DC. Platform dependencies in bottom-up hydrogen/deuterium exchange mass spectrometry. *Mol Cell Proteomics*. 2013;12(2):539-548. doi:10.1074/mcp.M112.023770

349. Burns KM, Sarpe V, Wagenbach M, Wordeman L, Schriemer DC. HX-MS2 for high performance conformational analysis of complex protein states. *Protein Sci*. 2015;24(8):1313-1324. doi:10.1002/pro.2707

350. Burns LR, Bazzoli GJ, Dynan L, Wholey DR. Managed care, market stages, and integrated delivery systems: is there a relationship? *Health Aff*. 1997;16(6):204-218. doi:10.1377/hlthaff.16.6.204

351. Burns LR, Cacciamani J, Clement J, Aquino W. The fall of the house of AHERF: the Allegheny bankruptcy. *Health Aff (Millwood)*. 2000;19(1):7-41. doi:10.1377/hlthaff.19.1.7

352. Burns LR, Pauly M V. Big Med’s Spread. *Milbank Q*. 2023;101(2):287-324. doi:10.1111/1468-0009.12613

353. Burns M. Outpatient pulmonary rehabilitation. A new lease on life. *Postgrad Med*. 1989;86(6):129-130,135-137. doi:10.1080/00325481.1989.11704479

354. Burns M, Robben P, Venkataraman R. Lyme Carditis With Complete Heart Block Successfully Treated With Oral Doxycycline. *Mil Med*. Published online 2021. doi:10.1093/milmed/usab420

355. Burns M. An overview of field sobriety test research. *Percept Mot Skills*. 2003;97(3 Pt 2):1187-1199. doi:10.2466/pms.2003.97.3f.1187

356. Burns MPA, Reges CR, Barnhill SW, et al. Chronic cold exposure causes left ventricular hypertrophy that appears to be physiological. *J Exp Biol*. 2024;227(20). doi:10.1242/jeb.247476

357. Burns N. Lymphedema awareness. *Dermatology Nurs*. 1999;11(5):388.

358. Burns N, Finucane FM, Hatunic M, et al. Early-onset type 2 diabetes in obese white subjects is characterised by a marked defect in beta cell insulin secretion, severe insulin resistance and a lack of response to aerobic exercise training. *Diabetologia*. 2007;50(7):1500-1508. doi:10.1007/s00125-007-0655-7

359. Burns N, Shriki JE, Farvid AM, et al. Calcific constrictive pericarditis demonstrated on 99mTc-MDP bone scintigraphy. *J Radiol Case Rep*. 2009;3(5):11-15. doi:10.3941/jrcr.v3i5.63

360. Burns PA, Marecki MA, Dittmar SS, Bullough B. Kegel’s exercises with biofeedback therapy for treatment of stress incontinence. *Nurse Pract*. 1985;10(2):28,33-34,46.

361. Burns PA, Nochajski TH, Pranikoff K. Factors discriminating between genuine stress and mixed incontinence. *J Am Acad Nurse Pract*. 1992;4(1):15-21. doi:10.1111/j.1745-7599.1992.tb01106.x

362. Burns PA, Pranikoff K, Nochajski TH, Hadley EC, Levy KJ, Ory MG. A comparison of effectiveness of biofeedback and pelvic muscle exercise treatment of stress incontinence in older community-dwelling women. *J Gerontol*. 1993;48(4):M167-74. doi:10.1093/geronj/48.4.m167

363. Burns PA, Pranikoff K, Nochajski T, Desotelle P, Harwood MK. Treatment of stress incontinence with pelvic floor exercises and biofeedback. *J Am Geriatr Soc*. 1990;38(3):341-344. doi:10.1111/j.1532-5415.1990.tb03517.x

364. Burns P, Gough S, AW B. Management of peripheral arterial disease in primary care. *BMJ Br Med J (International Ed*. 2003;326(7389):584-588. doi:10.1136/bmj.326.7389.584

365. Burns P, Lima E, Bradbury AW. What constitutes best medical therapy for peripheral arterial disease? *Eur J Vasc Endovasc Surg Off J Eur Soc Vasc Surg*. 2002;24(1):6-12. doi:10.1053/ejvs.2002.1684

366. Burns P, Lima E, Bradbury AW. Second best medical therapy. *Eur J Vasc Endovasc Surg Off J Eur Soc Vasc Surg*. 2002;24(5):400-404. doi:10.1053/ejvs.2002.1737

367. Burns P, Wilmink T, Fegan C, Bradbury AW. Exercise in claudicants is accompanied by excessive thrombin generation. *Eur J Vasc Endovasc Surg Off J Eur Soc Vasc Surg*. 2003;26(2):150-155. doi:10.1053/ejvs.2002.1918

368. Burns P, Kressler J, Nash MS. Physiological Responses to Exergaming After Spinal Cord Injury. *Top Spinal Cord Inj Rehabil*. 2012;18(4):331-339. doi:10.1310/sci1804-331

369. Burns RA, Dunkman JAJ. Ordinal position learning and remote anticipation. *J Gen Psychol*. 2000;127(2):229-238. doi:10.1080/00221300009598581

370. Burns RA, French D, Luszcz M, Kendig HL, Anstey KJ. Heterogeneity in the Health and Functional Capacity of Adults Aged 85+as Risk for Mortality. *J Am Geriatr Soc*. 2019;67(5):1036-1042. doi:10.1111/jgs.15780

371. Burns RD, Armstrong JA. Associations of connectedness and parental behaviors with adolescent physical activity and mental health during COVID-19: A mediation analysis using the 2021 adolescent behaviors and experiences survey. *Prev Med (Baltim)*. 2022;164. doi:10.1016/j.ypmed.2022.107299

372. Burns RD, Bai Y, Fu Y, Brusseau TA. Associations of adolescent lifestyle behaviors with body mass index within a nationally representative sample of US adolescents: a quantile regression analysis. *Public Health*. 2020;179:51-58. doi:10.1016/j.puhe.2019.10.002

373. Burns RD, Bilic A, Bai Y, Brusseau TA, Lucero JE, Jensen JK. Bidirectional associations of physical activity, sleep, and self-reported mental health in young adults participating in an online wellness intervention during the COVID-19 pandemic. *Front PUBLIC Heal*. 2023;11. doi:10.3389/fpubh.2023.1168702

374. Burns RD, Brusseau TA, Fu Y, Bai Y, Byun W. Segmented School Physical Activity And Weight Status In Children: Application Of Compositional Data Analysis. *Med Sci Sport Exerc*. 2021;53(World Congress on Exercise is Medicine):184.

375. Burns RD, Brusseau TA, Fu Y, Zhang P. Development of Step-Count Cut Points for School-Day Vigorous Physical Activity. *Biomed Res Int*. 2018;2018. doi:10.1155/2018/9717848

376. Burns RD, Byun W, Bai Y, Silveira JFD, Reuter CP. Dose-response associations of Monitor-Independent Movement Summary with health-related fitness in youth. *Scand J Med Sci Sports*. 2023;33(11):2286-2298. doi:10.1111/sms.14448

377. Burns RD, Sehn AP, Brand C, Silveira JFD, Reuter CP. Moderating Influence of Home Location and School Type across Time on Cardiometabolic Risk and Active School Commuting: A Five-Year Longitudinal Study. *Child Obes*. 2023;19(4):258-266. doi:10.1089/chi.2021.0299

378. Burns RJ. Effect of exercise on labeled blood pool activity. *Circulation*. 1983;67(2):478.

379. Burns RJ, Bar-Shlomo BZ, Druck MN, et al. Detection of radiation cardiomyopathy by gated radionuclide angiography. *Am J Med*. 1983;74(2):297-302. doi:10.1016/0002-9343(83)90631-9

380. Burns RJ, Galligan L, Wright LM, Lawand S, Burke RJ, Gladstone PJ. Improved specificity of myocardial thallium-201 single-photon emission computed tomography in patients with left bundle branch block by dipyridamole. *Am J Cardiol*. 1991;68(5):504-508. doi:10.1016/0002-9149(91)90786-k

381. Burns RJ, Iles S, Fung AY, Wright LM, Daigneault L. The Canadian exercise technetium 99m-labeled teboroxime single-photon emission computed tomographic study. Canadian Exercise Teboroxime SPECT Study Investigators. *J Nucl Cardiol Off Publ Am Soc Nucl Cardiol*. 1995;2(2 Pt 1):117-125. doi:10.1016/s1071-3581(95)80022-0

382. Burns RJ, Kruzyk GC, Armitage DL, Druck MN. Effect of antianginal medications on the prognostic value of exercise thallium scintigraphy. *Can J Cardiol*. 1989;5(1):29-32.

383. Burns RJ, Liu PP, Druck MN, Seawright SJ, Williams WG, McLaughlin PR. Analysis of adults with and without complex ventricular arrhythmias after repair of tetralogy of Fallot. *J Am Coll Cardiol*. 1984;4(2):226-233. doi:10.1016/s0735-1097(84)80206-5

384. Burns RJ, Rothman AJ. Comparing Types of Financial Incentives to Promote Walking: An Experimental Test. *Appl Psychol WELL BEING*. 2018;10(2):193-214. doi:10.1111/aphw.12126

385. Burns R, Welker P. Interstitiality in the smart city: More than top-down and bottom-up smartness. *URBAN Stud*. 2023;60(2):308-324. doi:10.1177/00420980221097590

386. Burns RJ, Deschênes SS, Schmitz N. Associations Between Depressive Symptoms and Indices of Obesity in Adults With Prediabetes and Normal Blood Glucose Levels: Results From the Emotional Health and Wellbeing Study. *Can J diabetes*. 2018;42(6):626-631. doi:10.1016/j.jcjd.2018.05.005

387. Burns RJ, Fillo J, Deschênes SS, Schmitz N. Dyadic associations between physical activity and body mass index in couples in which one partner has diabetes: results from the Lifelines cohort study. *J Behav Med*. 2020;43(1):143-149. doi:10.1007/s10865-019-00055-y

388. Burns R, Olson I, Kazmucha J, Balise R, Chin R, Chin C. Correlation of subjective questionnaires with cardiac function as determined by exercise testing in a pediatric population. *Pediatr Cardiol*. 2010;31(7):1043-1048. doi:10.1007/s00246-010-9761-2

389. Burns RW. Exercise guidelines for adults: past, present & future. *Mo Med*. 2010;107(1):65-68.

390. Burns RB, Jay MR, Thorndike AN, Kanjee Z. How Would You Manage This Patient With Obesity? Grand Rounds Discussion From Beth Israel Deaconess Medical Center. *Ann Intern Med*. 2024;177(10):1415-1424. doi:10.7326/ANNALS-24-01740

391. Burns RD. Public Health Implications of the Dose-Response Association Between Physical Activity and Cardiometabolic Health in Young Adults. *J Adolesc Heal Off Publ Soc Adolesc Med*. 2020;67(2):155-156. doi:10.1016/j.jadohealth.2020.05.008

392. Burns RD. Public health implications of replacing screen time with physical activity and sleep in Brazilian children. *J Pediatr (Rio J)*. 2024;100(2):121-123. doi:10.1016/j.jped.2023.11.004

393. Burns RD. Enjoyment, self-efficacy, and physical activity within parent-adolescent dyads: Application of the actor-partner interdependence model. *Prev Med (Baltim)*. 2019;126:N.PAG-N.PAG. doi:10.1016/j.ypmed.2019.105756

394. Burns RD. Energy balance-related factors associating with adolescent weight loss intent: evidence from the 2017 National Youth Risk Behavior Survey. *BMC Public Health*. 2019;19(1):N.PAG-N.PAG. doi:10.1186/s12889-019-7565-8

395. Burns RD, Bai Y, Brusseau TA. Physical Activity and Sports Participation Associates With Cognitive Functioning and Academic Progression: An Analysis Using the Combined 2017-2018 National Survey of Children’s Health. *J Phys Act Health*. 2020;17(12):1197-1204. doi:10.1123/jpah.2020-0148

396. Burns RD, Bai Y, Byun W, et al. Bidirectional relationships of physical activity and gross motor skills before and after summer break: Application of a cross-lagged panel model. *J Sport Heal Sci*. 2022;11(2):244-251. doi:10.1016/j.jshs.2020.07.001

397. Burns RD, Bai Y, Fu Y, Pfledderer CD, Brusseau TA. Parent Engagement and Support, Physical Activity, and Academic Performance (PESPAAP): A Proposed Theoretical Model. *Int J Environ Res Public Health*. 2019;16(23). doi:10.3390/ijerph16234698

398. Burns RD, Bai Y, Pfledderer CD, Brusseau TA, Byun W. Movement Behaviors and Perceived Loneliness and Sadness within Alaskan Adolescents. *Int J Environ Res Public Health*. 2020;17(18). doi:10.3390/ijerph17186866

399. Burns RD, Brusseau TA, Fang Y, Fu Y, Hannon JC. Waist-to-Height Ratio, Aerobic Fitness, and Cardiometabolic Risk in Hispanic Children From Low-Income U.S. Schools. *Pediatr Exerc Sci*. 2016;28(3):388-396. doi:10.1123/pes.2016-0016

400. Burns RD, Brusseau TA, Fang Y, Myrer RS, Fu Y, Hannon JC. Predictors and grade level trends of school day physical activity achievement in low-income children from the U.S. *Prev Med reports*. 2015;2:868-873. doi:10.1016/j.pmedr.2015.10.002

401. Burns RD, Brusseau TA, Fu Y. Moderators of School-Based Physical Activity Interventions on Cardiorespiratory Endurance in Primary School-Aged Children: A Meta-Regression. *Int J Environ Res Public Health*. 2018;15(8). doi:10.3390/ijerph15081764

402. Burns RD, Brusseau TA, Fu Y, Hannon JC. Establishing school day pedometer step count cut-points using ROC curves in low-income children. *Prev Med (Baltim)*. 2016;86:117-122. doi:10.1016/j.ypmed.2016.02.018

403. Burns RD, Brusseau TA, Fu Y, Myrer RS, Hannon JC. Comprehensive School Physical Activity Programming and Classroom Behavior. *Am J Health Behav*. 2016;40(1):100-107. doi:10.5993/AJHB.40.1.11

404. Burns RD, Brusseau TA, Hannon JC. Physical Activity Trajectories During Daily Middle School Physical Education. *J Phys Act Health*. 2015;12(7):982-989. doi:10.1123/jpah.2014-0151

405. Burns RD, Brusseau TA, Hannon JC. Effect of a Comprehensive School Physical Activity Program on School Day Step Counts in Children. *J Phys Act Health*. 2015;12(12):1536-1542. doi:10.1123/jpah.2014-0578

406. Burns RD, Brusseau TA, Hannon JC. Effect of Comprehensive School Physical Activity Programming on Cardiometabolic Health Markers in Children From Low-Income Schools. *J Phys Act Health*. 2017;14(9):671-676. doi:10.1123/jpah.2016-0691

407. Burns RD, Fu Y, Fang Y, Hannon JC, Brusseau TA. Effect of a 12-Week Physical Activity Program on Gross Motor Skills in Children. *Percept Mot Skills*. 2017;124(6):1121-1133. doi:10.1177/0031512517720566

408. Burns RD, Fu Y, Hannon JC, Brusseau TA. School Physical Activity Programming and Gross Motor Skills in Children. *Am J Health Behav*. 2017;41(5):591-598. doi:10.5993/AJHB.41.5.8

409. Burns RD, Fu Y, Podlog LW. School-based physical activity interventions and physical activity enjoyment: A meta-analysis. *Prev Med (Baltim)*. 2017;103:84-90. doi:10.1016/j.ypmed.2017.08.011

410. Burns RD, Fu Y, Zhang P. Resistance Training and Insulin Sensitivity in Youth: A Meta-analysis. *Am J Health Behav*. 2019;43(2):228-242. doi:10.5993/AJHB.43.2.1

411. Burns RD, Hannon JC, Brusseau TA, et al. Cross-Validation of Aerobic Capacity Prediction Models in Adolescents. *Pediatr Exerc Sci*. 2015;27(3):404-411. doi:10.1123/pes.2014-0175

412. Burns RD, Hannon JC, Brusseau TA, et al. Development of an aerobic capacity prediction model from one-mile run/walk performance in adolescents aged 13–16 years. *J Sports Sci*. 2016;34(1):18-26. doi:10.1080/02640414.2015.1031163

413. Burns RD, Kim Y, Fu Y, Byun W, Bai Y. Independent and joint associations of aerobic and muscle-strengthening exercise with mental health in adolescents: A cross-sectional analysis before and during COVID-19 using the 2015–2021 National Youth Risk Behavior Survey. *Prev Med (Baltim)*. 2023;177:N.PAG-N.PAG. doi:10.1016/j.ypmed.2023.107750

414. Burns RD, Pfledderer CD, Fu Y. The neighbourhood social environment correlates with meeting 24-h movement behaviour recommendations in females: a cross-sectional study using the 2019 National Survey of Children’s Health. *Appl Physiol Nutr Metab*. 2021;46(4):408-411. doi:10.1139/apnm-2021-0045

415. Burns RD, Podlog LW, Bai Y. Enjoyment Mediates Associations of the Physical and Family Environment With Adolescent Physical Activity: A Structural Equation Modeling Approach. *J Adolesc Heal Off Publ Soc Adolesc Med*. 2022;71(5):628-634. doi:10.1016/j.jadohealth.2022.06.002

416. Burns RD, Fu Y. Testing the Motor Competence and Health-Related Variable Conceptual Model: A Path Analysis. *J Funct Morphol Kinesiol*. 2018;3(4). doi:10.3390/jfmk3040061

417. Burns R, Hannon JC, Brusseau TA, Shultz B, Eisenman P. Indices of abdominal adiposity and cardiorespiratory fitness test performance in middle-school students. *J Obes*. 2013;2013:912460. doi:10.1155/2013/912460

418. Burns R, Kim Y, Byun W, Brusseau T. Associations of School Day Sedentary Behavior and Physical Activity With Gross Motor Skills: Use of Compositional Data Analysis. *J Phys Act Health*. 2019;16(10):811-817. doi:10.1123/jpah.2018-0549

419. Burns SM, Sharples LD, Tait S, Caine N, Wallwork J, Schofield PM. The transmyocardial laser revascularization international registry report. *Eur Heart J*. 1999;20(1):31-37. doi:10.1053/euhj.1998.1202

420. Burns SP, Golding DG, Rolle WAJ, Graziani V, Ditunno JFJ. Recovery of ambulation in motor-incomplete tetraplegia. *Arch Phys Med Rehabil*. 1997;78(11):1169-1172. doi:10.1016/s0003-9993(97)90326-9

421. Burns SC, Kogan CS, Heyman RE, et al. Evaluating the Relationship Between Intimate Partner Violence-Related Training and Mental Health Professionals’ Assessment of Relationship Problems. *J Interpers Violence*. 2022;37(15/16):NP14262-NP14288. doi:10.1177/08862605211005154

422. Burns SA, Cleland JA, Cook CE, Bade M, Rivett DA, Snodgrass S. Variables Describing Individuals With Improved Pain and Function With a Primary Complaint of Low Back Pain: A Secondary Analysis. *J Manipulative Physiol Ther*. 2018;41(6):467-474. doi:10.1016/j.jmpt.2017.11.006

423. Burns SA, Cleland JA, Rivett DA, et al. When Treating Coexisting Low Back Pain and Hip Impairments, Focus on the Back: Adding Specific Hip Treatment Does Not Yield Additional Benefits-A Randomized Controlled Trial. *J Orthop Sports Phys Ther*. 2021;51(12):581-601. doi:10.2519/jospt.2021.10593

424. Burns SA, Cleland JA, Rivett DA, Snodgrass SJ. Examination procedures and interventions for the hip in the management of low back pain: a survey of physical therapists. *Brazilian J Phys Ther*. 2019;23(5):419-427. doi:10.1016/j.bjpt.2018.09.007

425. Burns SA, Cleland JA, Rivett DA, Snodgrass SJ. Effectiveness of physical therapy interventions for low back pain targeting the low back only or low back plus hips: a randomized controlled trial protocol. *Brazilian J Phys Ther*. 2018;22(5):424-430. doi:10.1016/j.bjpt.2018.08.014

426. Burns SA, Mintken PE, Austin GP. Clinical decision making in a patient with secondary hip-spine syndrome. *Physiother Theory Pract*. 2011;27(5):384-397. doi:10.3109/09593985.2010.509382

427. Burns SA, Mintken PE, Austin GP, Cleland J. Short-term response of hip mobilizations and exercise in individuals with chronic low back pain: a case series. *J Man Manip Ther*. 2011;19(2):100-107. doi:10.1179/2042618610Y.0000000007

428. Burns SM, Brown S, White CA, et al. Quantitative analysis of myocardial perfusion changes with transmyocardial laser revascularization. *Am J Cardiol*. 2001;87(7):861-867. doi:10.1016/S0002-9149(00)01527-7

429. Burns SH, Murray AD. Creating health through physical activity. *Br J Sports Med*. 2014;48(3):167-169. doi:10.1136/bjsports-2012-091769

430. Burns SB, Burns EA. Physical Exercise: The French Military in World War I. *J Altern Complement Med*. 2001;7(6):615-616. doi:10.1089/10755530152755153

431. Burns SF. Reductions in postprandial lipemia with exercise: is timing important? *Med Sci Sports Exerc*. 2008;40(7):1353; author reply 1354. doi:10.1249/MSS.0b013e3181775aa0

432. Burns SF. Re.: “Aerobic exercise alters postprandial lipemia in African American versus White women”. *Int J Sport Nutr Exerc Metab*. 2008;18(5):441-442; author reply 442. doi:10.1123/ijsnem.18.5.441

433. Burns SF. Metabolic Resistance With Inactivity Relates to Low Exercise Energy Expenditure. *Exerc Sport Sci Rev*. 2022;50(4):230. doi:10.1249/JES.0000000000000298

434. Burns SF, Broom DR, Miyashita M, Mundy C, Stensel DJ. A single session of treadmill running has no effect on plasma total ghrelin concentrations. *J Sports Sci*. 2007;25(6):635-642. doi:10.1080/02640410600831856

435. Burns SF, Broom DR, Miyashita M, Ueda C, Stensel DJ. Increased postprandial triacylglycerol concentrations following resistance exercise. *Med Sci Sports Exerc*. 2006;38(3):527-533. doi:10.1249/01.mss.0000187414.72289.89

436. Burns SF, Corrie H, Holder E, Nightingale T, Stensel DJ. A single session of resistance exercise does not reduce postprandial lipaemia. *J Sports Sci*. 2005;23(3):251-260. doi:10.1080/02640410410001730142

437. Burns SF, Hardman AE, Stensel DJ. Brisk walking offsets the increase in postprandial TAG concentrations found when changing to a diet with increased carbohydrate. *Br J Nutr*. 2009;101(12):1787-1796. doi:10.1017/S0007114508133591

438. Burns SF, Miyashita M, Ueda C, Stensel DJ. Multiple bouts of resistance exercise and postprandial triacylglycerol and serum C-reactive-protein concentrations. *Int J Sport Nutr Exerc Metab*. 2007;17(6):556-573. doi:10.1123/ijsnem.17.6.556

439. Burns SF, Oo HH, Tran ATT. Effect of Sprint Interval Exercise on Postexercise Metabolism and Blood Pressure in Adolescents. *Int J Sport Nutr Exerc Metab*. 2012;22(1):47-54. doi:10.1123/ijsnem.22.1.47

440. Burns SF, Stensel DJ. Effects of low- and high-volume resistance exercise on postprandial lipaemia: comments by Burns and Stensel. *Br J Nutr*. 2008;99(1):211; discussion 212-3. doi:10.1017/S0007114507761755

441. Burns SP, Rivara FP, Johansen JM, et al. Rehabilitation of traumatic injuries: use of the Delphi method to identify topics for evidence-based review. *Am J Phys Med Rehabil*. 2003;82(5):410-414. doi:10.1097/01.phm.0000064739.60860.a7

442. Burns S, Miyashita M, Stensel D. High-Intensity Interval Exercise and Postprandial Triacylglycerol. *Sport Med*. 2015;45(7):957-968. doi:10.1007/s40279-015-0327-6

443. Burns SP, Terblanche M, Perea J, et al. mHealth Intervention Applications for Adults Living With the Effects of Stroke: A Scoping Review. *Arch Rehabil Res Clin Transl*. 2021;3(1):100095. doi:10.1016/j.arrct.2020.100095

444. Burns SL. Concussion Treatment Using Massage Techniques: a Case Study. *Int J Ther Massage Bodywork*. 2015;8(2):12-17.

445. Burns TE, Clayton HM. Comparison of the temporal kinematics of the canter pirouette and collected canter. *Equine Vet J Suppl*. 1997;(23):58-61. doi:10.1111/j.2042-3306.1997.tb05055.x

446. Burns TP, Turba JE. Arthroscopic treatment of shoulder impingement in athletes. *Am J Sports Med*. 1992;20(1):13-16. doi:10.1177/036354659202000104

447. Burns T, Knapp M, Catty J, et al. Home treatment for mental health problems: a systematic review. *Health Technol Assess*. 2001;5(15):1-139. doi:10.3310/hta5150

448. Burns T, Rugkåsa J, Yeeles K, Catty J. *No Title*.; 2016. doi:10.3310/pgfar04210

449. Burns V. Stress and antibody response to vaccination: implications of animal studies for human clinical research. *Expert Rev Vaccines*. 2004;3(2):141-149. doi:10.1586/14760584.3.2.141

450. Burns VE. Using vaccinations to assess in vivo immune function in psychoneuroimmunology. *Methods Mol Biol*. 2012;934:371-381. doi:10.1007/978-1-62703-071-7_19

451. Burns VE, Carroll D, Drayson M, Whitham M, Ring C. Life events, perceived stress and antibody response to influenza vaccination in young, healthy adults. *J Psychosom Res*. 2003;55(6):569-572. doi:10.1016/s0022-3999(03)00073-4

452. Burns VE, Carroll D, Ring C, Drayson M. Antibody response to vaccination and psychosocial stress in humans: relationships and mechanisms. *Vaccine*. 2003;21(19-20):2523-2534. doi:10.1016/s0264-410x(03)00041-0

453. Burns VE, Carroll D, Ring C, Harrison LK, Drayson M. Stress, coping, and hepatitis B antibody status. *Psychosom Med*. 2002;64(2):287-293. doi:10.1097/00006842-200203000-00012

454. Burns VE, Drayson M, Ring C, Carroll D. Perceived stress and psychological well-being are associated with antibody status after meningitis C conjugate vaccination. *Psychosom Med*. 2002;64(6):963-970. doi:10.1097/01.psy.0000038936.67401.28

455. Burns VE, Edwards KM, Ring C, Drayson M, Carroll D. Complement cascade activation after an acute psychological stress task. *Psychosom Med*. 2008;70(4):387-396. doi:10.1097/PSY.0b013e31816ded22

456. Burns VE, Gallagher S. Antibody response to vaccination as a marker of in vivo immune function in psychophysiological research. *Neurosci Biobehav Rev*. 2010;35(1):122-126. doi:10.1016/j.neubiorev.2010.01.005

457. Burns VE, Ring C, Carroll D. Factors influencing influenza vaccination uptake in an elderly, community-based sample. *Vaccine*. 2005;23(27):3604-3608. doi:10.1016/j.vaccine.2004.12.031

458. Burns VE, Ring C, Drayson M, Carroll D. Cortisol and cardiovascular reactions to mental stress and antibody status following hepatitis B vaccination: a preliminary study. *Psychophysiology*. 2002;39(3):361-368. doi:10.1017/s0048577201393022

459. Burns VE, Ring C, Harrison LK, Carroll D, Drayson M. Reductions in secretory immunoglobulin A to cold pressor stress are not influenced by timing of saliva sampling. *Biol Psychol*. 2004;66(1):91-98. doi:10.1016/j.biopsycho.2003.07.001

460. Burns WP, Nugent CD, McCullagh PJ, et al. Evaluation of a technology enabled garment for older walkers. *Annu Int Conf IEEE Eng Med Biol Soc IEEE Eng Med Biol Soc Annu Int Conf*. 2012;2012:2100-2103. doi:10.1109/EMBC.2012.6346374

461. Bushby K, Finkel R, Wong B, et al. Ataluren treatment of patients with nonsense mutation dystrophinopathy. *Muscle Nerve*. 2014;50(4):477-487. doi:10.1002/mus.24332

462. Butler RN, Davis R, Lewis CB, Nelson ME, Strauss E. Physical fitness: how to help older patients live stronger and longer. (1). *Geriatrics*. 1998;53(9):26-28,31-32,39-40.

463. Buttaci CJ. Erythromelalgia: a case report and literature review. *Pain Med*. 2006;7(6):534-538. doi:10.1111/j.1526-4637.2006.00240.x

464. Butz M, Conrady D, Baumgärtler H, Mentzel HE. [Rehabilitation of burn victims. A difficult path back to normality]. *MMW Fortschr Med*. 2002;144(24):32-34.

465. Bystrova T V, Gerasimova LI, Lapshin VP, Menchukov ON. [The central hemodynamic indices during graded physical loading in burn patients with respiratory tract involvement]. *Vestn Khir Im I I Grek*. 1991;146(4):66-70.

466. Calder PC, Yaqoob P. Glutamine and the immune system. *Amino Acids*. 1999;17(3):227-241. doi:10.1007/BF01366922

467. Camaione DN, Burns KJ, Chatterton CT. Counseling for physical activity: what primary-care physicians should know. *Conn Med*. 1997;61(7):391-395.

468. Cambiaso-Daniel J, Parry I, Rivas E, et al. Strength and Cardiorespiratory Exercise Rehabilitation for Severely Burned Patients During Intensive Care Units: A Survey of Practice. *J Burn CARE Res*. 2018;39(6):897-901. doi:10.1093/jbcr/iry002

469. Cambiaso-Daniel J, Rivas E, Carson JS, et al. Cardiorespiratory Capacity and Strength Remain Attenuated in Children with Severe Burn Injuries at Over 3 Years Postburn. *J Pediatr*. 2018;192:152-158. doi:10.1016/j.jpeds.2017.09.015

470. Campbell JP, Edwards KM, Ring C, et al. The effects of vaccine timing on the efficacy of an acute eccentric exercise intervention on the immune response to an influenza vaccine in young adults. *Brain Behav Immun*. 2010;24(2):236-242. doi:10.1016/j.bbi.2009.10.001

471. Campbell JP, Riddell NE, Burns VE, et al. Acute exercise mobilises CD8+ T lymphocytes exhibiting an effector-memory phenotype. *Brain Behav Immun*. 2009;23(6):767-775. doi:10.1016/j.bbi.2009.02.011

472. Campeau RJ, Spellman JG, Tenaglia AN. Spontaneous coronary artery spasm documented in a young woman. A case report. *Clin Nucl Med*. 1996;21(6):452-455. doi:10.1097/00003072-199606000-00003

473. Careau V, Halsey LG, Pontzer H, et al. Energy compensation and adiposity in humans. *Curr Biol*. 2021;31(20):4659-4666.e2. doi:10.1016/j.cub.2021.08.016

474. Carethers M. Health promotion in the elderly. *Am Fam Physician*. 1992;45(5):2253-2259.

475. Carey DG. Quantifying differences in the “fat burning” zone and the aerobic zone: implications for training. *J strength Cond Res*. 2009;23(7):2090-2095. doi:10.1519/JSC.0b013e3181bac5c5

476. Carlin A, Perchoux C, Puggina A, et al. A life course examination of the physical environmental determinants of physical activity behaviour: A “Determinants of Diet and Physical Activity” (DEDIPAC) umbrella systematic literature review. *PLoS One*. 2017;12(8):e0182083. doi:10.1371/journal.pone.0182083

477. Carman C, Chang B. Treadmill injuries to the upper extremity in pediatric patients. *Ann Plast Surg*. 2001;47(1):15-19. doi:10.1097/00000637-200107000-00003

478. Carpenter JS, Burns DS, Wu J, et al. Strategies used and data obtained during treatment fidelity monitoring. *Nurs Res*. 2013;62(1):59-65. doi:10.1097/NNR.0b013e31827614fd

479. Carraro F, Hartl WH, Stuart CA, Layman DK, Jahoor F, Wolfe RR. Whole body and plasma protein synthesis in exercise and recovery in human subjects. *Am J Physiol*. 1990;258(5 Pt 1):E821-31. doi:10.1152/ajpendo.1990.258.5.E821

480. Carraro F, Kimbrough TD, Wolfe RR. Urea kinetics in humans at two levels of exercise intensity. *J Appl Physiol*. 1993;75(3):1180-1185. doi:10.1152/jappl.1993.75.3.1180

481. Carraro F, Klein S, Rosenblatt JI, Wolfe RR. Effect of dichloroacetate on lactate concentration in exercising humans. *J Appl Physiol*. 1989;66(2):591-597. doi:10.1152/jappl.1989.66.2.591

482. Carraro F, Naldini A, Weber JM, Wolfe RR. Alanine kinetics in humans during low-intensity exercise. *Med Sci Sports Exerc*. 1994;26(3):348-353.

483. Carraro F, Stuart CA, Hartl WH, Rosenblatt J, Wolfe RR. Effect of exercise and recovery on muscle protein synthesis in human subjects. *Am J Physiol*. 1990;259(4 Pt 1):E470-6. doi:10.1152/ajpendo.1990.259.4.E470

484. Carr-Collins JA. Pressure techniques for the prevention of hypertrophic scar. *Clin Plast Surg*. 1992;19(3):733-743.

485. Carrougher GJ, Hoffman HG, Nakamura D, et al. The effect of virtual reality on pain and range of motion in adults with burn injuries. *J Burn care Res*. 2009;30(5 CC-Injuries CC-Pain, Palliative and Supportive Care):785‐791. doi:10.1097/BCR.0b013e3181b485d3

486. Carter EA, Paul K, Bonab AA, Tompkins RG, Fischman AJ. Effect of exercise on burn-induced changes in tissue-specific glucose metabolism. *J Burn Care Res*. 2014;35(6):470-473. doi:10.1097/BCR.0000000000000036

487. Cartotto R, Johnson L, Rood JM, et al. Clinical Practice Guideline: Early Mobilization and Rehabilitation of Critically Ill Burn Patients. *J Burn CARE Res*. 2023;44(1):1-15. doi:10.1093/jbcr/irac008

488. Casa B, Caleffi E, Bocchi A, Ferraro F, Del Piano P. [Rehabilitation of burned patients. II. Therapeutic exercise]. *Acta bio-medica L’Ateneo Parm organo della Soc di Med e Sci Nat di Parma*. 1990;61(1-2):67-71.

489. Casaburi R, Porszasz J, Burns MR, Carithers ER, Chang RS, Cooper CB. Physiologic benefits of exercise training in rehabilitation of patients with severe chronic obstructive pulmonary disease. *Am J Respir Crit Care Med*. 1997;155(5):1541-1551. doi:10.1164/ajrccm.155.5.9154855

490. Caserta A, Morgan P, McKay MJ, Baldwin JN, Burns J, Williams C. Children with idiopathic toe walking display differences in lower limb joint ranges and strength compared to peers: a case control study. *J Foot Ankle Res*. 2022;15(1):70. doi:10.1186/s13047-022-00576-x

491. Celis MM, Suman OE, Huang TT, Yen P, Herndon DN. Effect of a supervised exercise and physiotherapy program on surgical interventions in children with thermal injury. *J Burn Care Rehabil*. 2003;24(1 CC-HS-HANDSRCH CC-Child Health CC-Effective Practice and Organisation of Care CC-SR-REHAB CC-Wounds):57‐61; discussion 56. doi:10.1097/00004630-200301000-00014

492. Chadorneshin HT, Golestani A, Jamali F, Shirvan SMM, Sarir H, Eivary SHA. The response of intercellular adhesion molecule-1 to exhaustive submaximal exercise and its correlation with physiological and anthropometric measures. *J Med Life*. 2018;11(1):36-41.

493. CHAI J ke. [Mechanisms of skeletal muscle wasting after severe burn and its treatment]. *Zhonghua Shao Shang Za Zhi*. 2009;25(4):243-245.

494. Chai J ke, Song H feng, Chen M liang, et al. [The treatment of deformity of axillary scar contracture after burns]. *Zhonghua Yi Xue Za Zhi*. 2004;84(10):830-832.

495. Champ CE, Ohri N, Klement RJ, et al. Assessing Changes in the Activity Levels of Breast Cancer Patients During Radiation Therapy. *Clin Breast Cancer*. 2018;18(1):E1-E6. doi:10.1016/j.clbc.2017.08.009

496. Chan HH, Burns SF. Oxygen consumption, substrate oxidation, and blood pressure following sprint interval exercise. *Appl Physiol Nutr Metab*. 2013;38(2):182-187. doi:10.1139/apnm-2012-0136

497. Chan SY, Mancini GBJ, Burns S, et al. Dietary measures and exercise training contribute to improvement of endothelial function and atherosclerosis even in patients given intensive pharmacologic therapy. *J Cardiopulm Rehabil*. 2006;26(5):288-293. doi:10.1097/00008483-200609000-00002

498. Chan WY, Figus A, Ekwobi C, Srinivasan JR, Ramakrishnan V V. The “round-the-clock” training model for assessment and warm up of microsurgical skills: a validation study. *J Plast Reconstr Aesthet Surg*. 2010;63(8):1323-1328. doi:10.1016/j.bjps.2009.06.027

499. Chang CCA, Lin YC. Physical activity and food consumption: The moderating role of individual dieting tendency. *J Health Psychol*. 2015;20(5):490-499. doi:10.1177/1359105315573469

500. Chang SKY, Tominaga GT, Wong JH, Weldon EJ, Kaan KT. Risk factors for water sports-related cervical spine injuries. *J Trauma*. 2006;60(5):1041-1046. doi:10.1097/01.ta.0000218256.39295.8f

501. Chang YT, Liu CT, Hsu SW, Lee CC, Huang PC. Functional Connectivity, Physical Activity, and Neurocognitive Performances in Patients with Vascular Cognitive Impairment, No Dementia. *Curr Alzheimer Res*. 2022;19(1):56-67. doi:10.2174/1567205019666220127103852

502. Chao T, Parry I, Palackic A, et al. The effects of short bouts of ergometric exercise for severely burned children in intensive care: A randomized controlled trial. *Clin Rehabil*. 2022;36(8):1052-1061. doi:10.1177/02692155221095643

503. Chao T, Porter C, Herndon DN, et al. Propranolol and Oxandrolone Therapy Accelerated Muscle Recovery in Burned Children. *Med Sci Sports Exerc*. 2018;50(3):427‐435. doi:10.1249/MSS.0000000000001459

504. Chappel SE, Aisbett B, Vincent GE, Ridgers ND. Firefighters’ Physical Activity across Multiple Shifts of Planned Burn Work. *Int J Environ Res Public Health*. 2016;13(10). doi:10.3390/ijerph13100973

505. Charbonneau RME, McVeigh SA, Thompson K. Brachial neuropraxia in Canadian Atlantic University sport football players: what is the incidence of “stingers”? *Clin J Sport Med Off J Can Acad Sport Med*. 2012;22(6):472-477. doi:10.1097/JSM.0b013e3182699ed5

506. Chastin SFM, De Craemer M, Lien N, et al. The SOS-framework (Systems of Sedentary behaviours): an international transdisciplinary consensus framework for the study of determinants, research priorities and policy on sedentary behaviour across the life course: a DEDIPAC-study. *Int J Behav Nutr Phys Act*. 2016;13:83. doi:10.1186/s12966-016-0409-3

507. Chatzivasiloglou F, Katsenos S, Psara A, Tsintiris K. Orange-Pigmented Sputum as a Manifestation of Smoke Grenade Inhalation Injury. *J Bronchology Interv Pulmonol*. 2016;23(1):76-78. doi:10.1097/LBR.0000000000000191

508. Chee C, Shannon CE, Burns A, et al. Increasing skeletal muscle carnitine content in older individuals increases whole-body fat oxidation during moderate-intensity exercise. *Aging Cell*. 2021;20(2). doi:10.1111/acel.13303

509. Chee C, Shannon CE, Burns A, et al. Relative Contribution of Intramyocellular Lipid to Whole-Body Fat Oxidation Is Reduced With Age but Subsarcolemmal Lipid Accumulation and Insulin Resistance Are Only Associated With Overweight Individuals. *Diabetes*. 2016;65(4):840-850. doi:10.2337/db15-1383

510. Chen B, Lu S, Li S, Wang B. Impact of fine particulate fluctuation and other variables on Beijing’s air quality index. *Environ Sci Pollut Res Int*. 2015;22(7):5139-5151. doi:10.1007/s11356-014-4024-z

511. Chen F, Wu J, Zhang X, Chen Z, Yue X, Chen B. An Innovative Approach to Classifying and Treating Axillary Scar Contracture. *Ann Plast Surg*. 2024;93(1):48-58. doi:10.1097/SAP.0000000000004014

512. Chen H, Sun L, Feng L, et al. Intermittent fasting promotes type 3 innate lymphoid cells secreting IL-22 contributing to the beigeing of white adipose tissue. *Elife*. 2024;12. doi:10.7554/eLife.91060

513. Chen JY, Fu CW, Ho HY, Lu YC. Surgical treatment of postburn heterotopic ossification around the elbow: Three case reports. *Medicine (Baltimore)*. 2019;98(6):e14403. doi:10.1097/MD.0000000000014403

514. Chen P, Yu N, Tsai H, et al. Hospitalized burn injury risk associated with benzodiazepines and Z-drugs in elders: A population-based case-control study. *Int J Geriatr Psychiatry*. 2019;34(10):1465-1472. doi:10.1002/gps.5155

515. Chen Q, Huang S, Chen X, Feng L, Zhu X. Clinical efficacy of multi-pattern detumescence after total knee arthroplasty treated with acupoint massage and mild moxibustion. *Zhongguo zhen jiu [Chinese Acupunct moxibustion]*. 2016;36(5 CC-Complementary Medicine):471‐475. https://www.cochranelibrary.com/central/doi/10.1002/central/CN-01200097/full

516. Chen S, Du C, Shen M, et al. Sympathetic stimulation facilitates thrombopoiesis by promoting megakaryocyte adhesion, migration, and proplatelet formation. *Blood*. 2016;127(8):1024-1035. doi:10.1182/blood-2015-07-660746

517. Chen SG, Tzeng YS, Wang CH. Treatment of severe burn with DermACELL(®), an acellular dermal matrix. *Int J Burns Trauma*. 2012;2(2):105-109.

518. Chen W hao, Ye H feng, Wu Y xuan, et al. Association of creatinine-albumin ratio with 28-day mortality in major burned patients: A retrospective cohort study. *Burns*. 2023;49(7):1614-1620. doi:10.1016/j.burns.2023.04.002

519. Cheng AJ, Hawke TJ. Wasting away: AJP-Cell Physiology initiates thematic reviews on skeletal muscle wasting. *Am J Physiol Cell Physiol*. 2021;321(1):C38-C39. doi:10.1152/ajpcell.00165.2021

520. Cheng X, Dib-Hajj SD, Tyrrell L, Wright DA, Fischer TZ, Waxman SG. Mutations at opposite ends of the DIII/S4-S5 linker of sodium channel Na V 1.7 produce distinct pain disorders. *Mol Pain*. 2010;6:24. doi:10.1186/1744-8069-6-24

521. Chenoweth D. Sitting around, burning money. *Bus Health*. 2001;19(2):27-28.

522. Cheruvu VPR, Gaba S, John JR, Rawat S. Management of extra-articular shaft fractures of the non-thumb metacarpals: plate-screw fixation versus K-wire fixation. *Int J Burns Trauma*. 2021;11(5):365-376.

523. Chester JE, Rowneki M, Van Doren W, Helmer DA. Progression of intervention-focused research for Gulf War illness. *Mil Med Res*. 2019;6(1):31. doi:10.1186/s40779-019-0221-x

524. Chester M, Hammond C, Leach A, Chester M, Hammond C, Leach A. Long-term benefits of stellate ganglion block in severe chronic refractory angina. *Pain*. 2000;87(1):103-105. doi:10.1016/S0304-3959(00)00270-0

525. Chew EY, Burns SA, Abraham AG, et al. Standardization and clinical applications of retinal imaging biomarkers for cardiovascular disease: a Roadmap from an NHLBI workshop. *Nat Rev Cardiol*. Published online July 2024. doi:10.1038/s41569-024-01060-8

526. Chia JS, Burns SF, Barrett LA, Chow JY. Increased Complexities in Visual Search Behavior in Skilled Players for a Self-Paced Aiming Task. *Front Psychol*. 2017;8:987. doi:10.3389/fpsyg.2017.00987

527. Chia JS, Chow JY, Barrett LA, Burns SF. Reliability of a Novel Badminton Intermittent Exercise Protocol. *Res Q Exerc Sport*. 2019;90(4):487-496. doi:10.1080/02701367.2019.1620911

528. Chia J, Barrett L, Chow J, Burns S. Effects of Caffeine Supplementation on Performance in Ball Games. *Sport Med*. 2017;47(12):2453-2471. doi:10.1007/s40279-017-0763-6

529. Chiang BY, Olsen DB, Gaykowski R, et al. Evaluation of treadmill exercise on total artificial heart recipients. *Trans Am Soc Artif Intern Organs*. 1984;30:514-519.

530. Chiang BY, Pantalos GM, Burns GL, et al. Oxygen metabolism in animals with total artificial hearts. *ASAIO J*. 1994;40(3):M510-3. doi:10.1097/00002480-199407000-00052

531. Chiang BY, Pantalos GM, Burns GL, et al. Adaptive responses of total artificial heart animals to treadmill exercise. *ASAIO J*. 1993;39(3):M381-5.

532. Chiang BY, Pantalos G, Burns GL, et al. Anaerobic threshold in total artificial heart animals. *ASAIO J*. 1994;40(3):M335-8. doi:10.1097/00002480-199407000-00019

533. Chiu CH, Burns SF, Yang TJ, et al. Energy replacement using glucose does not increase postprandial lipemia after moderate intensity exercise. *Lipids Health Dis*. 2014;13:177. doi:10.1186/1476-511X-13-177

534. Chiwaridzo M, Zinyando VJ, Dambi JM, Kaseke F, Munambah N, Mudawarima T. Perspectives of caregivers towards physiotherapy treatment for children with burns in Harare, Zimbabwe: A cross-sectional study. *Burn trauma*. 2016;4:31. doi:10.1186/s41038-016-0057-5

535. Cho BC, Lee JH, Weinzweig N, Baik BS. Use of the free innervated dorsalis pedis tendocutaneous flap in composite hand reconstruction. *Ann Plast Surg*. 1998;40(3):268-276. doi:10.1097/00000637-199803000-00013

536. Choo HC, Choo DHW, Tan I, et al. Effect of ice slurry ingestion on thermoregulatory responses during fixed-intensity cycling in humid and dry heat. *Eur J Appl Physiol*. 2023;123(10):2225-2237. doi:10.1007/s00421-023-05235-y

537. Choong K, Foster G, Fraser DD, et al. Acute rehabilitation practices in critically ill children: a multicenter study. *Pediatr Crit care Med a J Soc Crit Care Med World Fed Pediatr Intensive Crit Care Soc*. 2014;15(6):e270-9. doi:10.1097/PCC.0000000000000160

538. Choron G, Dutheil F, Lesage FX. Are nurses burned out? *Int J Nurs Stud*. 2016;58:80-81. doi:10.1016/j.ijnurstu.2016.02.002

539. Chouinard NH, Beaudoin Cloutier C, Chang SL, et al. The economic burden of burned patients for hospitalization in Canada. *Burns*. 2024;50(6):1494-1503. doi:10.1016/j.burns.2024.03.036

540. Chown GA. A retrospective case series study of Deepavali fire walkers in Singapore. *Burns*. 2010;36(5):711-715. doi:10.1016/j.burns.2009.10.005

541. Christen AG, Cooper KH. Strategic withdrawal from cigarette smoking. *CA Cancer J Clin*. 1979;29(2):96-107. doi:10.3322/canjclin.29.2.96

542. Christian H, Lester L, Trost SG, et al. Shade coverage, ultraviolet radiation and children’s physical activity in early childhood education and care. *Int J Public Health*. 2019;64(9):1325-1333. doi:10.1007/s00038-019-01289-y

543. Chu BM, Brody G. Nondestructive measurements of the properties of healing burn scars. *Med Instrum*. 1975;9(3):139-142.

544. Chua MT, Sim A, Burns SF. Acute and Chronic Effects of Blood Flow Restricted High-Intensity Interval Training: A Systematic Review. *Sport Med - Open*. 2022;8(1):1-26. doi:10.1186/s40798-022-00506-y

545. Chuang S, Chang KS, Woods DD, Chen HC, Reynolds ME, Chien DK. Beyond surge: Coping with mass burn casualty in the closest hospital to the Formosa Fun Coast Dust Explosion. *Burn*. 2019;45(4):964-973. doi:10.1016/j.burns.2018.12.003

546. Chubirko KI, Horlenko OM, Bentsa TM, et al. Syndromal characteristics of the combined course of chronic pancreatitis and arterial hypertension. *Wiad Lek*. 2020;73(3):428-433.

547. Chung HG, Wick MR, Joo CE, Harriger JA. Physical attributes of workout instructors and appearance-related messaging in a sample of home workout videos on YouTube: A content analysis. *J Health Psychol*. Published online April 2024:13591053241242534. doi:10.1177/13591053241242534

548. Çinar MA, Bayramlar K, Erkiliç A, et al. The effects of early physiotherapy on biochemical parameters in major burn patients: A burn center’s experience. *Ulus TRAVMA VE ACIL CERRAHI DERGISI-TURKISH J TRAUMA Emerg Surg*. 2019;25(5):461-466. doi:10.5505/tjtes.2018.05950

549. Çınar MA, Bayramlar K, Erkılıc A, Güneş A, Yakut Y. Effect of three different exercise trainings on functional capacity in early stage severe burn patients: A randomized controlled trial. *Ulus travma ve acil cerrahi Derg = Turkish J trauma Emerg Surg TJTES*. 2024;30(8):270-562. doi:10.14744/tjtes.2024.59987

550. Çinar MA, Erkiliç A. Effect of aerobic exercise on neutrophil-lymphocyte ratio, platelet-lymphocyte ratio, and lymphocyte-monocyte ratio in burn patients: A randomized controlled trial. *J Plast Reconstr Aesthet Surg*. 2024;95:199-206. doi:10.1016/j.bjps.2024.05.032

551. Ciniglio R, Kime M, Burns TL, Vandenberg BF. Rapid resolution of hyperkinesis after exercise. Two-dimensional echocardiographic studies in normal subjects. *Chest*. 1993;104(3):712-717. https://search.ebscohost.com/login.aspx?direct=true&AuthType=cookie,ip,shib,uid&db=cul&AN=136618917&site=ehost-live&scope=site

552. CL J, VJ C, Johnson CL, Cain VJ. CE burn care: the rehab guide. *AJN Am J Nurs*. 1985;85(1):48-50. https://search.ebscohost.com/login.aspx?direct=true&AuthType=cookie,ip,shib,uid&db=cul&AN=107580857&site=ehost-live&scope=site

553. Clark DJ, Chatterjee SA, Skinner JW, et al. Combining Frontal Transcranial Direct Current Stimulation With Walking Rehabilitation to Enhance Mobility and Executive Function: A Pilot Clinical Trial. *Neuromodulation*. 2021;24(5):950-959. doi:10.1111/ner.13250

554. Clark JE, Welch S. Comparing effectiveness of fat burners and thermogenic supplements to diet and exercise for weight loss and cardiometabolic health: Systematic review and meta-analysis. *Nutr Health*. 2021;27(4):445-459. doi:10.1177/0260106020982362

555. Clarke A, Simmons J, White P, et al. Attitudes to face transplantation: results of a public engagement exercise at the Royal Society Summer Science Exhibition. *J Burn Care Res*. 2006;27(3):394-398. doi:10.1097/01.bcr.0000217578.47909.7f

556. Clarke HM, Wittpenn GP, McLeod AM, et al. Acute management of pediatric hand burns. *Hand Clin*. 1990;6(2):221-232.

557. Clausen T. Hormonal and pharmacological modification of plasma potassium homeostasis. *Fundam Clin Pharmacol*. 2010;24(5):595-605. doi:10.1111/j.1472-8206.2010.00859.x

558. Clausen T. Na+-K+ pump stimulation improves contractility in damaged muscle fibers. *Ann N Y Acad Sci*. 2005;1066:286-294. doi:10.1196/annals.1363.021

559. Clayton NA, Ward EC, Maitz PKM. Full thickness facial burns: Outcomes following orofacial rehabilitation. *Burn*. 2015;41(7):1599-1606. doi:10.1016/j.burns.2015.04.003

560. Clayton NA, Ward EC, Maitz PKM. Orofacial contracture management outcomes following partial thickness facial burns. *Burn*. 2015;41(6):1291-1297. doi:10.1016/j.burns.2015.02.015

561. Clayton NA, Ward EC, Nicholls C, Giannone R, Skylas K, Maitz PK. The addition of respiratory muscle strength training to facilitate swallow and pulmonary rehabilitation following massive tissue loss and severe deconditioning: A case series. *Aust Crit care Off J Confed Aust Crit Care Nurses*. 2022;35(2):210-216. doi:10.1016/j.aucc.2021.03.003

562. Clayton NA, Ward EC, Maitz PK. Intensive swallowing and orofacial contracture rehabilitation after severe burn: A pilot study and literature review. *Burn*. 2017;43(1):e7-e17. doi:10.1016/j.burns.2016.07.006

563. Clayton RP, Wurzer P, Andersen CR, Mlcak RP, Herndon DN, Suman OE. Effects of different duration exercise programs in children with severe burns. *Burns*. 2017;43(4):796‐803. doi:10.1016/j.burns.2016.11.004

564. Clearie KL, Williamson PA, Vaidyanathan S, et al. Disconnect between standardized field-based testing and mannitol challenge in Scottish elite swimmers. *Clin Exp allergy J Br Soc Allergy Clin Immunol*. 2010;40(5):731-737. doi:10.1111/j.1365-2222.2010.03461.x

565. Cleland I, Nugent CD, Finlay DD, et al. Effects of BMI and abdominal volume on the accuracy of step count obtained from a tri-axial accelerometer. *Annu Int Conf IEEE Eng Med Biol Soc IEEE Eng Med Biol Soc Annu Int Conf*. 2011;2011:3656-3659. doi:10.1109/IEMBS.2011.6090616

566. Coban YK, Uzel M, Balik O. Plantar burns after sunbathing and walking of a patient with complete spinal cord injury. *Burns*. 2005;31(3):398-399. doi:10.1016/j.burns.2004.10.024

567. Cobb WS, Burns JM, Kercher KW, Matthews BD, James Norton H, Todd Heniford B. Normal intraabdominal pressure in healthy adults. *J Surg Res*. 2005;129(2):231-235. doi:10.1016/j.jss.2005.06.015

568. Coda A, Sculley D, Santos D, et al. Harnessing interactive technologies to improve health outcomes in juvenile idiopathic arthritis. *Pediatr Rheumatol Online J*. 2017;15(1):40. doi:10.1186/s12969-017-0168-y

569. Coetsee M. Consensus, convergence, and COVID-19: The ethical role of religious reasons in leaders’ response to COVID-19. *LEADERSHIP*. 2022;18(3):446-464. doi:10.1177/17427150211064402

570. Coggan AR. Plasma glucose metabolism during exercise: effect of endurance training in humans. *Med Sci Sports Exerc*. 1997;29(5):620-627. doi:10.1097/00005768-199705000-00006

571. Coggan AR. Muscle biopsy as a tool in the study of aging. *J Gerontol A Biol Sci Med Sci*. 1995;50 Spec No:30-34. doi:10.1093/gerona/50a.special_issue.30

572. Coggan AR. The glucose crossover concept is not an important new concept in exercise metabolism. *Clin Exp Pharmacol Physiol*. 1997;24(11):896-900. doi:10.1111/j.1440-1681.1997.tb02713.x

573. Coggan AR, Raguso CA, Gastaldelli A, Sidossis LS, Yeckel CW. Fat metabolism during high-intensity exercise in endurance-trained and untrained men. *Metabolism*. 2000;49(1):122-128. doi:10.1016/s0026-0495(00)90963-6

574. Coggan AR, Raguso CA, Gastaldelli A, Williams BD, Wolfe RR. Regulation of glucose production during exercise at 80% of VO2peak in untrained humans. *Am J Physiol*. 1997;273(2 Pt 1):E348-54. doi:10.1152/ajpendo.1997.273.2.E348

575. Coggan AR, Raguso CA, Williams BD, Sidossis LS, Gastaldelli A. Glucose kinetics during high-intensity exercise in endurance-trained and untrained humans. *J Appl Physiol*. 1995;78(3):1203-1207. doi:10.1152/jappl.1995.78.3.1203

576. Coggan AR, Swanson SC, Mendenhall LA, Habash DL, Kien CL. Effect of endurance training on hepatic glycogenolysis and gluconeogenesis during prolonged exercise in men. *Am J Physiol*. 1995;268(3 Pt 1):E375-83. doi:10.1152/ajpendo.1995.268.3.E375

577. Cohen L, Brown J, Haukness H, Walsh L, Robinson JK. Sun protection counseling by pediatricians has little effect on parent and child sun protection behavior. *J Pediatr*. 2013;162(2):381-386. doi:10.1016/j.jpeds.2012.07.045

578. Cohen S, Nathan JA, Goldberg AL. Muscle wasting in disease: molecular mechanisms and promising therapies. *Nat Rev Drug Discov*. 2015;14(1):58-74. doi:10.1038/nrd4467

579. Colditz GA. Carpe Diem: time to seize the opportunity for cancer prevention. *Am Soc Clin Oncol Educ book Am Soc Clin Oncol Annu Meet*. Published online 2014:8-12. doi:10.14694/EdBook_AM.2014.34.8

580. Colfer HT, Ribner HS, Gradman A, Hughes C V, Kapoor A, Laidlaw JC. Effects of once-daily benazepril therapy on exercise tolerance and manifestations of chronic congestive heart failure. The Benazepril Heart Failure Study Group. *Am J Cardiol*. 1992;70(3):354-358. doi:10.1016/0002-9149(92)90618-9

581. Colford JMJ, Wade TJ, Schiff KC, et al. Water quality indicators and the risk of illness at beaches with nonpoint sources of fecal contamination. *Epidemiology*. 2007;18(1):27-35. doi:10.1097/01.ede.0000249425.32990.b9

582. Coll F, Hill K, Burrows S, Watson C, Edgar D. Modified Chester Step Test in a Healthy Adult Population: Measurement Properties and Development of a Regression Equation to Estimate Test Duration. *Phys Ther*. 2020;100(8):1411-1418. doi:10.1093/ptj/pzaa088

583. Condello G, Puggina A, Aleksovska K, et al. Behavioral determinants of physical activity across the life course: a “DEterminants of DIet and Physical ACtivity” (DEDIPAC) umbrella systematic literature review. *Int J Behav Nutr Phys Act*. 2017;14(1):58. doi:10.1186/s12966-017-0510-2

584. Cone DC, MacMillan DS, Van Gelder C, Brown DJ, Weir SD, Bogucki S. Noninvasive fireground assessment of carboxyhemoglobin levels in firefighters. *Prehospital Emerg care*. 2005;9(1):8-13. doi:10.1080/10903120590891912

585. Conlon KM, Martin S. “Just send them all to a burn centre”: managing burn resources in a mass casualty incident. *J Bus Contin Emer Plan*. 2011;5(2):150-160.

586. Conn AS, Hall MS, Quinn K, Wiggins B, Memmott C, Brusseau TAJ. An Examination of a Yoga Intervention With Pediatric Burn Survivors. *J Burn care Res Off Publ Am Burn Assoc*. 2017;38(1):e337-e342. doi:10.1097/BCR.0000000000000385

587. Coombes BK, Bisset LM, Sierra-Silvestre E, et al. Personal Activity Intelligence eHealth intervention in people with diabetic peripheral neuropathy: A feasibility study. *Aust J Gen Pract*. 2023;52(11):771-777. doi:10.31128/AJGP-04-23-6797

588. Coombes BK, Sierra-Silvestre E, Bisset LM, et al. Pain exacerbation following physical activity in adults with diabetic neuropathy: Ecological momentary assessment of foot symptoms. *Aust J Gen Pract*. 2024;53(3):93-98. doi:10.31128/AJGP-05-23-6821

589. Coons D, Godleski M. Range of motion exercises in the setting of burn-associated heterotopic ossification at the elbow: case series and discussion. *Burns*. 2013;39(4):e34-8. doi:10.1016/j.burns.2012.10.014

590. Cooper MT. Common Painful Foot and Ankle Conditions: A Review. *JAMA*. 2023;330(23):2285-2294. doi:10.1001/jama.2023.23906

591. Coppa LM, Nehal KS, Young JW, Halpern AC. Erythromelalgia precipitated by acral erythema in the setting of thrombocytopenia. *J Am Acad Dermatol*. 2003;48(6):973-975. doi:10.1067/mjd.2003.304

592. Cornet PA, Niemeijer AS, Figaroa GD, et al. Clinical outcome of patients with self-inflicted burns. *Burns*. 2017;43(4):789-795. doi:10.1016/j.burns.2016.11.005

593. Corrigan E, Samrasinghe I. Disaster preparedness in an Australian urban trauma center: staff knowledge and perceptions. *Prehospital Disaster Med*. 2012;27(5):432-438. doi:10.1017/S1049023X12001045

594. Cortis C, Puggina A, Pesce C, et al. Psychological determinants of physical activity across the life course: A “DEterminants of DIet and Physical ACtivity” (DEDIPAC) umbrella systematic literature review. *PLoS One*. 2017;12(8):e0182709. doi:10.1371/journal.pone.0182709

595. Costanzo V. Critically appraised paper: In adults with severe lower limb burns, adding a Wii Fit program to usual physiotherapy improves lower limb function and functional mobility [commentary]. *J Physiother*. 2022;68(4):277. doi:10.1016/j.jphys.2022.08.001

596. Costanzo V, Cavalheri V. Critically appraised paper: In adults with severe lower limb burns, adding a Wii Fit program to usual physiotherapy improves lower limb function and functional mobility. *J Physiother*. 2022;68(4):277. doi:10.1016/j.jphys.2022.08.002

597. Coughenour C, Burns MS. Community Design Impacts on Health Habits in Low-income Southern Nevadans. *Am J Health Behav*. 2016;40(4):534-544. doi:10.5993/AJHB.40.4.15

598. Counce JS, Cone JB, McAlister L, Wallace B, Caldwell FTJ. Surgical complications of thermal injury. *Am J Surg*. 1988;156(6):556-557. doi:10.1016/s0002-9610(88)80552-x

599. Cowgill LW. Burn or why your treadmill hour never budges the scale. *Am J Biol Anthropol*. 2022;177(1):182-183. doi:10.1002/ajpa.24409

600. Cox GW, Griswold JA. Current therapy of hand burn injuries. *J Miss State Med Assoc*. 1991;32(9):339-342.

601. Cox JL, Wright LM, Burns RJ. Prognostic significance of increased thallium-201 lung uptake during dipyridamole myocardial scintigraphy: comparison with exercise scintigraphy. *Can J Cardiol*. 1995;11(8):689-694.

602. Craig T V, Rhodes RE, Sui W. Examining and Comparing the Energy Expenditure of Two Modes of a Virtual Reality Fitness Game (Supernatural): Indirect Calorimetry Study. *JMIR serious games*. 2024;12:e53999. doi:10.2196/53999

603. Craig TJ, Henao MP. Advances in managing COPD related to α(1) -antitrypsin deficiency: An under-recognized genetic disorder. *Allergy*. 2018;73(11):2110-2121. doi:10.1111/all.13558

604. Cramer MN, Moralez G, Huang M, Kouda K, Poh PYS, Crandall CG. Exercise Core Temperature Response with a Simulated Burn Injury: Effect of Body Size. *Med Sci Sports Exerc*. 2020;52(3):705-711. doi:10.1249/MSS.0000000000002160

605. Cramer MN, Gagnon D, Laitano O, Crandall CG. Human temperature regulation under heat stress in health, disease, and injury. *Physiol Rev*. 2022;102(4):1907-1989. doi:10.1152/physrev.00047.2021

606. CRAMER MN, HUANG MU, FISCHER M, MORALEZ G, CRANDALL CG. Thermoregulatory Responses with Size-matched Simulated Torso or Limb Skin Grafts. *Med Sci Sport Exerc*. 2021;53(10):2190-2195. doi:10.1249/MSS.0000000000002694

607. CRAMER MN, MORALEZ G, HUANG MU, CRANDALL CG. No Thermoregulatory Impairment in Skin Graft Donor Sites during Exercise-Heat Stress. *Med Sci Sport Exerc*. 2019;51(5):868-873. doi:10.1249/MSS.0000000000001883

608. CRAMER MN, MORALEZ G, HUANG MU, KOUDA KEN, POH PYS, CRANDALL CG. Exercise Thermoregulation with a Simulated Burn Injury: Impact of Air Temperature. *Med Sci Sport Exerc*. 2020;52(3):712-719. doi:10.1249/MSS.0000000000002184

609. Cramer-Kruit JJJ, Akkerman M, Mouton LJ, et al. Fatigue in children and adolescents after burns: evaluating the problem using longitudinal data. *Disabil Rehabil*. 2024;46(13):2828-2838. doi:10.1080/09638288.2023.2232729

610. Crandall CG, Cramer MN, Kowalske KJ. Edward F. Adolph Distinguished Lecture. It’s more than skin deep: thermoregulatory and cardiovascular consequences of severe burn injuries in humans. *J Appl Physiol*. 2021;131(6):1852-1866. doi:10.1152/japplphysiol.00620.2021

611. Crandall CG, Davis SL. Cutaneous vascular and sudomotor responses in human skin grafts. *J Appl Physiol*. 2010;109(5):1524-1530. doi:10.1152/japplphysiol.00466.2010

612. Crawford C. The burn rehabilitation swimming program. *J Burn Care Rehabil*. 1988;9(3):290-291.

613. Crawford CM, Varghese G, Mani MM, Neff JR. Heterotopic ossification: are range of motion exercises contraindicated? *J Burn Care Rehabil*. 1986;7(4):323-327. doi:10.1097/00004630-198607000-00005

614. Cree MG, Aarsland A, Herndon DN, Wolfe RR. Role of fat metabolism in burn trauma-induced skeletal muscle insulin resistance. *Crit Care Med*. 2007;35(9 Suppl):S476-83. doi:10.1097/01.CCM.0000278066.05354.53

615. Cretoiu SM, Zugravu CA. Nutritional Considerations in Preventing Muscle Atrophy. In: Xiao J, ed. *MUSCLE ATROPHY*. Vol 1088. ; 2018:497-528. doi:10.1007/978-981-13-1435-3_23

616. Crocker T, Forster A, Young J, et al. Physical rehabilitation for older people in long-term care. *Cochrane database Syst Rev*. 2013;(2):CD004294. doi:10.1002/14651858.CD004294.pub3

617. Cronan T, Hammond J, Ward CG. The value of isokinetic exercise and testing in burn rehabilitation and determination of back-to-work status. *J Burn Care Rehabil*. 1990;11(3):224-227. doi:10.1097/00004630-199005000-00008

618. Crosbie J, Burns J. Are in-shoe pressure characteristics in symptomatic idiopathic pes cavus related to the location of foot pain? *Gait Posture*. 2008;27(1):16-22. doi:10.1016/j.gaitpost.2006.12.013

619. Cucuzzo NA, Ferrando A, Herndon DN. The effects of exercise programming vs traditional outpatient therapy in the rehabilitation of severely burned children. *J Burn Care Rehabil*. 2001;22(3 CC-Child Health):214‐220. doi:10.1097/00004630-200105000-00006

620. Cucuzzo NA, Ferrando A, Herndon DN. The Effects of Exercise Programming vs Traditional Burned Children. *J Burn Care Rehabil*. 2001;22(3):214-220.

621. Cui Z, Yang X, Shou J, Wang G. [Effectiveness of scar split thickness skin graft combined with acellular allogeneic dermis in treatment of large deep II degree burn scar]. *Zhongguo xiu fu chong jian wai ke za zhi = Zhongguo xiufu chongjian waike zazhi = Chinese J reparative Reconstr Surg*. 2014;28(12):1502-1504.

622. Cuijpers MD, Baartmans MGA, Joosten KFM, et al. The efficacy of therapeutic interventions on paediatric burn patients’ height, weight, body composition, and muscle strength: A systematic review and meta-analysis. *Burns*. 2024;50(6):1437-1455. doi:10.1016/j.burns.2024.03.012

623. Cundiff DK, Nigg CR. Diet and diabetic retinopathy: insights from the Diabetes Control and Complications Trial (DCCT). *MedGenMed*. 2005;7(1):3.

624. Cutting KF, White R. Defined and refined: criteria for identifying wound infection revisited. *Br J Community Nurs*. 2004;9(3):S6-15. doi:10.12968/bjcn.2004.9.Sup1.12495

625. Cutts RR, Burns SP. Resistance and Aerobic Training Sequence Effects on Energy Consumption in Females. *Int J Exerc Sci*. 2010;3(3):143-149.

626. CW H, Crighton E, Dziewulski P, Horner CWM, Crighton E, Dziewulski P. 30 years of burn disasters within the UK: guidance for UK emergency preparedness. *Burn*. 2012;38(4):578-584. doi:10.1016/j.burns.2011.10.007

627. da Cunha Moraes G, Vitoretti LB, de Brito AA, et al. Low-Level Laser Therapy Reduces Lung Inflammation in an Experimental Model of Chronic Obstructive Pulmonary Disease Involving P2X7 Receptor. *Oxid Med Cell Longev*. 2018;2018:6798238. doi:10.1155/2018/6798238

628. da Silva MMM, Travensolo C de F, Probst VS, Felcar JM. Quantification of changes in functional capacity and muscle strength in patients: a burn intensive care unit cohort study. *Burns*. 2022;48(4):833-840. doi:10.1016/j.burns.2022.01.010

629. Dal Lago D, Burns E, Gaunt E, Peers E, Jackson RC, Wilcockson TDW. Alcohol Use Predicts Face Perception Impairments and Difficulties in Face Recognition. *Subst Use Misuse*. 2023;58(13):1734-1741. doi:10.1080/10826084.2023.2247059

630. Dal Lago D, Burns E, Jackson RC, Wilcockson TDW. Are alcohol-related attentional biases and holistic perception independent processes? *Exp Clin Psychopharmacol*. 2024;32(5):579-587. doi:10.1037/pha0000727

631. Damanti S, Senini E, De Lorenzo R, et al. Acute Sarcopenia: Mechanisms and Management. *Nutrients*. 2024;16(20). doi:10.3390/nu16203428

632. Damas J, Garbacki N, Liégeois JF, Juchmes J. [Control of cutaneous blood vessels]. *Rev Med Liege*. 2001;56(12):846-849.

633. Daniels P, Burns RD, Brusseau TA, et al. Effect of a randomised 12-week resistance training programme on muscular strength, cross-sectional area and muscle quality in women having undergone Roux-en-Y gastric bypass. *J Sports Sci*. 2018;36(5):529-535. doi:10.1080/02640414.2017.1322217

634. D’Antono B, Dupuis G, Fortin C, Arsenault A, Burelle D. Detection of exercise-induced myocardial ischemia from symptomatology experienced during testing in men and women. *Can J Cardiol*. 2006;22(5):411-417. doi:10.1016/s0828-282x(06)70927-8

635. Darnall BD, Ziadni MS, Roy A, et al. Comparative Efficacy and Mechanisms of a Single-Session Pain Psychology Class in Chronic Low Back Pain: Study Protocol for a Randomized Controlled Trial. *Trials*. 2018;19:1. doi:10.1186/s13063-018-2537-3

636. Daskalopoulou SS, Rabi DM, Zarnke KB, et al. The 2015 Canadian Hypertension Education Program recommendations for blood pressure measurement, diagnosis, assessment of risk, prevention, and treatment of hypertension. *Can J Cardiol*. 2015;31(5):549-568. doi:10.1016/j.cjca.2015.02.016

637. Dauber A, PF O, AJ B, HL V, DB C. Chronic persistent pain after severe burns: a survey of 358 burn survivors. *Pain Med*. 2002;3(1):6-17. doi:10.1046/j.1526-4637.2002.02004.x

638. David TE, Burns RJ, Bacchus CM, Druck MN. Mitral valve replacement for mitral regurgitation with and without preservation of chordae tendineae. *J Thorac Cardiovasc Surg*. 1984;88(5 Pt 1):718-725.

639. Davies JC, Ravichandiran M, Agur AM, Fattah A. Evaluation of clinically relevant landmarks of the marginal mandibular branch of the facial nerve: A three-dimensional study with application to avoiding facial nerve palsy. *Clin Anat*. 2016;29(2):151-156. doi:10.1002/ca.22570

640. Davis F. Therapeutic Massage Provides Pain Relief to a Client with Morton’s Neuroma: A Case Report. *Int J Ther Massage Bodywork*. 2012;5(2):12-19.

641. Davis J, Taira DA, Lim E, Chen J. Daily Moderate-to-Vigorous Activity of Native Hawaiians and Pacific Islanders and Seven Asian Subgroups by Types of Activities, American Time Use Survey, 2010-2019. *Healthc (Basel, Switzerland)*. 2024;12(2). doi:10.3390/healthcare12020205

642. Davis SL, Shibasaki M, Low DA, et al. Skin grafting impairs postsynaptic cutaneous vasodilator and sweating responses. *J Burn care Res Off Publ Am Burn Assoc*. 2007;28(3):435-441. doi:10.1097/BCR.0B013E318053d32E

643. Davis SL, Shibasaki M, Low DA, et al. Impaired cutaneous vasodilation and sweating in grafted skin during whole-body heating. *J Burn care Res Off Publ Am Burn Assoc*. 2007;28(3):427-434. doi:10.1097/BCR.0B013E318053D312

644. Davis SL, Shibasaki M, Low DA, et al. Sustained impairments in cutaneous vasodilation and sweating in grafted skin following long-term recovery. *J Burn care Res Off Publ Am Burn Assoc*. 2009;30(4):675-685. doi:10.1097/BCR.0b013e3181abfd43

645. Day MA, Ehde DM, Burns J, et al. A randomized trial to examine the mechanisms of cognitive, behavioral and mindfulness-based psychosocial treatments for chronic pain: Study protocol. *Contemp Clin Trials*. 2020;93:106000. doi:10.1016/j.cct.2020.106000

646. de Arruda Mello PA, Yannoulis NC, Haque RM. Safety of unoprostone isopropyl as mono- or adjunctive therapy in patients with primary open-angle glaucoma or ocular hypertension. *Drug Saf*. 2002;25(8):583-597. doi:10.2165/00002018-200225080-00004

647. de Baère T, Risse O, Kuoch V, et al. Adverse events during radiofrequency treatment of 582 hepatic tumors. *AJR Am J Roentgenol*. 2003;181(3):695-700. doi:10.2214/ajr.181.3.1810695

648. de Figueiredo TB, Utsunomiya KF, de Oliveira AMRR, Pires-Neto RC, Tanaka C. Mobilization practices for patients with burn injury in critical care. *Burns*. 2020;46(2):314-321. doi:10.1016/j.burns.2019.07.037

649. de Jong AEE, Gamel C. Use of a simple relaxation technique in burn care: literature review. *J Adv Nurs*. 2006;54(6):710-721. doi:10.1111/j.1365-2648.2006.03858.x

650. De La Garza R 2nd, Yoon JH, Thompson-Lake DGY, et al. Treadmill exercise improves fitness and reduces craving and use of cocaine in individuals with concurrent cocaine and tobacco-use disorder. *Psychiatry Res*. 2016;245:133-140. doi:10.1016/j.psychres.2016.08.003

651. de Lange P, Lombardi A, Silvestri E, Goglia F, Lanni A, Moreno M. Peroxisome Proliferator-Activated Receptor Delta: A Conserved Director of Lipid Homeostasis through Regulation of the Oxidative Capacity of Muscle. *PPAR Res*. 2008;2008:172676. doi:10.1155/2008/172676

652. de Lateur BJ, Magyar-Russell G, Bresnick MG, et al. Augmented exercise in the treatment of deconditioning from major burn injury. *Arch Phys Med Rehabil*. 2007;88(12 Suppl 2 CC-SR-REHAB):S18‐23. doi:10.1016/j.apmr.2007.09.003

653. de Lateur BJ, Shore WS. Exercise following burn injury. *Phys Med Rehabil Clin N Am*. 2011;22(2):347-350, vii. doi:10.1016/j.pmr.2011.02.003

654. de Macêdo MRC, Marques RF, Silva AJS, Navarro F, Coppi Navarro A. Systematic Review: Models of Changes in Gene Expression of MTOR, MURF-1, and MAFBX in Rats and Mice. *Crit Rev Eukaryot Gene Expr*. 2020;30(1):57-75. doi:10.1615/CritRevEukaryotGeneExpr.2020027491

655. de Paula CS, Donlevy G, Cardoso J, et al. Translating a Clinical Practice Guideline to a Portuguese, Spanish and English Practice Brief to promote exercise therapy for paediatric Charcot-Marie-Tooth disease. *Physiotherapy*. 2024;125:101419. doi:10.1016/j.physio.2024.101419

656. de Sousa LN, Sant’ana DSP, Siqueira Dos Santos RG, et al. Involvement of serotonergic pathways in gastric dysmotility induced by fat burning nutritional supplements in mice. *Curr Res Pharmacol drug Discov*. 2021;2:100018. doi:10.1016/j.crphar.2021.100018

657. Dean JM, Murton AJ, Glover SQ, et al. Use of Isokinetic Dynamometry To Assess Muscle Function In Burned Patients Is A Reliable Tool To Assist Progressive Resistance Exercise Prescription. *J Burn CARE Res*. 2023;44(3):546-550. doi:10.1093/jbcr/irz003

658. Deans KA, Bezlyak V, Ford I, et al. Differences in atherosclerosis according to area level socioeconomic deprivation: cross sectional, population based study. *BMJ*. 2009;339:b4170. doi:10.1136/bmj.b4170

659. Deans S, Burns D, McGarry A, et al. Motivations and barriers to prosthesis users participation in physical activity, exercise and sport: a review of the literature. *Prosthetics Orthot Int*. 2012;36(3):260-269. doi:10.1177/0309364612437905

660. Deflorin C, Hohenauer E, Stoop R, van Daele U, Clijsen R, Taeymans J. Physical Management of Scar Tissue: A Systematic Review and Meta-Analysis. *J Altern Complement Med*. 2020;26(10):854-865. doi:10.1089/acm.2020.0109

661. Deflorin C, Hohenauer E, Stoop R, van Daele U, Clijsen R, Taeymans J. Response to Vercelli et al. re: “Physical Management of Scar Tissue: A Systematic Review and Meta-Analysis”. *J Altern Complement Med*. 2021;27(4):374-376. doi:10.1089/acm.2020.29089.cde

662. DeFor TE, Burns LJ, Gold EMA, Weisdorf DJ. A randomized trial of the effect of a walking regimen on the functional status of 100 adult allogeneic donor hematopoietic cell transplant patients. *Biol blood marrow Transplant J Am Soc Blood Marrow Transplant*. 2007;13(8):948-955. doi:10.1016/j.bbmt.2007.04.008

663. Degtiareva SA, Shmelev EI, Smirnov S V, Brygin PA. [Stepwise treatment in patients with isolated thermal inhalation injury]. *Ter Arkh*. 2013;85(8):56-59.

664. Dehghani GA, Parvizi MR, Sharif-Kazemi MB, Raj H, Anand A, Paintal AS. Presence of lobeline-like sensations in exercising patients with left ventricular dysfunction. *Respir Physiol Neurobiol*. 2004;143(1):9-20. doi:10.1016/j.resp.2004.07.003

665. Delaney K, Canty J. The development of a sunscreen. *Aust Fam Physician*. 1976;5(1):55-68.

666. Delye H, Lagae L, Vermylen J, Nuttin B. Thalamic stimulation as a treatment for primary erythromelalgia: technical case report. *Neurosurgery*. 2005;57(4 Suppl):E404; discussion E404. doi:10.1227/01.neu.0000176703.27632.6d

667. Demashkieh M, Dalan R, Burns SF. Cardiorespiratory fitness and fat oxidation during exercise in Chinese, Indian, and Malay men with elevated body mass index. *Appl Physiol Nutr Metab*. 2022;47(8):888-892. doi:10.1139/apnm-2022-0106

668. Demling RH, DeSanti L. The rate of restoration of body weight after burn injury, using the anabolic agent oxandrolone, is not age dependent. *Burns*. 2001;27(1 CC-Metabolic and Endocrine Disorders CC-Injuries):46‐51. doi:10.1016/s0305-4179(00)00064-4

669. Demling RH, DeSanti L. Oxandrolone induced lean mass gain during recovery from severe burns is maintained after discontinuation of the anabolic steroid. *Burns*. 2003;29(8 CC-Metabolic and Endocrine Disorders):793‐797. doi:10.1016/j.burns.2003.08.003

670. Deng H, Abouzeid CA, Shepler LJ, et al. Moderation Effects of Daily Behavior on Associations Between Symptoms and Social Participation Outcomes After Burn Injury: A 6-Month Digital Phenotyping Study. *Arch Phys Med Rehabil*. 2024;105(9):1700-1708. doi:10.1016/j.apmr.2024.05.011

671. Deng H, Chen J, Li F, et al. Effects of mobility training on severe burn patients in the BICU: A retrospective cohort study. *Burns*. 2016;42(7):1404-1412. doi:10.1016/j.burns.2016.07.029

672. Desai MH, Mlcak RP, Robinson E, et al. Does inhalation injury limit exercise endurance in children convalescing from thermal injury? *J Burn Care Rehabil*. 1993;14(1):12-16. doi:10.1097/00004630-199301000-00004

673. Detres LL. [Taking control with our diet]. *SIDAhora : un proyecto del Departamento de Publicaciones del PWA Coalition, NY*. 1995:11-14.

674. Dewey WS, Cunningham KB, Shingleton SK, Pruskowski KA, Welsh A, Rizzo JA. Safety of Early Postoperative Range of Motion in Burn Patients With Newly Placed Hand Autografts: A Pilot Study. *J Burn care Res Off Publ Am Burn Assoc*. 2020;41(4):809-813. doi:10.1093/jbcr/iraa072

675. Dhawan S, Andrews R, Kumar L, Wadhwa S, Shukla G. A Randomized Controlled Trial to Assess the Effectiveness of Muscle Strengthening and Balancing Exercises on Chemotherapy-Induced Peripheral Neuropathic Pain and Quality of Life Among Cancer Patients. *Cancer Nurs*. 2020;43(4 CC-Gynaecological, Neuro-oncology and Orphan Cancer):269‐280. doi:10.1097/NCC.0000000000000693

676. Dib-Hajj SD, Rush AM, Cummins TR, et al. Gain-of-function mutation in Nav1.7 in familial erythromelalgia induces bursting of sensory neurons. *Brain*. 2005;128(Pt 8):1847-1854. doi:10.1093/brain/awh514

677. Dickerson D, Baldwin JA, Belcourt A, et al. Encompassing Cultural Contexts Within Scientific Research Methodologies in the Development of Health Promotion Interventions. *Prev Sci*. 2020;21:S33-S42. doi:10.1007/s11121-018-0926-1

678. Diego AM, Serghiou M, Padmanabha A, Porro LJ, Herndon DN, Suman OE. Exercise training after burn injury: a survey of practice. *J Burn care Res Off Publ Am Burn Assoc*. 2013;34(6):e311-7. doi:10.1097/BCR.0b013e3182839ae9

679. Dikhit PS, Srivastava A, Boyena KK. Injury to the oral mucosa by organophosphates without systemic toxicity: a rare case. *Br J Oral Maxillofac Surg*. 2018;56(8):755-757. doi:10.1016/j.bjoms.2018.07.016

680. Dikkema Y, Mouton N, Gerrits K, et al. Identification and Quantification of Activities Common to Intensive Care Patients; Development and Validation of a Dual-Accelerometer-Based Algorithm. *Sensors (Basel)*. 2023;23(3). doi:10.3390/s23031720

681. Dinyer TK, Byrd MT, Garver MJ, et al. Low-Load vs. High-Load Resistance Training to Failure on One Repetition Maximum Strength and Body Composition in Untrained Women. *J strength Cond Res*. 2019;33(7):1737‐1744. doi:10.1519/JSC.0000000000003194

682. Dionisi B, Anglana F, Inghirami P, Lippa P, Senatori R. [Use of transcutaneous electrical stimulation and biofeedback for the treatment of vulvodynia (vulvar vestibular syndrome): result of 3 years of experience]. *Minerva Ginecol*. 2008;60(6):485-491.

683. Disseldorp LM, Mouton LJ, Takken T, et al. Design of a cross-sectional study on physical fitness and physical activity in children and adolescents after burn injury. *BMC Pediatr*. 2012;12:195. doi:10.1186/1471-2431-12-195

684. Disseldorp LM, Mouton LJ, Takken T, et al. Design of a cross-sectional study on physical fitness and physical activity in children and adolescents after burn injury. *BMC Pediatr*. 2012;12(1):195. doi:10.1186/1471-2431-12-195

685. Disseldorp LM, Mouton LJ, Van der Woude LH V, Van Brussel M, Nieuwenhuis MK. Anthropometry, muscular strength and aerobic capacity up to 5 years after pediatric burns. *Burn*. 2015;41(8):1839-1846. doi:10.1016/j.burns.2015.08.025

686. Disseldorp LM, Nieuwenhuis MK, Van Baar ME, Mouton LJ. Physical fitness in people after burn injury: a systematic review. *Arch Phys Med Rehabil*. 2011;92(9):1501-1510. doi:10.1016/j.apmr.2011.03.025

687. Ditunno JFJ, Ditunno PL, Scivoletto G, et al. The Walking Index for Spinal Cord Injury (WISCI/WISCI II): nature, metric properties, use and misuse. *Spinal Cord*. 2013;51(5):346-355. doi:10.1038/sc.2013.9

688. Do W, Kang D, Hong P, Kim HJ, Baik J, Lee D. Incidental operating room fire from a breathing circuit warmer system: a case report. *BMC Anesthesiol*. 2021;21(1). doi:10.1186/s12871-021-01488-2

689. Dodd H, Fletchall S, Starnes C, Jacobson K. Current Concepts Burn Rehabilitation, Part II: Long-Term Recovery. *Clin Plast Surg*. 2017;44(4):713-728. doi:10.1016/j.cps.2017.05.013

690. Doherty CJ, Incognito A V, Notay K, et al. Muscle sympathetic nerve responses to passive and active one-legged cycling: insights into the contributions of central command. *Am J Physiol Circ Physiol*. 2018;314(1):H3-H10. doi:10.1152/ajpheart.00494.2017

691. Doig GS, Simpson F, Heighes PT, et al. Restricted versus continued standard caloric intake during the management of refeeding syndrome in critically ill adults: a randomised, parallel-group, multicentre, single-blind controlled trial. *Lancet Respir Med*. 2015;3(12):943-952. doi:10.1016/S2213-2600(15)00418-X

692. Dombrecht D, Van Daele U, Van Asbroeck B, et al. Molecular mechanisms of post-burn muscle wasting and the therapeutic potential of physical exercise. *J Cachexia Sarcopenia Muscle*. 2023;14(2):758-770. doi:10.1002/jcsm.13188

693. Dona AC, Jewett P, Henning-Smith C, Ahmed RL, Lazovich D, Vogel RI. Rural-urban differences in meeting of physical activity recommendations by sun exposure and protection behaviors in the United States. *Prev Med (Baltim)*. 2024;181:107919. doi:10.1016/j.ypmed.2024.107919

694. Donahue PT, Grove G, Stillman C, et al. Estimating the financial costs associated with a phase III, multi-site exercise intervention trial: Investigating Gains in Neurocognition in an Intervention Trial of Exercise (IGNITE). *Contemp Clin Trials*. 2021;105. doi:10.1016/j.cct.2021.106401

695. Dong H, Qian LB, Cui YX, et al. Online Accurate Detection of Breath Acetone Using Metal Oxide Semiconductor Gas Sensor and Diffusive Gas Separation. *Front Bioeng Biotechnol*. 2022;10. doi:10.3389/fbioe.2022.861950

696. Dong L, Li J, Lian Y, et al. Long-Term Intensive Lifestyle Intervention Promotes Improvement of Stage III Diabetic Nephropathy. *Med Sci Monit*. 2019;25:3061-3068. doi:10.12659/MSM.913512

697. Dong X, Yu D. Application of cicatricial contracture release principles in muscular torticollis treatment. *Aesthetic Plast Surg*. 2013;37(5):950-955. doi:10.1007/s00266-013-0122-4

698. Donnelly S, Collins K, Burns C, O’Neill C, Mangan S. A Comparison of Elite and Sub-elite Match-Play Running Performance of Gaelic Football Players. *J strength Cond Res*. 2024;38(5):912-923. doi:10.1519/JSC.0000000000004707

699. Dorling J, James LJ, King JA, et al. Acute and Chronic Effects of Exercise on Appetite, Energy Intake, and Appetite-Related Hormones: The Modulating Effect of Adiposity, Sex, and Habitual Physical Activity. *Nutrients*. 2018;10(9):1140. doi:10.3390/nu10091140

700. Dow L, Phelps L, Fowler L, Waters K, Coggon D, Holgate ST. Respiratory symptoms in older people and use of domestic gas appliances. *Thorax*. 1999;54(12):1104-1106. doi:10.1136/thx.54.12.1104

701. Dragoo JL, Johnson C, McConnell J. Evaluation and treatment of disorders of the infrapatellar fat pad. *Sports Med*. 2012;42(1):51-67. doi:10.2165/11595680-000000000-00000

702. Drucker AM, Kleiner O, Manion R, et al. Top Ten Research Priorities for Psoriasis, Atopic Dermatitis and Hidradenitis Suppurativa: The SkIN Canada Priority Setting Initiative. *J Cutan Med Surg*. 2023;27(2):133-139. doi:10.1177/12034754231156103

703. Du WL, Hu XH, Shen YM, Teng X. Surgical management of acute compartment syndrome and sequential complications. *BMC Musculoskelet Disord*. 2019;20. doi:10.1186/s12891-019-2476-5

704. Du Y, Lv GZ, Yu S, Wang D, Tan Q. Long-term medical treatment of patients with severe burns at exposed sites. *World J Clin cases*. 2020;8(16):3515-3526. doi:10.12998/wjcc.v8.i16.3515

705. Dudhagara DR, Rajpara RK, Bhatt JK, Gosai HB, Sachaniya BK, Dave BP. Distribution, sources and ecological risk assessment of PAHs in historically contaminated surface sediments at Bhavnagar coast, Gujarat, India. *Environ Pollut*. 2016;213:338-346. doi:10.1016/j.envpol.2016.02.030

706. Duke JM, Randall SM, Fear MW, Boyd JH, Rea S, Wood FM. Understanding the long-term impacts of burn on the cardiovascular system. *Burns*. 2016;42(2):366-374. doi:10.1016/j.burns.2015.08.020

707. Duncan CE. Use of a ramp surface for lower extremity exercise with burn-injured patients. *J Burn Care Rehabil*. 1989;10(4):346-349. doi:10.1097/00004630-198907000-00010

708. Duncan CE. Use of a pillow in exercises for burn patients. *J Burn Care Rehabil*. 1988;9(3):293.

709. Durant DJ, Colburn VF, Guerrazzi-Young C. Impact of a mindful self-compassion workshop incorporating sandtray on burnout in undergraduate nursing students: A mixed methods, pre-post study. *Nurse Educ Pract*. 2024;82:104203. doi:10.1016/j.nepr.2024.104203

710. Durão S, Burns J, Schmidt BM, et al. Infrastructure, policy and regulatory interventions to increase physical activity to prevent cardiovascular diseases and diabetes: a systematic review. *BMC Public Health*. 2023;23(1):112. doi:10.1186/s12889-022-14841-y

711. Durham WJ, Miller SL, Yeckel CW, et al. Leg glucose and protein metabolism during an acute bout of resistance exercise in humans. *J Appl Physiol*. 2004;97(4):1379-1386. doi:10.1152/japplphysiol.00635.2003

712. Dütsch M, Hilz MJ. Neurological complications in Fabry disease. *La Rev Med interne*. 2010;31 Suppl 2:S243-50. doi:10.1016/S0248-8663(10)70021-7

713. Dutta GP. Female tubal sterilisation. *J Indian Med Assoc*. 1979;72(8):193-194.

714. E L, Burns JM, Swerdlow RH. Effect of high-intensity exercise on aged mouse brain mitochondria, neurogenesis, and inflammation. *Neurobiol Aging*. 2014;35(11):2574-2583. doi:10.1016/j.neurobiolaging.2014.05.033

715. E L, Lu J, Burns JM, Swerdlow RH. Effect of exercise on mouse liver and brain bioenergetic infrastructures. *Exp Physiol*. 2013;98(1):207-219. doi:10.1113/expphysiol.2012.066688

716. E L, Lu J, Selfridge JE, Burns JM, Swerdlow RH. Lactate administration reproduces specific brain and liver exercise-related changes. *J Neurochem*. 2013;127(1):91-100. doi:10.1111/jnc.12394

717. Ebid AA, Omar MT, Abd El Baky AM. Effect of 12-week isokinetic training on muscle strength in adult with healed thermal burn. *Burns*. 2012;38(1):61‐68. doi:10.1016/j.burns.2011.05.007

718. Ebid AA, Attalla AF, Ibrahim AR, Mohamdy HM. Effect of anti-gravity treadmill (Alter G) training on gait characteristics and postural stability in adult with healed burns: A single blinded randomized controlled trial. *Burns*. 2024;50(1):106-114. doi:10.1016/j.burns.2023.09.004

719. Ebid AA, El-Shamy SM, Amer MA. Effect of vitamin D supplementation and isokinetic training on muscle strength, explosive strength, lean body mass and gait in severely burned children: A randomized controlled trial. *Burn*. 2017;43(2):357-365. doi:10.1016/j.burns.2016.08.018

720. Ebid AA, El-Shamy SM, Draz AH. Effect of isokinetic training on muscle strength , size and gait after healed pediatric burn : A randomized controlled study. *Burns*. 2014;40(1):97-105. doi:10.1016/j.burns.2013.05.022

721. Edger-Lacoursière Z, Zhu M, Jean S, Marois-Pagé E, Nedelec B. Evidence Supporting Conservative Scar Management Interventions Following Burn Injury: a review article. *J Burn care Res Off Publ Am Burn Assoc*. Published online November 2024. doi:10.1093/jbcr/irae204

722. Edionwe J, Hess C, Fernandez-Rio J, et al. Effects of whole-body vibration exercise on bone mineral content and density in thermally injured children. *Burns*. 2016;42(3):605‐613. doi:10.1016/j.burns.2015.10.017

723. Edlich RF, Muir A, Persing JA, et al. Special considerations in the management of a patient with multiple sclerosis and a burn injury. *J Burn Care Rehabil*. 1991;12(2):162-169. doi:10.1097/00004630-199103000-00016

724. Edouard P, Hollander K. After burning, the phoenix will be reborn from the ashes: possible measures for burnout prevention in Sports and Exercise Medicine academics. *BMJ open Sport Exerc Med*. 2023;9(4):e001809. doi:10.1136/bmjsem-2023-001809

725. Edstrom LE, Robson MC, Macchiaverna JR, Scala AD. Prospective randomized treatments for burned hands: nonoperative vs. operative. Preliminary report. *Scand J Plast Reconstr Surg*. 1979;13(1):131-135. doi:10.3109/02844317909013040

726. Edwards KM, Burns VE, Adkins AE, Carroll D, Drayson M, Ring C. Meningococcal A vaccination response is enhanced by acute stress in men. *Psychosom Med*. 2008;70(2):147-151. doi:10.1097/PSY.0b013e318164232e

727. Edwards KM, Burns VE, Allen LM, et al. Eccentric exercise as an adjuvant to influenza vaccination in humans. *Brain Behav Immun*. 2007;21(2):209-217. doi:10.1016/j.bbi.2006.04.158

728. Edwards KM, Burns VE, Carroll D, Drayson M, Ring C. The acute stress-induced immunoenhancement hypothesis. *Exerc Sport Sci Rev*. 2007;35(3):150-155. doi:10.1097/JES.0b013e3180a031bd

729. Edwards KM, Burns VE, Reynolds T, Carroll D, Drayson M, Ring C. Acute stress exposure prior to influenza vaccination enhances antibody response in women. *Brain Behav Immun*. 2006;20(2):159-168. doi:10.1016/j.bbi.2005.07.001

730. Edwards KM, Burns VE, Ring C, Carroll D. Sex differences in the interleukin-6 response to acute psychological stress. *Biol Psychol*. 2006;71(3):236-239. doi:10.1016/j.biopsycho.2005.06.006

731. Edwards KM, Burns VE, Ring C, Carroll D. Individual differences in the interleukin-6 response to maximal and submaximal exercise tasks. *J Sports Sci*. 2006;24(8):855-862. doi:10.1080/02640410500245645

732. Edwards KM, Campbell JP, Ring C, et al. Exercise intensity does not influence the efficacy of eccentric exercise as a behavioural adjuvant to vaccination. *Brain Behav Immun*. 2010;24(4):623-630. doi:10.1016/j.bbi.2010.01.009

733. Edwards L. New concepts in vulvodynia. *Am J Obstet Gynecol*. 2003;189(3 Suppl):S24-30. doi:10.1067/s0002-9378(03)00790-7

734. Eid MM, Abdelbasset WK, Abdelaty FM, Ali ZA. Effect of physical therapy rehabilitation program combined with music on children with lower limb burns: a twelve-week randomized controlled study. *Burns*. 2021;47(5):1146‐1152. doi:10.1016/j.burns.2020.11.006

735. Eikermann M, Velmahos G, Abbara S, et al. Case records of the Massachusetts General Hospital. Case 11-2014. A man with traumatic injuries after a bomb explosion at the Boston Marathon. *N Engl J Med*. 2014;370(15):1441-1451. doi:10.1056/NEJMcpc1314240

736. El Boghdady M, Ramakrishnan G, Alijani A. A study of the visual symptoms in two-dimensional versus three-dimensional laparoscopy. *Am J Surg*. 2018;216(6):1114‐1117. doi:10.1016/j.amjsurg.2018.07.051

737. ElAbd R, AlMojel M, AlSabah S, et al. Complications Post Abdominoplasty After Surgical Versus Non-surgical Massive Weight Loss: a Comparative Study. *Obes Surg*. 2022;32(12):3847-3853. doi:10.1007/s11695-022-06309-0

738. Elenkov IJ, Chrousos GP, Wilder RL. Neuroendocrine regulation of IL-12 and TNF-alpha/IL-10 balance. Clinical implications. *Ann N Y Acad Sci*. 2000;917:94-105. doi:10.1111/j.1749-6632.2000.tb05374.x

739. Ellingham TR. The immediate care of eye injuries. *Anaesthesia*. 1976;31(3):433-438. doi:10.1111/j.1365-2044.1976.tb12340.x

740. Ellis C, Burns D. All about oxygen: using near-infrared spectroscopy to understand bioenergetics. *Adv Physiol Educ*. 2022;46(4):685-692. doi:10.1152/advan.00106.2022

741. Ellis JPJ, Burns JW. Modified handling of internal catecholamine standards in radioenzymic assays. *Clin Chem*. 1983;29(1):144-147.

742. Ellis J, Warden J, Molassiotis A, et al. Participation in a randomised controlled feasibility study of a complex intervention for the management of the Respiratory Symptom Distress Cluster in lung cancer: patient, carer and research staff views. *Eur J Cancer Care (Engl)*. 2017;26(6). doi:10.1111/ecc.12538

743. Ellis RC, Kohler CL, Dvorak JE. Cryotherapy Induced Burns: A Case Series of Three Patients. *J Burn Care Res*. 2022;43(3):746-748. doi:10.1093/jbcr/irac027

744. El-Mazny A, El-Sharkawy M, Hassan A. A prospective randomized clinical trial comparing immediate versus delayed removal of urinary catheter following elective cesarean section. *Eur J Obstet Gynecol Reprod Biol*. 2014;181:111-114. doi:10.1016/j.ejogrb.2014.07.034

745. Elnaggar RK, Osailan AM, Alsubaie SF, Moawd SA, Abd El-Nabie WA. Graded aerobic exercise (GAEx): an effective exercise regimen to improve cardio-respiratory fitness and physical and psychosocial functioning in children with burn sequelae of the chest. *Burns*. 2022;48(2):337‐344. doi:10.1016/j.burns.2021.05.004

746. Elnaggar RK, Osailan AM, Mahmoud WS, Alqahtani BA, Azab AR. Beyond the Acute Phase: Understanding Relationships Among Cardiorespiratory Response to Exercises, Physical Activity Levels, and Quality of Life in Children After Burn Injuries. *J Burn CARE Res*. 2022;43(4):827-833. doi:10.1093/jbcr/irab203

747. Elnaggar RK, Osailan AM, Alsubaie SF, Moawd SA, El-nabie WAA. Graded aerobic exercise ( GAEx ): An effective exercise regimen to improve cardio-respiratory fitness and physical and psychosocial functioning in children with burn sequelae of the chest. *Burns*. 2021;48(2):337-344. doi:10.1016/j.burns.2021.05.004

748. Elnaggar RK, Osailan AM, Mahmoud WS, Alqahtani BA, Azab AR. Beyond the Acute Phase: Understanding Relationships Among Cardiorespiratory Response to Exercises, Physical Activity Levels, and Quality of Life in Children After Burn Injuries. *J Burn care Res Off Publ Am Burn Assoc*. 2022;43(4):827-833. doi:10.1093/jbcr/irab203

749. Elneima O, McAuley HJC, Leavy OC, et al. Cohort Profile: Post-Hospitalisation COVID-19 (PHOSP-COVID) study. *Int J Epidemiol*. 2024;53(1). doi:10.1093/ije/dyad165

750. El-Sayed Attalla AF, Ahmed KT, Abd El Monem M. Effects of Inspiratory Muscle Training on Clinical Predictors of Respiratory Muscle Strength and Lung Function in Burned Patients with Inhalation Injury. *J Burn care Res Off Publ Am Burn Assoc*. 2023;44(1):140-145. doi:10.1093/jbcr/irac163

751. Elsayed NM. Antioxidant mobilization in response to oxidative stress: a dynamic environmental-nutritional interaction. *Nutrition*. 2001;17(10):828-834. doi:10.1016/s0899-9007(01)00646-3

752. Emmett L, Iwanochko RM, Freeman MR, Barolet A, Lee DS, Husain M. Reversible regional wall motion abnormalities on exercise technetium-99m–gated cardiac single photon emission computed tomography predict high-grade angiographic stenoses. *J Am Coll Cardiol*. 2002;39(6):991-998. doi:10.1016/S0735-1097(02)01707-2

753. Emsen IM. Management of an unusual extreme extension contracture of an index finger: a custom-designed exercise program in achieving a good range of movement and prevention of recontraction with the reverse fasciocutaneous forearm flap and full-thickness skin graf. *Plast Reconstr Surg*. 2007;119(5):1630-1631. doi:10.1097/01.prs.0000256522.22911.ed

754. Emtner M, Porszasz J, Burns M, Somfay A, Casaburi R. Benefits of supplemental oxygen in exercise training in nonhypoxemic chronic obstructive pulmonary disease patients. *Am J Respir Crit Care Med*. 2003;168(9):1034-1042. doi:10.1164/rccm.200212-1525OC

755. Erickson KI, Grove GA, Burns JM, et al. Investigating Gains in Neurocognition in an Intervention Trial of Exercise (IGNITE): Protocol. *Contemp Clin Trials*. 2019;85:105832. doi:10.1016/j.cct.2019.105832

756. Espitia O, Dréno B, Cassagnau E, et al. Exercise-Induced Vasculitis: A Review with Illustrated Cases. *Am J Clin Dermatol*. 2016;17(6):635-642. doi:10.1007/s40257-016-0218-0

757. Esquivel M, Burns RJ, Ogilvie RI. Cardiovascular effects of enprofylline and theophylline. *Clin Pharmacol Ther*. 1986;39(4):395-402. doi:10.1038/clpt.1986.61

758. Evans EB. Orthopaedic measures in the treatment of severe burns. *J Bone Joint Surg Am*. 1966;48(4):643-669.

759. Everett T, Parker K, Fish J, et al. The construction and implementation of a novel postburn pruritus scale for infants and children aged five years or less: introducing the Toronto Pediatric Itch Scale. *J Burn care Res Off Publ Am Burn Assoc*. 2015;36(1):44-49. doi:10.1097/BCR.0000000000000129

760. Evon DM, Burns JW. Process and outcome in cardiac rehabilitation: an examination of cross-lagged effects. *J Consult Clin Psychol*. 2004;72(4):605-616. doi:10.1037/0022-006X.72.4.605

761. Fadeyibi IO, Ugburo AO, Ogunbanjo C V, Ilombu CA, Ademiluyi SA. The surgical repair of macrostomia. *Cleft palate-craniofacial J Off Publ Am Cleft Palate-Craniofacial Assoc*. 2009;46(6):642-647. doi:10.1597/07-178.1

762. Fadeyibi IO, Coker OA, Zacchariah MP, Fasawe A, Ademiluyi SA. Psychosocial effects of cleft lip and palate on Nigerians: the Ikeja-Lagos experience. *J Plast Surg Hand Surg*. 2012;46(1):13-18. doi:10.3109/2000656X.2011.643027

763. Falder S, Browne A, Edgar D, et al. Core outcomes for adult burn survivors: A clinical overview. *Burns*. 2009;35(5):618-641. doi:10.1016/j.burns.2008.09.002

764. Falvo MJ, Osinubi OY, Sotolongo AM, Helmer DA. Airborne hazards exposure and respiratory health of Iraq and Afghanistan veterans. *Epidemiol Rev*. 2015;37:116-130. doi:10.1093/epirev/mxu009

765. Fan PW, Burns SF, Lee JKW. Correction: Fan et al. Efficacy of Ingesting an Oral Rehydration Solution after Exercise on Fluid Balance and Endurance Performance. Nutrients 2020, 12, 3826. *Nutrients*. 2021;13(11). doi:10.3390/nu13113855

766. Fan PW, Burns SF, Lee JKW. Efficacy of Ingesting an Oral Rehydration Solution after Exercise on Fluid Balance and Endurance Performance. *Nutrients*. 2020;12(12):3826. doi:10.3390/nu12123826

767. Fanzani A, Conraads VM, Penna F, Martinet W. Molecular and cellular mechanisms of skeletal muscle atrophy: an update. *J Cachexia Sarcopenia Muscle*. 2012;3(3):163-179. doi:10.1007/s13539-012-0074-6

768. Fauré J, De Winne MT, Van Vlanderen W, Verbanck R. [Nursing and kinesitherapy in burns]. *Hosp Prog*. 1969;50(7):29-33.

769. Fearon KC, Plumb JA, Burns HJ, Calman KC. Reduction of the growth rate of the Walker 256 tumor in rats by rhodamine 6G together with hypoglycemia. *Cancer Res*. 1987;47(14):3684-3687.

770. Fee LL, Smith RM, English MB. Enhanced ventilatory and exercise performance in athletes with slight expiratory resistive loading. *J Appl Physiol*. 1997;83(2):503-510. doi:10.1152/jappl.1997.83.2.503

771. Feigel ED, Bird MB, Koltun KJ, et al. Association of clinically-measured and dynamic ankle dorsiflexion assessed by markerless motion capture during the drop-jump task on landing biomechanics and risk of ankle injury in military personnel undergoing 10 weeks of physical training. *J Sci Med Sport*. 2023;26(9):476-481. doi:10.1016/j.jsams.2023.07.012

772. Feldmann ME, Evans J, O SJ. Early management of the burned pediatric hand. *J Craniofac Surg*. 2008;19(4):942-950. doi:10.1097/SCS.0b013e318175f38d

773. Felker GM, Whellan D, Kraus WE, et al. N-terminal pro-brain natriuretic peptide and exercise capacity in chronic heart failure: data from the Heart Failure and a Controlled Trial Investigating Outcomes of Exercise Training (HF-ACTION) study. *Am Heart J*. 2009;158(4 Suppl):S37-44. doi:10.1016/j.ahj.2009.07.011

774. Fent KW, Mayer A, Bertke S, Kerber S, Smith D, Horn GP. Understanding airborne contaminants produced by different fuel packages during training fires. *J Occup Environ Hyg*. 2019;16(8):532-543. doi:10.1080/15459624.2019.1617870

775. Fenzl N, Bartsch K, Koenigstorfer J. Labeling exercise fat-burning increases post-exercise food consumption in self-imposed exercisers. *Appetite*. 2014;81:1‐7. doi:10.1016/j.appet.2014.05.030

776. Ferguson A, Wright S. Innovative chest physiotherapy techniques (the MetaNeb® System) in the intubated child with extensive burns. *Respir Med Case Reports*. 2017;22:232-234. doi:10.1016/j.rmcr.2017.08.020

777. Ferguson SL, Voll K V. Burn pain and anxiety: the use of music relaxation during rehabilitation. *J Burn Care Rehabil*. 2004;25(1 CC-HS-HANDSRCH CC-Complementary Medicine CC-SR-REHAB CC-Pain, Palliative and Supportive Care CC-Effective Practice and Organisation of Care):8‐14. doi:10.1097/01.BCR.0000105056.74606.9E

778. Fernandez S, Tobin JN, Cassells A, Diaz-Gloster M, Kalida C, Ogedegbe G. The counselling African Americans to control hypertension (caatch) trial: baseline demographic, clinical, psychosocial, and behavioral characteristics. *Implement Sci*. 2011;6 CC-H:100. doi:10.1186/1748-5908-6-100

779. Fernandez-Ruiz J, Montero-Vilchez T, Buendia-Eisman A, Arias-Santiago S. Knowledge, Behaviour and Attitudes Related to Sun Exposure in Sportspeople: A Systematic Review. *Int J Environ Res Public Health*. 2022;19(16). doi:10.3390/ijerph191610175

780. Fernstrom JD, Wolfe RR. Introduction to symposium on branched-chain amino acids in exercise. *J Nutr*. 2006;136(2):524S. doi:10.1093/jn/136.2.524S

781. Ferrando AA, Tipton KD, Bamman MM, Wolfe RR. Resistance exercise maintains skeletal muscle protein synthesis during bed rest. *J Appl Physiol*. 1997;82(3):807-810. doi:10.1152/jappl.1997.82.3.807

782. Field CJ, Johnson I, Pratt VC. Glutamine and arginine: immunonutrients for improved health. *Med Sci Sports Exerc*. 2000;32(7 Suppl):S377-88. doi:10.1097/00005768-200007001-00002

783. Fields AI, Cuerdon TT, Brasseux CO, et al. Physician burnout in pediatric critical care medicine. *Crit Care Med*. 1995;23(8):1425-1429. doi:10.1097/00003246-199508000-00018

784. Filiberti R, Giacosa A, Brignoli O. High-risk subjects for vitamin deficiency. *Eur J cancer Prev Off J Eur Cancer Prev Organ*. 1997;6 Suppl 1:S37-42. doi:10.1097/00008469-199703001-00008

785. Finnerty CC, Capek KD, Voigt C, et al. The P50 Research Center in Perioperative Sciences: How the investment by the National Institute of General Medical Sciences in team science has reduced postburn mortality. *J Trauma Acute Care Surg*. 2017;83(3):532-542. doi:10.1097/TA.0000000000001644

786. Firestone R, Faeamani G, Okiakama E, et al. Pasifika Prediabetes Youth Empowerment Programme: learnings from a youth-led community-based intervention study. *N Z Med J*. 2021;134(1530):57-68.

787. FISCHER M, CRAMER MN, HUANG MU, et al. Burn Injury Does Not Exacerbate Heat Strain during Exercise while Wearing Body Armor. *Med Sci Sport Exerc*. 2020;52(10):2235-2241. doi:10.1249/MSS.0000000000002375

788. Fisher M, Burns J, Symons H, et al. Treatment of eating disorders in a division of adolescent medicine. *Int J Adolesc Med Heal*. 2002;14(4):283-295. https://search.ebscohost.com/login.aspx?direct=true&AuthType=cookie,ip,shib,uid&db=cul&AN=105822056&site=ehost-live&scope=site

789. Fitzpatrick R, Chambers J, Burns T, et al. A systematic review of outcome measures used in forensic mental health research with consensus panel opinion. *Health Technol Assess (Rockv)*. 2010;14(55):1-94. doi:10.3310/hta14180

790. Fleg JL, Huang Z, Reynolds HR, et al. Ischemia Severity, Coronary Artery Disease Extent, and Exercise Capacity in ISCHEMIA. *Circulation*. 2024;150(2):165-167. doi:10.1161/CIRCULATIONAHA.123.066980

791. Fletcher JR, Esau SP, Holash RJ, MacIntosh BR. Feasiblity of the two-hour marathon is a burning issue. *J Appl Physiol*. 2011;110(1):282; discussion 294. doi:10.1152/japplphysiol.01259.2010

792. Flores O, Tyack Z, Stockton K, Paratz JD. The use of exercise in burns rehabilitation: A worldwide survey of practice. *Burns*. 2020;46(2):322-332. doi:10.1016/j.burns.2019.02.016

793. Flores O, Tyack Z, Stockton K, Ware R, Paratz JD. Exercise training for improving outcomes post-burns: a systematic review and meta-analysis. *Clin Rehabil*. 2018;32(6):734-746. doi:10.1177/0269215517751586

794. Flores O, Tyack Z, Stockton K, Ware R, Paratz JD. Exercise training for improving outcomes post-burns: a systematic review and meta-analysis. *Clin Rehabil*. 2018;32(6):734-746. doi:10.1177/0269215517751586

795. Flynn KE, Lin L, Ellis SJ, et al. Outcomes, health policy, and managed care: relationships between patient-reported outcome measures and clinical measures in outpatients with heart failure. *Am Heart J*. 2009;158(4 Suppl):S64-71. doi:10.1016/j.ahj.2009.07.010

796. Flynn KE, Piña IL, Whellan DJ, et al. Effects of exercise training on health status in patients with chronic heart failure: HF-ACTION randomized controlled trial. *JAMA*. 2009;301(14):1451-1459. doi:10.1001/jama.2009.457

797. FN W, MG J, DL C, et al. Modulation of the hypermetabolic response to trauma: temperature, nutrition, and drugs. *J Am Coll Surg*. 2009;208(4):489-502. doi:10.1016/j.jamcollsurg.2009.01.022

798. Foncerrada G, Capek KD, Wurzer P, et al. Functional Exercise Capacity in Children With Electrical Burns. *J Burn care Res Off Publ Am Burn Assoc*. 2017;38(3):e647-e652. doi:10.1097/BCR.0000000000000443

799. Foncerrada G, Capek KD, Wurzer P, et al. Functional Exercise Capacity in Children With Electrical Burns. *J Burn Care Res*. 2017;38(3):e647-e652. doi:10.1097/BCR.0000000000000443

800. Ford KJ, Burns RJ. The role of positive psychological wellbeing in walking speed differences among married and unmarried English older adults. *Aging Ment Health*. 2024;28(4):684-691. doi:10.1080/13607863.2023.2268032

801. Forman DE, Clare R, Kitzman DW, et al. Relationship of age and exercise performance in patients with heart failure: the HF-ACTION study. *Am Heart J*. 2009;158(4 Suppl):S6-S15. doi:10.1016/j.ahj.2009.07.018

802. Forster A, Lambley R, Hardy J, et al. Rehabilitation for older people in long-term care. *Cochrane database Syst Rev*. 2009;(1):CD004294. doi:10.1002/14651858.CD004294.pub2

803. Foster J, Watso J, Crandall CG. Evidence for Chronotropic Incompetence in Well-healed Burn Survivors. *J Burn CARE Res*. 2023;44(2):431-437. doi:10.1093/jbcr/irac056

804. Foster N, Kornhaber R, McGarry S, Wood FM, Edgar DW. Heterotopic Ossification in adults following a burn: A phenomenological analysis. *Burns*. 2017;43(6):1250-1262. doi:10.1016/j.burns.2017.03.001

805. Fram RY, Cree MG, Chinkes DL, Herndon DN, Wolfe RR. Recovery of labeled CO2 from acetate in severely burned children. *Am J Physiol Endocrinol Metab*. 2007;293(6):E1726-9. doi:10.1152/ajpendo.00388.2007

806. Francescato MP, Geat M, Accardo A, Blokar M, Cattin L, Noacco C. Exercise and glycemic imbalances: a situation-specific estimate of glucose supplement. *Med Sci Sports Exerc*. 2011;43(1):2-11. doi:10.1249/MSS.0b013e3181e6d6a1

807. Franco JA, Leví P de LÁ. Feelings, Stress, and Adaptation Strategies of Nurses against COVID-19 in Guayaquil. *Investig y Educ en Enferm*. 2020;38(3). doi:10.17533/udea.iee.v38n3e07

808. Frank LD, Greenwald MJ, Winkelman S, Chapman J, Kavage S. Carbonless footprints: promoting health and climate stabilization through active transportation. *Prev Med (Baltim)*. 2010;50 Suppl 1:S99-105. doi:10.1016/j.ypmed.2009.09.025

809. Frederix I, Dendale P, Berger J, Vandereyt F, Everts S, Hansen D. Comparison of two motion sensors for use in cardiac telerehabilitation. *J Telemed Telecare*. 2011;17(5):231-234. doi:10.1258/jtt.2010.100914

810. Friedrich JB, Muzaffar AR, Hanel DP. Pediatric hand friction burns from treadmill contact. *Hand (N Y)*. 2007;2(4):188-193. doi:10.1007/s11552-007-9046-2

811. From LJ, Bergen LG, Humlie CJ. The effects of open leaf burning on spirometric measurements in asthma. *Chest*. 1992;101(5):1236-1239. doi:10.1378/chest.101.5.1236

812. Fu J, Li F, Tang Y, et al. The Emerging Role of Irisin in Cardiovascular Diseases. *J Am Heart Assoc*. 2021;10(20):e022453. doi:10.1161/JAHA.121.022453

813. Fu L, Burns RD, Xie Y, Lucero JE, Brusseau TA, Bai Y. Associations of an Online Health Coaching Intervention with Movement Behaviors and Perceived Health: A Mediation Analysis. *J Heal Eat Act living*. 2024;4(1):19-31.

814. Fu L, Burns RD, Zhe S, Bai Y. What explains adolescents’ physical activity and sports participation during the COVID-19 pandemic? - an interpretable machine learning approach. *J Sports Sci*. 2024;42(17):1651-1663. doi:10.1080/02640414.2024.2404783

815. Fu Y, Burns RD, Brusseau TA, Zhang P, Constantino N. Influence of meeting weekday and weekend step count recommendations on weight statusin children. *J Sports Sci*. 2021;39(7):808-814. doi:10.1080/02640414.2020.1847489

816. Fu Y, Burns RD, Constantino N, Zhang P. Differences in Step Counts, Motor Competence, and Enjoyment Between an Exergaming Group and a Non-Exergaming Group. *Games Health J*. 2018;7(5):335-340. doi:10.1089/g4h.2017.0188

817. Fu Y, Burns RD, Hsu YW, Zhang P. Motivation, Segmented Physical Activity, Sedentary Behavior, and Weight Status in Adolescents: A Path Analysis. *Res Q Exerc Sport*. 2022;93(1):204-209. doi:10.1080/02701367.2020.1804520

818. Fu Y, Brusseau TA, Hannon JC, Burns RD. Effect of a 12-Week Summer Break on School Day Physical Activity and Health-Related Fitness in Low-Income Children from CSPAP Schools. *J Environ Public Heal*. Published online March 9, 2017:1-7. doi:10.1155/2017/9760817

819. Fu Y, Burns RD. Effect of an Active Video Gaming Classroom Curriculum on Health-Related Fitness, School Day Step Counts, and Motivation in Sixth Graders. *J Phys Act Health*. 2018;15(9):644-650. doi:10.1123/jpah.2017-0481

820. Fu Y, Burns RD, Brusseau TA, Hannon JC. Comprehensive School Physical Activity Programming and Activity Enjoyment. *Am J Health Behav*. 2016;40(4):496-502. doi:10.5993/AJHB.40.4.11

821. Fu Y, Burns RD, Gomes E, Savignac A, Constantino N. Trends in Sedentary Behavior, Physical Activity, and Motivation during a Classroom-Based Active Video Game Program. *Int J Environ Res Public Health*. 2019;16(16). doi:10.3390/ijerph16162821

822. Fu Y, Gao Z, Hannon JC, Burns RD, Brusseau TAJ. Effect of the SPARK Program on Physical Activity, Cardiorespiratory Endurance, and Motivation in Middle-School Students. *J Phys Act Health*. 2016;13(5):534-542. doi:10.1123/jpah.2015-0351

823. Fukushima Y, Kurose S, Shinno H, et al. Relationships between serum irisin levels and metabolic parameters in Japanese patients with obesity. *Obes Sci Pract*. 2016;2(2):203-209. doi:10.1002/osp4.43

824. Fuller NR, Burns J, Sainsbury A, et al. Examining the association between depression and obesity during a weight management programme. *Clin Obes*. 2017;7(6):354-359. doi:10.1111/cob.12208

825. Galipeau N, Sugarman SL, Waller E. Local Dose Coefficients for Radionuclide Contamination in Wounds. *Health Phys*. 2023;125(3):159-174. doi:10.1097/HP.0000000000001704

826. Gammanpila UP, Burns A, Heller RF, Purandare N. What are the benefits of cognitive enhancers for Alzheimer’s Disease: use of Population Impact Measures. *BMC Geriatr*. 2007;7:25. doi:10.1186/1471-2318-7-25

827. Ganio MS, Pearson J, Schlader ZJ, et al. Aerobic Fitness Is Disproportionately Low in Adult Burn Survivors Years After Injury. *J Burn Care Res*. 2015;36(4):513-519. doi:10.1097/BCR.0b013e3182a22915

828. GANIO MS, SCHLADER ZJ, PEARSON J, et al. Nongrafted Skin Area Best Predicts Exercise Core Temperature Responses in Burned Humans. *Med Sci Sport Exerc*. 2015;47(10):2224-2232. doi:10.1249/MSS.0000000000000655

829. García-Malinis AJ, Gracia-Cazaña T, Zazo M, et al. Sun Protection Behaviors and Knowledge in Mountain Marathon Runners and Risk Factors for Sunburn. *Actas Dermosifiliogr*. 2021;112(2):159-166. doi:10.1016/j.ad.2020.11.003

830. Gardin JM, Leifer ES, Fleg JL, et al. Relationship of Doppler-Echocardiographic left ventricular diastolic function to exercise performance in systolic heart failure: the HF-ACTION study. *Am Heart J*. 2009;158(4 Suppl):S45-52. doi:10.1016/j.ahj.2009.07.015

831. Garrido-Ardila EM, Santos-Domínguez M, Rodríguez-Mansilla J, et al. A Systematic Review of the Effectiveness of Virtual Reality-Based Interventions on Pain and Range of Joint Movement Associated with Burn Injuries. *J Pers Med*. 2022;12(8). doi:10.3390/jpm12081269

832. Garside T, Wood FM, Vallence AM. Case series investigating the cortical silent period after burns using transcranial magnetic stimulation. *BURNS*. 2018;44(5):1195-1202. doi:10.1016/j.burns.2018.04.010

833. Gauffin E, Öster C. Patient perception of long-term burn-specific health and congruence with the Burn Specific Health Scale-Brief. *Burn*. 2019;45(7):N.PAG-N.PAG. doi:10.1016/j.burns.2018.12.015

834. Gaur A, Sinclair M, Caruso E, Peretti G, Zaleske D. Heterotopic ossification around the elbow following burns in children: results after excision. *J Bone Joint Surg Am*. 2003;85(8):1538-1543. doi:10.2106/00004623-200308000-00016

835. Gaztelu Valdés V, Gago Fornells M, Garcia González RF, Guijón del Castillo AJ, Morales Gutiérrez A, Torra i Bou JE. Hot sand burns on the sole of a patient with diabetes. *J Wound Care*. 2002;11(5):170-171. doi:10.12968/jowc.2002.11.5.26401

836. Geller AC, Glanz K, Shigaki D, Isnec MR, Sun T, Maddock J. Impact of skin cancer prevention on outdoor aquatics staff: the Pool Cool program in Hawaii and Massachusetts. *Prev Med (Baltim)*. 2001;33(3):155-161. doi:10.1006/pmed.2001.0870

837. Gellman R, Burns S. Walking aches and running pains. Injuries of the foot and ankle. *Prim Care*. 1996;23(2):263-280. doi:10.1016/s0095-4543(05)70275-x

838. Gelwick R. The Patient Self Determination Act and “Dax’s case”. *J Med Humanit*. 1992;13(3):177-187. doi:10.1007/BF01127376

839. Gerhart JI, Sanchez Varela V, Burns JW. Brief Training on Patient Anger Increases Oncology Providers’ Self-Efficacy in Communicating With Angry Patients. *J Pain Symptom Manag*. 2017;54(3):355-360.e2. doi:10.1016/j.jpainsymman.2017.07.039

840. Gerhart J, Ramos K, Porter LS, et al. Top Ten Tips Palliative Care Clinicians Should Know About Behavioral Pain Management for Persistent Pain. *J Palliat Med*. 2023;26(7):992-998. doi:10.1089/jpm.2022.0571

841. Gerhartl F, Strang A, Coster A. [Rehabilitation of patients with burns]. *Tijdschr Voor Ziekenverpl*. 1984;37(12):384-387.

842. Gersh BJ, Sliwa K, Mayosi BM, Yusuf S. Novel therapeutic concepts: the epidemic of cardiovascular disease in the developing world: global implications. *Eur Heart J*. 2010;31(6):642-648. doi:10.1093/eurheartj/ehq030

843. Gesik NY, Tan SKG, Prentiss GT, Fitzsimmons S, Nichols AW. The use of pregame hyperhydration with intravenous fluids in National Collegiate Athletic Association Football Bowl Subdivision teams. *Clin J Sport Med Off J Can Acad Sport Med*. 2013;23(6):488-490. doi:10.1097/JSM.0b013e31828563b2

844. Ghio AJ, Soukup JM, Case M, et al. Exposure to wood smoke particles produces inflammation in healthy volunteers. *Occup Environ Med*. 2012;69(3):170-175. doi:10.1136/oem.2011.065276

845. Giacomazzi S, Urits I, Hoyt B, et al. Comprehensive Review and Update of Burning Eye Syndrome. *J patient-centered Res Rev*. 2021;8(3):255-260. doi:10.17294/2330-0698.1813

846. Gibas MK, Gibas KJ. Induced and controlled dietary ketosis as a regulator of obesity and metabolic syndrome pathologies. *Diabetes Metab Syndr*. 2017;11 Suppl 1:S385-S390. doi:10.1016/j.dsx.2017.03.022

847. Gieg LM, Duncan KE, Suflita JM. Bioenergy production via microbial conversion of residual oil to natural gas. *Appl Environ Microbiol*. 2008;74(10):3022-3029. doi:10.1128/AEM.00119-08

848. Gies P, Glanz K, O’Riordan D, Elliott T, Nehl E. Measured occupational solar UVR exposures of lifeguards in pool settings. *Am J Ind Med*. 2009;52(8):645-653. doi:10.1002/ajim.20722

849. Giesbrecht GG, Walpoth BH. Risk of Burns During Active External Rewarming for Accidental Hypothermia. *WILDERNESS Environ Med*. 2019;30(4):431-436. doi:10.1016/j.wem.2019.06.005

850. Gil F, Pla A, Hernández AF, Mercado JM, Méndez F. A fatal case following exposure to zinc chloride and hexachloroethane from a smoke bomb in a fire simulation at a school. *Clin Toxicol (Phila)*. 2008;46(6):563-565. doi:10.1080/15563650701610890

851. Gilchrist JD, Morris KL, Dwyer LA, Conroy DE. Patterns of sun safety behaviors in parents: Associations with physical activity, sedentary behavior, and access to neighborhood physical activity resources. *Prev Med (Baltim)*. 2020;132:105976. doi:10.1016/j.ypmed.2019.105976

852. Giménez-Egido JM, Ortega E, Verdu-Conesa I, Cejudo A, Torres-Luque G. Using Smart Sensors to Monitor Physical Activity and Technical-Tactical Actions in Junior Tennis Players. *Int J Environ Res Public Health*. 2020;17(3). doi:10.3390/ijerph17031068

853. Gittings PM, Wand BM, Hince DA, Grisbrook TL, Wood FM, Edgar DW. The efficacy of resistance training in addition to usual care for adults with acute burn injury: A randomised controlled trial. *BURNS*. 2021;47(1):84-100. doi:10.1016/j.burns.2020.03.015

854. Gittings PM, Wand BM, Hince DA, Grisbrook TL, Wood FM, Edgar DW. The efficacy of resistance training in addition to usual care for adults with acute burn injury: A randomised controlled trial. *Burns*. 2021;47(1):84-100. doi:10.1016/j.burns.2020.03.015

855. Gittings PM, Grisbrook TL, Edgar DW, Wood FM, Wand BM, Connell NEO. ScienceDirect Resistance training for rehabilitation after burn injury : A systematic literature review &. *Burns*. 2017;44(4):731-751. doi:10.1016/j.burns.2017.08.009

856. Gittings PM, Grisbrook TL, Edgar DW, Wood FM, Wand BM, O’Connell NE. Corrigendum to “Resistance training for rehabilitation after burn injury: A systematic literature review & meta-analysis” [Burns 44 (2018) 731-751]. *Burns*. 2020;46(5):1240-1241. doi:10.1016/j.burns.2020.02.012

857. Gittings PM, Grisbrook TL, Edgar DW, Wood FM, Wand BM, O’Connell NE. Resistance training for rehabilitation after burn injury: A systematic literature review & meta-analysis. *Burns*. 2018;44(4):731-751. doi:10.1016/j.burns.2017.08.009

858. Glaser J, Hansson E, Weiss I, et al. Preventing kidney injury among sugarcane workers: promising evidence from enhanced workplace interventions. *Occup Environ Med*. 2020;77(8):527-534. doi:10.1136/oemed-2020-106406

859. Glusman M, Coromilas J, Clark WC, et al. Pain sensitivity in silent myocardial ischemia. *Pain*. 1996;64(3):477-483. doi:10.1016/0304-3959(95)00142-5

860. Godleski M, Oeffling A, Bruflat AK, Craig E, Weitzenkamp D, Lindberg G. Treating burn-associated joint contracture: results of an inpatient rehabilitation stretching protocol. *J Burn care Res Off Publ Am Burn Assoc*. 2013;34(4):420-426. doi:10.1097/BCR.0b013e3182700178

861. Gohel S, Baldwin KD, Hill JF. CLOSED REDUCTION OF PEDIATRIC DISTAL RADIAL FRACTURES AND EPIPHYSEAL SEPARATIONS. *JBJS Essent Surg Tech*. 2020;10(4). doi:10.2106/JBJS.ST.19.00059

862. Goldberg CS, Gaynor JW, Mahle WT, et al. The pediatric heart network’s study on long-term outcomes of children with HLHS and the impact of Norwood Shunt type in the single ventricle reconstruction trial cohort (SVRIII): Design and adaptations. *Am Heart J*. 2022;254:216-227. doi:10.1016/j.ahj.2022.09.005

863. Goldberg CS, Trachtenberg F, Gaynor JW, et al. Longitudinal Follow-Up of Children With HLHS and Association Between Norwood Shunt Type and Long-Term Outcomes: The SVR III Study. *Circulation*. 2023;148(17):1330-1339. doi:10.1161/CIRCULATIONAHA.123.065192

864. Golden KJ, Diana RM. A Case of Brachioradial Pruritus Treated with Chiropractic and Acupuncture. *Case Rep Dermatol*. 2022;14(1):93-97. doi:10.1159/000524054

865. Golshan M, Faghihi M, Roushan-Zamir T, et al. Early effects of burning rice farm residues on respiratory symptoms of villagers in suburbs of Isfahan, Iran. *Int J Environ Health Res*. 2002;12(2):125-131. doi:10.1080/09603120220129283

866. Gomez A, Sharma AK, Mallott EK, et al. Plasticity in the Human Gut Microbiome Defies Evolutionary Constraints. *MSPHERE*. 2019;4(4). doi:10.1128/mSphere.00271-19

867. Gomez A, Sharma AK, Mallott EK, et al. Erratum for Gomez et al., “Plasticity in the Human Gut Microbiome Defies Evolutionary Constraints”. *mSphere*. 2021;6(2). doi:10.1128/mSphere.00168-21

868. Gomez J, Hoffman HG, Bistricky SL, et al. The Use of Virtual Reality Facilitates Dialectical Behavior Therapy® “Observing Sounds and Visuals” Mindfulness Skills Training Exercises for a Latino Patient with Severe Burns: A Case Study. *Front Psychol*. 2017;8:1611. doi:10.3389/fpsyg.2017.01611

869. Gong D, Lei J, He X, et al. Keys to the switch of fat burning: stimuli that trigger the uncoupling protein 1 (UCP1) activation in adipose tissue. *Lipids Health Dis*. 2024;23(1):322. doi:10.1186/s12944-024-02300-z

870. Goodwin CW, Maguire MS, McManus WF, Pruitt BAJ. Prospective study of burn wound excision of the hands. *J Trauma*. 1983;23(6):510-517. doi:10.1097/00005373-198306000-00012

871. Gordon G, Rhoads A. Field-deployable measurements of free-living individuals to determine energy balance: fuel substrate usage through *δ*<SUP>13</SUP>C in breath CO_2_ and diet through hair *δ*<SUP>13</SUP>C and *δ*<SUP>15</SUP>N values. *Isotopes Environ Health Stud*. 2019;55(1):70-79. doi:10.1080/10256016.2018.1562448

872. Gordon KR, Burns P, Keller G. Experimental changes in mineral content of juvenile mouse femora. *Calcif Tissue Int*. 1992;51(3):229-232. doi:10.1007/BF00334552

873. Görig T, Apfelbacher C, Drewitz KP, Reimers AK, Breitbart EW, Diehl K. Sunburn and sun protection during recreational outdoor sport in summer: Findings from the German general population aged 16-65 years. *Photodermatol Photoimmunol Photomed*. 2023;39(6):589-597. doi:10.1111/phpp.12898

874. Goudeau S, Sanrey C, Stanczak A, Manstead A, Darnon C. Why lockdown and distance learning during the COVID-19 pandemic are likely to increase the social class achievement gap. *Nat Hum Behav*. 2021;5(10):1273-1281. doi:10.1038/s41562-021-01212-7

875. Goyal J, Iyer S, Palande C, Brahmankar U, John J, Patil K. Comparative assessment of the efficacy of an intralesional injection of placentrex, hyaluronidase and dexamethasone in the management of oral submucous fibrosis: A randomized controlled trial. *Med Int*. 2024;4(2):19. doi:10.3892/mi.2024.143

876. Goyal M, Aggarwal A, Goyal K, Garg P. Effectiveness of Osteopathic Therapy in the Treatment of Oral Submucous Fibrosis. *Contemp Clin Dent*. 2017;8(1):145-147. doi:10.4103/ccd.ccd_999_16

877. Graham ZA, Lavin KM, O’Bryan SM, et al. Mechanisms of exercise as a preventative measure to muscle wasting. *Am J Physiol Cell Physiol*. 2021;321(1):C40-C57. doi:10.1152/ajpcell.00056.2021

878. Graif Y, Teplitski V. [Exercise induced vasculitis]. *Harefuah*. 2013;152(7):389-390,434,435.

879. Gras LZ, Kanaan SF, McDowd JM, Colgrove YM, Burns J, Pohl PS. Balance and gait of adults with very mild Alzheimer disease. *J Geriatr Phys Ther*. 2015;38(1):1-7. doi:10.1519/JPT.0000000000000020

880. Graven-Nielsen T, Jansson Y, Segerdahl M, et al. Experimental pain by ischaemic contractions compared with pain by intramuscular infusions of adenosine and hypertonic saline. *Eur J Pain*. 2003;7(1):93-102. doi:10.1016/s1090-3801(02)00069-1

881. Gray K, Pacey V, Gibbons P, Little D, Burns J. Interventions for congenital talipes equinovarus (clubfoot). *Cochrane database Syst Rev*. 2014;2014(8):CD008602. doi:10.1002/14651858.CD008602.pub3

882. Gray K, Pacey V, Gibbons P, Little D, Frost C, Burns J. Interventions for congenital talipes equinovarus (clubfoot). *Cochrane database Syst Rev*. 2012;(4):CD008602. doi:10.1002/14651858.CD008602.pub2

883. Grealy R, Herruer J, Smith CLE, Hiller D, Haseler LJ, Griffiths LR. Evaluation of a 7-Gene Genetic Profile for Athletic Endurance Phenotype in Ironman Championship Triathletes. *PLoS One*. 2015;10(12):e0145171. doi:10.1371/journal.pone.0145171

884. Green JT, Chess AC, Burns M, Schachinger KM, Thanellou A. The effects of two forms of physical activity on eyeblink classical conditioning. *Behav Brain Res*. 2011;219(1):165-174. doi:10.1016/j.bbr.2011.01.016

885. Green JMH, Cranston GR, Sutherland WJ, et al. Research priorities for managing the impacts and dependencies of business upon food, energy, water and the environment. *Sustain Sci*. 2017;12(2):319-331. doi:10.1007/s11625-016-0402-4

886. Green ZD, John CS, Kueck PJ, et al. Rationale and methods to characterize the acute exercise response in aging and Alzheimer’s Disease: the AEROBIC pilot study. *Contemp Clin Trials*. 2021;107. doi:10.1016/j.cct.2021.106457

887. Green ZD, John CS, Kueck PJ, et al. Acute exercise alters brain glucose metabolism in aging and Alzheimer’s disease. *J Physiol*. Published online September 2024. doi:10.1113/JP286923

888. Greene BR, Foran TG, McGrath D, Doheny EP, Burns A, Caulfield B. A comparison of algorithms for body-worn sensor-based spatiotemporal gait parameters to the GAITRite electronic walkway. *J Appl Biomech*. 2012;28(3):349-355. doi:10.1123/jab.28.3.349

889. Greer M, Dimick S, Burns S. Heart rate and blood pressure response to several methods of strength training. *Phys Ther*. 1984;64(2):179-183. doi:10.1093/ptj/64.2.179

890. Greviskes LE, Podlog L, Burns RD, et al. Caring Rehabilitation Climate, the Tripartite Efficacy Framework, and Adherence to Rehabilitation Programs Among Individuals With Parkinson’s Disease: A Multiple Mediation Analysis. *J Geriatr Phys Ther*. 2020;43(3):E16-E24. doi:10.1519/JPT.0000000000000211

891. Greviskes LE, Podlog L, Newton M, et al. Caring Interactions in Secondary Prevention Programs: A Qualitative Inquiry of Individuals With Parkinson’s Disease. *J Geriatr Phys Ther*. 2019;42(3):167-175. doi:10.1519/JPT.0000000000000151

892. Grigor’ev MG, Atiasov NI, Dmitriev GI, Razvozova EP. [Restoration of the function of the upper extremities in deformities following burns]. *Ortop Travmatol Protez*. 1971;32(8):15-19.

893. Grimaldi PA. Regulatory role of peroxisome proliferator-activated receptor delta (PPAR delta) in muscle metabolism. A new target for metabolic syndrome treatment? *Biochimie*. 2005;87(1):5-8. doi:10.1016/j.biochi.2004.11.009

894. Gripp CL, Salvaggio J, Fratianne RB, CL G, Salvaggio J, RB F. PT/OT forum. Use of burn intensive care unit gymnasium as an adjunct to therapy. *J Burn Care Rehabil*. 1995;16(2 part 1):160-161. doi:10.1097/00004630-199503000-00014

895. Grisbrook TL, Elliott CM, Edgar DW, Wallman KE, Wood FM, Reid SL. Burn-injured adults with long term functional impairments demonstrate the same response to resistance training as uninjured controls. *Burn*. 2013;39(4):680-686. doi:10.1016/j.burns.2012.09.005

896. Grisbrook TL, Reid SL, Edgar DW, Wallman KE, Wood FM, Elliott CM. Exercise training to improve health related quality of life in long term survivors of major burn injury: a matched controlled study. *Burns*. 2012;38(8):1165-1173. doi:10.1016/j.burns.2012.03.007

897. Grisbrook TL, Reid SL, Elliott CM, Elliott BC, Edgar DW, Wood FM. Lower limb functional outcome assessment following burn injury: a novel use for 3D laboratory-based movement analysis. *Burns*. 2010;36(3):e24-30. doi:10.1016/j.burns.2009.01.006

898. Grisbrook TL, Stearne SM, Reid SL, Wood FM, Rea SM, Elliott CM. Demonstration of the use of the ICF framework in detailing complex functional deficits after major burn. *Burns*. 2012;38(1):32-43. doi:10.1016/j.burns.2011.04.001

899. Grisbrook TL, Wallman KE, Elliott CM, Wood FM, Edgar DW, Reid SL. The effect of exercise training on pulmonary function and aerobic capacity in adults with burn. *Burns*. 2012;38(4):607-613. doi:10.1016/j.burns.2011.11.004

900. Grisbrook TL, Gittings PM, Wood FM, Edgar DW. The effectiveness of session rating of perceived exertion to monitor resistance training load in acute burns patients. *Burns*. 2017;43(1):169-175. doi:10.1016/j.burns.2016.07.021

901. Grisbrook TL, Kenworthy P, Phillips M, Wood FM, Edgar DW. Nanocrystalline silver dressings significantly influence bioimpedance spectroscopy measurements of fluid volumes in burns patients. *Burns*. 2016;42(7):1548-1555. doi:10.1016/j.burns.2016.04.008

902. Gross KN, Allen LE, Hagele AM, et al. A Dose-Response Study to Examine Paraxanthine’s Impact on Energy Expenditure, Hunger, Appetite, and Lipolysis. *J Diet Suppl*. 2024;21(5):608-632. doi:10.1080/19390211.2024.2351222

903. Gross MT, Tyson AD, Burns CB, MT G, AD T, CB B. Effect of knee angle and ligament insufficiency on anterior tibial translation during quadriceps muscle contraction: a preliminary report. *J Orthop Sport Phys Ther*. 1993;17(3):133-143. doi:10.2519/jospt.1993.17.3.133

904. Grün F, Blumberg B. Minireview: the case for obesogens. *Mol Endocrinol*. 2009;23(8):1127-1134. doi:10.1210/me.2008-0485

905. Guan Y, Ma D. Fibrodysplasia ossificans progressiva complicated with post traumatic and infectious myositis ossificans in masseter: A case report. *Medicine (Baltimore)*. 2024;103(37):e39648. doi:10.1097/MD.0000000000039648

906. Guardino CM, Hobel CJ, Shalowitz MU, Ramey SL, Dunkel Schetter C. Psychosocial and demographic predictors of postpartum physical activity. *J Behav Med*. 2018;41(5):668-679. doi:10.1007/s10865-018-9931-x

907. Guillermau A. [Kinesitherapy and burn patients]. *Soins*. 1979;24(20):35-39.

908. Gujral S, Burns M, Erickson KI, et al. Dose-response effects of exercise on mental health in community-dwelling older adults: Exploration of genetic moderators. *Int J Clin Health Psychol*. 2024;24(1):100443. doi:10.1016/j.ijchp.2024.100443

909. Guntur VP, Nemkov T, de Boer E, et al. Signatures of Mitochondrial Dysfunction and Impaired Fatty Acid Metabolism in Plasma of Patients with Post-Acute Sequelae of COVID-19 (PASC). *Metabolites*. 2022;12(11). doi:10.3390/metabo12111026

910. Guo L, Huang Y, He J, et al. Associations of lifestyle characteristics with circulating immune markers in the general population based on NHANES 1999 to 2014. *Sci Rep*. 2024;14(1):13444. doi:10.1038/s41598-024-63875-2

911. Guo ZR, Li F, Tu HX, et al. [Initiation, development, and achievements of burn rehabilitation therapy in China]. *Zhonghua Shao Shang Za Zhi*. 2018;34(12):835-839. doi:10.3760/cma.j.issn.1009-2587.2018.12.002

912. Gupta K, Mehrotra M, Kumar P, Gogia AR, Prasad A, Fisher JA. Smoke Inhalation Injury: Etiopathogenesis, Diagnosis, and Management. *Indian J Crit care Med peer-reviewed, Off Publ Indian Soc Crit Care Med*. 2018;22(3):180-188. doi:10.4103/ijccm.IJCCM_460_17

913. Gupta S, Piyush P, Mahajan A, Mohanty S, Ghosh S, Singh K. Fibrotomy with diode laser (980nm) and habit correlation in oral submucous fibrosis: a report of 30 cases. *LASERS Med Sci*. 2018;33(8):1739-1745. doi:10.1007/s10103-018-2531-8

914. Guzick SS. Skin care: burn survivor case study. Part 2. *Dermatology Nurs*. 1993;5(3):209-212,236.

915. Gwynne Jones DP, Theis JC. Acute compartment syndrome due to closed muscle rupture. *Aust N Z J Surg*. 1997;67(4):227-228. doi:10.1111/j.1445-2197.1997.tb01950.x

916. Habal MB. The burned hand: a planned treatment program. *J Trauma*. 1978;18(8):587-595.

917. Hackam DG, Khan NA, Hemmelgarn BR, et al. The 2010 Canadian Hypertension Education Program recommendations for the management of hypertension: part 2 - therapy. *Can J Cardiol*. 2010;26(5):249-258. doi:10.1016/s0828-282x(10)70379-2

918. Hackam DG, Quinn RR, Ravani P, et al. The 2013 Canadian Hypertension Education Program recommendations for blood pressure measurement, diagnosis, assessment of risk, prevention, and treatment of hypertension. *Can J Cardiol*. 2013;29(5):528-542. doi:10.1016/j.cjca.2013.01.005

919. Hackett D, Roberts-Clarke D, Jain N, et al. Body composition and its association with physical performance, quality of life, and clinical indictors in Charcot-Marie-Tooth disease: a pilot study. *Disabil Rehabil*. 2019;41(4):405-412. doi:10.1080/09638288.2017.1395083

920. Hafen BB, Burns B. Physiology, Smooth Muscle. In: ; 2024.

921. Hafen BB, Shook M, Burns B. Anatomy, Smooth Muscle. In: ; 2024.

922. Hagedorn JM, Canzanello N, Lamer TJ. Dorsal Root Ganglion Stimulation for Erythromelalgia Related Foot Pain: A Case Report and Review of the Literature. *Pain Pract*. 2021;21(6):698-702. doi:10.1111/papr.12998

923. Hägele FA, Büsing F, Nas A, et al. Appetite Control Is Improved by Acute Increases in Energy Turnover at Different Levels of Energy Balance. *J Clin Endocrinol Metab*. 2019;104(10):4481‐4491. doi:10.1210/jc.2019-01164

924. Hahn T, Paplham P, Austin-Ketch T, et al. Ascertainment of Unmet Needs and Participation in Health Maintenance and Screening of Adult Hematopoietic Cell Transplantation Survivors Followed in a Formal Survivorship Program. *Biol blood marrow Transplant J Am Soc Blood Marrow Transplant*. 2017;23(11):1968-1973. doi:10.1016/j.bbmt.2017.07.024

925. Hall DM, McCarty F, Elliott T, Glanz K. Lifeguards’ sun protection habits and sunburns: association with sun-safe environments and skin cancer prevention program participation. *Arch Dermatol*. 2009;145(2):139-144. doi:10.1001/archdermatol.2008.553

926. Hall WF, Salisbury RE. Physical therapy for burns of the upper extremity. *Major Probl Clin Surg*. 1976;19:116-126.

927. Hallward L, Duncan LR. “Compulsive exercise is a socially acceptable prison cell”: Exploring experiences with compulsive exercise across social media. *Int J Eat Disord*. 2021;54(9):1663-1671. doi:10.1002/eat.23577

928. Halsey LG. The Mystery of Energy Compensation. *Physiol Biochem Zool*. 2021;94(6):380-393. doi:10.1086/716467

929. Halsey LG, Watkins DAR, Duggan BM. The energy expenditure of stair climbing one step and two steps at a time: estimations from measures of heart rate. *PLoS One*. 2012;7(12):e51213. doi:10.1371/journal.pone.0051213

930. Hambraeus A, Ransjö U. Attempts to control clothes-borne infection in a burn unit. I. Experimental investigations of some clothes for barrier nursing. *J Hyg (Lond)*. 1977;79(2):193-202. doi:10.1017/s0022172400052992

931. Hamilton J. Physiotherapy in the treatment of leprosy. *Prog Phys Ther*. 1970;1(4):307-311.

932. Hamilton M, Tomlinson G, Chu L, et al. Determinants of Depressive Symptoms at 1 Year Following ICU Discharge in Survivors of ≥ 7 Days of Mechanical Ventilation: Results From the RECOVER Program, a Secondary Analysis of a Prospective Multicenter Cohort Study. *Chest*. 2019;156(3):466-476. doi:10.1016/j.chest.2019.04.104

933. Han CM, Wang XG. [Interpretation of International Society for Burn Injury practice guidelines for burn care published in 2018]. *Zhonghua Shao Shang Za Zhi*. 2021;37(2):196-200. doi:10.3760/cma.j.cn501120-20191129-00447

934. Han C, Rush AM, Dib-Hajj SD, et al. Sporadic onset of erythermalgia: a gain-of-function mutation in Nav1.7. *Ann Neurol*. 2006;59(3):553-558. doi:10.1002/ana.20776

935. Han D, Hyun MC, Miller RJH, et al. 10-year experience of utilizing a stress-first SPECT myocardial perfusion imaging. *Int J Cardiol*. 2024;401:131863. doi:10.1016/j.ijcard.2024.131863

936. Han F, Hu D, Liu Y, et al. [Repair of skin and soft tissue defects around the knee joints combined with patellar ligament defects using free anterolateral thigh flaps with iliotibial tracts]. *Zhonghua Shao Shang Za Zhi*. 2015;31(5):327-330.

937. Han Y, John GF, Clement TP. Understanding the thermal degradation patterns of hopane biomarker compounds present in crude oil. *Sci Total Environ*. 2019;667:792-798. doi:10.1016/j.scitotenv.2019.02.445

938. Hanley B, Bissas A, Merlino S, Burns GT. Changes in running biomechanics during the 2017 IAAF world championships men’s 1500 m final. *Scand J Med Sci Sports*. 2023;33(6):931-942. doi:10.1111/sms.14331

939. Hanna L, Burns C, O’Neill C, Coughlan E. A Systematic Review of the Implementation and Effectiveness of “The Daily Mile” on Markers of Children’s Health. *Int J Environ Res Public Health*. 2023;20(13). doi:10.3390/ijerph20136203

940. Hannabass K, Olsen KR. Fat burn X: burning more than fat. *BMJ Case Rep*. 2016;2016. doi:10.1136/bcr-2015-213374

941. Hansson E, Glaser J, Weiss I, et al. Workload and cross-harvest kidney injury in a Nicaraguan sugarcane worker cohort. *Occup Environ Med*. 2019;76(11):818-826. doi:10.1136/oemed-2019-105986

942. Hansson E, Jakobsson K, Glaser J, et al. Impact of heat and a rest-shade-hydration intervention program on productivity of piece-paid industrial agricultural workers at risk of chronic kidney disease of nontraditional origin. *Ann Work Expo Heal*. 2024;68(4):366-375. doi:10.1093/annweh/wxae007

943. Hardee JP, Porter C, Sidossis LS, et al. Early rehabilitative exercise training in the recovery from pediatric burn. *Med Sci Sports Exerc*. 2014;46(9):1710-1716. doi:10.1249/MSS.0000000000000296

944. Hardwicke J. The influence of outcomes on the provision and practice of burn care. *Burns*. 2016;42(2):307-315. doi:10.1016/j.burns.2015.07.002

945. Harkin A, Connor TJ, Burns MP, Kelly JP. Nitric oxide synthase inhibitors augment the effects of serotonin re-uptake inhibitors in the forced swimming test. *Eur Neuropsychopharmacol J Eur Coll Neuropsychopharmacol*. 2004;14(4):274-281. doi:10.1016/j.euroneuro.2003.08.010

946. Harper SA, Peters FJ, Pollock BS, Burns K, McDaniel J, Ridgel AL. Design of an eccentric recumbent ergometer to elicit delayed onset muscle soreness. *Res Directs Heal Sci*. 2021;1(1):3.

947. Harré N, Coveney A. School-based scalds prevention: reaching children and their families. *Health Educ Res*. 2000;15(2 CC-Wounds):191‐202. doi:10.1093/her/15.2.191

948. Harrer JU, Uçeyler N, Doppler K, et al. Neuropathic pain in two-generation twins carrying the sodium channel Nav1.7 functional variant R1150W. *Pain*. 2014;155(10):2199-2203. doi:10.1016/j.pain.2014.08.003

949. Harrington RA, Arena R, Després JP, Ciarochi A, Croll E, Bloch KD. More than 10 million steps in the right direction: results from the first American Heart Association scientific sessions walking challenge. *Prog Cardiovasc Dis*. 2015;57(4):296-298. doi:10.1016/j.pcad.2014.09.009

950. Harris MB, Kuo CH. Scientific Challenges on Theory of Fat Burning by Exercise. *Front Physiol*. 2021;12. doi:10.3389/fphys.2021.685166

951. Harrison H, Burns M, Darko N, Jones C. Exploring the benefits of nature-based interventions in socio-economically deprived communities: a narrative review of the evidence to date. *Perspect Public Health*. 2023;143(3):156-172. doi:10.1177/17579139231170768

952. Harrison LK, Carroll D, Burns VE, et al. Cardiovascular and secretory immunoglobulin A reactions to humorous, exciting, and didactic film presentations. *Biol Psychol*. 2000;52(2):113-126. doi:10.1016/s0301-0511(99)00033-2

953. Harrison LK, Denning S, Easton HL, et al. The effects of competition and competitiveness on cardiovascular activity. *Psychophysiology*. 2001;38(4):601-606.

954. Hartigan C, JA P, SC W, et al. An overview of muscle strengthening. *J Burn Care Rehabil*. 1989;10(3):251-257. https://search.ebscohost.com/login.aspx?direct=true&AuthType=cookie,ip,shib,uid&db=cul&AN=107535106&site=ehost-live&scope=site

955. Hartung GH, Blancq RJ, Lally DA, Krock LP. Estimation of aerobic capacity from submaximal cycle ergometry in women. *Med Sci Sports Exerc*. 1995;27(3):452-457.

956. Hartung GH, Kohl HW, Blair SN, Lawrence SJ, Harrist RB. Exercise tolerance and alcohol intake. Blood pressure relation. *Hypertens (Dallas, Tex 1979)*. 1990;16(5):501-507. doi:10.1161/01.hyp.16.5.501

957. Hartung GH, Myhre LG, Tucker DM, Burns JW. Hormone and energy substrate changes during prolonged exercise in the heat. *Aviat Space Environ Med*. 1987;58(1):24-28.

958. Harvey DR, McGauran AMT, Murphy J, Burns L, McMonagle E, Commins S. Emergence of an egocentric cue guiding and allocentric inferring strategy that mirrors hippocampal brain-derived neurotrophic factor (BDNF) expression in the Morris water maze. *Neurobiol Learn Mem*. 2008;89(4):462-479. doi:10.1016/j.nlm.2007.08.013

959. Hasan R, Bhatt D, Khan S, et al. Association of Her-2 Expression and Clinicopathological Parameters in Colorectal Carcinoma in Indian Population. *Open access Maced J Med Sci*. 2019;7(1):6-11. doi:10.3889/oamjms.2019.008

960. Hasegawa K, Namba Y, Kimata Y. Negative pressure wound therapy incorporating early exercise therapy in hand surgery: bag-type negative pressure wound therapy. *Acta Med Okayama*. 2013;67(4):271-276. doi:10.18926/AMO/51073

961. Hatton AL, Chatfield MD, Gane EM, et al. The effects of wearing textured versus smooth shoe insoles for 4-weeks in people with diabetic peripheral neuropathy: a randomised controlled trial. *Disabil Rehabil*. Published online May 2024:1-11. doi:10.1080/09638288.2024.2360658

962. Hatunic M, Finucane F, Burns N, Gasparro D, Nolan JJ. Vascular inflammatory markers in early-onset obese and type 2 diabetes subjects before and after three months’ aerobic exercise training. *Diabetes Vasc Dis Res*. 2007;4(3):231-234. doi:10.3132/dvdr.2007.045

963. Hausdorf K, Eakin E, Whiteman D, Rogers C, Aitken J, Newman B. Prevalence and correlates of multiple cancer risk behaviors in an Australian population-based survey: results from the Queensland Cancer Risk Study. *Cancer Causes Control*. 2008;19(10):1339-1347. doi:10.1007/s10552-008-9205-y

964. Haverkamp FJC, Giesbrecht GG, Tan ECTH. The prehospital management of hypothermia - An up-to-date overview. *Injury*. 2018;49(2):149-164. doi:10.1016/j.injury.2017.11.001

965. Hawke F, Chuter V, Burns J. Factors associated with night-time calf muscle cramps: A case-control study. *Muscle Nerve*. 2013;47(3):339-343. doi:10.1002/mus.23531

966. Hawke F, McKay MJ, Baldwin JN, et al. Correlates of night‐time and exercise‐associated lower limb cramps in healthy adults. *Muscle Nerve*. 2021;64(3):301-308. doi:10.1002/mus.27359

967. Hawke F, Sadler SG, Katzberg HD, Pourkazemi F, Chuter V, Burns J. Non-drug therapies for the secondary prevention of lower limb muscle cramps. *Cochrane database Syst Rev*. 2021;5(5):CD008496. doi:10.1002/14651858.CD008496.pub3

968. Hawkins LK, Burns L, Swancutt D, Moghadam S, Pinkney J, Tarrant M. Which components of behavioral weight management programs are essential for weight loss in people living with obesity? A rapid review of systematic reviews. *Obes Rev an Off J Int Assoc Study Obes*. 2024;25(10):e13798. doi:10.1111/obr.13798

969. Hawley JA. Fat burning during exercise: can ergogenics change the balance? *Phys Sportsmed*. 1998;26(9):56-68. doi:10.3810/psm.1998.09.1143

970. Haynes BWJ. Outpatient burns. *Clin Plast Surg*. 1974;1(4):645-651.

971. He S, Li L, Hu J, Chen Q, Shu W. Effectiveness of Traditional Chinese Medicine (TCM) treatments on the cognitive functioning of elderly persons with mild cognitive impairment associated with white matter lesions. *Shanghai Arch psychiatry*. 2015;27(5):289-295. doi:10.11919/j.issn.1002-0829.215109

972. Heckman CJ, Auerbach M V, Darlow S, Handorf EA, Raivitch S, Manne SL. Association of Skin Cancer Risk and Protective Behaviors with Health Literacy Among Young Adults in the USA. *Int J Behav Med*. 2019;26(4):372-379. doi:10.1007/s12529-019-09788-1

973. Heels-Ansdell D, Kelly L, O’Grady HK, et al. Early In-Bed Cycle Ergometry With Critically Ill, Mechanically Ventilated Patients: Statistical Analysis Plan for CYCLE (Critical Care Cycling to Improve Lower Extremity Strength), an International, Multicenter, Randomized Clinical Trial. *JMIR Res Protoc*. 2024;13:e54451. doi:10.2196/54451

974. Hegel MT, Ayllon T, VanderPlate C, Spiro-Hawkins H. A behavioral procedure for increasing compliance with self-exercise regimens in severely burn-injured patients. *Behav Res Ther*. 1986;24(5):521-528. doi:10.1016/0005-7967(86)90032-x

975. Heggie TW, Heggie TM. The epidemiology of extreme hiking injuries in volcanic environments. *Med Sport Sci*. 2012;58:130-141. doi:10.1159/000338721

976. Helal B, Chapman R, Ellis M, Gifford D. The use of silicone oil for mobilisation of the hand. *J Bone Joint Surg Br*. 1982;64(1):67-69. doi:10.1302/0301-620X.64B1.7068722

977. Helle C, Sommer AK, Syversen PV, Lauritzen F. Doping substances in dietary supplements. *Tidsskr den Nor laegeforening Tidsskr Prakt Med ny raekke*. 2019;139(4). doi:10.4045/tidsskr.18.0502

978. Helm PA, Kevorkian CG, Lushbaugh M, Pullium G, Head MD, Cromes GF. Burn injury: rehabilitation management in 1982. *Arch Phys Med Rehabil*. 1982;63(1):6-16.

979. Helm P, Herndon DN, Delateur B. Restoration of function. *J Burn care Res Off Publ Am Burn Assoc*. 2007;28(4):611-614. doi:10.1097/BCR.0B013E318093E4CA

980. Hemington-Gorse SJ, Potokar TS, Drew PJ, Dickson WA. Burn care costing: the Welsh experience. *Burns*. 2009;35(3):378-382. doi:10.1016/j.burns.2008.08.012

981. Heng JS, Clancy O, Atkins J, et al. Revised Baux Score and updated Charlson comorbidity index are independently associated with mortality in burns intensive care patients. *Burns*. 2015;41(7):1420-1427. doi:10.1016/j.burns.2015.06.009

982. Hernández-Alvarez MI, Thabit H, Burns N, et al. Subjects with early-onset type 2 diabetes show defective activation of the skeletal muscle PGC-1{alpha}/Mitofusin-2 regulatory pathway in response to physical activity. *Diabetes Care*. 2010;33(3):645-651. doi:10.2337/dc09-1305

983. Herndon DN. Nutritional and pharmacological support of the metabolic response to injury. *Minerva Anestesiol*. 2003;69(4):264-274.

984. Herndon DN, Tompkins RG. Support of the metabolic response to burn injury. *Lancet (London, England)*. 2004;363(9424):1895-1902. doi:10.1016/S0140-6736(04)16360-5

985. Herold R, Gevorgyan H, Damerau LS, et al. Effects of Smart Glasses on the Visual Acuity and Eye Strain of Employees in Logistics and Picking: A Six-Month Observational Study. *Sensors (Basel)*. 2024;24(20). doi:10.3390/s24206515

986. Herpin D, Amiel A, Boutaud P, Ciber MA, Demange J. [Evaluation of the antihypertensive effect and tolerability of a new delayed-action calcium channel blocker: nitrendipine, prescribed as a single daily dose of 20 mg]. *Ann Cardiol Angeiol (Paris)*. 1986;35(9):561-566.

987. Herrmann NP, Bennett JL. The differentiation of traumatic and heat-related fractures in burned bone. *J Forensic Sci*. 1999;44(3):461-469.

988. Herrmann SD, Martin LE, Breslin FJ, et al. Neuroimaging studies of factors related to exercise: rationale and design of a 9 month trial. *Contemp Clin Trials*. 2014;37(1):58-68. doi:10.1016/j.cct.2013.11.007

989. Hesseln H, Loomis JB, González-Cabán A, Alexander S. Wildfire effects on hiking and biking demand in New Mexico: a travel cost study. *J Environ Manage*. 2003;69(4):359-368. doi:10.1016/j.jenvman.2003.09.012

990. Heyer GR, Hornstein OP. Recent studies of cutaneous nociception in atopic and non-atopic subjects. *J Dermatol*. 1999;26(2):77-86. doi:10.1111/j.1346-8138.1999.tb03516.x

991. HG H, DR P, Seibel E, et al. Virtual reality pain control during burn wound debridement in the hydrotank. *Clin J Pain*. 2008;24(4):299-304. doi:10.1097/ajp.0b013e318164d2cc

992. Hicks JH. Swimming and the skin. *Cutis*. 1977;19(4):448-450.

993. Hildebrandt T, Harty S, Langenbucher JW. Fitness supplements as a gateway substance for anabolic-androgenic steroid use. *Psychol Addict Behav J Soc Psychol Addict Behav*. 2012;26(4):955-962. doi:10.1037/a0027877

994. Hinds TDJ, Burns KA, Hosick PA, et al. Biliverdin Reductase A Attenuates Hepatic Steatosis by Inhibition of Glycogen Synthase Kinase (GSK) 3β Phosphorylation of Serine 73 of Peroxisome Proliferator-activated Receptor (PPAR) α. *J Biol Chem*. 2016;291(48):25179-25191. doi:10.1074/jbc.M116.731703

995. Hinds TDJ, Hosick PA, Chen S, et al. Mice with hyperbilirubinemia due to Gilbert’s syndrome polymorphism are resistant to hepatic steatosis by decreased serine 73 phosphorylation of PPARα. *Am J Physiol Endocrinol Metab*. 2017;312(4):E244-E252. doi:10.1152/ajpendo.00396.2016

996. Hinwood AL, Trout M, Murby J, Barton C, Symons B. Assessing urinary levoglucosan and methoxyphenols as biomarkers for use in woodsmoke exposure studies. *Sci Total Environ*. 2008;402(1):139-146. doi:10.1016/j.scitotenv.2008.04.012

997. Hjellestad M. [Physical therapy of extensive burns]. *Tidsskr den Nor laegeforening Tidsskr Prakt Med ny raekke*. 1989;109(31):3200-3202.

998. Hochstatter KR, Hull SJ, Sethi AK, Burns ME, Mundt MP, Westergaard RP. Promoting safe injection practices, substance use reduction, hepatitis c testing, and overdose prevention among syringe service program clients using a computer-tailored intervention: Pilot randomized controlled trial. *J Med Internet Res*. 2020;22(9). doi:10.2196/19703

999. Hocking P, Broadhurst M, Nixon RDV, Gannoni A. Validation of the Psychosocial Assessment Tool 2.0 for paediatric burn patients. *Burns*. 2023;49(7):1632-1642. doi:10.1016/j.burns.2023.05.002

1000. Hodges DL, McGuire TJ. Burning and pain after injury. Is it causalgia or reflex sympathetic dystrophy? *Postgrad Med*. 1988;83(2):185-192. doi:10.1080/00325481.1988.11700147

1001. Hodgson CL, Stiller K, Needham DM, et al. Expert consensus and recommendations on safety criteria for active mobilization of mechanically ventilated critically ill adults. *Crit Care*. 2014;18(6):658. doi:10.1186/s13054-014-0658-y

1002. Hoffer MM, Brody G, Ferlic F. Excision of heterotopic ossification about elbows in patients with thermal injury. *J Trauma*. 1978;18(9):667-670. doi:10.1097/00005373-197809000-00009

1003. Hoffman HG, Patterson DR, Carrougher GJ. Use of virtual reality for adjunctive treatment of adult burn pain during physical therapy: a controlled study. *Clin J Pain*. 2000;16(3 CC-SR-REHAB CC-Pain, Palliative and Supportive Care):244‐250. doi:10.1097/00002508-200009000-00010

1004. Hoffman HG, Patterson DR, Carrougher GJ, Sharar SR. Effectiveness of virtual reality-based pain control with multiple treatments. *Clin J Pain*. 2001;17(3 CC-Pain, Palliative and Supportive Care):229‐235. doi:10.1097/00002508-200109000-00007

1005. Holavanahalli RK, Helm PA, Kowalske KJ, Hynan LS. Effectiveness of Paraffin and Sustained Stretch in Treatment of Shoulder Contractures Following a Burn Injury. *Arch Phys Med Rehabil*. 2020;101(1S):S42-S49. doi:10.1016/j.apmr.2019.08.482

1006. Holavanahalli RK, Helm PA, Parry IS, Dolezal CA, Greenhalgh DG. Select practices in management and rehabilitation of burns: a survey report. *J Burn care Res Off Publ Am Burn Assoc*. 2011;32(2):210-223. doi:10.1097/BCR.0b013e31820aadd5

1007. Holden MR, Watson MC, Clifford MJ, Beard MR. Parents’ perceptions of unintentional paediatric burn injuries - A qualitative study. *Burn*. 2020;46(5):1179-1192. doi:10.1016/j.burns.2019.12.009

1008. Holman DM, Ding H, Guy GPJ, Watson M, Hartman AM, Perna FM. Prevalence of Sun Protection Use and Sunburn and Association of Demographic and Behaviorial Characteristics With Sunburn Among US Adults. *JAMA dermatology*. 2018;154(5):561-568. doi:10.1001/jamadermatol.2018.0028

1009. Homayounzade M. Positive input observer-based controller design for blood glucose regulation for type 1 diabetic patients: A backstepping approach. *IET Syst Biol*. 2022;16(5):157-172. doi:10.1049/syb2.12049

1010. Honari S, Caceres M, Romo M, Gibran NS, Gamelli RL. The Role of a Burn Research Coordinator: A Guide for Novice Coordinators. *J Burn Care Res*. 2016;37(2):127-134. doi:10.1097/BCR.0000000000000264

1011. Honea RA, John CS, Green ZD, et al. Relationship of fasting glucose and longitudinal Alzheimer’s disease imaging markers. *ALZHEIMERS DEMENTIA-TRANSLATIONAL Res Clin Interv*. 2022;8(1). doi:10.1002/trc2.12239

1012. Hong RA, Rivera KK, Jittirat A, Choi JJ. Flecainide suppresses defibrillator-induced storming in catecholaminergic polymorphic ventricular tachycardia. *Pacing Clin Electrophysiol*. 2012;35(7):794-797. doi:10.1111/j.1540-8159.2012.03421.x

1013. Hopkins M, Andrews R, Salem V, et al. Improving understanding of type 2 diabetes remission: research recommendations from Diabetes UK’s 2019 remission workshop. *Diabet Med*. 2020;37(11):1944-1950. doi:10.1111/dme.14358

1014. Horton JW, White DJ. Diminished cardiac contractile response to burn injury in aged guinea pigs. *J Trauma*. 1993;34(3):429-436. doi:10.1097/00005373-199303000-00021

1015. Horwich TB, Leifer ES, Brawner CA, Fitz-Gerald MB, Fonarow GC. The relationship between body mass index and cardiopulmonary exercise testing in chronic systolic heart failure. *Am Heart J*. 2009;158(4 Suppl):S31-6. doi:10.1016/j.ahj.2009.07.016

1016. Houmard JA, Costill DL, Mitchell JB, Park SH, Fink WJ, Burns JM. Testosterone, cortisol, and creatine kinase levels in male distance runners during reduced training. *Int J Sports Med*. 1990;11(1):41-45. doi:10.1055/s-2007-1024760

1017. Hourani L, Tueller S, Kizakevich P, et al. Toward Preventing Post-Traumatic Stress Disorder: Development and Testing of a Pilot Predeployment Stress Inoculation Training Program. *Mil Med*. 2016;181(9):1151-1160. doi:10.7205/MILMED-D-15-00192

1018. Howell JW. Management of the acutely burned hand for the nonspecialized clinician. *Phys Ther*. 1989;69(12):1077-1090. doi:10.1093/ptj/69.12.1077

1019. Howells ME, Mayfour KLW, April TB, Bender RL, Loudon JE. Is there a difference in student physical activity between a field school and a traditional classroom setting? *Am J Hum Biol*. 2022;34(12). doi:10.1002/ajhb.23799

1020. Hoyos AE, Perez ME, Domínguez-Millán R. Variable Sculpting in Dynamic Definition Body Contouring: Procedure Selection and Management Algorithm. *AESTHETIC Surg J*. 2021;41(3):318-332. doi:10.1093/asj/sjaa133

1021. Hristina K, Langerholc T, Trapecar M. Novel metabolic roles of L-arginine in body energy metabolism and possible clinical applications. *J Nutr Health Aging*. 2014;18(2):213-218. doi:10.1007/s12603-014-0015-5

1022. Huan J ning. [Advances in the research of fluid resuscitation for burn shock]. *Zhonghua Shao Shang Za Zhi*. 2013;29(3):285-288.

1023. Huang KL, Chien PS, Kishi Y, Lin YC. Application of conservation principle in estimating body volume in rats. *J Appl Physiol*. 1994;76(1):391-396. doi:10.1152/jappl.1994.76.1.391

1024. Huang KL, Chen CW, Chu SJ, Perng WC, Wu CP. Systemic inflammation caused by white smoke inhalation in a combat exercise. *Chest*. 2008;133(3):722-728. doi:10.1378/chest.07-2076

1025. Huang M, Moralez G, Romero SA, et al. The benefits of an unsupervised exercise program in persons with well-healed burn injuries within the International Classification of Functioning, Disability and Health (ICF). *BURNS*. 2020;46(6):1280-1288. doi:10.1016/j.burns.2020.06.023

1026. Huang SH, Yang SM, Lo JJ, Wu SH, Tai MH. Irisin Gene Delivery Ameliorates Burn-Induced Sensory and Motor Neuropathy. *Int J Mol Sci*. 2020;21(20). doi:10.3390/ijms21207798

1027. Huber FX, Eckstein HH, Allenberg JR. [Bilateral tibial compartment syndrome with crush symptoms after cannabis abuse]. *Chirurg*. 1997;68(8):829-831. doi:10.1007/s001040050280

1028. Hull JH, Burns P, Carre J, et al. BTS clinical statement for the assessment and management of respiratory problems in athletic individuals. *Thorax*. 2022;77(6):540-551. doi:10.1136/thoraxjnl-2021-217904

1029. Hull KL, Abell L, Adenwalla SF, et al. Impact of physical activity on surrogate markers of cardiovascular disease in the haemodialysis population. *Clin Kidney J*. 2024;17(7):sfae198. doi:10.1093/ckj/sfae198

1030. Hume E, Armstrong M, Manifield J, et al. Impact of COVID-19 shielding on physical activity and quality of life in patients with COPD. *BREATHE*. 2020;16(3). doi:10.1183/20734735.0231-2020

1031. Hundeshagen G, Herndon DN, Clayton RP, et al. Long-term effect of critical illness after severe paediatric burn injury on cardiac function in adolescent survivors: an observational study. *Lancet Child Adolesc Heal*. 2017;1(4):293-301. doi:10.1016/S2352-4642(17)30122-0

1032. Hundeshagen G, Suman OE, Branski LK. Rehabilitation in the Acute Versus Outpatient Setting. *Clin Plast Surg*. 2017;44(4):729-735. doi:10.1016/j.cps.2017.05.004

1033. Hunt PAF, Smith JE. Self assessment exercises in emergency medicine. Question 5. Head and neck burns with evidence of airway involvement. *J R Army Med Corps*. 2005;151(3):192-193,196-198.

1034. Hunter AL, Unosson J, Bosson JA, et al. Effect of wood smoke exposure on vascular function and thrombus formation in healthy fire fighters. *Part Fibre Toxicol*. 2014;11:62. doi:10.1186/s12989-014-0062-4

1035. Hunter DJ, Eyles J, Murphy NJ, et al. Multi-centre randomised controlled trial comparing arthroscopic hip surgery to physiotherapist-led care for femoroacetabular impingement (FAI) syndrome on hip cartilage metabolism: the Australian FASHIoN trial. *BMC Musculoskelet Disord*. 2021;22(1):697. doi:10.1186/s12891-021-04576-z

1036. Huo T, Ruan JJ, Jiang MJ, et al. [Prospective study on the effects of resistance training with elastic band at home on muscle function and walking ability of severely burned children]. *Zhonghua shao shang yu chuang mian xiu fu za zhi*. 2023;39(12):1131-1139. doi:10.3760/cma.j.cn501225-20230729-00022

1037. Huo T, Xu XY, Xie WG, Liu SH. [Research advances on the application of rehabilitation exercise training in pediatric burn rehabilitation]. *Zhonghua Shao Shang Za Zhi*. 2023;39(3):275-279. doi:10.3760/cma.j.cn501225-20220116-00008

1038. Hur GY, Rhee BJ, Ko JH, et al. Correction of postburn equinus deformity. *Ann Plast Surg*. 2013;70(3):276-279. doi:10.1097/SAP.0b013e31827a6c83

1039. Hurren JS. Can blood taken from intraosseous cannulations be used for blood analysis? *Burns*. 2000;26(8):727-730. doi:10.1016/s0305-4179(00)00034-6

1040. Hurst JR, Abbas SH, Bintalib HM, et al. Granulomatous-lymphocytic interstitial lung disease: an international research prioritisation. *ERJ open Res*. 2021;7(4). doi:10.1183/23120541.00467-2021

1041. Hurwitz EL. Commentary: Exercise and spinal manipulative therapy for chronic low back pain: time to call for a moratorium on future randomized trials? *Spine J*. 2011;11(7):599-600. doi:10.1016/j.spinee.2011.04.021

1042. Hurwitz EL, Carragee EJ, van der Velde G, et al. Treatment of neck pain: noninvasive interventions: results of the Bone and Joint Decade 2000-2010 Task Force on Neck Pain and Its Associated Disorders. *J Manipulative Physiol Ther*. 2009;32(2 Suppl):S141-75. doi:10.1016/j.jmpt.2008.11.017

1043. Hurwitz EL, Carragee EJ, van der Velde G, et al. Treatment of neck pain: noninvasive interventions: results of the Bone and Joint Decade 2000-2010 Task Force on Neck Pain and Its Associated Disorders. *Spine (Phila Pa 1976)*. 2008;33(4 Suppl):S123-52. doi:10.1097/BRS.0b013e3181644b1d

1044. Huynh DP, Figueroa K, Hoang N, Pulst SM. Nuclear localization or inclusion body formation of ataxin-2 are not necessary for SCA2 pathogenesis in mouse or human. *Nat Genet*. 2000;26(1):44-50. doi:10.1038/79162

1045. Hylden C, Burns T, Stinner D, Owens J. Blood flow restriction rehabilitation for extremity weakness: a case series. *J Spec Oper Med a peer Rev J SOF Med Prof*. 2015;15(1):50-56.

1046. Imbus SH, Zawacki BE. Autonomy for burned patients when survival is unprecedented. *N Engl J Med*. 1977;297(6):308-311. doi:10.1056/NEJM197708112970605

1047. Inagaki M, Ohno K, Hisatome I, Tanaka Y, Takeshita K. Relative hypoxia of the extremities in Fabry disease. *Brain Dev*. 1992;14(5):328-333. doi:10.1016/s0387-7604(12)80153-7

1048. Inagaki M, Ohno K, Ohta S, Sakuraba H, Takeshita K. Relief of chronic burning pain in Fabry disease with neurotropin. *Pediatr Neurol*. 1990;6(3):211-213. doi:10.1016/0887-8994(90)90067-b

1049. Incognito A V, Doherty CJ, Lee JB, Burns MJ, Millar PJ. Interindividual variability in muscle sympathetic responses to static handgrip in young men: evidence for sympathetic responder types? *Am J Physiol Integr Comp Physiol*. 2018;314(1):R114-R121. doi:10.1152/ajpregu.00266.2017

1050. Incognito A V, Doherty CJ, Lee JB, Burns MJ, Millar PJ. Ischemic preconditioning does not alter muscle sympathetic responses to static handgrip and metaboreflex activation in young healthy men. *Physiol Rep*. 2017;5(14). doi:10.14814/phy2.13342

1051. Ingen-Housz-Oro S, Schmidt V, Ameri MM, et al. Post-acute phase and sequelae management of epidermal necrolysis: an international, multidisciplinary DELPHI-based consensus. *Orphanet J Rare Dis*. 2023;18(1). doi:10.1186/s13023-023-02631-7

1052. Inoue N, Matsunaga Y, Satoh H, Takahashi M. Enhanced energy expenditure and fat oxidation in humans with high BMI scores by the ingestion of novel and non-pungent capsaicin analogues (capsinoids). *Biosci Biotechnol Biochem*. 2007;71(2):380-389. doi:10.1271/bbb.60341

1053. Ippen H. [Letter: Chemical burns while bathing. A differential diagnostic report]. *Dtsch Med Wochenschr*. 1973;98(43):2048-2049.

1054. Ivanova E, Burns RJ, Deschênes SS, Knäuper B, Schmitz N. A Longitudinal Investigation of Anxiety and Depressive Symptomatology and Exercise Behaviour Among Adults With Type 2 Diabetes Mellitus. *Can J Diabetes*. 2017;41(1):73-81. doi:10.1016/j.jcjd.2016.07.006

1055. Izumi AK, Moore RE. Seaweed (Lyngbya majuscula) dermatitis. *Clin Dermatol*. 1987;5(3):92-100. doi:10.1016/s0738-081x(87)80014-7

1056. Jacka B, Larance B, Copeland J, et al. Health care engagement behaviors of men who use performance- and image-enhancing drugs in Australia. *Subst Abus*. 2020;41(1):139-145. doi:10.1080/08897077.2019.1635954

1057. Jackson C, Stewart ID, Plekhanova T, et al. Effects of sleep disturbance on dyspnoea and impaired lung function following hospital admission due to COVID-19 in the UK: a prospective multicentre cohort study. *Lancet Respir Med*. 2023;11(8):673-684. doi:10.1016/S2213-2600(23)00124-8

1058. JACKSON DM. The treatment of burns: an exercise in emergency surgery. *Ann R Coll Surg Engl*. 1953;13(4):236-257.

1059. Jacobs KA, Burns P, Kressler J, Nash MS. Heavy reliance on carbohydrate across a wide range of exercise intensities during voluntary arm ergometry in persons with paraplegia. *J Spinal Cord Med*. 2013;36(5):427-435. doi:10.1179/2045772313Y.0000000123

1060. Jacobs LM, Burns KJ. Terrorism preparedness: Web-based resource management and the TOPOFF 3 exercise. *J Trauma*. 2006;60(3):566-572. doi:10.1097/01.ta.0000197379.40878.c7

1061. Jacobs PL, Burns P. Acute enhancement of lower-extremity dynamic strength and flexibility with whole-body vibration. *J strength Cond Res*. 2009;23(1):51-57. doi:10.1519/JSC.0b013e3181839f19

1062. Jacobson K, Fletchall S, Dodd H, Starnes C. Current Concepts Burn Rehabilitation, Part I: Care During Hospitalization. *Clin Plast Surg*. 2017;44(4):703-712. doi:10.1016/j.cps.2017.05.003

1063. Jaeger DL. Maintenance of function of the burn patient. *Phys Ther*. 1972;52(6):627-633. doi:10.1093/ptj/52.6.627

1064. Jaeschke L, Steinbrecher A, Luzak A, et al. Socio-cultural determinants of physical activity across the life course: a “Determinants of Diet and Physical Activity” (DEDIPAC) umbrella systematic literature review. *Int J Behav Nutr Phys Act*. 2017;14(1):173. doi:10.1186/s12966-017-0627-3

1065. Jäger M, Lindhardt MC, Pedersen JR, et al. Putting the pieces together: A qualitative study exploring perspectives on self-management and exercise behavior among people living with multimorbidity, healthcare professionals, relatives, and patient advocates. *J multimorbidity comorbidity*. 2022;12:26335565221100172. doi:10.1177/26335565221100172

1066. Jagnoor J, Lukaszyk C, Fraser S, et al. Rehabilitation practices for burn survivors in low and middle income countries: A literature review. *Burns*. 2018;44(5):1052-1064. doi:10.1016/j.burns.2017.10.007

1067. Jahangiri FR, Sheryar M, Al Behairy Y. Early detection of pedicle screw-related spinal cord injury by continuous intraoperative neurophysiological monitoring (IONM). *Neurodiagn J*. 2014;54(4):323-337. doi:10.1080/21646821.2014.11106817

1068. Jaishree, Bhowmik S. Letter to the Editor on: “Optimization of pulmonary function, functional capacity, and quality of life in adolescents with thoracic burns after a 2-month arm cycling exercise programme: a randomized controlled study.” *Burns*. 2022;48(5):1268‐1269. doi:10.1016/j.burns.2022.04.018

1069. James DL, Jowza M. Principles of Burn Pain Management. *Clin Plast Surg*. 2017;44(4):737-747. doi:10.1016/j.cps.2017.05.005

1070. Janz KF, Burns TL, Witt JD, Mahoney LT. Longitudinal analysis of scaling VO2 for differences in body size during puberty: the Muscatine Study. *Med Sci Sports Exerc*. 1998;30(9):1436-1444. doi:10.1097/00005768-199809000-00014

1071. Janz KF, Medema-Johnson HC, Letuchy EM, et al. Subjective and objective measures of physical activity in relationship to bone mineral content during late childhood: the Iowa Bone Development Study. *Br J Sports Med*. 2008;42(8):658-663. doi:10.1136/bjsm.2008.047779

1072. Janz KF, Boros P, Letuchy EM, Kwon S, Burns TL, Levy SM. Physical Activity, Not Sedentary Time, Predicts Dual-Energy X-ray Absorptiometry-measured Adiposity Age 5 to 19 Years. *Med Sci Sport Exerc*. 2017;49(10):2071-2077. doi:10.1249/MSS.0000000000001336

1073. Janz KF, Burns TL, Levy SM. Tracking of activity and sedentary behaviors in childhood: the Iowa Bone Development Study. *Am J Prev Med*. 2005;29(3):171-178. doi:10.1016/j.amepre.2005.06.001

1074. Janz KF, Burns TL, Levy SM, et al. Everyday activity predicts bone geometry in children: the iowa bone development study. *Med Sci Sports Exerc*. 2004;36(7):1124-1131. doi:10.1249/01.mss.0000132275.65378.9d

1075. Janz KF, Gilmore JME, Levy SM, Letuchy EM, Burns TL, Beck TJ. Physical activity and femoral neck bone strength during childhood: the Iowa Bone Development Study. *Bone*. 2007;41(2):216-222. doi:10.1016/j.bone.2007.05.001

1076. Janz KF, Letuchy EM, Burns TL, Eichenberger Gilmore JM, Torner JC, Levy SM. Objectively measured physical activity trajectories predict adolescent bone strength: Iowa Bone Development Study. *Br J Sports Med*. 2014;48(13):1032-1036. doi:10.1136/bjsports-2014-093574

1077. Janz KF, Letuchy EM, Eichenberger Gilmore JM, et al. Early physical activity provides sustained bone health benefits later in childhood. *Med Sci Sports Exerc*. 2010;42(6):1072-1078. doi:10.1249/MSS.0b013e3181c619b2

1078. Janz KF, Letuchy EM, Francis SL, Metcalf KM, Burns TL, Levy SM. Objectively measured physical activity predicts hip and spine bone mineral content in children and adolescents ages 5-15 years: iowa bone development study. *Front Endocrinol (Lausanne)*. 2014;5:112. doi:10.3389/fendo.2014.00112

1079. Janz KF, Levy SM, Burns TL, Torner JC, Willing MC, Warren JJ. Fatness, physical activity, and television viewing in children during the adiposity rebound period: the Iowa Bone Development Study. *Prev Med (Baltim)*. 2002;35(6):563-571. doi:10.1006/pmed.2002.1113

1080. Jardine A, Bright M, Knight L, Perina H, Vardon P, Harper C. Does physical activity increase the risk of unsafe sun exposure? *Heal Promot J Aust Off J Aust Assoc Heal Promot Prof*. 2012;23(1):52-57. doi:10.1071/he12052

1081. Jarrett M, McMahon M, Stiller K, Jarrett M, McMahon M, Stiller K. Physical outcomes of patients with burn injuries--a 12 month follow-up. *J Burn Care Res*. 2008;29(6):975-984. doi:10.1097/BCR.0b013e31818ba172

1082. Jawad AM, Kadhum M, Evans J, Cubitt JJ, Martin N. Recovery of functional independence following major burn: A systematic review. *Burns*. 2024;50(6):1406-1423. doi:10.1016/j.burns.2024.02.017

1083. JD P, Stockton K, Plaza A, Muller M, RJ B. Intensive exercise after thermal injury improves physical, functional, and psychological outcomes. *J Trauma Acute Care Surg*. 2012;73(1):186-194. doi:10.1097/ta.0b013e31824baa52

1084. Jean S, Godleski M. Anterior Elbow Heterotopic Ossification in Patient With Pemphigus Vulgaris: An Unheard Complication of a Rare Condition. *J Burn care Res Off Publ Am Burn Assoc*. 2023;44(1):214-217. doi:10.1093/jbcr/irac162

1085. Jedel E, Elfström ML, Hägglin C. Differences in personality, perceived stress and physical activity in women with burning mouth syndrome compared to controls. *Scand J pain*. 2021;21(1):183-190. doi:10.1515/sjpain-2020-0110

1086. Jefferson A. Preparing for a healthier older age. *J Fam Health Care*. 2006;16(1):9-11.

1087. Jekic M, Ding Y, Dzwonczyk R, Burns P, Raman S V, Simonetti OP. Magnetic field threshold for accurate electrocardiography in the MRI environment. *Magn Reson Med*. 2010;64(6):1586-1591. doi:10.1002/mrm.22419

1088. Jenkinson E, Williamson H, Byron-Daniel J, Moss TP. Systematic Review: Psychosocial Interventions for Children and Young People With Visible Differences Resulting From Appearance Altering Conditions, Injury, or Treatment Effects. *J Pediatr Psychol*. 2015;40(10):1017-1033. doi:10.1093/jpepsy/jsv048

1089. Jensen J. Nutrition power “upside-down”. *STEP perspective*. 1995:2-3.

1090. Jeong WJ, Holavanahalli RK, Kowalske KJ. Evaluation of Kinesiophobia in Survivors of Major Burn Injury. *J Burn CARE Res*. 2022;43(6):1380-1385. doi:10.1093/jbcr/irac043

1091. Jeschke MG, Herndon DN, Oscar E. Effects of exercise training on resting energy expenditure and lean mass during pediatric burn rehabilitation. *J Burn Care Res*. 2013;31(April 2006). doi:10.1097/BCR.0b013e3181db5317.Effects

1092. Jewett PI, Henning-Smith C, Lazovich D, Ahmed RL, Vogel RI. Incidental sun exposures as a source of sunburn among rural compared to urban residents in the United States. *J Rural Heal Off J Am Rural Heal Assoc Natl Rural Heal Care Assoc*. 2023;39(2):402-407. doi:10.1111/jrh.12712

1093. Jha SK, Karna B, Goodman MB. Erythromelalgia. In: ; 2024.

1094. Jiang XS, Xu L, Li L, Zhang LQ. [Investigation and analysis of the difference between the nursing needs of adult burn patients and nurses’ cognition]. *Zhonghua Shao Shang Za Zhi*. 2018;34(10):731-735. doi:10.3760/cma.j.issn.1009-2587.2018.10.016

1095. JM B, BB C, HS A, et al. Cardiorespiratory fitness and brain atrophy in early Alzheimer disease. *Neurology*. 2008;71(3):210-216. doi:10.1212/01.wnl.0000317094.86209.cb

1096. Johnson J, Silverberg R. Serial casting of the lower extremity to correct contractures during the acute phase of burn care. *Phys Ther*. 1995;75(4):262-266. doi:10.1093/ptj/75.4.262

1097. Johnson KB, Connolly CP, Cho SP, Miller TK, Sallis RE, Hiller WDB. Clinical presentation of exercise-associated hyponatremia in male and female IRONMAN<SUP>®</SUP> triathletes over three decades. *Scand J Med Sci Sports*. 2023;33(9):1841-1849. doi:10.1111/sms.14401

1098. Johnson LS, Shupp JW, Pavlovich AR, Pezzullo JC, Jeng JC, Jordan MH. Hospital length of stay--does 1% TBSA really equal 1 day? *J Burn Care Res*. 2011;32(1):13-19. doi:10.1097/BCR.0b013e318204b3ab

1099. Johnson TA, Jinnah HA, Kamatani N. Shortage of Cellular ATP as a Cause of Diseases and Strategies to Enhance ATP. *Front Pharmacol*. 2019;10. doi:10.3389/fphar.2019.00098

1100. Jones C, Burns S, Howat P, et al. Playgroups as a setting for nutrition and physical activity interventions for mothers with young children: exploratory qualitative findings. *Heal Promot J Aust*. 2010;21(2):92-98. doi:10.1071/he10092

1101. Jones C, Jancey J, Howat P, et al. Utility of stages of change construct in the planning of physical activity interventions among playgroup mothers. *BMC Res Notes*. 2013;6:300. doi:10.1186/1756-0500-6-300

1102. Jones D, Skrepnik N, Toselli RM, Leroy B. Incorporating Novel Mobile Health Technologies Into Management of Knee Osteoarthritis in Patients Treated With Intra-Articular Hyaluronic Acid: Rationale and Protocol of a Randomized Controlled Trial. *JMIR Res Protoc*. 2016;5(3):e164. doi:10.2196/resprot.5940

1103. Jones K, Hawke F, Newman J, et al. Interventions for promoting physical activity in people with neuromuscular disease. *Cochrane database Syst Rev*. 2021;5(5):CD013544. doi:10.1002/14651858.CD013544.pub2

1104. Jones NL, O’Byrne P. Respiratory medicine at McMaster University, Hamilton, Ontario: 1968 to 2013. *Can Respir J*. 2014;21(6):325. doi:10.1155/2014/860834

1105. Jones RH, Carek PJ. Management of varicose veins. *Am Fam Physician*. 2008;78(11):1289-1294.

1106. Jones RH, Velazquez EJ, Michler RE, et al. Coronary bypass surgery with or without surgical ventricular reconstruction. *N Engl J Med*. 2009;360(17):1705-1717. doi:10.1056/NEJMoa0900559

1107. Joo SY, Lee SY, Cho YS, Lee KJ, Seo CH. Effects of robot-assisted gait training in patients with burn injury on lower extremity: a single-blind, randomized controlled trial. *J Clin Med*. 2020;9(9):1‐12. doi:10.3390/jcm9092813

1108. Joo SY, Cho YS, Lee KJ, Lee SY, Seo CH. Frontal lobe oxyhemoglobin levels in patients with lower extremity burns assessed using a functional near-Infrared spectroscopy device during usual walking: a pilot study. *Comput Methods Biomech Biomed Engin*. 2021;24(2):115-121. doi:10.1080/10255842.2020.1812583

1109. Joo SY, Lee SY, Cho YS, Lee KJ, Kim SH, Seo CH. Effectiveness of robot-assisted gait training on patients with burns: a preliminary study. *Comput Methods Biomech Biomed Engin*. 2020;23(12):888-893. doi:10.1080/10255842.2020.1769080

1110. Joshi PR, Deschauer M, Zierz S. Clinically symptomatic heterozygous carnitine palmitoyltransferase II (CPT II) deficiency. *Wien Klin Wochenschr*. 2012;124(23-24):851-854. doi:10.1007/s00508-012-0296-9

1111. Jostkleigrewe F, Räder M. [Arthrolysis of the elbow joint in patients with heterotopic calcifications after burn injuries]. *Handchirurgie, Mikrochirurgie, Plast Chir Organ der Deutschsprachigen Arbeitsgemeinschaft fur Handchirurgie Organ der Deutschsprachigen Arbeitsgemeinschaft fur Mikrochirurgie der Peripher Nerven und Gefasse Organ der V.* 2002;34(5):319-323. doi:10.1055/s-2002-36304

1112. Josty IC, MacQuillan AHF, Murison MSC. Functional outcomes following surgical repair of wrist extensor tendons. *Br J Plast Surg*. 2003;56(2):120-124. doi:10.1016/s0007-1226(03)00040-7

1113. Joubert DP, Dominy TA, Burns GT. Effects of Highly Cushioned and Resilient Racing Shoes on Running Economy at Slower Running Speeds. *Int J Sports Physiol Perform*. 2023;18(2):164-170. doi:10.1123/ijspp.2022-0227

1114. Joubert DP, Oehlert GM, Jones EJ, Burns GT. Comparative Effects of Advanced Footwear Technology in Track Spikes and Road-Racing Shoes on Running Economy. *Int J Sport Physiol Perform*. 2024;19(7):705-711. doi:10.1123/ijspp.2023-0372

1115. Jouper J, Johansson M. Qigong and mindfulness-based mood recovery: exercise experiences from a single case. *J Bodyw Mov Ther*. 2013;17(1):69-76. doi:10.1016/j.jbmt.2012.06.004

1116. Jr. BJB, Branson R, SL B, et al. Emergency airway placement by EMS providers: comparison between the King LT supralaryngeal airway and endotracheal intubation. *Prehospital Disaster Med*. 2010;25(1):92-95. doi:10.1017/s1049023x00007743

1117. Jr. EWH, Burns R, SP M, et al. A randomized trial comparing aerobic exercise and resistance exercise with a health education program in older adults with knee osteoarthritis. The Fitness Arthritis and Seniors Trial (FAST). *JAMA J Am Med Assoc*. 1997;277(1):25-31. doi:10.1001/jama.277.1.25

1118. JS C, DS B, Wu J, et al. Paced respiration for vasomotor and other menopausal symptoms: a randomized, controlled trial. *JGIM J Gen Intern Med*. 2013;28(2):193-200. doi:10.1007/s11606-012-2202-6

1119. Juang D, Fike FB, Laituri CA, Mortellaro VE, St Peter SD. Treadmill injuries in the pediatric population. *J Surg Res*. 2011;170(1):139-142. doi:10.1016/j.jss.2011.02.015

1120. Julsrud ME. An unusual cause of tarsal tunnel syndrome. *J foot ankle Surg Off Publ Am Coll Foot Ankle Surg*. 1995;34(3):289-293. doi:10.1016/S1067-2516(09)80062-8

1121. Kabir S. Accidental hero. Interview by Carol Davis. *Nurs Stand*. 2006;20(33):20-21.

1122. Kabos P, Kabosova A, Neuman T. Blocking HES1 expression initiates GABAergic differentiation and induces the expression of p21(CIP1/WAF1) in human neural stem cells. *J Biol Chem*. 2002;277(11):8763-8766. doi:10.1074/jbc.C100758200

1123. Kaczynski AT, Besenyi GM, Child S, et al. Relationship of objective street quality attributes with youth physical activity: findings from the Healthy Communities Study. *Pediatr Obes*. 2018;13:7-13. doi:10.1111/ijpo.12429

1124. Kadowaki S, Miura K, Kadowaki T, et al. International Comparison of Abdominal Fat Distribution Among Four Populations: The ERA-JUMP Study. *Metab Syndr Relat Disord*. 2018;16(4):166-173. doi:10.1089/met.2017.0132

1125. Kahn SA, Patel JH, Lentz CW, Bell DE. Firefighter burn injuries: predictable patterns influenced by turnout gear. *J Burn care Res Off Publ Am Burn Assoc*. 2012;33(1):152-156. doi:10.1097/BCR.0b013e318234d8d9

1126. Kaholokula JK, Look M, Mabellos T, et al. A Cultural Dance Program Improves Hypertension Control and Cardiovascular Disease Risk in Native Hawaiians: A Randomized Controlled Trial. *Ann Behav Med*. 2021;55(10):1005-1018. doi:10.1093/abm/kaaa127

1127. Kaholokula JK, Wilson RE, Townsend CKM, et al. Translating the Diabetes Prevention Program in Native Hawaiian and Pacific Islander communities: the PILI ’Ohana Project. *Transl Behav Med*. 2014;4(2):149-159. doi:10.1007/s13142-013-0244-x

1128. Kaholokula JK, Townsend CKM, Ige A, et al. Sociodemographic, behavioral, and biological variables related to weight loss in native Hawaiians and other Pacific Islanders. *Obesity (Silver Spring)*. 2013;21(3):E196-203. doi:10.1002/oby.20038

1129. Kakitsuka EE, Morita AA, Itakussu EY, et al. Six-minute walk test in burned subjects: Applicability, reproducibility and performance at hospital discharge. *BURNS*. 2020;46(7):1540-1547. doi:10.1016/j.burns.2020.03.004

1130. Kamel NM, Toson RA, Elsayeh SM. Response of Aerobic Capacity to Low-Level Laser Therapy in Burned Patients. *J Burn care Res*. 2022;43(3):685‐690. doi:10.1093/jbcr/irab173

1131. Kaminsky DA, Guntupalli KK, Lippmann J, et al. Effect of Yoga Breathing (Pranayama) on Exercise Tolerance in Patients with Chronic Obstructive Pulmonary Disease: A Randomized, Controlled Trial. *J Altern Complement Med*. 2017;23(9):696-704. doi:10.1089/acm.2017.0102

1132. Kang HJ, Wang JCK, Burns SF, Leow MKS. Is Self-Determined Motivation a Useful Agent to Overcome Perceived Exercise Barriers in Patients With Type 2 Diabetes Mellitus? *Front Psychol*. 2021;12. doi:10.3389/fpsyg.2021.627815

1133. Kang X, Berman DS, Lewin HC, et al. Incremental prognostic value of myocardial perfusion single photon emission computed tomography in patients with diabetes mellitus. *Am Heart J*. 1999;138(6 Pt 1):1025-1032. doi:10.1016/s0002-8703(99)70066-9

1134. Kang X, Berman DS, Lewin HC, et al. Comparative localization of myocardial ischemia by exercise electrocardiography and myocardial perfusion SPECT. *J Nucl Cardiol Off Publ Am Soc Nucl Cardiol*. 2000;7(2):140-145. doi:10.1016/s1071-3581(00)90034-5

1135. Kang X, Shaw LJ, Hayes SW, et al. Impact of body mass index on cardiac mortality in patients with known or suspected coronary artery disease undergoing myocardial perfusion single-photon emission computed tomography. *J Am Coll Cardiol*. 2006;47(7):1418-1426. doi:10.1016/j.jacc.2005.11.062

1136. Kanjani V, Annigeri RG, Revanappa MM, Rani A. Efficacy of Spirulina along with Different Physiotherapeutic Modalities in the Management of Oral Submucous Fibrosis. *Ann Maxillofac Surg*. 2019;9(1):23-27. doi:10.4103/ams.ams_3_19

1137. Kaplan SH. Patient education techniques used at burn centers. *Am J Occup Ther Off Publ Am Occup Ther Assoc*. 1985;39(10):655-658. doi:10.5014/ajot.39.10.655

1138. Kappagoda CT, Ma A, Cort DA, et al. Cardiac event rate in a lifestyle modification program for patients with chronic coronary artery disease. *Clin Cardiol*. 2006;29(7):317-321. doi:10.1002/clc.4960290709

1139. Karlsson J, Fong KSK, Hansson MJ, Elmér E, Csiszar K, Keep MF. Life span extension and reduced neuronal death after weekly intraventricular cyclosporin injections in the G93A transgenic mouse model of amyotrophic lateral sclerosis. *J Neurosurg*. 2004;101(1):128-137. doi:10.3171/jns.2004.101.1.0128

1140. Karnatovskaia L V, Leoni JC, Freeman ML. Cardiac arrest in a 21-year-old man after ingestion of 1,3-DMAA-containing workout supplement. *Clin J Sport Med Off J Can Acad Sport Med*. 2015;25(1):e23-5. doi:10.1097/JSM.0000000000000103

1141. Kartiosuo N, Ramakrishnan R, Lemeshow S, et al. Predicting overweight and obesity in young adulthood from childhood body-mass index: comparison of cutoffs derived from longitudinal and cross-sectional data. *LANCET CHILD Adolesc Heal*. 2019;3(11):795-802. doi:10.1016/S2352-4642(19)30204-4

1142. Kashiwabara K, Kidokoro T, Yanaoka T, Burns SF, Stensel DJ, Miyashita M. Different Patterns of Walking and Postprandial Triglycerides in Older Women. *Med Sci Sports Exerc*. 2018;50(1):79-87. doi:10.1249/MSS.0000000000001413

1143. Kastrup J, Haack-Sørensen M, Juhl M, et al. Cryopreserved Off-the-Shelf Allogeneic Adipose-Derived Stromal Cells for Therapy in Patients with Ischemic Heart Disease and Heart Failure-A Safety Study. *Stem Cells Transl Med*. 2017;6(11):1963-1971. doi:10.1002/sctm.17-0040

1144. Katsanos CS, Grandjean PW, Moffatt RJ. Effects of low and moderate exercise intensity on postprandial lipemia and postheparin plasma lipoprotein lipase activity in physically active men. *J Appl Physiol*. 2004;96(1):181-188. doi:10.1152/japplphysiol.00243.2003

1145. Katsanos CS, Moffatt RJ. Reliability of heart rate responses at given ratings of perceived exertion in cycling and walking. *Res Q Exerc Sport*. 2005;76(4):433-439. doi:10.1080/02701367.2005.10599316

1146. Katsu A, Tyack Z, Mackey M, Elliott JM, Mackenzie L. Return to employment for working-aged adults after burn injury: a scoping review protocol. *BMJ Open*. 2021;11(1):e044145. doi:10.1136/bmjopen-2020-044145

1147. Kaufman CS, Honea RA, Pleen J, et al. Aerobic exercise improves hippocampal blood flow for hypertensive Apolipoprotein E4 carriers. *J Cereb BLOOD FLOW Metab*. 2021;41(8):2026-2037. doi:10.1177/0271678X21990342

1148. Kaufman CS, Vidoni ED, Burns JM, Alwatban MR, Billinger SA. Self-Reported Omega-3 Supplement Use Moderates the Association between Age and Exercising Cerebral Blood Flow Velocity in Older Adults. *Nutrients*. 2020;12(3). doi:10.3390/nu12030697

1149. Kaufman CS, Morris JK, Vidoni ED, Burns JM, Billinger SA. Apolipoprotein E4 Moderates the Association Between Vascular Risk Factors and Brain Pathology. *Alzheimer Dis Assoc Disord*. 2021;35(3):223-229. doi:10.1097/WAD.0000000000000442

1150. Kaur D, Sharma N, Samuel AJ. Web-based E-survey in identifying current physiotherapy practices in paediatric burns. *BURNS*. 2023;49(6):1474-1481. doi:10.1016/j.burns.2023.01.009

1151. Kawabata M, Lee K, Choo HC, Burns SF. Breakfast and Exercise Improve Academic and Cognitive Performance in Adolescents. *Nutrients*. 2021;13(4). doi:10.3390/nu13041278

1152. Kawabata M, Burns SF, Choo HC, Lee K. Weekday breakfast habits and mood at the start of the school morning. *Nutr Health*. 2024;30(1):149-156. doi:10.1177/02601060221105413

1153. Kawakami C, Chung A, Arndt R, et al. Improving Interprofessional Collaboration Between Social Work and Pharmacy Through Hybrid and Virtual Learning Experiences. *Hawai’i J Heal Soc Welf*. 2024;83(2):36-44.

1154. Kawka M, Mak S, Qiu SY, Gall TMH, Jiao LR. Hepatic epithelioid hemangioendothelioma (HEHE)-rare vascular malignancy mimicking cholangiocarcinoma: a case report. *Transl Gastroenterol Hepatol*. 2022;7. doi:10.21037/tgh-20-310

1155. Kayambu G, Boots RJ, Paratz JD. Early rehabilitation in sepsis: a prospective randomised controlled trial investigating functional and physiological outcomes The i-PERFORM Trial (Protocol Article). *BMC Anesthesiol*. 2011;11:21. doi:10.1186/1471-2253-11-21

1156. Kayambu G, Boots R, Paratz J. Early physical rehabilitation in intensive care patients with sepsis syndromes: a pilot randomised controlled trial. *Intensive Care Med*. 2015;41(5):865-874. doi:10.1007/s00134-015-3763-8

1157. Kayambu G, Boots R, Paratz J. Physical therapy for the critically ill in the ICU: a systematic review and meta-analysis. *Crit Care Med*. 2013;41(6):1543-1554. doi:10.1097/CCM.0b013e31827ca637

1158. Kealey GP, Jensen KT. Aggressive approach to physical therapy management of the burned hand. A clinical report. *Phys Ther*. 1988;68(5):683-685. doi:10.1093/ptj/68.5.683

1159. Kearns RD, Cairns BA, Hickerson WL, Holmes 4th JH, Holmes JH 4th. ABA Southern Region Burn disaster plan: the process of creating and experience with the ABA southern region burn disaster plan. *J Burn Care Res*. 2014;35(1):e43-8. doi:10.1097/BCR.0b013e3182957468

1160. Kearns RD, Conlon KM, Valenta AL, et al. Disaster planning: the basics of creating a burn mass casualty disaster plan for a burn center. *J Burn care Res Off Publ Am Burn Assoc*. 2014;35(1):e1-e13. doi:10.1097/BCR.0b013e31829afe25

1161. Kearns RD, Holmes JH 4th, Skarote MB, et al. Disasters; the 2010 Haitian earthquake and the evacuation of burn victims to US burn centers. *Burns*. 2014;40(6):1121-1132. doi:10.1016/j.burns.2013.12.015

1162. Keast R, Sundaresan P, Burns M, Butow PN, Dhillon HM. Exploring head and neck cancer patients’ experiences with radiation therapy immobilisation masks: A qualitative study. *Eur J Cancer Care (Engl)*. 2020;29(2):e13215. doi:10.1111/ecc.13215

1163. Kelly B, Innes A, Holl M, et al. Scalable modEls of Community rehAbilitation for Individuals Recovering From COVID:19 reLated illnEss: A Longitudinal Service Evaluation Protocol-"SeaCole Cohort Evaluation". *Front PUBLIC Heal*. 2021;9. doi:10.3389/fpubh.2021.628333

1164. Kennedy RA, Carroll K, Paterson KL, et al. Physical activity of children and adolescents with Charcot-Marie-Tooth neuropathies: A cross-sectional case-controlled study. *PLoS One*. 2019;14(6). doi:10.1371/journal.pone.0209628

1165. Kenney JL, Carlberg KA. The effect of choline and myo-inositol on liver and carcass fat levels in aerobically trained rats. *Int J Sports Med*. 1995;16(2):114-116. doi:10.1055/s-2007-972975

1166. Kenworthy P, Phillips M, Grisbrook TL, Gibson W, Wood FM, Edgar DW. Monitoring wound healing in minor burns-A novel approach. *BURNS*. 2018;44(1):70-76. doi:10.1016/j.burns.2017.06.007

1167. Kenworthy P, Phillips M, Grisbrook TL, Gibson W, Wood FM, Edgar DW. An objective measure for the assessment and management of fluid shifts in acute major burns. *Burn TRAUMA*. 2018;6. doi:10.1186/s41038-017-0105-9

1168. Kenworthy P, Grisbrook TL, Phillips M, Gibson W, Wood FM, Edgar DW. Addressing the Barriers to Bioimpedance Spectroscopy Use in Major Burns: Alternate Electrode Placement. *J Burn care Res Off Publ Am Burn Assoc*. 2017;38(6):e952-e959. doi:10.1097/BCR.0000000000000527

1169. Kenworthy P, Grisbrook TL, Phillips M, et al. Bioimpedance spectroscopy: A technique to monitor interventions for swelling in minor burns. *Burns*. 2017;43(8):1725-1735. doi:10.1016/j.burns.2017.04.022

1170. Kessel B. Hip fracture prevention in postmenopausal women. *Obstet Gynecol Surv*. 2004;59(6):446-455; quiz 485. doi:10.1097/00006254-200406000-00023

1171. Keteyian SJ, Isaac D, Thadani U, et al. Safety of symptom-limited cardiopulmonary exercise testing in patients with chronic heart failure due to severe left ventricular systolic dysfunction. *Am Heart J*. 2009;158(4 Suppl):S72-7. doi:10.1016/j.ahj.2009.07.014

1172. Key MN, Shaw AR, Erickson KI, Burns JM, Vidoni ED. A retrospective analysis of serious adverse events and deaths in U.S.-based lifestyle clinical trials for cognitive health. *Contemp Clin trials Commun*. 2024;38:101277. doi:10.1016/j.conctc.2024.101277

1173. Key MN, Shaw AR, Erickson KI, Burns JM, Vidoni ED. A Retrospective Analysis of Serious Adverse Events and Deaths in US-Based Lifestyle Clinical Trials for Cognitive Health. *medRxiv Prepr Serv Heal Sci*. Published online September 2023. doi:10.1101/2023.09.27.23296243

1174. KF J, Kwon S, EM L, et al. Sustained effect of early physical activity on body fat mass in older children. *Am J Prev Med*. 2009;37(1):35-40. doi:10.1016/j.amepre.2009.03.012

1175. Khamees KM, Deldar K, Yazarlu O, et al. Effect of augmented reality-based rehabilitation of hand burns on hand function in children: A randomized controlled trial. *J hand Ther Off J Am Soc Hand Ther*. Published online February 2024. doi:10.1016/j.jht.2023.10.009

1176. Khan NA, Hemmelgarn B, Herman RJ, et al. The 2009 Canadian Hypertension Education Program recommendations for the management of hypertension: Part 2--therapy. *Can J Cardiol*. 2009;25(5):287-298. doi:10.1016/s0828-282x(09)70492-1

1177. Khan NA, Hemmelgarn B, Herman RJ, et al. The 2008 Canadian Hypertension Education Program recommendations for the management of hypertension: part 2 - therapy. *Can J Cardiol*. 2008;24(6):465-475. doi:10.1016/s0828-282x(08)70620-2

1178. Khan NA, Hemmelgarn B, Padwal R, et al. The 2007 Canadian Hypertension Education Program recommendations for the management of hypertension: part 2 - therapy. *Can J Cardiol*. 2007;23(7):539-550. doi:10.1016/s0828-282x(07)70798-5

1179. Khataei T, Benson CJ. ASIC3 plays a protective role in delayed-onset muscle soreness (DOMS) through muscle acid sensation during exercise. *Front PAIN Res*. 2023;4. doi:10.3389/fpain.2023.1215197

1180. Khavinson VK, Kuznik BI, Tarnovskaya SI, Lin’kova NS. Short Peptides and Telomere Length Regulator Hormone Irisin. *Bull Exp Biol Med*. 2016;160(3):347-349. doi:10.1007/s10517-016-3167-y

1181. Kho ME, Molloy AJ, Clarke FJ, et al. Multicentre pilot randomised clinical trial of early in-bed cycle ergometry with ventilated patients. *BMJ open Respir Res*. 2019;6(1):e000383. doi:10.1136/bmjresp-2018-000383

1182. Kho ME, Molloy AJ, Clarke F, et al. CYCLE pilot: a protocol for a pilot randomised study of early cycle ergometry versus routine physiotherapy in mechanically ventilated patients. *BMJ Open*. 2016;6(4):e011659. doi:10.1136/bmjopen-2016-011659

1183. Khodasevich LS, Mironov VI, Rassokha IA, Popov GK, Sharapova SA. [Hydrogen sulfide balneotherapy in comprehensive sanatorium-resort treatment of post-burn scars in children]. *Vopr Kurortol Fizioter Lech Fiz Kult*. 2024;101(3):32-40. doi:10.17116/kurort202410103132

1184. Killinger KA, Boura JA, Peters KM. Pain in interstitial cystitis/bladder pain syndrome: do characteristics differ in ulcerative and non-ulcerative subtypes? *Int Urogynecol J*. 2013;24(8):1295-1301. doi:10.1007/s00192-012-2003-9

1185. Kim HD, Hwang SM, Lim KR, Jung YH, Ahn SM, Song JK. Toe Tissue Transfer for Reconstruction of Damaged Digits due to Electrical Burns. *Arch Plast Surg*. 2012;39(2):138-142. doi:10.5999/aps.2012.39.2.138

1186. Kim JB, Cho YS, Jang KU, Joo SY, Choi JS, Seo CH. Effects of sustained release growth hormone treatment during the rehabilitation of adult severe burn survivors. *Growth Horm IGF Res*. 2016;27:1‐6. doi:10.1016/j.ghir.2015.12.009

1187. Kim MO, Burns AS, Ditunno JFJ, Marino RJ. The assessment of walking capacity using the walking index for spinal cord injury: self-selected versus maximal levels. *Arch Phys Med Rehabil*. 2007;88(6):762-767. doi:10.1016/j.apmr.2007.03.021

1188. Kim NR, David K, Corbeels K, et al. Testosterone Reduces Body Fat in Male Mice by Stimulation of Physical Activity Via Extrahypothalamic ERα Signaling. *Endocrinology*. 2021;162(6). doi:10.1210/endocr/bqab045

1189. Kim SC, Lee HJ, Shin DM, et al. Cardiovascular risk in fire academy instructors during live-fire simulation activity. *Ann Burns Fire Disasters*. 2018;31(4):313-321.

1190. Kim T, Sternson SM. Exercise molecule burns away hunger. *Nature*. 2022;606(7915):655-656. doi:10.1038/d41586-022-01321-x

1191. Kim Y, Burns RD, Lee DC, Welk GJ. Associations of movement behaviors and body mass index: comparison between a report-based and monitor-based method using Compositional Data Analysis. *Int J Obes (Lond)*. 2021;45(1):266-275. doi:10.1038/s41366-020-0638-z

1192. Kim ZM, Oh H, Kim HG, Lim CG, Oh KJ, Choi HJ. Modeling long-term human activeness using recurrent neural networks for biometric data. *BMC Med Inform Decis Mak*. 2017;17(Suppl 1):57. doi:10.1186/s12911-017-0453-1

1193. Kimura IF, Stickley CD, Lentz MA, Wages JJ, Yanagi K, Hetzler RK. Validity and reliability of the Hawaii anaerobic run test. *J strength Cond Res*. 2014;28(5):1386-1393. doi:10.1519/JSC.0000000000000261

1194. KING JA, DEIGHTON K, BROOM DR, et al. Individual Variation in Hunger, Energy Intake, and Ghrelin Responses to Acute Exercise. *Med Sci Sport Exerc*. 2017;49(6):1219-1228. doi:10.1249/MSS.0000000000001220

1195. Kirk B, Elliott-Burke T. The effect of visceral manipulation on Diastasis Recti Abdominis (DRA): A case series. *J Bodyw Mov Ther*. 2021;26:471-480. doi:10.1016/j.jbmt.2020.06.007

1196. Kirkham-King M, Brusseau TA, Hannon JC, Castelli DM, Hilton K, Burns RD. Elementary physical education: A focus on fitness activities and smaller class sizes are associated with higher levels of physical activity. *Prev Med reports*. 2017;8:135-139. doi:10.1016/j.pmedr.2017.09.007

1197. Kiss D, Veegh W, Schragel D, Bachl C, Stöllberger C, Sertl K. Bronchial asthma causing symptoms suggestive of angina pectoris. *Eur Respir J*. 2003;21(3):473-477. doi:10.1183/09031936.03.02582001

1198. Kittrell HD, Dimenna FJ, Arad AD, et al. Discrepancy between predicted and measured exercise intensity for eliciting the maximal rate of lipid oxidation. *Nutr Metab Cardiovasc Dis*. 2023;33(11):2189-2198. doi:10.1016/j.numecd.2023.07.014

1199. Klassen A, Di Iorio B, Guastaferro P, Bahner U, Heidland A, De Santo N. High-tone external muscle stimulation in end-stage renal disease: effects on symptomatic diabetic and uremic peripheral neuropathy. *J Ren Nutr Off J Counc Ren Nutr Natl Kidney Found*. 2008;18(1):46-51. doi:10.1053/j.jrn.2007.10.010

1200. Klein GL, Wolf SE, Goodman WG, Phillips WA, Herndon DN. The management of acute bone loss in severe catabolism due to burn injury. *Horm Res*. 1997;48 Suppl 5:83-87. doi:10.1159/000191334

1201. Klein MA, Kadidlo D, McCullough J, McKenna DH, Burns LJ. Microbial contamination of hematopoietic stem cell products: incidence and clinical sequelae. *Biol blood marrow Transplant J Am Soc Blood Marrow Transplant*. 2006;12(11):1142-1149. doi:10.1016/j.bbmt.2006.06.011

1202. Klein P, Wienkötter R. [Cultivation of the epithelium in burns]. *Chirurg*. 1976;47(7):400-405.

1203. Kleinhapl J, Knappskog K, Finnerty CC, Branski L, Suman OE. The Historical Evolvement of Movement and Exercise Training in Patients with Severe Burns and Their Potential Effects on Grafts, Scars, and Function. *Semin Plast Surg*. 2024;38(2):157-161. doi:10.1055/s-0044-1785216

1204. Klionsky DJ, Abdel-Aziz AK, Abdelfatah S, et al. Guidelines for the use and interpretation of assays for monitoring autophagy (4th edition)(1). *Autophagy*. 2021;17(1):1-382. doi:10.1080/15548627.2020.1797280

1205. Knechtle B. [Exercise intensity and fat burning--theoretical principles and practical considerations]. *Praxis (Bern 1994)*. 2002;91(21):915-919. doi:10.1024/0369-8394.91.21.915

1206. Knox ADC, Shih JG, Warren RJ, Gilardino MS, Anastakis DJ. Consensus of Leaders in Plastic Surgery: Identifying Procedural Competencies for Canadian Plastic Surgery Residency Training Using a Modified Delphi Technique. *Plast Reconstr Surg*. 2018;141(68th Annual Meeting of the Canadian-Society-of-Plastic-Surgeons):417E-429E. doi:10.1097/PRS.0000000000004132

1207. Knuth CM, Auger C, Jeschke MG. Burn-induced hypermetabolism and skeletal muscle dysfunction. *Am J Physiol Cell Physiol*. 2021;321(1):C58-C71. doi:10.1152/ajpcell.00106.2021

1208. Koch BM, Wu CM, Randolph J, Eng GD. Heterotopic ossification in children with burns: two case reports. *Arch Phys Med Rehabil*. 1992;73(11):1104-1106.

1209. Kocher MH, Hetzler RK, Shikuma CM, et al. Autonomic Function is Associated with Fitness Level in HIV-Infected Individuals. *Jacobs J AIDS/HIV*. 2015;1(1).

1210. Kocher M, McDermott M, Lindsey R, et al. Short Communication: HIV Patient Systemic Mitochondrial Respiration Improves with Exercise. *AIDS Res Hum Retroviruses*. 2017;33(10):1035-1037. doi:10.1089/AID.2016.0287

1211. Koepke GH. The role of physical medicine in the treatment of burns. *Surg Clin North Am*. 1970;50(6):1385-1399. doi:10.1016/s0039-6109(16)39296-9

1212. Koepke GH, Feller I. Physical measures for the prevention and treatment of deformities following burns. *JAMA*. 1967;199(11):791-793.

1213. Kofoed-Enevoldsen A, Mølvig JC, Zerahn B, Ebbehøj NE. [Zinc chloride smoke pollution. Effects of minimal exposure]. *Ugeskr Laeger*. 1997;159(49):7318-7321.

1214. Kokorina VE, Bykov IA. [Evaluation of the clinical efficacy of the effect of herbal medicine on the symptoms of SARS-COV-2 associated pharyngitis and the formation of post-covid syndrome]. *Vestn Otorinolaringol*. 2023;88(1):35-43. doi:10.17116/otorino20228801135

1215. Kolmus AM, Holland AE, Byrne MJ, Cleland HJ. The effects of splinting on shoulder function in adult burns. *Burns*. 2012;38(5):638‐644. doi:10.1016/j.burns.2012.01.010

1216. Kolodziej F, McDonagh B, Burns N, Goljanek-Whysall K. MicroRNAs as the Sentinels of Redox and Hypertrophic Signalling. *Int J Mol Sci*. 2022;23(23). doi:10.3390/ijms232314716

1217. Komeda M, David TE, Rao V, Sun Z, Weisel RD, Burns RJ. Late hemodynamic effects of the preserved papillary muscles during mitral valve replacement. *Circulation*. 1994;90(5 Pt 2):II190-4.

1218. Kondo T, Tsuboi H, Nishiyama K, Takahashi G, Nishimura Y. Effects of rehabilitation treatments jointly considered by physiatrists and rehabilitation therapists in patients with severe burn injury. *Burns*. 2024;50(6):1621-1631. doi:10.1016/j.burns.2024.03.034

1219. Kondo T, Tsuboi H, Nishiyama K, Takahashi G, Nishimura Y. Effects of rehabilitation treatments jointly considered by physiatrists and rehabilitation therapists in patients with severe burn injury. *Burns*. 2024;50(6):1621-1631. doi:10.1016/j.burns.2024.03.034

1220. Kong PW, Chua YH, Kawabata M, Burns SF, Cai CC. Effect of Post-Exercise Massage on Passive Muscle Stiffness Measured Using Myotonometry -- A Double-Blind Study. *J Sports Sci Med*. 2018;17(4):599-606. https://search.ebscohost.com/login.aspx?direct=true&AuthType=cookie,ip,shib,uid&db=cul&AN=133278320&site=ehost-live&scope=site

1221. Kopchinski B, Lein B, Kopchinski B, Lein B. U.S. Army noncombat munitions injuries. *Mil Med*. 2001;166(2):135-138. https://search.ebscohost.com/login.aspx?direct=true&AuthType=cookie,ip,shib,uid&db=cul&AN=107029167&site=ehost-live&scope=site

1222. Kopel J, Brower GL, Sorensen G, Griswold J. Application of beta-blockers in burn management. *Baylor Univ Med Cent Proc*. 2022;35(1):46-50. doi:10.1080/08998280.2021.2002110

1223. Korn GP, Alvarenga EH de L, Dall’Oglio GP, et al. The Effects in the Upper Airway of Heat and Exposure to Combustion Byproducts of Burning Materials on Larynx of Firefighters. *J Voice*. 2022;36(5):737.e11-737.e16. doi:10.1016/j.jvoice.2020.08.020

1224. Kortmann T, Schumacher G. [Physical activity in obesity and overweight]. *Ther Umsch*. 2013;70(2):113-117. doi:10.1024/0040-5930/a000375

1225. Kotowski SE, Davis KG, Barratt CL. Teachers feeling the burden of COVID-19: Impact on well-being, stress, and burnout. *Work J Prev Assess Rehabil*. 2022;71(2):407-415. doi:10.3233/WOR-210994

1226. Kotz CM, Perez-Leighton CE, Teske JA, Billington CJ. Spontaneous Physical Activity Defends Against Obesity. *Curr Obes Rep*. 2017;6(4):362-370. doi:10.1007/s13679-017-0288-1

1227. Kousar R, Burns C, Lewandowski P. A culturally appropriate diet and lifestyle intervention can successfully treat the components of metabolic syndrome in female Pakistani immigrants residing in Melbourne, Australia. *Metabolism*. 2008;57(11):1502-1508. doi:10.1016/j.metabol.2008.06.003

1228. Kovalenko E V. [Medical gymnastics in generalized tissue defects of the head and neck]. *Med Sestra*. 1988;47(4):34-36.

1229. Kovalenko LN, Bizhko IP, Fomin VA, Khapat’ko GE. [Treatment of the victims of thermal hand injuries]. *Klin Khir*. 1981;(3):32-35.

1230. Kowalske KJ. Hand burns. *Phys Med Rehabil Clin N Am*. 2011;22(2):249-259, vi. doi:10.1016/j.pmr.2011.03.003

1231. Kragh JFJ, Doukas WC, Basamania CJ. Primary coracoid impingement syndrome. *Am J Orthop (Belle Mead NJ)*. 2004;33(5):229-232; discussion 232.

1232. Kramer P, Bressan P. Our (Mother’s) Mitochondria and Our Mind. *Perspect Psychol Sci*. 2018;13(1):88-100. doi:10.1177/1745691617718356

1233. Krause V, Vincent LC, Goncalo JA. Creative ideation activates disinhibited reward-seeking and indulgent choices. *J Pers Soc Psychol*. 2024;127(4):796-821. doi:10.1037/pspa0000408

1234. Kressler J, Jacobs K, Burns P, Betancourt L, Nash MS. Effects of Circuit Resistance Training and P Timely Protein Supplementation on Exercise-Induced Fat Oxidation in Tetraplegic Adults. *Top Spinal Cord Inj Rehabil*. 2014;20(2):113-122. doi:10.1310/sci2002-113

1235. Kressler J, Burns PA, Betancourt L, Nash MS. Circuit Training and Protein Supplementation in Persons with Chronic Tetraplegia. *Med Sci Sport Exerc*. 2014;46(7):1277-1284. doi:10.1249/MSS.0000000000000250

1236. Kressler J, Nash MS, Burns PA, Field-Fote EC. Metabolic responses to 4 different body weight-supported locomotor training approaches in persons with incomplete spinal cord injury. *Arch Phys Med Rehabil*. 2013;94(8):1436-1442. doi:10.1016/j.apmr.2013.02.018

1237. Krizek M, Robbe M, Bilterys L, Vandenbussche F. Treatment of 100 burned hands by early excision and skin grafting. *Ann Chir la main organe Off des Soc Chir la main*. 1982;1(2):125-136. doi:10.1016/s0753-9053(82)80068-9

1238. Krizek M, Robbe M, Vandervord J, Besse MD, Vandenbussche F. [Value of the combination of early excision-graft, kinesitherapy and Jobst compressive gloves in the treatment of burned hands. Preliminary analysis of 62 excised hands]. *Ann Chir Plast*. 1980;25(3):265-270.

1239. Kukuia KKE, Burns FB, Adutwum-Ofosu KK, et al. Increased Brain-Derived Neurotrophic Factor and Hippocampal Dendritic Spine Density Are Associated with the Rapid Antidepressant-like Effect of Iron-citalopram and Iron-Imipramine Combinations in Mice. *Neuroscience*. 2023;519:90-106. doi:10.1016/j.neuroscience.2023.03.014

1240. Kumar R, Hunt CR, Gupta A, et al. Purkinje cell-specific males absent on the first (mMof) gene deletion results in an ataxia-telangiectasia-like neurological phenotype and backward walking in mice. *Proc Natl Acad Sci U S A*. 2011;108(9):3636-3641. doi:10.1073/pnas.1016524108

1241. Kung E, Kahn AM, Burns JC, Marsden A. In Vitro Validation of Patient-Specific Hemodynamic Simulations in Coronary Aneurysms Caused by Kawasaki Disease. *Cardiovasc Eng Technol*. 2014;5(2):189-201. doi:10.1007/s13239-014-0184-8

1242. Kuo CH, Harris MB. Abdominal fat reducing outcome of exercise training: fat burning or hydrocarbon source redistribution? *Can J Physiol Pharmacol*. 2016;94(7):695-698. doi:10.1139/cjpp-2015-0425

1243. Kurti SP, Kurti AN, Emerson SR, et al. Household Air Pollution Exposure and Influence of Lifestyle on Respiratory Health and Lung Function in Belizean Adults and Children: A Field Study. *Int J Environ Res Public Health*. 2016;13(7). doi:10.3390/ijerph13070643

1244. Kurzrock R, Cohen PR. Erythromelalgia and myeloproliferative disorders. *Arch Intern Med*. 1989;149(1):105-109.

1245. Kwan K, Chlebowski RT, McTiernan A, et al. Walking speed, physical activity, and breast cancer in postmenopausal women. *Eur J cancer Prev Off J Eur Cancer Prev Organ*. 2014;23(1):49-52. doi:10.1097/CEJ.0b013e328361627e

1246. Kwon S, Burns TL, Levy SM, Janz KF. Breaks in sedentary time during childhood and adolescence: Iowa bone development study. *Med Sci Sports Exerc*. 2012;44(6):1075-1080. doi:10.1249/MSS.0b013e318245ca20

1247. Kwon S, Janz KF, Letuchy EM, Burns TL, Levy SM. Parental characteristic patterns associated with maintaining healthy physical activity behavior during childhood and adolescence. *Int J Behav Nutr Phys Act*. 2016;13:58. doi:10.1186/s12966-016-0383-9

1248. Kwon S, Janz KF, Letuchy EM, Burns TL, Levy SM. Developmental Trajectories of Physical Activity, Sports, and Television Viewing During Childhood to Young Adulthood: Iowa Bone Development Study. *JAMA Pediatr*. 2015;169(7):666-672. doi:10.1001/jamapediatrics.2015.0327

1249. Kwon S, Burns RD, Kim Y, Bai Y, Byun W. Inter-Device Agreement between Fitbit Flex 1 and 2 for Assessing Sedentary Behavior and Physical Activity. *Int J Environ Res Public Health*. 2021;18(5). doi:10.3390/ijerph18052716

1250. Kwon S, Kim Y, Bai Y, Burns RD, Brusseau TA, Byun W. Validation of the Apple Watch for Estimating Moderate-to-Vigorous Physical Activity and Activity Energy Expenditure in School-Aged Children. *Sensors (Basel)*. 2021;21(19). doi:10.3390/s21196413

1251. Kwon S, Wan N, Burns RD, et al. The Validity of MotionSense HRV in Estimating Sedentary Behavior and Physical Activity under Free-Living and Simulated Activity Settings. *Sensors (Basel)*. 2021;21(4). doi:10.3390/s21041411

1252. L van der L, RJ G, van der Laan L, Goris RJ. Reflex sympathetic dystrophy after a burn injury. *Burn*. 1996;22(4):303-306. https://search.ebscohost.com/login.aspx?direct=true&AuthType=cookie,ip,shib,uid&db=cul&AN=105853786&site=ehost-live&scope=site

1253. La Gerche A, Burns AT, Mooney DJ, et al. Exercise-induced right ventricular dysfunction and structural remodelling in endurance athletes. *Eur Heart J*. 2012;33(8):998-1006. doi:10.1093/eurheartj/ehr397

1254. La Gerche A, Claessen G, Burns AT. To assess exertional breathlessness you must exert the breathless. *Eur J Heart Fail*. 2013;15(7):713-714. doi:10.1093/eurjhf/hft074

1255. La Gerche A, HeidbÜChel H, Burns AT, et al. Disproportionate Exercise Load and Remodeling of the Athlete’s Right Ventricle. *Med Sci Sport Exerc*. 2011;43(6):974-981. doi:10.1249/MSS.0b013e31820607a3

1256. La Gerche A, MacIsaac AI, Burns AT, et al. Pulmonary transit of agitated contrast is associated with enhanced pulmonary vascular reserve and right ventricular function during exercise. *J Appl Physiol*. 2010;109(5):1307-1317. doi:10.1152/japplphysiol.00457.2010

1257. La Shell MS, Tankersley MS, Guerra A. Pruritus, papules, and perspiration. *Ann allergy, asthma Immunol Off Publ Am Coll Allergy, Asthma, Immunol*. 2007;98(3):299-302. doi:10.1016/S1081-1206(10)60723-5

1258. Labandter H, Kaplan I, Shavitt C. Burns of the dorsum of the hand: conservative treatment with intensive physiotherapy versus tangential excision and grafting. *Br J Plast Surg*. 1976;29(4):352-354. doi:10.1016/0007-1226(76)90021-7

1259. Labotz M, Wolff TK, Nakasone KT, Kimura IF, Hetzler RK, Nichols AW. Selective serotonin reuptake inhibitors and rhabdomyolysis after eccentric exercise. *Med Sci Sports Exerc*. 2006;38(9):1539-1542. doi:10.1249/01.mss.0000227643.06478.c6

1260. Lafrance D, Lands LC, Burns DH. Measurement of lactate in whole human blood with near-infrared transmission spectroscopy. *Talanta*. 2003;60(4):635-641. doi:10.1016/S0039-9140(03)00042-0

1261. Laidler NK, Chan J. Treatment of scalp dysesthesia utilising simple exercises and stretches: A pilot study. *Australas J Dermatol*. 2018;59(4):318-321. doi:10.1111/ajd.12807

1262. Lake I. Nutritional ketosis is well-tolerated, even in type 1 diabetes: the ZeroFive100 Project; a proof-of-concept study. *Curr Opin Endocrinol Diabetes Obes*. 2021;28(5):453-462. doi:10.1097/MED.0000000000000666

1263. Lakin R, Polidovitch N, Yang SB, et al. Inhibition of soluble TNFα prevents adverse atrial remodeling and atrial arrhythmia susceptibility induced in mice by endurance exercise. *J Mol Cell Cardiol*. 2019;129:165-173. doi:10.1016/j.yjmcc.2019.01.012

1264. Lal H, Sharma DK, Mittal D. Prosthesis-free solution for below-knee amputations. *Orthopedics*. 2012;35(5):e766-9. doi:10.3928/01477447-20120426-39

1265. Lamb SE, Bruce J, Hossain A, et al. Screening and Intervention to Prevent Falls and Fractures in Older People. *N Engl J Med*. 2020;383(19):1848-1859. doi:10.1056/NEJMoa2001500

1266. Lamberts RP, van Vleuten A, Dumoulin T, Delahaije L, van Erp T. Racing Demands for Winning a Grand Tour: Differences and Similarities Between a Female and a Male Winner. *Int J Sports Physiol Perform*. 2024;19(11):1209-1217. doi:10.1123/ijspp.2023-0476

1267. Landa AM, Starikova MN. [Use of inductothermy, ultraviolet rays, aero-ionization and exercise therapy in the complex treatment of thermal burns]. *Vopr Kurortol Fizioter Lech Fiz Kult*. 1965;30(3):229-232.

1268. Lange-Maia BS, Newman AB, Cauley JA, et al. Sensorimotor Peripheral Nerve Function and the Longitudinal Relationship With Endurance Walking in the Health, Aging and Body Composition Study. *Arch Phys Med Rehabil*. 2016;97(1):45-52. doi:10.1016/j.apmr.2015.08.423

1269. Langer A, Burns RJ, Freeman MR, et al. Reverse redistribution on exercise thallium scintigraphy: relationship to coronary patency and ventricular function after myocardial infarction. *Can J Cardiol*. 1992;8(7):709-715.

1270. Lankao PR, Tribbia JL, Nychka D. Testing theories to explore the drivers of cities’ atmospheric emissions. *Ambio*. 2009;38(4):236-244. doi:10.1579/0044-7447-38.4.236

1271. Lankisch PG, Fuchs K, Schmidt H, Peiper HJ, Creutzfeldt W. [Results of operative treatment of chronic pancreatitis, especially exocrine and endocrine functions (author’s transl)]. *Dtsch Med Wochenschr*. 1975;100(19):1048-1050,1059-1060. doi:10.1055/s-0028-1106333

1272. Lantis KD, Schne P, Bland CR, et al. Biomechanical effect of neurologic dance training (NDT) for breast cancer survivors with chemotherapy-induced neuropathy: study protocol for a randomized controlled trail and preliminary baseline data. *Res Sq*. Published online 2023. doi:10.21203/rs.3.rs-2988661/v1

1273. Lantis KD, Schne P, Bland CR, et al. Biomechanical effect of neurologic dance training (NDT) for breast cancer survivors with chemotherapy-induced neuropathy: study protocol for a randomized controlled trial and preliminary baseline data. *Trials*. 2023;24(1). doi:10.1186/s13063-023-07554-z

1274. Lapshin VP, Buriakov VF, Seraia E V, Shakhlamov M V. [Therapeutic exercise in patients with thermoinhalation trauma at the early hospital stage]. *Vopr Kurortol Fizioter Lech Fiz Kult*. 2004;(6):44-45.

1275. Larwa J, Buchanan TR, Janke RL, et al. Characteristics of rehabilitation protocols following operative treatment of terrible triad elbow injuries and the influence of early motion: A systematic review and meta-analysis. *Shoulder Elb*. Published online August 2024:17585732241269808. doi:10.1177/17585732241269807

1276. LaSCOLA P, Heidorn CE, Pollock B, Burns K, McDANIEL J. Physiological Responses to Counterweighted Single-Leg Cycling in Older Males. *Int J Exerc Sci*. 2020;13(2):1487-1500.

1277. Latessa V. Erythromelalgia: a rare microvascular disease. *J Vasc Nurs Off Publ Soc Peripher Vasc Nurs*. 2010;28(2):67-71. doi:10.1016/j.jvn.2009.11.002

1278. Lateur BJ De, Magyar-russell G, Bresnick MG, et al. Augmented Exercise in the Treatment of Deconditioning From Major Burn Injury. *Arch Phys Med Rehabil*. 2007;88(December):18-23. doi:10.1016/j.apmr.2007.09.003

1279. Latey PJ, Eisenhuth J, McKay MJ, et al. Feasibility of the Archercise biofeedback device to strengthen foot musculature. *J Foot Ankle Res*. 2020;13(1). doi:10.1186/s13047-020-00394-z

1280. Latey PJ, Burns J, Nightingale EJ, Clarke JL, Hiller CE. Reliability and correlates of cross-sectional area of abductor hallucis and the medial belly of the flexor hallucis brevis measured by ultrasound. *J Foot Ankle Res*. 2018;11:28. doi:10.1186/s13047-018-0259-0

1281. Lauer RM, Burns TL, Clarke WR, Mahoney LT. Childhood predictors of future blood pressure. *Hypertens (Dallas, Tex 1979)*. 1991;18(3 Suppl):I74-81. doi:10.1161/01.hyp.18.3_suppl.i74

1282. Laughlin MH, Burns JW, Fanton J, Ripperger J, Peterson DF. Coronary blood flow reserve during +Gz stress and treadmill exercise in miniature swine. *J Appl Physiol*. 1988;64(6):2589-2596. doi:10.1152/jappl.1988.64.6.2589

1283. Lauwens Y, Rafaatpoor F, Corbeel K, Broekmans S, Toelen J, Allegaert K. Immersive Virtual Reality as Analgesia during Dressing Changes of Hospitalized Children and Adolescents with Burns: A Systematic Review with Meta-Analysis. *Children*. 2020;7(11):1-22. doi:10.3390/children7110194

1284. Leao JC, Marques C, Duarte A, de Almeida OP, Porter S, Gueiros LA. Chikungunya fever: General and oral healthcare implications. *Oral Dis*. 2018;24(1-2):233-237. doi:10.1111/odi.12777

1285. Learmonth YC, Assunta H, Skeffington P, Diana W, Kermode AG, Marck CH. Healthcare complexities during community crises: Recommendation for access to healthcare for Australians with multiple sclerosis. *Mult Scler Relat Disord*. 2023;71. doi:10.1016/j.msard.2023.104531

1286. Leatherdale ST, Burns KE, Qian W, Faulkner G, Carson V. Evaluating the Impact of the Healthy Kids Community Challenge (HKCC) on Physical Activity of Older Youth. *Int J Environ Res Public Health*. 2021;18(6). doi:10.3390/ijerph18063083

1287. Lebedeva OD, Achilov AA, Mavlyanova ZF, et al. Is relaxation exercise therapy effective in the management of patients with severe arterial hypertension? *Eur J Transl Myol*. 2021;31(4). doi:10.4081/ejtm.2021.10327

1288. Lebiedowska MK, Gaebler-Spira D, Burns RS, Fisk JR. Biomechanic characteristics of patients with spastic and dystonic hypertonia in cerebral palsy. *Arch Phys Med Rehabil*. 2004;85(6):875-880. doi:10.1016/j.apmr.2003.06.032

1289. Leblebici B, Adam M, Baǧiş S, et al. Quality of life after burn injury: The impact of joint contracture. *J Burn Care Res*. 2006;27(6):864-868. doi:10.1097/01.BCR.0000245652.26648.36

1290. Leblebici B, Sezgin N, Ulusan SN, Tarim AM, Akman MN, Haberal MA. Bone loss during the acute stage following burn injury. *J Burn care Res Off Publ Am Burn Assoc*. 2008;29(5):763-767. doi:10.1097/BCR.0b013e31818480f4

1291. Leciejewska N, Pruszynska-Oszmalek E, Sassek M, et al. Ostarine does not enhance the metabolic effect of exercise in obese rats. *J Physiol Pharmacol an Off J Polish Physiol Soc*. 2023;74(4). doi:10.26402/jpp.2023.4.06

1292. Lee AC, Harvey WF, Wong JB, et al. Effects of Tai Chi versus Physical Therapy on Mindfulness in Knee Osteoarthritis. *Mindfulness (N Y)*. 2017;8(5):1195-1205. doi:10.1007/s12671-017-0692-3

1293. Lee BY, Adam A, Zenkov E, et al. Modeling The Economic And Health Impact Of Increasing Children’s Physical Activity In The United States. *Health Aff (Millwood)*. 2017;36(5):902-908. doi:10.1377/hlthaff.2016.1315

1294. Lee CH, Loh CYY, Chen YH, Lin YT. Proximal Interphalangeal Joint Adipofascial Flap Resurfacing Improves the Active Motion of the Proximal Interphalangeal Joint after Contracture Release. *Plast Reconstr Surg*. 2020;145(2):445-455. doi:10.1097/PRS.0000000000006472

1295. Lee DS, Husain M, Wang X, Austin PC, Iwanochko RM. Cardiovascular outcomes after pharmacologic stress myocardial perfusion imaging. *Am Heart J*. 2016;174:138-146. doi:10.1016/j.ahj.2016.01.012

1296. Lee DS, Verocai F, Husain M, et al. Cardiovascular outcomes are predicted by exercise-stress myocardial perfusion imaging: Impact on death, myocardial infarction, and coronary revascularization procedures. *Am Heart J*. 2011;161(5):900-907. doi:10.1016/j.ahj.2011.01.019

1297. Lee HS, Kim MJ. [A study on fall accident]. *Taehan Kanho*. 1997;36(5):45-62.

1298. Lee JKW, Fan PW, Burns SF. Reply to Dumke, C. Comment on “Fan et al. Efficacy of Ingesting an Oral Rehydration Solution after Exercise on Fluid Balance and Endurance Performance. *Nutrients* 2020, *12*, 3826” *Reply*. *Nutrients*. 2021;13(9). doi:10.3390/nu13093215

1299. Lee JM, Del Balso C, Gupta S, Tay S, Daniels TR, Halai M. A Two-Stage Diabetic Foot Salvage Using Synthetic Bone Void Filler and Lesser Toe Fillet Flap: A Case Report. *JBJS case Connect*. 2022;12(1). doi:10.2106/JBJS.CC.21.00514

1300. Lee JO, Benjamin D, Herndon DN. Nutrition support strategies for severely burned patients. *Nutr Clin Pract Off Publ Am Soc Parenter Enter Nutr*. 2005;20(3):325-330. doi:10.1177/0115426505020003325

1301. Lee JO, Herndon DN, Andersen C, Suman OE, Huang TT. Effect of Exercise Training on the Frequency of Contracture-Release Surgeries in Burned Children. *Ann Plast Surg*. 2017;79(4):346-349. doi:10.1097/SAP.0000000000001071

1302. Lee LY. Respiratory sensations evoked by activation of bronchopulmonary C-fibers. *Respir Physiol Neurobiol*. 2009;167(1):26-35. doi:10.1016/j.resp.2008.05.006

1303. Lee SY, Burns SF, Ng KKC, et al. Fibroblast Growth Factor 21 Mediates the Associations between Exercise, Aging, and Glucose Regulation. *Med Sci Sports Exerc*. 2020;52(2):370-380. doi:10.1249/MSS.0000000000002150

1304. Lee SY, Burns SF, Ng KKC, et al. Pulse Wave Velocity Is Associated with Increased Plasma oxLDL in Ageing but Not with FGF21 and Habitual Exercise. *ANTIOXIDANTS*. 2020;9(3). doi:10.3390/antiox9030221

1305. Lee S, Burns SF, White D, Kuk JL, Arslanian S. Effects of acute exercise on postprandial triglyceride response after a high-fat meal in overweight black and white adolescents. *Int J Obes*. 2013;37(7):966-971. doi:10.1038/ijo.2013.29

1306. Lee SM, Tudor-Locke C, Burns EK. Application of a walking suitability assessment to the immediate built environment surrounding elementary schools. *Health Promot Pract*. 2008;9(3):246-252. doi:10.1177/1524839907301403

1307. Lee T. Ask the doctor. I had a nuclear exercise test last fall, and it was perfectly normal. Imagine my surprise this spring when I developed burning chest pain that turned out to be a heart attack on the bottom part of my heart. Did the doctors mess up the rea. *Harv Heart Lett*. 2010;20(12):8.

1308. Leenen FH, Strauss MH, Chan YK, Burns RJ. Cardiac effects of prazosin in chronic aortic insufficiency. *Can J Cardiol*. 1991;7(6):265-269.

1309. Leggett H, Sandars J, Burns P. Helping students to improve their academic performance: a pilot study of a workbook with self-monitoring exercises. *Med Teach*. 2012;34(9):751-753. doi:10.3109/0142159X.2012.691188

1310. Lehnhard AR, Sehn AP, Silveira JF, Burns RD, Reuter CP, Franke SI. Longitudinal relationship between screen time, cardiorespiratory fitness, and waist circumference of children and adolescents: a 3-year cohort study. *BMC Pediatr*. 2023;23(1). doi:10.1186/s12887-023-04378-3

1311. Lei F, Tang Y, Chen P, Luo H, Wang J, Xie W. [Curative effects of kinesitherapy in combination with self-made simple orthosis in treatment of scar contracture of burned hand in children]. *Zhonghua Shao Shang Za Zhi*. 2014;30(6):477-481.

1312. Leitzelar BN, Koltyn KF. Exercise and Neuropathic Pain: A General Overview of Preclinical and Clinical Research. *Sport Med - open*. 2021;7(1):21. doi:10.1186/s40798-021-00307-9

1313. Leman CJ. Splints and accessories following burn reconstruction. *Clin Plast Surg*. 1992;19(3):721-731.

1314. Lemes VB, Sehn AP, Reuter CP, et al. Associations of sleep time, quality of life, and obesity indicators on physical literacy components: a structural equation model. *BMC Pediatr*. 2024;24(1):159. doi:10.1186/s12887-024-04609-1

1315. Lemière C, Malo JL, Garbe-Galanti L. [Bronchial irritation syndrome following inhalation or urea. Histologic and immunohistochemical evaluation]. *Rev Mal Respir*. 1996;13(6):595-597.

1316. Lemmey S, Burns J, Jones F. Developing additional competition classes for athletes with intellectual impairments: Conceptual approach and efficacy of an ICF derived measure. *J Sports Sci*. 2021;39(sup1):99-108. doi:10.1080/02640414.2021.1881302

1317. Lenne RL, Mann T, Burns RJ, Vickers Z, Redden JP. Variety Salience and Enjoyment of Repetitiously Consumed Foods: a Field Experiment. *Int J Behav Med*. 2021;28(3):286-291. doi:10.1007/s12529-020-09916-2

1318. Lerch S, Berndt T, Lipka W, Rühmann O. [Screw arthrodesis of the shoulder]. *Oper Orthop Traumatol*. 2011;23(3):215-226. doi:10.1007/s00064-011-0035-9

1319. Lesho E, Lesho E. Prospective data, experience, and lessons learned at a surgically augmented brigade medical company (Level II+) during the 2007 Iraq surge. *Mil Med*. 2011;176(7):763-768. doi:10.7205/MILMED-D-10-00330

1320. Lestyk KC, Folkow LP, Blix AS, Hammill MO, Burns JM. Development of myoglobin concentration and acid buffering capacity in harp (Pagophilus groenlandicus) and hooded (Cystophora cristata) seals from birth to maturity. *J Comp Physiol B, Biochem Syst Environ Physiol*. 2009;179(8):985-996. doi:10.1007/s00360-009-0378-9

1321. Leth PM, Banner J. Forensic medical examination of refugees who claim to have been tortured. *Am J Forensic Med Pathol*. 2005;26(2):125-130.

1322. Leung AA, Daskalopoulou SS, Dasgupta K, et al. Hypertension Canada’s 2017 Guidelines for Diagnosis, Risk Assessment, Prevention, and Treatment of Hypertension in Adults. *Can J Cardiol*. 2017;33(5):557-576. doi:10.1016/j.cjca.2017.03.005

1323. Leung AA, Nerenberg K, Daskalopoulou SS, et al. Hypertension Canada’s 2016 Canadian Hypertension Education Program Guidelines for Blood Pressure Measurement, Diagnosis, Assessment of Risk, Prevention, and Treatment of Hypertension. *Can J Cardiol*. 2016;32(5):569-588. doi:10.1016/j.cjca.2016.02.066

1324. Leung B, Younger JF, Stockton K, Muller M, Paratz J. Cardiovascular risk profile in burn survivors. *Burn*. 2017;43(7):1411-1417. doi:10.1016/j.burns.2017.07.010

1325. Leuzzi S, Maruccia M, Elia R, et al. Lymphatic-venous anastomosis in a rat model: A novel exercise for microsurgical training. *J Surg Oncol*. 2018;118(6):936-940. doi:10.1002/jso.25234

1326. Levine N. Dermatologic aspects of sports medicine. *J Am Acad Dermatol*. 1980;3(4):415-424. doi:10.1016/s0190-9622(80)80337-9

1327. Lewin HC, Hachamovitch R, Harris AG, et al. Sustained reduction of exercise perfusion defect extent and severity with isosorbide mononitrate (Imdur) as demonstrated by means of technetium 99m sestamibi. *J Nucl Cardiol Off Publ Am Soc Nucl Cardiol*. 2000;7(4):342-353. doi:10.1067/mnc.2000.106966

1328. Lewis MI, Fournier M, Storer TW, et al. Skeletal muscle adaptations to testosterone and resistance training in men with COPD. *J Appl Physiol*. 2007;103(4):1299-1310. doi:10.1152/japplphysiol.00150.2007

1329. Lewis MI, Fournier M, Wang H, et al. Metabolic and morphometric profile of muscle fibers in chronic hemodialysis patients. *J Appl Physiol*. 2012;112(1):72-78. doi:10.1152/japplphysiol.00556.2011

1330. Lewis MI, Fournier M, Wang H, Storer TW, Casaburi R, Kopple JD. Effect of endurance and/or strength training on muscle fiber size, oxidative capacity, and capillarity in hemodialysis patients. *J Appl Physiol*. 2015;119(8):865-871. doi:10.1152/japplphysiol.01084.2014

1331. Li H, Zhang J, Chen J, et al. [Integration of burn treatment and rehabilitation for a child with extremely severe burn]. *Zhonghua Shao Shang Za Zhi*. 2015;31(2):130-134.

1332. Li JX, Xia YM, Xu HH, et al. Activation of brain lactate receptor GPR81 aggravates exercise-induced central fatigue. *Am J Physiol Integr Comp Physiol*. 2022;323(5):R822-R831. doi:10.1152/ajpregu.00094.2022

1333. Li JD, Lv GZ, Abdelbasset WK, et al. Response to Letter to the Editor on “Potential efficacy of sensorimotor exercise program on pain, proprioception, mobility, and quality of life in diabetic patients with foot burns: a 12-week randomized control study.” *Burns*. 2021;47(3):587-593. doi:10.1016/j.burns.2020.08.002

1334. Li J, Xu X, Li W, Zhang X. Behavioural and physiological responses to low- and high-intensity locomotion in Chinese shrimp Fenneropenaeus chinensis. *J Comp Physiol A, Neuroethol sensory, neural, Behav Physiol*. 2019;205(1):87-102. doi:10.1007/s00359-018-1306-9

1335. Li JJ, Ping Z, Wang ZW, Wang YB, Shi CM, Cao XB. [Experimental study of mitochondrion-targeted small molecule IR-61 ameliorated exhaustive exercise-induced cardiac injury in rats]. *Zhongguo ying yong sheng li xue za zhi = Zhongguo yingyong shenglixue zazhi = Chinese J Appl Physiol*. 2022;38(5):497-503. doi:10.12047/j.cjap.6260.2022.093

1336. Li L, Dai JX, Xu L, et al. The effect of a rehabilitation nursing intervention model on improving the comprehensive health status of patients with hand burns. *Burns*. 2017;43(4):877‐885. doi:10.1016/j.burns.2016.11.003

1337. Li M, Zeng XC, Xie WG. [Clinical characteristics of treadmill abrasion in children]. *Zhonghua Shao Shang Za Zhi*. 2020;36(10):966-968. doi:10.3760/cma.j.cn501120-20191022-00408

1338. Li N, Fu QQ, Luo Y, Li MJ, Chen HL, Liao JM. [Application effects of rehabilitation care decision-making scheme based on case management model in severe burn patients]. *Zhonghua shao shang yu chuang mian xiu fu za zhi*. 2024;40(1):78-86. doi:10.3760/cma.j.cn501225-20230905-00078

1339. Li Q, Ba T, Wang LF, Chen Q, Li F, Xue Y. Stratification of venous thromboembolism risk in burn patients by Caprini score. *Burns*. 2019;45(1):140-145. doi:10.1016/j.burns.2018.08.006

1340. Liao F, An R, Pu F, Burns S, Shen S, Jan YK. Effect of Exercise on Risk Factors of Diabetic Foot Ulcers: A Systematic Review and Meta-Analysis. *Am J Phys Med Rehabil*. 2019;98(2):103-116. doi:10.1097/PHM.0000000000001002

1341. Liew F, Efstathiou C, Fontanella S, et al. Large-scale phenotyping of patients with long COVID post-hospitalization reveals mechanistic subtypes of disease. *Nat Immunol*. 2024;25(4):607-621. doi:10.1038/s41590-024-01778-0

1342. Lim E, Davis J, Chen JJ. The Association of Race/Ethnicity, Dietary Intake, and Physical Activity with Depression. *J RACIAL Ethn Heal DISPARITIES*. 2021;8(2):315-331. doi:10.1007/s40615-020-00784-w

1343. Lima WP, Carnevali LCJ, Eder R, Costa Rosa LFBP, Bacchi EM, Seelaender MCL. Lipid metabolism in trained rats: effect of guarana (Paullinia cupana Mart.) supplementation. *Clin Nutr*. 2005;24(6):1019-1028. doi:10.1016/j.clnu.2005.08.004

1344. Lin AD, Miles K, Brinks M V. Prevalence of Pterygia in Hawaii: Examining Cumulative Surfing Hours as a Risk Factor. *Ophthalmic Epidemiol*. 2016;23(4):264-268. doi:10.3109/09286586.2015.1119284

1345. Lin YC, Shida KK. Mechanisms of hyperbaric bradycardia. *Chin J Physiol*. 1988;31(1):1-22.

1346. Lin YC, Shiraki K, Takeuchi H, Mohri M. Cardiovascular deconditioning occurs during a 7-day saturation dive at 31 ATA. *Aviat Space Environ Med*. 1995;66(7):656-660.

1347. Lin YR, Wang JY, Chang SC, et al. Developing a Delphi-Based Comprehensive Core Set from the International Classification of Functioning, Disability, and Health Framework for the Rehabilitation of Patients with Burn Injuries. *Int J Environ Res Public Health*. 2021;18(8). doi:10.3390/ijerph18083970

1348. Lindeborg MM, Shakya P, Pradhan B, et al. A task-shifted speech therapy program for cleft palate patients in rural Nepal: Evaluating impact and associated healthcare barriers. *Int J Pediatr Otorhinolaryngol*. 2020;134. doi:10.1016/j.ijporl.2020.110026

1349. Lindenhovius ALC, van de Luijtgaarden K, Ring D, Jupiter J. Open elbow contracture release: postoperative management with and without continuous passive motion. *J Hand Surg Am*. 2009;34(5):858-865. doi:10.1016/j.jhsa.2009.01.003

1350. Linder BA, Agostinelli PJ, Bordonie NC, Stute NL. Turn up and burn up: aerobic exercise training as a strategy to preserve non-shivering thermogenesis on an obesogenic diet. *J Physiol*. 2023;601(7):1179-1181. doi:10.1113/JP284459

1351. Lindstrom AR, von Schuckmann LA, Hughes MCB, Williams GM, Green AC, van der Pols JC. Regular Sunscreen Use and Risk of Mortality: Long-Term Follow-up of a Skin Cancer Prevention Trial. *Am J Prev Med*. 2019;56(5):742-746. doi:10.1016/j.amepre.2018.11.025

1352. Ling H, Lenz TL, Burns TL, Hilleman DE. Reducing the risk of obesity: defining the role of weight loss drugs. *Pharmacotherapy*. 2013;33(12):1308-1321. doi:10.1002/phar.1277

1353. Ling XW, Jiang X, Guo HL, Zhang TT. Deep burn surgery of the whole dorsum of the hand: Composite skin grafting over acellular dermal matrix versus thick split-thickness skin grafting. *Int Wound J*. 2024;21(5):e14934. doi:10.1111/iwj.14934

1354. Lipman GS, Burns P, Phillips C, et al. Effect of Sodium Supplements and Climate on Dysnatremia During Ultramarathon Running. *Clin J Sport Med*. 2021;31(Summer Conference of the Wilderness-Medical-Society):E327-E334. doi:10.1097/JSM.0000000000000832

1355. Lipman GS, Hew-Butler T, Phillips C, Krabak B, Burns P. Prospective Observational Study of Weight-based Assessment of Sodium Supplements on Ultramarathon Performance (WASSUP). *Sport Med*. 2021;7(1). doi:10.1186/s40798-021-00302-0

1356. Lipman GS, Shea K, Christensen M, et al. Ibuprofen versus placebo effect on acute kidney injury in ultramarathons: a randomised controlled trial. *Emerg Med J*. 2017;34(10):637-642. doi:10.1136/emermed-2016-206353

1357. Litschauer-Poursadrollah M, Mayer DE, Hemmer W, Jarisch R. [Jellyfish and poison-producing animals that endanger swimmers]. *Dtsch Med Wochenschr*. 2010;135(21):1073-1077. doi:10.1055/s-0030-1253702

1358. Little JW, Burns SP, James JJ, Stiens SA. Neurologic recovery and neurologic decline after spinal cord injury. *Phys Med Rehabil Clin N Am*. 2000;11(1):73-89.

1359. Little M, Stone T, Stone R, et al. The evacuation of cairns hospitals due to severe tropical cyclone Yasi. *Acad Emerg Med Off J Soc Acad Emerg Med*. 2012;19(9):E1088-98. doi:10.1111/j.1553-2712.2012.01439.x

1360. Liu CB, Xie HT, Wei P, et al. Clinical study of early rehabilitation training combined with negative pressure wound therapy for the treatment of deep partial-thickness hand burns. *Front Surg*. 2023;10:1040407. doi:10.3389/fsurg.2023.1040407

1361. Liu Q, Deng S, Wang Y. [Fiberoptic bronchoscopy in diagnosis and treatment of inhalation injury]. *Zhonghua zheng xing shao shang wai ke za zhi = Zhonghua zheng xing shao shang waikf [i.e waike] zazhi = Chinese J Plast Surg Burn*. 1999;15(3):218-219.

1362. Liu S, Zheng J, Zheng Q. A Case Report of Heterotopic Ossification Caused by High-Voltage Electric Injury. *J Burn Care Res*. 2020;41(5):1118-1121. doi:10.1093/jbcr/iraa076

1363. Lloyd EL. ABC of sports medicine. Temperature and performance--II: Heat. *BMJ*. 1994;309(6954):587-589. doi:10.1136/bmj.309.6954.587

1364. Lo CH, Nothdurft SH, Park HS, Paul E, Leong J. Distraction ligamentotaxis for complex proximal interphalangeal joint fracture dislocations: a clinical study and the modified pins rubber band traction system revisited. *Burn trauma*. 2018;6:23. doi:10.1186/s41038-018-0124-1

1365. Lohana P, Hemington-Gorse S, Thomas C, Potokar T, Wilson YT. Paediatric injuries due to home treadmill use: an emerging problem. *Ann R Coll Surg Engl*. 2012;94(2):121-123. doi:10.1308/003588412X13171221501942

1366. Lohman EB 3rd, Bains GS, Lohman T, DeLeon M, Petrofsky JS. A comparison of the effect of a variety of thermal and vibratory modalities on skin temperature and blood flow in healthy volunteers. *Med Sci Monit Int Med J Exp Clin Res*. 2011;17(9):MT72-81. doi:10.12659/msm.881921

1367. Long F. A healing machine. Rapid return to function makes continuous passive motion “must-have” technology. *Rehab Manag*. 2008;21(5):34-36.

1368. Long JE, Ring C, Bosch JA, et al. A life-style physical activity intervention and the antibody response to pneumococcal vaccination in women. *Psychosom Med*. 2013;75(8):774-782. doi:10.1097/PSY.0b013e3182a0b664

1369. Long JE, Ring C, Drayson M, et al. Vaccination response following aerobic exercise: can a brisk walk enhance antibody response to pneumococcal and influenza vaccinations? *Brain Behav Immun*. 2012;26(4):680-687. doi:10.1016/j.bbi.2012.02.004

1370. Lorenzo AR, Alvarez A, Garcia-Barreiro J, Centeno A, Lopez E, Martelo F. Design and creation of an experimental program of advanced training in reconstructive microsurgery. *Microsurgery*. 2006;26(6):421-428. doi:10.1002/micr.20265

1371. Louton H, Keppler C, Erhard M, et al. Animal-based welfare indicators of 4 slow-growing broiler genotypes for the approval in an animal welfare label program. *Poult Sci*. 2019;98(6):2326-2337. doi:10.3382/ps/pez023

1372. Lox CL, Burns SP, Treasure DC, Wasley DA. Physical and psychological predictors of exercise dosage in healthy adults. *Med Sci Sports Exerc*. 1999;31(7):1060-1064. doi:10.1097/00005768-199907000-00021

1373. Lucas RAI, Skinner BD, Arias-Monge E, et al. Targeting workload to ameliorate risk of heat stress in industrial sugarcane workers. *Scand J Work Environ Health*. 2023;49(1):43-52. doi:10.5271/sjweh.4057

1374. Lukkahatai N, Soivong P, Li D, et al. Feasibility of Using Mobile Technology to Improve Physical Activity Among People Living with Diabetes in Asia. *Asian Pacific Isl Nurs J*. 2021;5(4):236-247. doi:10.31372/20200504.1110

1375. Lum DP, Coel MN. Comparison of automatic quantification software for the measurement of ventricular volume and ejection fraction in gated myocardial perfusion SPECT. *Nucl Med Commun*. 2003;24(3):259-266. doi:10.1097/00006231-200303000-00005

1376. Luo M, Nguyen B, Nau T, et al. A Holistic Way to Understand the Determinants of Physical Activity in Urban New South Wales, Australia: A Codesigned Systems Mapping Project. *J Phys Act Health*. 2024;21(12):1325-1329. doi:10.1123/jpah.2024-0359

1377. Luquet S, Gaudel C, Holst D, et al. Roles of PPAR delta in lipid absorption and metabolism: a new target for the treatment of type 2 diabetes. *Biochim Biophys Acta*. 2005;1740(2):313-317. doi:10.1016/j.bbadis.2004.11.011

1378. Luquet S, Lopez-Soriano J, Holst D, et al. Roles of peroxisome proliferator-activated receptor delta (PPARdelta) in the control of fatty acid catabolism. A new target for the treatment of metabolic syndrome. *Biochimie*. 2004;86(11):833-837. doi:10.1016/j.biochi.2004.09.024

1379. Ly CL, Chun MBJ. Welcome to cultural competency: surgery’s efforts to acknowledge diversity in residency training. *J Surg Educ*. 2013;70(2):284-290. doi:10.1016/j.jsurg.2012.10.005

1380. Lye CT, Mukherjee S, Burns SF. Combining Plant Sterols With Walking Lowers Postprandial Triacylglycerol More Than Walking Only in Chinese Men With Elevated Body Mass Index. *Int J Sport Nutr Exerc Metab*. 2019;29(6):576-582. doi:10.1123/ijsnem.2018-0398

1381. Lymperopoulos NS, Jeevan R, Godwin L, Wilkinson D, Shokrollahi K, James MI. The Introduction of Standard Operating Procedures to Improve Burn Care in the United Kingdom. *J Burn Care Res*. 2015;36(5):565-573. doi:10.1097/BCR.0000000000000210

1382. Lynch GS, Schertzer JD, Ryall JG. Therapeutic approaches for muscle wasting disorders. *Pharmacol Ther*. 2007;113(3):461-487. doi:10.1016/j.pharmthera.2006.11.004

1383. Lysak A, Farnebo S, Geuna S, Dahlin LB. Muscle preservation in proximal nerve injuries: a current update. *J Hand Surg Eur Vol*. 2024;49(6):773-782. doi:10.1177/17531934231216646

1384. Lyu GZ, Xu LW. [Lay emphasis on early rehabilitation after extensive burn to prevent severe complications in late stage]. *Zhonghua Shao Shang Za Zhi*. 2017;33(5):257-259. doi:10.3760/cma.j.issn.1009-2587.2017.05.001

1385. Ma C, Wong L, Wen A, et al. Evaluation of distance facilitation and technology in an interprofessional simulation exercise. *Curr Pharm Teach Learn*. 2020;12(7):776-785. doi:10.1016/j.cptl.2020.02.007

1386. Ma JE, Lee JUJ, Sartori-Valinotti JC, Rooke TW, Sandroni P, Davis MDP. Erythromelalgia: A Review of Medical Management Options and Our Approach to Management. *Mayo Clin Proc*. 2023;98(1):136-149. doi:10.1016/j.mayocp.2022.08.005

1387. MacDessi SJ, Bhimani A, Burns AWR, et al. Does soft tissue balancing using intraoperative pressure sensors improve clinical outcomes in total knee arthroplasty? A protocol of a multicentre randomised controlled trial. *BMJ Open*. 2019;9(5):e027812. doi:10.1136/bmjopen-2018-027812

1388. Macfarlane A, O’Donnell C, Mair F, et al. REsearch into implementation STrategies to support patients of different ORigins and language background in a variety of European primary care settings (RESTORE): study protocol. *Implement Sci*. 2012;7(1):111. doi:10.1186/1748-5908-7-111

1389. Machová K, Dadová K, Chaloupková H, Svobodová I. Does having a pet influence the physical activity of their young female owners? *BMC Public Health*. 2019;19(1). doi:10.1186/s12889-019-7962-z

1390. Mackey L, White MJ, Tyack Z, Finlayson G, Dalton M, King NA. A dual-process psychobiological model of temperament predicts liking and wanting for food and trait disinhibition. *Appetite*. 2019;134:9-16. doi:10.1016/j.appet.2018.12.011

1391. Mackin EJ. Prevention of complications in hand therapy. *Hand Clin*. 1986;2(2):429-447.

1392. MacRoberts K. Stretching exercises for the burn patient. *Phys Ther*. 1979;59(6):769. doi:10.1093/ptj/59.6.769a

1393. Madden JW, Enna CD. The management of acute thermal injuries to the upper extremity. *J Hand Surg Am*. 1983;8(5 Pt 2):785-788. doi:10.1016/s0363-5023(83)80273-1

1394. Magkos F, Astrup A. Dietary Carbohydrate, Energy Expenditure, and Weight Loss: Is Eating Less and Burning More Possible? Comment. *J Nutr*. 2021;151(3):468-470. doi:10.1093/jn/nxaa423

1395. Magnani DM, Sassi FC, Vana LPM, et al. Orofacial rehabilitation after severe orofacial and neck burn: Experience in a Brazilian burn reference centre. *Burn*. 2021;47(2):439-446. doi:10.1016/j.burns.2020.07.011

1396. Magre J, Leroux P, Lerat MF, Herve P, Ledanois A. [Sterilization by celioscopy]. *Ouest Med*. 1973;26(12):1295-1299.

1397. Mahalik JR, Burns SM, Syzdek M. Masculinity and perceived normative health behaviors as predictors of men’s health behaviors. *Soc Sci Med*. 2007;64(11):2201-2209. doi:10.1016/j.socscimed.2007.02.035

1398. Mahfouz FM, Li T, Timmins HC, et al. Impact of Pain on Symptom Burden in Chemotherapy-Induced Peripheral Neurotoxicity. *J Natl Compr Canc Netw*. 2024;22(2):108-116. doi:10.6004/jnccn.2023.7083

1399. MAISELS DO. THE MIDDLE SLIP OR BOUTONNIERE DEFORMITY IN BURNED HANDS. *Br J Plast Surg*. 1965;18:117-129. doi:10.1016/s0007-1226(65)80015-7

1400. Majhail NS, Ness KK, Burns LJ, et al. Late effects in survivors of Hodgkin and non-Hodgkin lymphoma treated with autologous hematopoietic cell transplantation: a report from the bone marrow transplant survivor study. *Biol blood marrow Transplant J Am Soc Blood Marrow Transplant*. 2007;13(10):1153-1159. doi:10.1016/j.bbmt.2007.06.003

1401. Malik SS, Tassadaq N. Effectiveness of Deep Breathing Exercises and Incentive Spirometry on Arterial Blood Gases in Second Degree Inhalation Burn Patients. *J Coll Physicians Surg Pak*. 2019;29(10):954‐957. doi:10.29271/jcpsp.2019.10.954

1402. Malloizel-Delaunay J, Chantalat E, Bongard V, et al. Endermology treatment for breast cancer related lymphedema (ELOCS): Protocol for a phase II randomized controlled trial. *Eur J Obstet Gynecol Reprod Biol*. 2019;241:35-41. doi:10.1016/j.ejogrb.2019.07.040

1403. Małyszko J, Bachorzewska-Gajewska H, Tomaszuk-Kazberuk A, Matuszkiewicz-Rowińska J, Durlik M, Dobrzycki S. Cardiovascular disease and kidney transplantation‑evaluation of potential transplant recipient. *Pol Arch Med Wewn*. 2014;124(11):608-616.

1404. Mamon MA, Olthof SBH, Burns GT, Lepley AS, Kozloff KM, Zernicke RF. Position-Specific Physical Workload Intensities in American Collegiate Football Training. *J STRENGTH Cond Res*. 2022;36(2):420-426. doi:10.1519/JSC.0000000000004174

1405. Mandarakas MR, Young P, Burns J. Neuromuscular rehabilitation - what to do? *Curr Opin Neurol*. 2021;34(5):697-705. doi:10.1097/WCO.0000000000000974

1406. Mangan S, Malone S, Ryan M, et al. Influence of Team Rating on Running Performance in Elite Gaelic Football. *J strength Cond Res*. 2018;32(9):2584-2591. doi:10.1519/JSC.0000000000002316

1407. Mangan S, Ryan M, Shovlin A, et al. Seasonal Changes in Gaelic Football Match-Play Running Performance. *J strength Cond Res*. 2019;33(6):1685-1691. doi:10.1519/JSC.0000000000002269

1408. Mangge H, Ciardi C, Becker K, Strasser B, Fuchs D, Gostner JM. Influence of Antioxidants on Leptin Metabolism and its Role in the Pathogenesis of Obesity. *Adv Exp Med Biol*. 2017;960:399-413. doi:10.1007/978-3-319-48382-5_17

1409. Manore MM, Larson-Meyer DE, Lindsay AR, Hongu N, Houtkooper L. Dynamic Energy Balance: An Integrated Framework for Discussing Diet and Physical Activity in Obesity Prevention-Is it More than Eating Less and Exercising More? *Nutrients*. 2017;9(8). doi:10.3390/nu9080905

1410. Manouras L, Bastian JD, Beckmann NA, Tosounidis TH. The Top Three Burning Questions in Total Hip Arthroplasty. *Medicina (Kaunas)*. 2023;59(4). doi:10.3390/medicina59040655

1411. Maraki M, Sidossis LS. Effects of energy balance on postprandial triacylglycerol metabolism. *Curr Opin Clin Nutr Metab Care*. 2010;13(6):608-617. doi:10.1097/MCO.0b013e32833f1aae

1412. Marfleet P. Ultimate injuries: a survey. *Br J Sports Med*. 1991;25(4):235-240. doi:10.1136/bjsm.25.4.235

1413. Marino RJ, Scivoletto G, Patrick M, et al. Walking index for spinal cord injury version 2 (WISCI-II) with repeatability of the 10-m walk time: Inter- and intrarater reliabilities. *Am J Phys Med Rehabil*. 2010;89(1):7-15. doi:10.1097/PHM.0b013e3181c560eb

1414. Mars D, Davis BL, Montgomery AJ, Gregoski MJ, Burns DP, Coffey D. The Lived Experience of African-American Informal Caregivers of Family Members with Alzheimer’s Disease and Related Dementias. *J Natl Black Nurses Assoc*. 2017;28(2):19-25.

1415. Marshall HC, Hamlin MJ, Hellemans J, et al. Effects of intermittent hypoxia on SaO(2), cerebral and muscle oxygenation during maximal exercise in athletes with exercise-induced hypoxemia. *Eur J Appl Physiol*. 2008;104(2):383-393. doi:10.1007/s00421-007-0616-3

1416. Marshall J, Lourie GM. Pediatric hand friction burn injuries secondary to treadmills. *J Pediatr Orthop*. 2003;23(3):407-409.

1417. Martin L, Rea S, Wood F. A quantitative analysis of the relationship between posttraumatic growth, depression and coping styles after burn. *Burns*. 2021;47(8):1748-1755. doi:10.1016/j.burns.2021.05.019

1418. Martyn JA, White DA, Gronert GA, Jaffe RS, Ward JM. Up-and-down regulation of skeletal muscle acetylcholine receptors. Effects on neuromuscular blockers. *Anesthesiology*. 1992;76(5):822-843. doi:10.1097/00000542-199205000-00022

1419. Marzuki MIH, Mohamad MI, Chai WJ, et al. Prevalence of Relative Energy Deficiency in Sport (RED-S) among National Athletes in Malaysia. *Nutrients*. 2023;15(7). doi:10.3390/nu15071697

1420. Mascarenhas DD, El Ayadi A, Ravikumar P, et al. Positive effects of ferric iron on the systemic efficacy of nephrilin peptide in burn trauma. *Scars, Burn Heal*. 2020;6:2059513120928494. doi:10.1177/2059513120928494

1421. Maskarinec GG, Yalmadau K, Maluchmai MR, Tun P, Yinnifel C, Hancock WT. Palliative care and traditional practices of death and dying in Wa’ab (Yap Proper) and in the Outer Islands of Yap. *Hawaii Med J*. 2011;70(11 Suppl 2):27-30.

1422. Massarsky A, Abdel A, Glazer L, Levin ED, Di Giulio RT. Exposure to 1,2-Propanediol Impacts Early Development of Zebrafish (Danio rerio) and Induces Hyperactivity. *Zebrafish*. 2017;14(3):216-222. doi:10.1089/zeb.2016.1400

1423. Masters NJ, Burns FM, Lewis JCM. Peri-anaesthetic and anaesthetic-related mortality risks in great apes (Hominidae) in zoological collections in the UK and Ireland. *Vet Anaesth Analg*. 2007;34(6):431-442. doi:10.1111/j.1467-2995.2007.00345.x

1424. Mathews AL, Coleska A, Burns PB, Chung KC. Evolution of Patient Decision-Making Regarding Medical Treatment of Rheumatoid Arthritis. *Arthritis Care Res (Hoboken)*. 2016;68(3):318-324. doi:10.1002/acr.22688

1425. Mattson RE, Burns RD, Brusseau TA, Metos JM, Jordan KC. Comprehensive School Physical Activity Programming and Health Behavior Knowledge. *Front PUBLIC Heal*. 2020;8. doi:10.3389/fpubh.2020.00321

1426. Matveev BA. [Medical triage of the wounded and burned in a medical battalion under conditions of serious overload. 3. Triage of the wounded in military medical field exercises]. *Voen Med Zh*. 1973;4:11-14.

1427. Matzkin E, Singer DI. Scaphoid stress fracture in a 13-year-old gymnast: a case report. *J Hand Surg Am*. 2000;25(4):710-713. doi:10.1053/jhsu.2000.7382

1428. Mau MK, Glanz K, Severino R, Grove JS, Johnson B, Curb JD. Mediators of lifestyle behavior change in Native Hawaiians: initial findings from the Native Hawaiian Diabetes Intervention Program. *Diabetes Care*. 2001;24(10):1770-1775. doi:10.2337/diacare.24.10.1770

1429. Mau M, Minami CM, Stotz SA, Albright CL, Kana’iaupuni SM, Guth HK. Qualitative study on voyaging and health: perspectives and insights from the medical officers during the Worldwide Voyage. *BMJ Open*. 2021;11(7). doi:10.1136/bmjopen-2021-048767

1430. Mavanji V, Pomonis B, Kotz CM. Orexin, serotonin, and energy balance. *WIRES Mech Dis*. 2022;14(1). doi:10.1002/wsbm.1536

1431. May J, Colbert D, Rea S, Wood F, Nara-Venkata R. Preparedness and training in staff responding to a burns disaster. *Br J Nurs*. 2015;24(18):918-923. doi:10.12968/bjon.2015.24.18.918

1432. Mc Kittrick A, Gustafsson L, Hodson T, Di Tommaso A. Exploration of individuals perspectives of recovery following severe hand burn injuries. *Burns*. 2023;49(2):467-475. doi:10.1016/j.burns.2022.04.026

1433. McAuley HJC, Evans RA, Bolton CE, et al. Prevalence of physical frailty, including risk factors, up to 1 year after hospitalisation for COVID-19 in the UK: a multicentre, longitudinal cohort study. *EClinicalMedicine*. 2023;57:101896. doi:10.1016/j.eclinm.2023.101896

1434. McCann AL, Bonci L. Maintaining women’s oral health. *Dent Clin North Am*. 2001;45(3):571-601.

1435. McCloskey ML, Tarazona-Meza CE, Jones-Smith JC, et al. Disparities in dietary intake and physical activity patterns across the urbanization divide in the Peruvian Andes. *Int J Behav Nutr Phys Act*. 2017;14(1):90. doi:10.1186/s12966-017-0545-4

1436. MCCORMACK RM. STIFFNESS OF THE INJURED HAND: ANALYSIS, PREVENTION, AND TREATMENT. *J Trauma*. 1964;4:581-591. doi:10.1097/00005373-196409000-00003

1437. McCormick KM, Burns KL, Piccone CM, Gosselin LE, Brazeau GA. Effects of ovariectomy and estrogen on skeletal muscle function in growing rats. *J Muscle Res Cell Motil*. 2004;25(1):21-27. doi:10.1023/b:jure.0000021398.78327.39

1438. McCrindle BW, Zak V, Breitbart RE, et al. The relationship of patient medical and laboratory characteristics to changes in functional health status in children and adolescents after the Fontan procedure. *Pediatr Cardiol*. 2014;35(4):632-640. doi:10.1007/s00246-013-0831-0

1439. McDaniel DK, Ringel-Scaia VM, Morrison HA, et al. Pulmonary Exposure to Magneli Phase Titanium Suboxides Results in Significant Macrophage Abnormalities and Decreased Lung Function. *Front Immunol*. 2019;10. doi:10.3389/fimmu.2019.02714

1440. McDonnell AS, Strayer DL. The influence of a walk in nature on human resting brain activity: a randomized controlled trial. *Sci Rep*. 2024;14(1):27253. doi:10.1038/s41598-024-78508-x

1441. McElfish PA, Rowland B, Scott AJ, et al. Examining the Relationship Between Physical Activity and Self-Efficacy for Exercise Among Overweight and Obese Marshallese Adults. *J Immigr Minor Heal*. 2022;24(2):461-468. doi:10.1007/s10903-021-01194-8

1442. McElfish PA, Long CR, Kaholokula JK, et al. Design of a comparative effectiveness randomized controlled trial testing a faith-based Diabetes Prevention Program (WORD DPP) vs. a Pacific culturally adapted Diabetes Prevention Program (PILI DPP) for Marshallese in the United States. *Medicine (Baltimore)*. 2018;97(19):e0677. doi:10.1097/MD.0000000000010677

1443. McElroy K, MI A, PG H, MH D, DN H, MC R. Exercise stress testing for the pediatric patient with burns: a preliminary report. *J Burn Care Rehabil*. 1992;13(2, Part 1):236-238. https://search.ebscohost.com/login.aspx?direct=true&AuthType=cookie,ip,shib,uid&db=cul&AN=107486396&site=ehost-live&scope=site

1444. McEntire SJ, Herndon DN, Sanford AP, Suman OE. Thermoregulation during exercise in severely burned children. *Pediatr Rehabil*. 2006;9(1):57-64. doi:10.1080/13638490500074576

1445. McGahan JH, Mangan S, Collins K, Burns C, Gabbett T, O’Neill C. Match-Play Running Demands and Technical Performance Among Elite Gaelic Footballers: Does Divisional Status Count? *J strength Cond Res*. 2021;35(1):169-175. doi:10.1519/JSC.0000000000002450

1446. McGahan J, Burns C, Lacey S, Gabbett T, O’Neil C. Investigation in to the Positional Running Demands of Elite Gaelic Football Players: How Competition Data Can Inform Training Practice. *J strength Cond Res*. 2020;34(7):2040-2047. doi:10.1519/JSC.0000000000002492

1447. McGarry S, Elliott C, McDonald A, Valentine J, Wood F, Girdler S. Paediatric burns: from the voice of the child. *Burns*. 2014;40(4):606-615. doi:10.1016/j.burns.2013.08.031

1448. McGarry S, Girdler S, McDonald A, Valentine J, Wood F, Elliott C. Paediatric medical trauma: The impact on parents of burn survivors. *Burns*. 2013;39(6):1114-1121. doi:10.1016/j.burns.2013.01.009

1449. McGuire AP, Howard BAN, Burns C, Zambrano-Vazquez L, Szabo YZ. Biopsychosocial Correlates and Individual Differences for Eliciting Moral Elevation in Veterans With PTSD: An Experimental Case Series. *J Nerv Ment Dis*. 2024;212(1):33-42. doi:10.1097/NMD.0000000000001725

1450. McHargue C, Aden J, Pham TN, Salinas J, Rizzo JA. Precursors to oliguria during major burn resuscitation: An analysis of a prospective observational trial at 5 major US burn centers. *Burns*. 2024;50(6):1513-1518. doi:10.1016/j.burns.2024.03.007

1451. McHugh CM, Harron M, Kilcullen A, et al. The Experience of the Management of Eating Disorders in a Pop-up Eating Disorder Unit. *Ir Med J*. 2018;111(8):806.

1452. McKay MJ, Baldwin JN, Ferreira P, Simic M, Vanicek N, Burns J. Normative reference values for strength and flexibility of 1,000 children and adults. *Neurology*. 2017;88(1):36-43. doi:10.1212/WNL.0000000000003466

1453. McKay MJ, Baldwin JN, Ferreira P, Simic M, Vanicek N, Burns J. Reference values for developing responsive functional outcome measures across the lifespan. *Neurology*. 2017;88(16):1512-1519. doi:10.1212/WNL.0000000000003847

1454. McKay MJ, Baldwin JN, Ferreira P, et al. 1000 Norms Project: protocol of a cross-sectional study cataloging human variation. *Physiotherapy*. 2016;102(1):50-56. doi:10.1016/j.physio.2014.12.002

1455. McKay MJ, Baldwin JN, Ferreira P, et al. Spatiotemporal and plantar pressure patterns of 1000 healthy individuals aged 3-101 years. *Gait Posture*. 2017;58:78-87. doi:10.1016/j.gaitpost.2017.07.004

1456. McKelvie RS, Yusuf S, Pericak D, et al. Comparison of candesartan, enalapril, and their combination in congestive heart failure: randomized evaluation of strategies for left ventricular dysfunction (RESOLVD) pilot study. The RESOLVD Pilot Study Investigators. *Circulation*. 1999;100(10):1056-1064. doi:10.1161/01.cir.100.10.1056

1457. McKenna ZJ, Moralez G, Romero SA, et al. Cardiac remodeling in well-healed burn survivors after 6 months of unsupervised progressive exercise training. *J Appl Physiol*. 2023;134(2):405-414. doi:10.1152/japplphysiol.00630.2022

1458. McMullan II, Bunting BP, Burns A, et al. Is Physical Activity Associated With Loneliness or Social Isolation in Older Adults? Results of a Longitudinal Analysis Using the Irish Longitudinal Study on Ageing. *J Aging Phys Act*. 2021;29(4):562-572. doi:10.1123/japa.2020-0159

1459. McQuoid J, Regan T, Devkota J, et al. Situations and roles of cannabis versus cigarette use: Integrating ecological momentary assessment with qualitative mapping interviews. *Health Place*. 2024;89:103314. doi:10.1016/j.healthplace.2024.103314

1460. Mehrabi V. [Correction of burn scars (author’s transl)]. *Z Kinderchir Grenzgeb*. 1980;30 Suppl:146-148.

1461. Mehrani M, Nematollahi A, Hatami M, Hosseini K. Coronary artery dissection in a 33-year-oldman with fatigue and episodic retrosternal burning: a case report. *Eur Hear JOURNAL-CASE REPORTS*. 2018;2(3). doi:10.1093/ehjcr/yty068

1462. Meister V, Schulz H, Greving I, Imhoff M, Walter LD, May B. [Perforation of the esophagus after esophageal manometry]. *Dtsch Med Wochenschr*. 1997;122(46):1410-1414. doi:10.1055/s-2008-1047779

1463. Melchert-McKearnan K, Deitz J, JM E, White O. Children with burn injuries: purposeful activity versus rote exercise. *Am J Occup Ther*. 2000;54(4):381-390. doi:10.5014/ajot.54.4.381

1464. Mellor DJ, Burns M. Using the Five Domains Model to develop welfare assessment guidelines for Thoroughbred horses in New Zealand. *N Z Vet J*. 2020;68(3):150-156. doi:10.1080/00480169.2020.1715900

1465. Mellor SG, Cooper GJ. Analysis of 828 servicemen killed or injured by explosion in Northern Ireland 1970-84: the Hostile Action Casualty System. *Br J Surg*. 1989;76(10):1006-1010. doi:10.1002/bjs.1800761006

1466. Menon JE, Stensel DJ, Tolfrey K, Burns SF. Increased Meal Frequency With Exercise Mitigates Postprandial Triacylglycerol. *J Phys Act Health*. 2019;16(8):589-594. doi:10.1123/jpah.2018-0696

1467. Meroz M, Samberg Y. Disinfecting poultry production premises. *Rev Sci Tech*. 1995;14(2):273-291. doi:10.20506/rst.14.2.839

1468. Merritt EK. Why is it so hard to lose fat? Because it has to get out through your nose! An exercise physiology laboratory on oxygen consumption, metabolism, and weight loss. *Adv Physiol Educ*. 2021;45(3):599-606. doi:10.1152/advan.00006.2021

1469. Merz CN, Paul-Labrador M, Vongvanich P. Time to reevaluate risk stratification guidelines for medically supervised exercise training in patients with coronary artery disease. *JAMA*. 2000;283(11):1476-1478. doi:10.1001/jama.283.11.1476

1470. Messier SP, Royer TD, Craven TE, O’Toole ML, Burns R, Ettinger WHJ. Long-term exercise and its effect on balance in older, osteoarthritic adults: results from the Fitness, Arthritis, and Seniors Trial (FAST). *J Am Geriatr Soc*. 2000;48(2):131-138. doi:10.1111/j.1532-5415.2000.tb03903.x

1471. MF G, JC J, Browning S, et al. Admissions across state lines: harnessing the insight of the National Burn Repository for the healthcare accessibility, fiscal, and legislative concerns facing the American Burn Association. *J Burn Care Res*. 2008;29(1):151-157. doi:10.1097/BCR.0b013e31815fa480

1472. MH C, Dutcher K, JA M, et al. Efficacy of continuous passive motion (CPM) devices with hand burns. *J Burn Care Rehabil*. 1988;9(4):397-400. https://search.ebscohost.com/login.aspx?direct=true&AuthType=cookie,ip,shib,uid&db=cul&AN=107529405&site=ehost-live&scope=site

1473. Michiels JJ, van Joost T, Vuzevski VD. Idiopathic erythermalgia: a congenital disorder. *J Am Acad Dermatol*. 1989;21(5 Pt 2):1128-1130. doi:10.1016/s0190-9622(89)70313-3

1474. Michiels JJ, te Morsche RHM, Jansen JBMJ, Drenth JPH. Autosomal dominant erythermalgia associated with a novel mutation in the voltage-gated sodium channel alpha subunit Nav1.7. *Arch Neurol*. 2005;62(10):1587-1590. doi:10.1001/archneur.62.10.1587

1475. Michishita T, Kobayashi S, Katsuya T, Ogihara T, Kawabuchi K. Evaluation of the antiobesity effects of an amino acid mixture and conjugated linoleic acid on exercising healthy overweight humans: a randomized, double-blind, placebo-controlled trial. *J Int Med Res*. 2010;38(3 CC-Metabolic and Endocrine Disorders):844‐859. doi:10.1177/147323001003800311

1476. Milberger S, Davis RM, Douglas CE, et al. Tobacco manufacturers’ defence against plaintiffs’ claims of cancer causation: throwing mud at the wall and hoping some of it will stick. *Tob Control*. 2006;15 Suppl 4(Suppl 4):iv17-26. doi:10.1136/tc.2006.016956

1477. Milburn N, Yap L, Raval G. Heterotrophic Ossification in Patient With Prosthetic Leg. *Clin J Sport Med*. 2020;30(1):e18-e19. doi:10.1097/JSM.0000000000000701

1478. Miles W. Soft tissue trauma. *Hand Clin*. 1986;2(1):33-43.

1479. Miller JT, Btaiche IF. Oxandrolone in pediatric patients with severe thermal burn injury. *Ann Pharmacother*. 2008;42(9):1310-1315. doi:10.1345/aph.1L162

1480. Miller JT, Btaiche IF. Oxandrolone treatment in adults with severe thermal injury. *Pharmacotherapy*. 2009;29(2):213-226. doi:10.1592/phco.29.2.213

1481. Miller ME, Kral JG. Surgery for obesity in older women. *Menopause Int*. 2008;14(4):155-162. doi:10.1258/mi.2008.008028

1482. Mills DA, Chu AS, Burns A, et al. Dental Bite-Sized Bits: A Module for Teaching Common Oral Health Conditions to Multidisciplinary Students. *Med Sci Educ*. 2023;33(2):451-458. doi:10.1007/s40670-023-01760-9

1483. Milne SC, Corben LA, Roberts M, et al. Rehabilitation for ataxia study: protocol for a randomised controlled trial of an outpatient and supported home-based physiotherapy programme for people with hereditary cerebellar ataxia. *BMJ Open*. 2020;10(12):e040230. doi:10.1136/bmjopen-2020-040230

1484. Milne SC, Roberts M, Williams S, et al. Goal-Directed Rehabilitation Versus Standard Care for Individuals with Hereditary Cerebellar Ataxia: A Multicenter, Single-Blind, Randomized Controlled Superiority Trial. *Ann Neurol*. Published online November 2024. doi:10.1002/ana.27130

1485. Milne-Ives M, Swancutt D, Burns L, et al. The Effectiveness and Usability of Online, Group-Based Interventions for People With Severe Obesity: Protocol for a Systematic Review. *JMIR Res Protoc*. 2021;10(6):e26619. doi:10.2196/26619

1486. Mina WC, Burns RW, Terry BE. The treatment of obesity. *Mo Med*. 2003;100(3):248-255.

1487. Minaglia S, Liegl M. Moonless night sky increases Isistius species (cookiecutter shark) and live human contact. *PLoS One*. 2024;19(2):e0291852. doi:10.1371/journal.pone.0291852

1488. MINTKEN PE, MCDEVITT AMYW, CLELAND JA, et al. Cervicothoracic Manual Therapy Plus Exercise Therapy Versus Exercise Therapy Alone in the Management of Individuals With Shoulder Pain: A Multicenter Randomized Controlled Trial. *J Orthop Sport Phys Ther*. 2016;46(8):617-628. doi:10.2519/jospt.2016.6319

1489. MINTKEN PE, MCDEVITT AMYW, MICHENER LA, et al. Examination of the Validity of a Clinical Prediction Rule to Identify Patients With Shoulder Pain Likely to Benefit From Cervicothoracic Manipulation. *J Orthop Sport Phys Ther*. 2017;47(4):252-260. doi:10.2519/jospt.2017.7100

1490. Mintz EM, George DE, Hsu S. Silver sulfadiazine therapy in widespread bullous disorders: potential for toxicity. *Dermatol Online J*. 2008;14(3):19.

1491. Miri S, Hosseini SJ, Ghorbani Vajargah P, et al. Effects of massage therapy on pain and anxiety intensity in patients with burns: A systematic review and meta-analysis. *Int Wound J*. 2023;20(6):2440-2458. doi:10.1111/iwj.14089

1492. Miri S, Hosseini SJ, Takasi P, et al. Effects of breathing exercise techniques on the pain and anxiety of burn patients: A systematic review and meta‐analysis. *Int Wound J*. 2023;20(6):2360-2375. doi:10.1111/iwj.14057

1493. Miri S, Mobayen M, Aboutaleb E, Ezzati K, Feizkhah A, Karkhah S. Exercise as a rehabilitation intervention for severe burn survivors: Benefits & barriers. *Burn*. 2022;48(5):1269-1270. doi:10.1016/j.burns.2022.04.016

1494. Miri S, Mobayen M, Mazloum SMH, et al. The role of a structured rehabilitative exercise program as a safe and effective strategy for restoring the physiological function of burn survivors. *Burn*. 2022;48(6):1521-1523. doi:10.1016/j.burns.2022.06.008

1495. Miri S, Rashtiani S, Zabihi MR, Akhoondian M, Farzan R. Role of exercise in nursing care for burn wound patients: A narrative review from a nursing perspective. *J Nurs Reports Clin Pract*. 2024;0(0):0-0. doi:10.32598/jnrcp.23.101

1496. Mishra SR, Dempsey W, Klasnja P. A Text Messaging Intervention for Priming the Affective Rewards of Exercise in Adults: Protocol for a Microrandomized Trial. *JMIR Res Protoc*. 2023;12:e46560. doi:10.2196/46560

1497. Misra P. AMP activated protein kinase: a next generation target for total metabolic control. *Expert Opin Ther Targets*. 2008;12(1):91-100. doi:10.1517/14728222.12.1.91

1498. Mistry J, Falla D, Noblet T, Heneghan NR, Rushton A. Clinical indicators to identify neuropathic pain in low back related leg pain: a modified Delphi study. *BMC Musculoskelet Disord*. 2020;21(1). doi:10.1186/s12891-020-03600-y

1499. Mistry R, Wickramasingha N, Ogston S, Singh M, Devasiri V, Mukhopadhyay S. Wheeze and urban variation in South Asia. *Eur J Pediatr*. 2004;163(3):145-147. doi:10.1007/s00431-003-1393-6

1500. Mitchell UH, Johnson AW, Myrer JW, Hager RL, Eggett DL. A randomized single-blind controlled trial comparing two monochromatic near-infrared light devices: implications for tissue heating and safety. *Am J Phys Med Rehabil*. 2012;91(9):789-796. doi:10.1097/PHM.0b013e31825a1937

1501. Miyashita M, Edamoto K, Kidokoro T, et al. Interrupting Sitting Time with Regular Walks Attenuates Postprandial Triglycerides. *Int J Sports Med*. 2016;37(2):97-103. doi:10.1055/s-0035-1559791

1502. Miyashita M, Hamada Y, Fujihira K, et al. Energy replacement diminishes the postprandial triglyceride-lowering effect from accumulated walking in older women. *Eur J Nutr*. 2020;59(5):2261-2270. doi:10.1007/s00394-020-02234-z

1503. Miyashita M, Park JH, Takahashi M, et al. Physical activity status and postprandial lipaemia in older adults. *Int J Sports Med*. 2011;32(11):829-834. doi:10.1055/s-0031-1279770

1504. Miyashita M, SF B, DJ S. Exercise and postprandial lipemia: effect of continuous compared with intermittent activity patterns. *Am J Clin Nutr*. 2006;83(1):24-29. doi:10.1093/ajcn/83.1.24

1505. Miyashita M, SF B, DJ S. Acute effects of accumulating exercise on postprandial lipemia and C-reactive protein concentrations in young men. *Int J Sport Nutr Exerc Metab*. 2009;19(6):569-582. doi:10.1123/ijsnem.19.6.569

1506. Miyashita M, Takahashi M, Burns S. Increased participation in weekend physical activity reduces postprandial lipemia in postmenopausal women. *Int J Sports Med*. 2014;35(13):1059-1064. doi:10.1055/s-0034-1372641

1507. Miyashita M, Burns SF, Stensel DJ. Accumulating short bouts of brisk walking reduces postprandial plasma triacylglycerol concentrations and resting blood pressure in healthy young men. *Am J Clin Nutr*. 2008;88(5):1225-1231. doi:10.3945/ajcn.2008.26493

1508. Miyashita M, Burns SF, Stensel DJ. Accumulating short bouts of running reduces resting blood pressure in young normotensive/pre-hypertensive men. *J Sports Sci*. 2011;29(14):1473-1482. doi:10.1080/02640414.2011.593042

1509. Miyashita M, Burns SF, Stensel DJ. An update on accumulating exercise and postprandial lipaemia: translating theory into practice. *J Prev Med Public Heal*. 2013;46:S3-S11. doi:10.3961/jpmph.2013.46.S.S3

1510. Miyashita M, Hamada Y, Fujihira K, et al. Correction to: Energy replacement diminishes the postprandial triglyceride‑lowering effect from accumulated walking in older women. *Eur J Nutr*. 2020;59(5):2271-2272. doi:10.1007/s00394-020-02263-8

1511. Miyashita M, Stensel DJ, Burns SF. Effect of exercise timing on postprandial lipaemia. *J Atheroscler Thromb*. 2012;19(2):205-206. doi:10.5551/jat.11379

1512. Miyashita M, Stensel DJ, Burns SF, Sasai H, Tanaka K. The effects of 30 min of exercise on cardiovascular disease risk factors in healthy and obese individuals. *Atherosclerosis*. 2011;216(2):496-497. doi:10.1016/j.atherosclerosis.2011.02.001

1513. Mkumbuzi NS, September A V, Posthumus M, Oulo B, Mafu TS, Collins M. Characterisation of Achilles tendon pain in recreational runners using multidimensional pain scales. *J Sci Med Sport*. 2020;23(3):258-263. doi:10.1016/j.jsams.2019.10.016

1514. Mlcak RP, Desai MH, Robinson E, McCauley RL, Richardson J, Herndon DN. Increased physiological dead space/tidal volume ratio during exercise in burned children. *Burns*. 1995;21(5):337-339. doi:10.1016/0305-4179(94)00017-4

1515. Mlcak RP, Desai MH, Robinson E, McCauley RL, Robson MC, Herndon DN. Temperature changes during exercise stress testing in children with burns. *J Burn Care Rehabil*. 1993;14(4):427-430. doi:10.1097/00004630-199307000-00004

1516. Möckel F, Hoffmann G, Obermüller R, Drobnik W, Schmitz G. Influence of water-filtered infrared-A (wIRA) on reduction of local fat and body weight by physical exercise. *Ger Med Sci*. 2006;4:Doc05.

1517. Moehrle M. Outdoor sports and skin cancer. *Clin Dermatol*. 2008;26(1):12-15. doi:10.1016/j.clindermatol.2007.10.001

1518. Mohaddes Ardebili F, Manzari ZS, Bozorgnejad M. Effect of educational program based on exercise therapy on burned hand function. *World J Plast Surg*. 2014;3(1):39-46.

1519. Mohamed Muftah Alzaabi FS, Bairapareddy KC, Alaparthi GK, Hegazy F. Caregiver Perspectives on Physiotherapy Treatment for Paediatric Burns in the United Arab Emirates. *Patient Prefer Adherence*. 2022;16:1477-1486. doi:10.2147/PPA.S363312

1520. Mohammed J, Smith SR, Burns L, et al. Role of Physical Therapy before and after Hematopoietic Stem Cell Transplantation: White Paper Report. *Biol blood marrow Transplant J Am Soc Blood Marrow Transplant*. 2019;25(6):e191-e198. doi:10.1016/j.bbmt.2019.01.018

1521. Moiemen N, Mathers J, Jones L, et al. Pressure garment to prevent abnormal scarring after burn injury in adults and children: the PEGASUS feasibility RCT and mixed-methods study. *Health Technol Assess*. 2018;22(36 CC-Wounds):1‐162. doi:10.3310/hta22360

1522. Moin T, Damschroder LJ, AuYoung M, et al. Diabetes Prevention Program Translation in the Veterans Health Administration. *Am J Prev Med*. 2017;53(1):70-77. doi:10.1016/j.amepre.2016.11.009

1523. Mologne MS, Hu J, Carrillo E, et al. The Efficacy of an Immersive Virtual Reality Exergame Incorporating an Adaptive Cable Resistance System on Fitness and Cardiometabolic Measures: A 12-Week Randomized Controlled Trial. *Int J Environ Res Public Health*. 2022;20(1). doi:10.3390/ijerph20010210

1524. Monteiro SM, Jancey J, Howat P, et al. The protocol of a randomized controlled trial for playgroup mothers: Reminder on Food, Relaxation, Exercise, and Support for Health (REFRESH) Program. *BMC Public Health*. 2011;11. doi:10.1186/1471-2458-11-648

1525. Monteiro SMDR, Jancey J, Dhaliwal SS, et al. Results of a randomized controlled trial to promote physical activity behaviours in mothers with young children. *Prev Med (Baltim)*. 2014;59:12-18. doi:10.1016/j.ypmed.2013.10.022

1526. Montorsi F, Scuderi S, Briganti A, Gandaglia G. Re: Kerrington Powell, Michael C. Burns, Vinay Prasad. Relugolix: Five Reasons Why the US Food and Drug Administration Should Have Exercised Restraint. Eur Urol. 2023;83:101-2. *Eur Urol*. 2023;83(5):136. doi:10.1016/j.eururo.2023.01.025

1527. Moore FA, Phillips SM, McClain CJ, Patel JJ, Martindale RG. Nutrition Support for Persistent Inflammation, Immunosuppression, and Catabolism Syndrome. *Nutr Clin Pract*. 2017;32:121S-127S. doi:10.1177/0884533616687502

1528. Moore J, Morath K, Harré N. Follow-up study of a school-based scalds prevention programme. *Health Educ Res*. 2004;19(4):430-439. doi:10.1093/her/cyg047

1529. Morales Cardona T. [Establishment in Mayagüez of a coal-based thermoelectric plant: perspectives for human health]. *Bol Asoc Med P R*. 1991;83(7):285-291.

1530. Moreno-Ruiz JA, García-Lazaro JR, Arbelo M, Cantón-Garbín M. MODIS Sensor Capability to Burned Area Mapping-Assessment of Performance and Improvements Provided by the Latest Standard Products in Boreal Regions. *SENSORS*. 2020;20(18). doi:10.3390/s20185423

1531. Morgans R, Bezuglov E, Orme P, et al. The Physical Demands of Match-Play in Academy and Senior Soccer Players from the Scottish Premiership. *SPORTS*. 2022;10(10). doi:10.3390/sports10100150

1532. Morris JK, Honea RA, Vidoni ED, Swerdlow RH, Burns JM. Is Alzheimer’s disease a systemic disease? *Biochim Biophys Acta*. 2014;1842(9):1340-1349. doi:10.1016/j.bbadis.2014.04.012

1533. Morris JK, Vidoni ED, Johnson DK, et al. Aerobic exercise for Alzheimer’s disease: A randomized controlled pilot trial. *PLoS One*. 2017;12(2):e0170547. doi:10.1371/journal.pone.0170547

1534. Morris JG, Barrett LA, Burns SF, Gorely T. Editorial: School based physical activity: Can it work? *Front Sport Act living*. 2022;4:1107274. doi:10.3389/fspor.2022.1107274

1535. Morris LD, Louw QA, Crous LC. Feasibility and potential effect of a low-cost virtual reality system on reducing pain and anxiety in adult burn injury patients during physiotherapy in a developing country. *Burns*. 2010;36(5):659-664. doi:10.1016/j.burns.2009.09.005

1536. Moskowitz RM, Burns JJ, DiCarlo EF, et al. Cage size and exercise affects infarct size in rat after coronary artery cauterization. *J Appl Physiol*. 1979;47(2):393-396. doi:10.1152/jappl.1979.47.2.393

1537. Moulton AL, Brenner JI, Roberts G, et al. Subclavian flap repair of coarctation of the aorta in neonates. Realization of growth potential? *J Thorac Cardiovasc Surg*. 1984;87(2):220-235.

1538. Moynahan M, Mullin C, Cohn J, et al. Home use of a functional electrical stimulation system for standing and mobility in adolescents with spinal cord injury. *Arch Phys Med Rehabil*. 1996;77(10):1005-1013. doi:10.1016/s0003-9993(96)90060-x

1539. Mudawarima T, Chiwaridzo M, Jelsma J, Grimmer K, Muchemwa FC. A systematic review protocol on the effectiveness of therapeutic exercises utilised by physiotherapists to improve function in patients with burns. *Syst Rev*. 2017;6(1):207. doi:10.1186/s13643-017-0592-6

1540. Muehlberger T, MA S, Wong L, Muehlberger T, Smith MA, Wong L. Domiciliary oxygen and smoking: an explosive combination. *Burn*. 1998;24(7):658-660. https://search.ebscohost.com/login.aspx?direct=true&AuthType=cookie,ip,shib,uid&db=cul&AN=107044137&site=ehost-live&scope=site

1541. Mukhopadhyay R, Sambandam S, Pillarisetti A, et al. Cooking practices, air quality, and the acceptability of advanced cookstoves in Haryana, India: an exploratory study to inform large-scale interventions. *Glob Health Action*. 2012;5:1-13. doi:10.3402/gha.v5i0.19016

1542. Mulder MB, Sussman MS, Eidelson SA, et al. Heart Rate Complexity in US Army Forward Surgical Teams During Pre Deployment Training. *Mil Med*. 2020;185(5-6):e724-e733. doi:10.1093/milmed/usz434

1543. Mulder RL, Font-Gonzalez A, van Dulmen-den Broeder E, et al. Communication and ethical considerations for fertility preservation for patients with childhood, adolescent, and young adult cancer: recommendations from the PanCareLIFE Consortium and the International Late Effects of Childhood Cancer Guideline Harmoniz. *Lancet Oncol*. 2021;22(2):e68-e80. doi:10.1016/S1470-2045(20)30595-7

1544. Müller M, Kunz M, Schwarz L, Kindermann W, Buchter A. [Burning pain in calves after long-term pollutant exposure. Suspected toxicity is misleading]. *MMW Fortschr Med*. 2006;148(18):12.

1545. Muller MD, Seo Y, Kim CH, et al. Cold habituation does not improve manual dexterity during rest and exercise in 5 °C. *Int J Biometeorol*. 2014;58(3):383-394. doi:10.1007/s00484-013-0633-3

1546. Mulvagh SL, DeMaria AN, Feinstein SB, et al. Contrast echocardiography: current and future applications. *J Am Soc Echocardiogr*. 2000;13(4):331-342. https://search.ebscohost.com/login.aspx?direct=true&AuthType=cookie,ip,shib,uid&db=cul&AN=107113687&site=ehost-live&scope=site

1547. Murphy IG, Murphy CG, Heffernan EJ. A comparative analysis of the occupational energy expenditure of radiologists versus clinicians. *Ir J Med Sci*. 2015;184(4):889-892. doi:10.1007/s11845-014-1215-z

1548. Murphy LB, Santos-Ledo A, Dhanaseelan T, et al. Exercise, programmed cell death and exhaustion of cardiomyocyte proliferation in aging zebrafish. *Dis Model Mech*. 2021;14(7). doi:10.1242/dmm.049013

1549. Murphy NJ, Diamond LE, Bennell KL, et al. Which hip morphology measures and patient factors are associated with age of onset and symptom severity in femoroacetabular impingement syndrome? *HIP Int*. 2023;33(1):102-111. doi:10.1177/11207000211038550

1550. Murphy NJ, Eyles J, Bennell KL, et al. Protocol for a multi-centre randomised controlled trial comparing arthroscopic hip surgery to physiotherapy-led care for femoroacetabular impingement (FAI): the Australian FASHIoN trial. *BMC Musculoskelet Disord*. 2017;18(1):406. doi:10.1186/s12891-017-1767-y

1551. Murphy NJ, Eyles J, Spiers L, et al. Moderators, Mediators, and Prognostic Indicators of Treatment With Hip Arthroscopy or Physical Therapy for Femoroacetabular Impingement Syndrome: Secondary Analyses From the Australian FASHIoN Trial. *Am J Sports Med*. 2023;51(1):141-154. doi:10.1177/03635465221136547

1552. Murray BP, Ralston SA, Dunkley CA, Carpenter JE, Geller RJ, Kazzi Z. Pneumonitis and Respiratory Failure Secondary to Civilian Exposure to a Smoke Bomb in a Partially Enclosed Space. *J Spec Oper Med a peer Rev J SOF Med Prof*. 2018;18(4):24-26. doi:10.55460/UD9X-AUXA

1553. Murray C, Burns N. Wearing a Fitbit Increases Physical Activity Levels in Women with Gestational Diabetes. *Ir Med J*. 2024;117(3):924.

1554. Nadler SF, Prybicien M, Malanga GA, Sicher D. Complications from therapeutic modalities: results of a national survey of athletic trainers. *Arch Phys Med Rehabil*. 2003;84(6):849-853. doi:10.1016/s0003-9993(02)04955-9

1555. Nagayama C, Burns SF, Stensel DJ, Thackray AE, Takahashi M, Miyashita M. Effects of a single bout of walking on postprandial triglycerides in men of Chinese, European and Japanese descent: a multisite randomised crossover trial. *BMJ OPEN Sport Exerc Med*. 2020;6(1). doi:10.1136/bmjsem-2020-000928

1556. Nagayama C, Burns SF, Thackray AE, Stensel DJ, Miyashita M. Postprandial Metabolism and Physical Activity in Asians: A Narrative Review. *Int J Sports Med*. 2021;42(11):953-966. doi:10.1055/a-1493-2948

1557. Nahabedian MY, Hammer J. Use of Magnetic Resonance Imaging in Patients with Breast Tissue Expanders. *Plast Reconstr Surg*. 2022;150(5):963-968. doi:10.1097/PRS.0000000000009614

1558. Nahar VK, Ford MA, Boyas JF, et al. Skin cancer preventative behaviors in state park workers: a pilot study. *Environ Health Prev Med*. 2014;19(6):467-474. doi:10.1007/s12199-014-0412-8

1559. Najafi B, Barnica E, Wrobel JS, Burns J. Dynamic plantar loading index: understanding the benefit of custom foot orthoses for painful pes cavus. *J Biomech*. 2012;45(9):1705-1711. doi:10.1016/j.jbiomech.2012.03.006

1560. Nakamura A, Matsumura T, Takeshima Y, et al. The Association Between Physical Activity/Heart Rate Variability Data Obtained Using a Wearable Device and Timed Motor Functional Tests in Patients with Duchenne Muscular Dystrophy: A Pilot Study. *J Neuromuscul Dis*. 2024;11(3):715-724. doi:10.3233/JND-230142

1561. Nam K, Ringenbach SDR, Brusseau TA, et al. Immediate reinforcement increased duration of time riding the stationary bicycle in children with autism spectrum disorder: a pilot study. *Int J Dev Disabil*. 2022;68(3):388-394. doi:10.1080/20473869.2020.1783480

1562. Nambi G, Abdelbasset WK. Efficacy of Maitland joint mobilization technique on pain intensity, mouth opening, functional limitation, kinesiophobia, sleep quality and quality of life in temporomandibular joint dysfunction following bilateral cervicofacial burns. *Burns*. Published online 2020. https://www.cochranelibrary.com/central/doi/10.1002/central/CN-02130090/full

1563. Nambi G, Abdelbasset WK, Elshehawy AA, et al. Yoga in Burn: Role of pranayama breathing exercise on pulmonary function, respiratory muscle activity and exercise tolerance in full-thickness circumferential burns of the chest. *Burns*. 2021;47(1):206-214. doi:10.1016/j.burns.2020.06.033

1564. Nambi G, Alghadier M, Ebrahim EE, et al. Role of virtual reality distraction technique to improve chest burns with acute respiratory distress syndrome (ARDS) following smoke inhalation in middle-aged adults - A randomized controlled study. *Burns*. 2023;49(7):1643-1653. doi:10.1016/j.burns.2023.05.017

1565. Nash MS, Meltzer NM, Martins SC, Burns PA, Lindley SD, Field-Fote EC. Nutrient supplementation post ambulation in persons with incomplete spinal cord injuries: a randomized, double-blinded, placebo-controlled case series. *Arch Phys Med Rehabil*. 2007;88(2):228-233. doi:10.1016/j.apmr.2006.11.012

1566. Nash M, Cartwright K, Nguyen R, Middleton P, Maitz P. Surgical release of the chest wall skin and fascia for sclerodermatous graft versus host disease causing restrictive lung disease: A case report. *Int J Surg Case Rep*. 2024;117:109455. doi:10.1016/j.ijscr.2024.109455

1567. Natale VM, Brenner IK, Moldoveanu AI, Vasiliou P, Shek P, Shephard RJ. Effects of three different types of exercise on blood leukocyte count during and following exercise. *Sao Paulo Med J*. 2003;121(1):9‐14. doi:10.1590/s1516-31802003000100003

1568. Nathan A, Rose JB, Guite JW, Hehir D, Milovcich K. Primary erythromelalgia in a child responding to intravenous lidocaine and oral mexiletine treatment. *Pediatrics*. 2005;115(4):e504-7. doi:10.1542/peds.2004-1395

1569. Nathanson AT, Young JMJ, Young C. Pre-Participation Medical Evaluation for Adventure and Wilderness Watersports. *Wilderness Environ Med*. 2015;26(4 Suppl):S55-62. doi:10.1016/j.wem.2015.09.008

1570. Naylor WP, Manor RC. Fabrication of a flexible prosthesis for the edentulous scleroderma patient with microstomia. *J Prosthet Dent*. 1983;50(4):536-538. doi:10.1016/0022-3913(83)90577-2

1571. Nazeran H, Chatlapalli S, Krishnam R. Effect of Novel Nanoscale Energy Patches on Spectral and Nonlinear Dynamic Features of Heart Rate Variability Signals in Healthy Individuals during Rest and Exercise. *Conf Proc . Annu Int Conf IEEE Eng Med Biol Soc IEEE Eng Med Biol Soc Annu Conf*. 2005;2005:5563-5567. doi:10.1109/IEMBS.2005.1615745

1572. Nedelec B, Parry I, Acharya H, et al. Practice Guidelines for Cardiovascular Fitness and Strengthening Exercise Prescription After Burn Injury. *J Burn care Res Off Publ Am Burn Assoc*. 2016;37(6):e539-e558. doi:10.1097/BCR.0000000000000282

1573. Nedelec B, Serghiou MA, Niszczak J, McMahon M, Healey T. Practice guidelines for early ambulation of burn survivors after lower extremity grafts. *J Burn care Res Off Publ Am Burn Assoc*. 2012;33(3):319-329. doi:10.1097/BCR.0b013e31823359d9

1574. Nelson D, Harris A, Horner-Ibler B, Harris KS, Burns E. Hearing the Community: Evolution of a Nutrition and Physical Activity Program for African American Women to Improve Weight. *J Health Care Poor Underserved*. 2016;27(2):560-567. doi:10.1353/hpu.2016.0088

1575. Netzer NC, Chytra R, Kupper T. Low intense physical exercise in normobaric hypoxia leads to more weight loss in obese people than low intense physical exercise in normobaric sham hypoxia. *Schlaf & Atmung [Sleep & breathing]*. 2008;12(2):129‐134. doi:10.1007/s11325-007-0149-3

1576. Neugebauer CT, Serghiou M, Herndon DN, Suman OE. Effects of a 12-week rehabilitation program with music & exercise groups on range of motion in young children with severe burns. *J Burn care Res Off Publ Am Burn Assoc*. 2008;29(6):939-948. doi:10.1097/BCR.0b013e31818b9e0e

1577. Neville C, Walker S, Brown B, Bowens B, Dimick AR. Discharge planning for burn patients. *J Burn Care Rehabil*. 1988;9(4):414-420.

1578. Newman DK, Burns PA, DK N, PA B. New approaches for managing stress incontinence in women. *Lippincotts Prim Care Pract*. 1997;1(4):382-387. https://search.ebscohost.com/login.aspx?direct=true&AuthType=cookie,ip,shib,uid&db=cul&AN=107299470&site=ehost-live&scope=site

1579. Newsholme EA. Biochemical mechanisms to explain immunosuppression in well-trained and overtrained athletes. *Int J Sports Med*. 1994;15 Suppl 3:S142-7. doi:10.1055/s-2007-1021129

1580. Newsholme EA, Calder PC. The proposed role of glutamine in some cells of the immune system and speculative consequences for the whole animal. *Nutrition*. 1997;13(7-8):728-730. doi:10.1016/s0899-9007(97)83034-1

1581. Newsholme EA, Calder P, Yaqoob P. The regulatory, informational, and immunomodulatory roles of fat fuels. *Am J Clin Nutr*. 1993;57(5 Suppl):738S-750S; discussion 750S-751S. doi:10.1093/ajcn/57.5.738S

1582. Newton N, Bubenickova M. Rehabilitation of the autografted hand in children with burns. *Phys Ther*. 1977;57(12):1383-1388. doi:10.1093/ptj/57.12.1383

1583. Nganabashaka JP, Ntawuyirushintege S, Niyibizi JB, et al. Population-Level Interventions Targeting Risk Factors for Hypertension and Diabetes in Rwanda: A Situational Analysis. *Front public Heal*. 2022;10:882033. doi:10.3389/fpubh.2022.882033

1584. Nguyen LA, Cazin M, Miles JD. Thyrotoxic Periodic Paralysis in a Samoan Male With Metabolic Acidosis: A Case Report and Review of the Literature. *Cureus*. 2024;16(7):e65309. doi:10.7759/cureus.65309

1585. Nguyen MN, Poupart G, Normandeau J, Laplante L, Damestoy N. [The habits and perceptions of participants in water and other outdoor activities in terms of risk behaviors]. *Can J Public Health*. 2002;93(3):208-212. doi:10.1007/BF03405002

1586. Nichani V, Dirks K, Burns B, Bird A, Grant C. Green space and depression during pregnancy: Results from the growing up in New Zealand study. *Int J Environ Res Public Health*. 2017;14(9). doi:10.3390/ijerph14091083

1587. Nichani V, Dirks K, Burns B, Bird A, Morton S, Grant C. Green space and physical activity in pregnant women: Evidence from the growing up in new zealand study. *J Phys Act Heal*. 2016;13(12):1341-1350. doi:10.1123/jpah.2016-0013

1588. Nichols AW. The thoracic outlet syndrome in athletes. *J Am Board Fam Pract*. 1996;9(5):346-355.

1589. Nichols AW. Nonorthopaedic problems in the aquatic athlete. *Clin Sports Med*. 1999;18(2):395-411, viii. doi:10.1016/s0278-5919(05)70154-x

1590. Nichols AW. Does eccentric training of hamstring muscles reduce acute injuries in soccer? *Clin J Sport Med Off J Can Acad Sport Med*. 2013;23(1):85-86. doi:10.1097/JSM.0b013e31827e9f40

1591. Nichols AW. Heat-related illness in sports and exercise. *Curr Rev Musculoskelet Med*. 2014;7(4):355-365. doi:10.1007/s12178-014-9240-0

1592. Nichols AW. Medical Care of the Aquatics Athlete. *Curr Sports Med Rep*. 2015;14(5):389-396. doi:10.1249/JSR.0000000000000194

1593. Nichols AW. Author Response. *Curr Sports Med Rep*. 2016;15(1):47. doi:10.1249/JSR.0000000000000225

1594. Nichols AW, Hetzler RK, Villanueva RJ, Stickley CD, Kimura IF. Effects of combination oral contraceptives on strength development in women athletes. *J strength Cond Res*. 2008;22(5):1625-1632. doi:10.1519/JSC.0b013e31817ae1f3

1595. Nickel R, Troncoso F, Flores O, et al. Physiological response to eccentric and concentric cycling in patients with chronic obstructive pulmonary disease. *Appl Physiol Nutr Metab*. 2020;45(11):1232-1237. doi:10.1139/apnm-2020-0149

1596. Nickerl U, Resag I. [Ergotherapy of severely burned patients]. *Unfallchirurg*. 1995;98(4):204-208.

1597. Nicolucci A, Kovacs Burns K, Holt RIG, et al. Diabetes Attitudes, Wishes and Needs second study (DAWN2^TM^): cross-national benchmarking of diabetes-related psychosocial outcomes for people with diabetes. *Diabet Med*. 2013;30(7):767-777. doi:10.1111/dme.12245

1598. Niedermeier W, Huber M, Fischer D, et al. Significance of saliva for the denture-wearing population. *Gerodontology*. 2000;17(2):104-118. doi:10.1111/j.1741-2358.2000.00104.x

1599. Nigg CR. There is more to stages of exercise than just exercise. *Exerc Sport Sci Rev*. 2005;33(1):32-35.

1600. Nigg CR, Long CR. A systematic review of single health behavior change interventions vs. multiple health behavior change interventions among older adults. *Transl Behav Med*. 2012;2(2):163-179. doi:10.1007/s13142-012-0130-y

1601. Nimrouzi M, Zarshenas MM. Holistic Approach to Functional Constipation: Perspective of Traditional Persian Medicine. *Chin J Integr Med*. 2019;25(11):867-872. doi:10.1007/s11655-015-2302-3

1602. Nishigaki Y, Bonilla E, Shanske S, Gaskin DA, DiMauro S, Hirano M. Exercise-induced muscle “burning,” fatigue, and hyper-CKemia: mtDNA T10010C mutation in tRNA(Gly). *Neurology*. 2002;58(8):1282-1285. doi:10.1212/wnl.58.8.1282

1603. Nishina H, Slomka PJ, Abidov A, et al. Combined supine and prone quantitative myocardial perfusion SPECT: method development and clinical validation in patients with no known coronary artery disease. *J Nucl Med*. 2006;47(1):51-58.

1604. Nitz J, Burns Y, Wuthapanich N, Jackson R. A study of repeated lateral pinch grip in myotonic dystrophy. *Physiother Res Int*. 1999;4(1):1-11. https://search.ebscohost.com/login.aspx?direct=true&AuthType=cookie,ip,shib,uid&db=cul&AN=107212035&site=ehost-live&scope=site

1605. Nitzschke S, Offodile AC, Cauley RP, et al. Long term mortality in critically ill burn survivors. *Burns*. 2017;43(6):1155-1162. doi:10.1016/j.burns.2017.05.010

1606. Niumanlan, Jingming Y, Hao Q, Farzan R, Alizadeh Otaghvar H. A systematic review of the exercise effects on burn wound healing. *Int Wound J*. 2024;21(3):e14482. doi:10.1111/iwj.14482

1607. Noffsinger DL, Johnson SR, Wheeler K, Shi J, Xiang H, Groner JI. Exercise Treadmills: A Cause of Significant Hand Burns in Young Children. *J Burn care Res Off Publ Am Burn Assoc*. 2017;38(4):215-219. doi:10.1097/BCR.0000000000000446

1608. Norisue Y, Onopa J, Kaneshiro M, Tokuda Y. Surfing as a risk factor for gastroesophageal reflux disease. *Clin J Sport Med Off J Can Acad Sport Med*. 2009;19(5):388-393. doi:10.1097/JSM.0b013e3181b8ef41

1609. Norton ID, Wang L, Levine SA, et al. Efficacy of colonic submucosal saline solution injection for the reduction of iatrogenic thermal injury. *Gastrointest Endosc*. 2002;56(1):95-99. doi:10.1067/mge.2002.125362

1610. Norton PJ, Burns JA, Hope DA, Bauer BK. Generalization of social anxiety to sporting and athletic situations: gender, sports involvement, and parental pressure. *Depress Anxiety*. 2000;12(4):193-202. doi:10.1002/1520-6394(2000)12:4<193::AID-DA2>3.0.CO;2-X

1611. Novelli B, Melandri D, Bertolotti G, Vidotto G. Quality of life impact as outcome in burns patients. *G Ital Med Lav Ergon*. 2009;31(1 Suppl A):A58-63.

1612. Nuotio J, Laitinen TT, Sinaiko AR, et al. Obesity during childhood is associated with higher cancer mortality rate during adulthood: the i3C Consortium. *Int J Obes*. 2022;46(2):393-399. doi:10.1038/s41366-021-01000-3

1613. Nyarku M, Mazaheri M, Jayaratne R, et al. Mobile phones as monitors of personal exposure to air pollution: Is this the future? *PLoS One*. 2018;13(2). doi:10.1371/journal.pone.0193150

1614. Nykamp D, Marshall LL, Ashworth L. An active-learning assignment using nonprescription medicines. *Am J Pharm Educ*. 2008;72(1):20. doi:10.5688/aj720120

1615. Obiajulu J, DeSoucy E, Robb D, Atkins M, Rush S. Lessons Learned From a Large-Scale Military Mass Casualty Incident: Reevaluation of the Response to the Khobar Towers Bombing. *Mil Med*. Published online June 2024. doi:10.1093/milmed/usae316

1616. O’Connor CM, Whellan DJ. Understanding heart failure through the HF-ACTION baseline characteristics. *Am Heart J*. 2009;158(4 Suppl):S1-5. doi:10.1016/j.ahj.2009.07.013

1617. O’Connor CM, Whellan DJ, Lee KL, et al. Efficacy and safety of exercise training in patients with chronic heart failure: HF-ACTION randomized controlled trial. *JAMA*. 2009;301(14):1439-1450. doi:10.1001/jama.2009.454

1618. O’Donnell C, Iino M, Mansharan K, Leditscke J, Woodford N. Contribution of postmortem multidetector CT scanning to identification of the deceased in a mass disaster: Experience gained from the 2009 Victorian bushfires. *Forensic Sci Int*. 2011;205(1-3):15-28. doi:10.1016/j.forsciint.2010.05.026

1619. O’Donnell CA, Mair FS, Dowrick C, et al. Supporting the use of theory in cross-country health services research: a participatory qualitative approach using Normalisation Process Theory as an example. *BMJ Open*. 2017;7(8):e014289. doi:10.1136/bmjopen-2016-014289

1620. O’Donoghue G, Kennedy A, Puggina A, et al. Socio-economic determinants of physical activity across the life course: A “DEterminants of DIet and Physical ACtivity” (DEDIPAC) umbrella literature review. *PLoS One*. 2018;13(1):e0190737. doi:10.1371/journal.pone.0190737

1621. O’Grady HK, Edbrooke L, Farley C, et al. The sit-to-stand test as a patient-centered functional outcome for critical care research: a pooled analysis of five international rehabilitation studies. *Crit Care*. 2022;26(1):175. doi:10.1186/s13054-022-04048-3

1622. Oh EJ, Kim JS, Heo SJ. Chemical Burn Injury on the Cornea Following Skin Preparation With Chlorhexidine in Tonsillectomy and Adenoidectomy. *J Craniofac Surg*. 2024;35(2):e107-e108. doi:10.1097/SCS.0000000000009755

1623. Oh JK, Park E, Kim B, et al. Awareness of and practice toward cancer prevention recommendations: results of the Korean National Cancer Prevention Awareness and Practice Survey in 2021. *Epidemiol Health*. 2022;44. doi:10.4178/epih.e2022068

1624. Olander EK, Eves FF, Puig-Ribera A. Promoting stair climbing: stair-riser banners are better than posters... sometimes. *Prev Med (Baltim)*. 2008;46(4):308-310. doi:10.1016/j.ypmed.2007.11.009

1625. Oldenkamp R, Hoeks S, Cengic M, et al. A High-Resolution Spatial Model to Predict Exposure to Pharmaceuticals in European Surface Waters: ePiE. *Environ Sci Technol*. 2018;52(21):12494-12503. doi:10.1021/acs.est.8b03862

1626. Oleĭnik SD. [Therapeutic physical exercise in the combined treatment of eye burns in children]. *Oftalmol Zh*. 1984;(3):189.

1627. Oleson C V, Burns AS, Ditunno JF, Geisler FH, Coleman WP. Prognostic value of pinprick preservation in motor complete, sensory incomplete spinal cord injury. *Arch Phys Med Rehabil*. 2005;86(5):988-992. doi:10.1016/j.apmr.2004.09.031

1628. Olson K, Avdagovska M, Bistritz L, Kovacs Burns K, Cui Y, Gramlich L. Use of Nutrition and Physical Activity Modules to Promote Continuing Professional Development: A Pilot Test. *J Nurses Prof Dev*. 2017;33(6):E1-E6. doi:10.1097/NND.0000000000000389

1629. Omar MTA, Ibrahim ZM, Salama AB. Patterns and predictors of hand functional recovery following pediatric burn injuries: Prospective cohort study. *BURNS*. 2022;48(8):1863-1873. doi:10.1016/j.burns.2021.11.021

1630. Omar MTA, Hegazy FA, Mokashi SP. Influences of purposeful activity versus rote exercise on improving pain and hand function in pediatric burn. *Burns*. 2012;38(2):261-268. doi:10.1016/j.burns.2011.08.004

1631. O’Neil A, Hines D, Wirdzek E, Thornburg C, Murray D, Porter J. Early Mobilization, Early Ambulation, and Burn Therapy in the Acute Hospital Setting. *Phys Med Rehabil Clin N Am*. 2023;34(4):733-754. doi:10.1016/j.pmr.2023.06.029

1632. Öner M, Kalanlar B, Demir S, Özyurt N, Erul A, Şenel E. Challenges, expectations, and cultural care experiences of nurses regarding migrant children receiving burn treatment and their caregivers: A qualitative study. *Burns*. 2023;49(7):1706-1713. doi:10.1016/j.burns.2023.02.004

1633. Ono I, Gunji H, Zhang JZ, Maruyama K, Kaneko F. A study of cytokines in burn blister fluid related to wound healing. *Burns*. 1995;21(5):352-355. doi:10.1016/0305-4179(95)00005-4

1634. O’Riordan DL, Glanz K, Gies P, Elliott T. A pilot study of the validity of self-reported ultraviolet radiation exposure and sun protection practices among lifeguards, parents and children. *Photochem Photobiol*. 2008;84(3):774-778. doi:10.1111/j.1751-1097.2007.00262.x

1635. Osborne T, Edgar D, Gittings P, et al. A prospective pilot study of the energy balance profiles in acute non-severe burn patients. *BURNS*. 2022;48(1):184-190. doi:10.1016/j.burns.2021.03.002

1636. Osborne T, Wall B, Edgar DW, Fairchild T, Wood F. Current understanding of the chronic stress response to burn injury from human studies. *Burn trauma*. 2023;11:tkad007. doi:10.1093/burnst/tkad007

1637. O’Shea SD, Taylor NF, Paratz JD. Progressive resistance exercise improves muscle strength and may improve elements of performance of daily activities for people with COPD: a systematic review. *Chest*. 2009;136(5):1269-1283. doi:10.1378/chest.09-0029

1638. Osimitz TG, Droege W, Hendriks G, Blais MS. Evaluation of potential toxicity of smoke from controlled burns of furnished rooms - effect of flame retardancy. *J Toxicol Environ Heal A-CURRENT ISSUES*. 2022;85(19):783-797. doi:10.1080/15287394.2022.2087812

1639. Osváth P, Szucs M, Börzsei D, et al. Andrological Aspects of Exercise: Moderate Swimming Protects against Isoproterenol Induced Testis and Semen Abnormalities in Rats. *ANTIOXIDANTS*. 2022;11(3). doi:10.3390/antiox11030436

1640. Otaghvar HA. A systematic review of the exercise effects on burn wound healing. 2024;(October 2023):1-11. doi:10.1111/iwj.14482

1641. Othman EM, Toson RA. Response of bone mineral density and balance performance in post-burn patients with selected Qigong training: A single-blind randomized controlled trial. *Burns*. 2024;50(2):495-506. doi:10.1016/j.burns.2023.03.001

1642. O’Toole G, Rayatt S. Frostbite at the gym: a case report of an ice pack burn. *Br J Sports Med*. 1999;33(4):278-279. doi:10.1136/bjsm.33.4.278

1643. O’Toole JK, Hepps J, Starmer AJ, et al. I-PASS Mentored Implementation Handoff Curriculum: Frontline Provider Training Materials. *MedEdPORTAL J Teach Learn Resour*. 2020;16:10912. doi:10.15766/mep_2374-8265.10912

1644. Oudit GY, Butany J, Williams WG, Siu SC, Clarke JTR, Iwanochko RM. Left ventricular aneurysm in a patient with mucopolysaccharidosis type VI (Maroteaux-Lamy syndrome): clinical and pathological correlation. *Cardiovasc Pathol Off J Soc Cardiovasc Pathol*. 2007;16(4):237-240. doi:10.1016/j.carpath.2006.11.009

1645. Owen AM, Hampshire A, Grahn JA, et al. Putting brain training to the test. *Nature*. 2010;465(7299):775-778. doi:10.1038/nature09042

1646. Oyama IA, Aaronoff MC, Burlingame JM. Obstetric anal sphincter injury repair workshop for residents. *Hawaii Med J*. 2009;68(6):133-135.

1647. Oyo-Ita OE, Oyo-Ita IO. PAH depositional history and sources in recent sediment core from Ukwa Ibom Lake, S. E. Nigeria. *Environ Geochem Health*. 2013;35(2):185-199. doi:10.1007/s10653-012-9475-x

1648. Özkal Ö. Letter to the Editor Concerning the Article: “Treadmill Versus Overground Gait Training in Patients With Lower Limb Burn Injury: A Comparative Study” Is There just a Title Similarity? *J Burn care Res Off Publ Am Burn Assoc*. 2023;44(5):1264. doi:10.1093/jbcr/irad094

1649. Özkal Ö, Kısmet K, Konan A, Hayran M, Topuz S. Treadmill versus overground gait training in patients with lower limb burn injury: a matched control study. *Burns*. 2022;48(1):51‐58. doi:10.1016/j.burns.2021.04.019

1650. Özkal Ö, Seyyah M, Topuz S, Konan A. Lower limb functional status and its determinants in moderate/major burns 3-6 months following injury: A two-center observational study. *Burns*. 2021;47(3):676-683. doi:10.1016/j.burns.2020.07.025

1651. Özkal Ö, Seyyah M, Topuz S, Konan A. Lower limb functional status and its determinants in moderate/major burns 3–6 months following injury: A two-center observational study. *Burns*. 2021;47(3):676-683. doi:10.1016/j.burns.2020.07.025

1652. Özkal Ö, Topuz S, Karahan S, Erdem MM, Konan A, Yastı AÇ. Clinical predictors of pulmonary functions, respiratory/peripheral muscle strength and exercise capacity at discharge in adults with burn injury. *Disabil Rehabil*. 2021;43(20):2875-2881. doi:10.1080/09638288.2020.1720320

1653. Ozkal O, Yurdalan SU, Seyyah M, Acar HA. The effect of burn severity on functional capacity in patients with burn injury. *J Back Musculoskelet Rehabil*. 2019;32(2):215-221. doi:10.3233/BMR-171106

1654. Özlü Ö, Başaran A. Elderly burns: Clinical frailty scale and functional ambulation classification in predicting prognosis. *Ulus travma ve acil cerrahi Derg = Turkish J trauma Emerg Surg TJTES*. 2022;28(6):812-817. doi:10.14744/tjtes.2022.49400

1655. Pacheco M, Agner JL, Myers TK, et al. Health outcomes and healthcare utilization of Native Hawaiians and other Pacific Islanders living with HIV in Hawai’i: A mixed-methods study. *Ethn Health*. 2022;27(8):1841-1858. doi:10.1080/13557858.2021.1990219

1656. Packard CJ, Cavanagh J, McLean JS, et al. Interaction of personality traits with social deprivation in determining mental wellbeing and health behaviours. *J Public Health (Oxf)*. 2012;34(4):615-624. doi:10.1093/pubmed/fds030

1657. Pagliuca LM, Costa EM, Costa NM, Sousa KM. [Development of technologies for the prevention and treatment of domestic emergencies in the blind]. *Rev Bras Enferm*. 1996;49(1):83-104. doi:10.1590/s0034-71671996000100010

1658. Palackic A, Abazie S, Parry I, et al. Comparison of Six-Minute Walk Test and Modified Bruce Treadmill Test in Paediatric Patients With Severe Burns: a Cross-Over Study. *J Rehabil Med*. 2022;54:jrm00305. doi:10.2340/jrm.v54.1064

1659. Palackic A, Rego A, Parry I, et al. Effects of Aerobic Exercise in the Intensive Care Unit on Patient-Reported Physical Function and Mental Health Outcomes in Severely Burned Children—A Multicenter Prospective Randomized Trial. *J Pers Med*. 2023;13(3). doi:10.3390/jpm13030455

1660. Palackic A, Suman OE, Porter C, Murton AJ, Crandall CG, Rivas E. Rehabilitative Exercise Training for Burn Injury. *Sports Med*. 2021;51(12):2469-2482. doi:10.1007/s40279-021-01528-4

1661. Palejwala Z, Wallman K, Ward MK, et al. Effects of a hot ambient operating theatre on manual dexterity, psychological and physiological parameters in staff during a simulated burn surgery. *PLoS One*. 2019;14(10). doi:10.1371/journal.pone.0222923

1662. Palejwala Z, Wallman KE, Landers GJ, Anbalagan P, Wood FM, Maloney SK. Living in Western Australia induces some physiological adaptations of seasonal acclimatisation in the surgical burns team. *Temp (Austin, Tex)*. 2024;11(2):110-122. doi:10.1080/23328940.2023.2281210

1663. Palmer JA, Kaufman CS, Vidoni ED, Honea RA, Burns JM, Billinger SA. Sex Differences in Resilience and Resistance to Brain Pathology and Dysfunction Moderated by Cerebrovascular Response to Exercise and Genetic Risk for Alzheimer’s Disease. *J ALZHEIMERS Dis*. 2022;90(2):535-542. doi:10.3233/JAD-220359

1664. Palmer JA, Kaufman CS, Vidoni ED, Honea RA, Burns JM, Billinger SA. Cerebrovascular response to exercise interacts with individual genotype and amyloid-beta deposition to influence response inhibition with aging. *Neurobiol Aging*. 2022;114:15-26. doi:10.1016/j.neurobiolaging.2022.02.014

1665. Palmieri TL. What’s new in critical care of the burn-injured patient? *Clin Plast Surg*. 2009;36(4):607-615. doi:10.1016/j.cps.2009.05.012

1666. Palmieri T, Committee IPG. ISBI Practice Guidelines for Burn Care, Part 2. *Burn*. 2018;44(7):1617-1706. doi:10.1016/j.burns.2018.09.012

1667. Paluska SA. An overview of hip injuries in running. *Sports Med*. 2005;35(11):991-1014. doi:10.2165/00007256-200535110-00005

1668. Pandolf KB, Gange RW, Latzka WA, Blank IH, Kraning KK 2nd, Gonzalez RR. Human thermoregulatory responses during heat exposure after artificially induced sunburn. *Am J Physiol*. 1992;262(4 Pt 2):R610-6. doi:10.1152/ajpregu.1992.262.4.R610

1669. Pandolf KB, Gange RW, Latzka WA, Blank IH, Young AJ, Sawka MN. Human thermoregulatory responses during cold water immersion after artificially induced sunburn. *Am J Physiol*. 1992;262(4 Pt 2):R617-23. doi:10.1152/ajpregu.1992.262.4.R617

1670. Pantalos GM, Chaing BY, Bishop DN, et al. Development of smaller artificial ventricles and valves made by vacuum forming. *Int J Artif Organs*. 1988;11(5):373-380.

1671. Paraskevopoulou D, Liakakou E, Gerasopoulos E, Mihalopoulos N. Sources of atmospheric aerosol from long-term measurements (5 years) of chemical composition in Athens, Greece. *Sci Total Environ*. 2015;527-528:165-178. doi:10.1016/j.scitotenv.2015.04.022

1672. Paratz JD, Stockton K, Plaza A, Muller M, Boots RJ. Intensive exercise after thermal injury improves physical, functional, and psychological outcomes. *J Trauma Acute Care Surg*. 2012;73(1):186-194. doi:10.1097/TA.0b013e31824baa52

1673. Parikh JR, Van Moore A, Mead L, Bassett R, Rubin E. Prevalence of Burnout of Radiologists in Private Practice. *J Am Coll Radiol*. 2023;20(7):712-718. doi:10.1016/j.jacr.2023.01.007

1674. Paris PM, Stewart RD, Pelton GH, Porter G, Sanzo A. Triage success in disasters: dynamic victim-tracking cards. *Am J Emerg Med*. 1985;3(4):323-326. doi:10.1016/0735-6757(85)90057-9

1675. Parisi A V, Kimlin MG, Mulheran L, Meldrum LR, Randall C. Field-based measurements of personal erythemal ultraviolet exposure through a common summer garment. *Photodermatol Photoimmunol Photomed*. 2000;16(3):134-138. doi:10.1034/j.1600-0781.2000.d01-20.x

1676. Park E, Oh H, Kim T. The effects of relaxation breathing on procedural pain and anxiety during burn care. *Burn*. 2013;39(6):1101-1106. doi:10.1016/j.burns.2013.01.006

1677. Parker LK, Ponte C, Howell KJ, Ong VH, Denton CP, Schreiber BE. Clinical features and management of erythromelalgia: long term follow-up of 46 cases. *Clin Exp Rheumatol*. 2017;35(1):80-84.

1678. Parker M, Delahunty B, Heberlein N, et al. Interactive gaming consoles reduced pain during acute minor burn rehabilitation: a randomized, pilot trial. *Burns*. 2016;42(1):91‐96. doi:10.1016/j.burns.2015.06.022

1679. Parr TJ, Burns TC. Overuse injuries of the olecranon in adolescents. *Orthopedics*. 2003;26(11):1143-1146. doi:10.3928/0147-7447-20031101-16

1680. Parra R, Saud C, Espinoza C. Simulating PM_2.5_ Concentrations during New Year in Cuenca, Ecuador: Effects of Advancing the Time of Burning Activities. *TOXICS*. 2022;10(5). doi:10.3390/toxics10050264

1681. Parrott M, Ryan R, Parks DH, Wainwright DJ. Structured exercise circuit program for burn patients. *J Burn Care Rehabil*. 1988;9(6):666-668. doi:10.1097/00004630-198811000-00023

1682. Parry CB. Problems in rehabilitation of the burnt hand. *Hand*. 1970;2(2):140-144. doi:10.1016/0072-968x(70)90012-4

1683. Parry E, Ogollah R, Peat G. “Acute flare-ups” in patients with, or at high risk of, knee osteoarthritis: a daily diary study with case-crossover analysis. *Osteoarthr Cartil*. 2019;27(8):1124-1128. doi:10.1016/j.joca.2019.04.003

1684. Parry IS, Bagley A, Kawada J, Sen S, Greenhalgh DG, Palmieri TL. Commercially available interactive video games in burn rehabilitation: therapeutic potential. *Burns*. 2012;38(4):493-500. doi:10.1016/j.burns.2012.02.010

1685. Parry IS, Schneider JC, Yelvington M, et al. Systematic Review and Expert Consensus on the Use of Orthoses (Splints and Casts) with Adults and Children after Burn Injury to Determine Practice Guidelines. *J Burn care Res Off Publ Am Burn Assoc*. 2020;41(3):503-534. doi:10.1093/jbcr/irz150

1686. Parry I, Carbullido C, Kawada J, et al. Keeping up with video game technology: objective analysis of Xbox Kinect^TM^ and PlayStation 3 Move^TM^ for use in burn rehabilitation. *Burns*. 2014;40(5):852-859. doi:10.1016/j.burns.2013.11.005

1687. Parry I, Painting L, Bagley A, et al. A Pilot Prospective Randomized Control Trial Comparing Exercises Using Videogame Therapy to Standard Physical Therapy: 6 Months Follow-Up. *J Burn Care Res*. 2015;36(5):534-544. doi:10.1097/BCR.0000000000000165

1688. Parry I, Sen S, Palmieri T, Greenhalgh D. Nonsurgical scar management of the face: does early versus late intervention affect outcome? *J Burn Care Res*. 2013;34(5):569-575. doi:10.1097/BCR.0b013e318278906d

1689. Parry I, Sen S, Sattler-Petrocchi K, Greenhalgh D, Palmieri T. Cutaneous Functional Units Predict Shoulder Range of Motion Recovery in Children Receiving Rehabilitation. *J Burn Care Res*. 2017;38(2):106-111. doi:10.1097/BCR.0000000000000429

1690. Parry SW. Reconstruction of the burned hand. *Clin Plast Surg*. 1989;16(3):577-586.

1691. Parsons ECM, Dolman SJ, Wright AJ, Rose NA, Burns WCG. Navy sonar and cetaceans: just how much does the gun need to smoke before we act? *Mar Pollut Bull*. 2008;56(7):1248-1257. doi:10.1016/j.marpolbul.2008.04.025

1692. Patil PG, Hazarey V, Chaudhari R, Nimbalkar-Patil S. A randomized control trial measuring the effectiveness of a mouth-exercising device for mucosal burning in oral submucous fibrosis. *Oral Surg Oral Med Oral Pathol Oral Radiol*. 2016;122(6 CC-Oral Health):713‐718. doi:10.1016/j.oooo.2016.07.026

1693. Patil PG, Hazarey V, Chaudhari R, Nimbalkar-Patil S. Reply to: Details of the participants and medical intervention used in the study evaluating the effect of a mouth exercising device on burning sensation in oral submucous fibrosis. *Oral Surg Oral Med Oral Pathol Oral Radiol*. 2017;123(6):735-737. doi:10.1016/j.oooo.2017.01.017

1694. Patterson R, Wang L, McVeigh G, Burns R, Cohn J. Impedance cardiography: the failure of sternal electrodes to predict changes in stroke volume. *Biol Psychol*. 1993;36(1-2):33-41. doi:10.1016/0301-0511(93)90078-m

1695. Paul-Labrador M, Vongvanich P, Merz CN. Risk stratification for exercise training in cardiac patients: do the proposed guidelines work? *J Cardiopulm Rehabil*. 1999;19(2):118-125. doi:10.1097/00008483-199903000-00006

1696. Pearson FG, Brito-Filomeno L, Cooper JD. Experience with partial cricoid resection and thyrotracheal anastomosis. *Ann Otol Rhinol Laryngol*. 1986;95(6 Pt 1):582-585. doi:10.1177/000348948609500608

1697. Pearson J, Ganio MS, Schlader ZJ, et al. Post Junctional Sudomotor and Cutaneous Vascular Responses in Noninjured Skin Following Heat Acclimation in Burn Survivors. *J Burn Care Res*. 2017;38(1):e284-e292. doi:10.1097/BCR.0000000000000372

1698. Pegg SP, Cavaye D, Fowler D, Jones M. Results of early excision and grafting in hand burns. *Burns Incl Therm Inj*. 1984;11(2):99-103. doi:10.1016/0305-4179(84)90131-1

1699. Peña R, Suman OE, Rosenberg M, Andersen CR, Herndon DN, Meyer WJ. One-Year Comparison of a Community-Based Exercise Program Versus a Day Hospital-Based Exercise Program on Quality of Life and Mental Health in Severely Burned Children. *Arch Phys Med Rehabil*. 2020;101(1):S26-S35. doi:10.1016/j.apmr.2017.10.023

1700. Peña R, Ramirez LL, Crandall CG. Effects of Community-Based Exercise in Children with Severe Burns: A Randomized Trial. *Burns*. 2016;42(1):41-47. doi:10.1016/j.burns.2015.07.007.Effects

1701. Peng H, Liang PF, Wang A, Yue LQ. Influences of different rehabilitative methods on function of hands and psychological anxiety of patients with deeply burned hands retaining denatured dermis and grafting large autologous skin. *Zhonghua shao shang za zhi [Chinese J Burn*. 2017;33(5):272‐276. doi:10.3760/cma.j.issn.1009-2587.2017.05.004

1702. Penttilä S, Vihola A, Palmio J, Udd B. ANO5 Muscle Disease. In: Adam MP, Feldman J, Mirzaa GM, Pagon RA, Wallace SE, Amemiya A, eds. ; 1993.

1703. Perales J, Burns J, Vidoni ED, Hinton L, Burns J, Vidoni ED. Cardiovascular health and cognitive function among Mexican older adults: cross-sectional results from the WHO Study on Global Ageing and Adult Health. *Int Psychogeriatrics*. 2018;30(12):1827-1836. doi:10.1017/S1041610218000297

1704. Perdomo SJ, Ward J, Liu Y, et al. Cardiovascular disease risk is associated with middle cerebral artery blood flow velocity in older adults. *Cardiopulm Phys Ther J*. 2020;31(2):38-46. doi:10.1097/cpt.0000000000000110

1705. Perea R, Vidoni E, Morris J, et al. Cardiorespiratory fitness and white matter integrity in Alzheimer’s disease. *Brain Imaging Behav*. 2016;10(3):660-668. doi:10.1007/s11682-015-9431-3

1706. Pereira CE, Rover CA, Whiteley MS. Endovenous Thermal Ablation of Prominent Central Forehead Veins (Supratrochlear Veins). *DERMATOLOGIC Surg*. 2021;47(3):E97-E100. doi:10.1097/DSS.0000000000002778

1707. Pereira CT, Herndon DN. The pharmacologic modulation of the hypermetabolic response to burns. *Adv Surg*. 2005;39:245-261. doi:10.1016/j.yasu.2005.05.005

1708. Pereira CT, Murphy KD, Herndon DN. Altering metabolism. *J Burn Care Rehabil*. 2005;26(3):194-199.

1709. Pereira C, Murphy K, Herndon D. Outcome measures in burn care. Is mortality dead? *Burns*. 2004;30(8):761-771. doi:10.1016/j.burns.2004.05.012

1710. Pereira C, Murphy K, Jeschke M, Herndon DN. Post burn muscle wasting and the effects of treatments. *Int J Biochem Cell Biol*. 2005;37(10):1948-1961. doi:10.1016/j.biocel.2005.05.009

1711. Pereira JL, Garrido M, Gómez-Cía T, et al. [Enteral nutrition in burn patients]. *Nutr Hosp*. 1992;7(5):340-345.

1712. Perez M, Donaldson M, Jain N, Robinson JK. Sun Protection Behaviors in Head Start and Other Early Childhood Education Programs in Illinois. *JAMA dermatology*. 2018;154(3):336-340. doi:10.1001/jamadermatol.2017.5257

1713. Pérez-García A, Salom M, Villaverde-Doménech ME, Baixauli F, Simón-Sanz E. Free microvascular rotationplasty with nerve repair for rhabdomyosarcoma in a 18-month-old patient. *Microsurgery*. 2017;37(4):344-347. doi:10.1002/micr.30147

1714. Perri G, Mathers JC, Martin-Ruiz C, et al. The association between selenium status and global and attention-specific cognition in very old adults in the Newcastle 85+ Study: cross-sectional and longitudinal analyses. *Am J Clin Nutr*. 2024;120(5):1019-1028. doi:10.1016/j.ajcnut.2024.09.004

1715. Perry AW, Goodwin CW, Finkelstein JL, Madden MR, Krizek TJ. Use of vascular clips to approximate skin grafts. *J Burn Care Rehabil*. 1988;9(5):490-491. doi:10.1097/00004630-198809000-00010

1716. Peters J, Halloran K, Focht M, Huang K, Kersh M, Rice I. Cardiorespiratory Responses to an Acute Bout of High Intensity Interval Training and Moderate Intensity Continuous Training on a Recumbent Handcycle in People With Spinal Cord Injury: A Within-Subject Design. *Top Spinal Cord Inj Rehabil*. 2023;29(4):16-26. doi:10.46292/sci23-00026

1717. Petibois C, Cazorla G, Poortmans JR, Déléris G. Biochemical aspects of overtraining in endurance sports : the metabolism alteration process syndrome. *Sports Med*. 2003;33(2):83-94. doi:10.2165/00007256-200333020-00001

1718. Petinaux B, Valenta AL, Deatley C, Conlon KM, Ott JD, Jeng JC. District of Columbia Emergency Healthcare Coalition Burn Mass Casualty Plan: Development to Exercise Date. *J Burn Care Res*. 2017;38(1):e299-e305. doi:10.1097/BCR.0000000000000375

1719. Petkar K. Opposite-Running Double Abdominal Flap on Single Pivot Point (Udaipur Flap) for Circumferential Coverage of Wrist and Hand. *Ann Plast Surg*. 2020;85(5):495-501. doi:10.1097/SAP.0000000000002458

1720. Petrin Z, Wowkanech C, Sinha AN, Gupta S, Patel MK. Female Runner With Painful Left Thigh Swelling: A Case of May-Thurner Syndrome. *PM R*. 2018;10(2):227-229. doi:10.1016/j.pmrj.2017.06.016

1721. Petroff PA, Hander EW, Mason ADJ. Ventilatory patterns following burn injury and effect of sulfamylon. *J Trauma*. 1975;15(8):650-656. doi:10.1097/00005373-197508000-00005

1722. Petrofsky JS, McLellan K, Prowse M, et al. The effect of body fat, aging, and diabetes on vertical and shear pressure in and under a waist belt and its effect on skin blood flow. *Diabetes Technol Ther*. 2010;12(2):153-160. doi:10.1089/dia.2009.0123

1723. Pettersson LME, Danielsen N, Dahlin LB. Altered behavioural responses and functional recovery in rats following sciatic nerve compression and early vs late decompression. *J Plast Surg Hand Surg*. 2016;50(6):321-330. doi:10.3109/2000656X.2016.1168742

1724. Pettijohn KJ, Asdigian NL, Aalborg J, et al. Vacations to waterside locations result in nevus development in Colorado children. *Cancer Epidemiol biomarkers Prev a Publ Am Assoc Cancer Res cosponsored by Am Soc Prev Oncol*. 2009;18(2):454-463. doi:10.1158/1055-9965.EPI-08-0634

1725. Pfledderer CD, Bai Y, Brusseau TA, Burns RD, Jensen JLK. Changes in college students’ health behaviors and substance use after a brief wellness intervention during COVID-19. *Prev Med REPORTS*. 2022;26. doi:10.1016/j.pmedr.2022.101743

1726. Pfledderer CD, Burns RD, Byun W, Carson RL, Welk GJ, Brusseau TA. Parent Preferences for Physical Activity in Before and After School Programs in Rural and Suburban Communities: A Discrete Choice Experiment. *J Phys Act Health*. 2021;18(12):1479-1489. doi:10.1123/jpah.2021-0220

1727. Pfledderer CD, Kwon S, Strehli I, Byun W, Burns RD. The Effects of Playground Interventions on Accelerometer-Assessed Physical Activity in Pediatric Populations: A Meta-Analysis. *Int J Environ Res Public Health*. 2022;19(6). doi:10.3390/ijerph19063445

1728. Pfledderer CD, Burns RD, Brusseau TA. School environment, physical activity, and sleep as predictors of suicidal ideation in adolescents: Evidence from a national survey. *J Adolesc*. 2019;74:83-90. doi:10.1016/j.adolescence.2019.05.008

1729. Pfledderer CD, Burns RD, Byun W, Carson RL, Welk GJ, Brusseau TA. School-based physical activity interventions in rural and urban/suburban communities: A systematic review and meta-analysis. *Obes Rev an Off J Int Assoc Study Obes*. 2021;22(9):e13265. doi:10.1111/obr.13265

1730. Pfledderer CD, Burns RD, Byun W, Carson RL, Welk GJ, Brusseau TA. Parent and Child Perceptions of Barriers to Active School Commuting. *J Sch Health*. 2021;91(12):1014-1023. doi:10.1111/josh.13090

1731. Pham TN, Wong JN, Terken T, Gibran NS, Carrougher GJ, Bunnell A. Feasibility of a Kinect®-based rehabilitation strategy after burn injury. *Burns*. 2018;44(8):2080‐2086. doi:10.1016/j.burns.2018.08.032

1732. Pham TN, Wong JN, Terken T, Gibran NS, Carrougher GJ, Bunnell A. Feasibility of a Kinect(®)-based rehabilitation strategy after burn injury. *Burns*. 2018;44(8):2080-2086. doi:10.1016/j.burns.2018.08.032

1733. Phelan I, Furness PJ, Dunn HD, et al. Immersive virtual reality in children with upper limb injuries: Findings from a feasibility study. *J Pediatr Rehabil Med*. 2021;14(3):401-414. doi:10.3233/PRM-190635

1734. Phillips AC, Burns VE, Carroll D, Ring C, Drayson M. The association between life events, social support, and antibody status following thymus-dependent and thymus-independent vaccinations in healthy young adults. *Brain Behav Immun*. 2005;19(4):325-333. doi:10.1016/j.bbi.2004.10.004

1735. Phillips AC, Burns VE, Lord JM. Stress and exercise: Getting the balance right for aging immunity. *Exerc Sport Sci Rev*. 2007;35(1):35-39. doi:10.1097/jes.0b013e31802d7008

1736. Phillips AC, Carroll D, Burns VE, Drayson M. Neuroticism, cortisol reactivity, and antibody response to vaccination. *Psychophysiology*. 2005;42(2):232-238. doi:10.1111/j.1469-8986.2005.00281.x

1737. Phillips AC, Carroll D, Burns VE, Drayson M. Cardiovascular activity and the antibody response to vaccination. *J Psychosom Res*. 2009;67(1):37-43. doi:10.1016/j.jpsychores.2008.12.002

1738. Phillips AC, Carroll D, Burns VE, Ring C, Macleod J, Drayson M. Bereavement and marriage are associated with antibody response to influenza vaccination in the elderly. *Brain Behav Immun*. 2006;20(3):279-289. doi:10.1016/j.bbi.2005.08.003

1739. Phillips DS, Hannon JC, Gregory BB, Burns RD. Effect of Vigorous Physical Activity on Executive Control in Middle-School Students. *Int J Environ Res Public Health*. 2019;16(20). doi:10.3390/ijerph16203949

1740. Phillips L, Cohen J, Burns E, Abrams J, Renninger S. Self-management of chronic illness: the role of “habit” versus reflective factors in exercise and medication adherence. *J Behav Med*. 2016;39(6):1076-1091. doi:10.1007/s10865-016-9732-z

1741. Phillips SM, Glover EI, Rennie MJ. Alterations of protein turnover underlying disuse atrophy in human skeletal muscle. *J Appl Physiol*. 2009;107(3):645-654. doi:10.1152/japplphysiol.00452.2009

1742. Phillips SM, Tipton KD, Aarsland A, Wolf SE, Wolfe RR. Mixed muscle protein synthesis and breakdown after resistance exercise in humans. *Am J Physiol*. 1997;273(1 Pt 1):E99-107. doi:10.1152/ajpendo.1997.273.1.E99

1743. Phillips SM, Tipton KD, Ferrando AA, Wolfe RR. Resistance training reduces the acute exercise-induced increase in muscle protein turnover. *Am J Physiol*. 1999;276(1):E118-24. doi:10.1152/ajpendo.1999.276.1.E118

1744. Phoon Nguyen AH, Balasubramaniam R, Bellan V, Newport RN, Stanton TR. The effect of multisensory illusions on pain and perceived burning sensations in patients with Burning Mouth Syndrome: A proof-of-concept study. *J oral Pathol Med Off Publ Int Assoc Oral Pathol Am Acad Oral Pathol*. 2020;49(6):505-513. doi:10.1111/jop.13065

1745. Pieptu D, Luchian S, Copăceanu M. [The treatment of superficial burns of the hands under controlled microclimate conditions]. *Rev Med Chir Soc Med Nat Iasi*. 1997;101(1-2):160-163.

1746. Pierce B, Bowden B, McCullagh M, et al. A Summer Health Program for African-American High School Students in Baltimore, Maryland: Community Partnership for Integrative Health. *Explore (NY)*. 2017;13(3):186-197. doi:10.1016/j.explore.2017.02.002

1747. Piña IL, Kokkinos P, Kao A, et al. Baseline differences in the HF-ACTION trial by sex. *Am Heart J*. 2009;158(4 Suppl):S16-23. doi:10.1016/j.ahj.2009.07.012

1748. Platkovskaia RI. [Tubal breathing as an effective method during exercise therapy in burns]. *Vopr Kurortol Fizioter Lech Fiz Kult*. 1972;37(1):71-75.

1749. Plaza A, Adsett J, Byrnes A, McRae P. Physical Activity Levels in Hospitalized Adults With Burn Injuries. *J Burn CARE Res*. 2022;43(5):1048-1054. doi:10.1093/jbcr/irab233

1750. Plaza A, Paratz J, Cottrell M. A six-week physical therapy exercise program delivered via home-based telerehabilitation is comparable to in-person programs for patients with burn injuries: A randomized, controlled, non-inferiority clinical pilot trial. *Burn*. 2023;49(1):55-67. doi:10.1016/j.burns.2022.08.014

1751. Podlog L, Burns R, Dimmock JA, Jackson B, Hall MS, Fritz JM. Does motivation mediate the relationship between competence perceptions and patient outcomes among individuals with chronic low back pain? A multiple mediation analysis. *Disabil Rehabil*. 2021;43(7):953-959. doi:10.1080/09638288.2019.1643421

1752. Pollard CA, Burns DS, Ho B, Johnsto AM. Meningoencephalitis in a Royal Marine after skinning reindeer in Norway. *J R ARMY Med CORPS*. 2018;164(2):117-119. doi:10.1136/jramc-2017-000848

1753. Polychronopoulou E, Herndon DN, Porter C. The Long-Term Impact of Severe Burn Trauma on Musculoskeletal Health. *J Burn Care Res*. 2018;39(6):869-880. doi:10.1093/jbcr/iry035

1754. Pombo A, Cordovil R, Rodrigues LP, et al. Effect of Motor Competence and Health-Related Fitness in the Prevention of Metabolic Syndrome Risk Factors. *Res Q Exerc Sport*. 2024;95(1):110-117. doi:10.1080/02701367.2022.2158998

1755. Pontell ME, Sparber LS, Chamberlain RS. Corrective and Reconstructive Surgery in Patients With Postburn Heterotopic Ossification and Bony Ankylosis: An Evidence-Based Approach. *J Burn Care Res*. 2015;36(1):57-69. doi:10.1097/BCR.0000000000000116

1756. Porro LJ, Al-Mousawi AM, Williams F, Herndon DN, Mlcak RP, Suman OE. Effects of propranolol and exercise training in children with severe burns. *J Pediatr*. 2013;162(4):799‐803.e1. doi:10.1016/j.jpeds.2012.09.015

1757. Porro LJ, Herndon DN, Rodriguez NA, et al. Five-year outcomes after oxandrolone administration in severely burned children: a randomized clinical trial of safety and efficacy. *J Am Coll Surg*. 2012;214(4 CC-Injuries):489‐502; discussion 502‐4. doi:10.1016/j.jamcollsurg.2011.12.038

1758. Porro L, Rivero HG, Gonzalez D, Tan A, Herndon DN, Suman OE. Prediction of maximal aerobic capacity in severely burned children. *Burn*. 2011;37(4):682-686. doi:10.1016/j.burns.2010.12.021

1759. Porter C, Hardee JP, Herndon DN, Suman OE. The role of exercise in the rehabilitation of patients with severe burns. *Exerc Sport Sci Rev*. 2015;43(1):34-40. doi:10.1249/JES.0000000000000029

1760. Porter C, Hurren NM, Herndon DN, Børsheim E. Whole body and skeletal muscle protein turnover in recovery from burns. *Int J Burns Trauma*. 2013;3(1):9-17.

1761. Potts C, Lindstroem F, Bond R, et al. A Multilingual Digital Mental Health and Well-Being Chatbot (ChatPal): Pre-Post Multicenter Intervention Study. *J Med INTERNET Res*. 2023;25. doi:10.2196/43051

1762. Powell K, Burns MC, Prasad V. Relugolix: Five Reasons Why the US Food and Drug Administration Should Have Exercised Restraint. *Eur Urol*. 2023;83(2):101-102. doi:10.1016/j.eururo.2022.08.029

1763. Powers MB, Medina JL, Burns S, et al. Exercise Augmentation of Exposure Therapy for PTSD: Rationale and Pilot Efficacy Data. *Cogn Behav Ther*. 2015;44(4):314-327. doi:10.1080/16506073.2015.1012740

1764. Pramanik T, Regmi P, Shrestha P. Detection of individuals prone to develop hypertension in their future life. *Nepal Med Coll J*. 2008;10(1):35-37.

1765. Prasanna S, Barua S, Siller AF, Johnson JJ, Sabharwal A, DeSalvo DJ. Hypoglycemia risk with physical activity in type 1 diabetes: a data-driven approach. *Front Digit Heal*. 2023;5. doi:10.3389/fdgth.2023.1142021

1766. Prasetyono T, Caroline I. The role of two-sided splinting for recalcitrant paediatric post-burn hand flexion contracture: a case report. *Ann R Coll Surg Engl*. 2017;99(6):e185-e187. doi:10.1308/rcsann.2017.0103

1767. PRATT DR. INJURIES OF THE UPPER EXTREMITY. THE ROLE OF THE RECONSTRUCTIVE SURGEON. *Calif Med*. 1964;101(3):174-179.

1768. Prelack K, Yu YM, Dylewski M, Lydon M, Keaney TJ, Sheridan RL. Measures of Total Energy Expenditure and Its Components Using the Doubly Labeled Water Method in Rehabilitating Burn Children. *JPEN J Parenter Enteral Nutr*. 2017;41(3):470-480. doi:10.1177/0148607115597665

1769. Pretty IA, Webb DA, Sweet D. Dental participants in mass disasters--a retrospective study with future implications. *J Forensic Sci*. 2002;47(1):117-120.

1770. Pretz R, Brown C, Hughes WB, Altschuler EL. Maximizing functional mobility in an electrical burn patient using a patellar tendon bearing orthosis. *J Rehabil Med*. 2016;48(7):636-638. doi:10.2340/16501977-2114

1771. Prevedouros K, Jones KC, Sweetman AJ. Modelling the atmospheric fate and seasonality of polycyclic aromatic hydrocarbons in the UK. *Chemosphere*. 2004;56(3):195-208. doi:10.1016/j.chemosphere.2004.02.032

1772. Prewitt JS, Freistroffer D V, Schreer JF, Hammill MO, Burns JM. Postnatal development of muscle biochemistry in nursing harbor seal (Phoca vitulina) pups: limitations to diving behavior? *J Comp Physiol B, Biochem Syst Environ Physiol*. 2010;180(5):757-766. doi:10.1007/s00360-010-0448-z

1773. Pritchard A, Burns P, Correia J, et al. ARTP statement on cardiopulmonary exercise testing 2021. *BMJ OPEN Respir Res*. 2021;8(1). doi:10.1136/bmjresp-2021-001121

1774. Prohaska T, Burholt V, Burns A, et al. Consensus statement: loneliness in older adults, the 21st century social determinant of health? *BMJ Open*. 2020;10(8). doi:10.1136/bmjopen-2019-034967

1775. PROYARD. [SEVERAL PROBLEMS POSED BY HAND BURNS]. *Scalpel (Brux)*. 1964;117:305-313.

1776. Pryor RR, Bennett BL, O’Connor FG, Young JMJ, Asplund CA. Medical Evaluation for Exposure Extremes: Heat. *Wilderness Environ Med*. 2015;26(4 Suppl):S69-75. doi:10.1016/j.wem.2015.09.009

1777. Pryor RR, Bennett BL, OʼConnor FG, Young JMJ, Asplund CA. Medical Evaluation for Exposure Extremes: Heat. *Clin J Sport Med Off J Can Acad Sport Med*. 2015;25(5):437-442. doi:10.1097/JSM.0000000000000248

1778. Przkora R, Herndon DN, Suman OE. The effects of oxandrolone and exercise on muscle mass and function in children with severe burns. *Pediatrics*. 2007;119(1 CC-Metabolic and Endocrine Disorders CC-Injuries):e109‐16. doi:10.1542/peds.2006-1548

1779. Przkora R, Herndon DN, Suma OE. The Effects of Oxandrolone and Exercise on Muscle Mass and Function in Children With Severe Burns. *Pediatrics*. 2008;119(1):1-19.

1780. Pucker AD, Kerr AM, Sanderson J, Lievens C. Digital Eye Strain: Updated Perspectives. *Clin Optom*. 2024;16:233-246. doi:10.2147/OPTO.S412382

1781. Puddicombe BE, Nardone MA. Rehabilitation of the burned hand. *Hand Clin*. 1990;6(2):281-292.

1782. Puggina A, Aleksovska K, Buck C, et al. Policy determinants of physical activity across the life course: a “DEDIPAC” umbrella systematic literature review. *Eur J Public Health*. 2018;28(1):105-118. doi:10.1093/eurpub/ckx174

1783. Purdue GF, Hunt JL. Pulmonary emboli in burned patients. *J Trauma*. 1988;28(2):218-220. doi:10.1097/00005373-198802000-00017

1784. Puri V, Shrotriya R, Bachhav M. The scourge of burn contractures: Who will bell the cat? *BURNS*. 2019;45(4):791-797. doi:10.1016/j.burns.2019.01.001

1785. Pusineri M, Monteleone S, De Bernardi E, et al. Integrating and monitoring hand burn rehabilitative treatment using Virtual Reality. *G Ital Med Lav Ergon*. 2017;39(2):113-115.

1786. Quan H, Hao W, Li L, Sun M, Zhang K. Algorithm to improve accuracy of energy expended in a room calorimeter. *Med Biol Eng Comput*. 2017;55(8):1215-1225. doi:10.1007/s11517-016-1583-9

1787. Quartetti HR. Shedding pounds for life. Burning extra calories. Exercise is essential for avoiding unwanted pounds. *Diabetes Forecast*. 2003;56(4):71-72.

1788. Quested E, Bosch JA, Burns VE, Cumming J, Ntoumanis N, Duda JL. Basic psychological need satisfaction, stress-related appraisals, and dancers’ cortisol and anxiety responses. *J Sport Exerc Psychol*. 2011;33(6):828-846. doi:10.1123/jsep.33.6.828

1789. Quinn CJ, Burns PD, Gibson NM, Bashore A, Hayward R, Hydock DS. Effects of Chronic Endurance Exercise on Doxorubicin-Induced Thymic Damage. *Integr Cancer Ther*. 2016;15(4):535-541. doi:10.1177/1534735415617014

1790. RA H, GP T, Harsha A, et al. Cardiorespiratory fitness and preserved medial temporal lobe volume in Alzheimer disease. *Alzheimer Dis Assoc Disord*. 2009;23(3):188-197. doi:10.1097/WAD.0b013e31819cb8a2

1791. Rabinovitch S, Greyson ND, Weiser W, Hoffstein V. Clinical and laboratory features of acute sulfur dioxide inhalation poisoning: two-year follow-up. *Am Rev Respir Dis*. 1989;139(2):556-558. doi:10.1164/ajrccm/139.2.556

1792. Radford JA, Burns J, Buchbinder R, Landorf KB, Cook C. Does stretching increase ankle dorsiflexion range of motion? A systematic review. *Br J Sports Med*. 2006;40(10):870-875; discussion 875. doi:10.1136/bjsm.2006.029348

1793. Radford JA, Burns J, Buchbinder R, Landorf KB, Cook C. The effect of low-Dye taping on kinematic, kinetic, and electromyographic variables: a systematic review. *J Orthop Sports Phys Ther*. 2006;36(4):232-241. doi:10.2519/jospt.2006.36.4.232

1794. Radina ME, Armer JM, Culbertson SD, Dusold JM. Post-breast cancer lymphedema: understanding women’s knowledge of their condition. *Oncol Nurs Forum*. 2004;31(1):97-104. doi:10.1188/04.ONF.97-104

1795. Radwan NL, Ibrahim MM, Mahmoud WS. Effect of Wii-habilitation on spatiotemporal parameters and upper limb function post-burn in children. *Burns*. 2021;47(4):828-837. doi:10.1016/j.burns.2020.09.010

1796. Raetz J, Wilson M, Collins K. Varicose Veins: Diagnosis and Treatment. *Am Fam Physician*. 2019;99(11):682-688.

1797. Raguso CA, Coggan AR, Gastaldelli A, Sidossis LS, Bastyr EJ 3rd, Wolfe RR. Lipid and carbohydrate metabolism in IDDM during moderate and intense exercise. *Diabetes*. 1995;44(9):1066-1074. doi:10.2337/diab.44.9.1066

1798. Raguso CA, Coggan AR, Sidossis LS, Gastaldelli A, Wolfe RR. Effect of theophylline on substrate metabolism during exercise. *Metabolism*. 1996;45(9):1153-1160. doi:10.1016/s0026-0495(96)90016-5

1799. Rahman J, Scragg R. Factors associated with self-reported sun exposure in a multi-ethnic community sample from New Zealand. *J Steroid Biochem Mol Biol*. 2022;221. doi:10.1016/j.jsbmb.2022.106131

1800. Raichurkar P, Denehy L, Solomon M, et al. Research Priorities in Prehabilitation for Patients Undergoing Cancer Surgery: An International Delphi Study. *Ann Surg Oncol*. 2023;30(12):7226-7235. doi:10.1245/s10434-023-14192-x

1801. Raine LB, Erickson KI, Grove G, et al. Cardiorespiratory fitness levels and body mass index of pre-adolescent children and older adults during the COVID-19 pandemic. *Front public Heal*. 2022;10:1052389. doi:10.3389/fpubh.2022.1052389

1802. Raipure A, Patil S, Pathan H. Effectiveness of Early Physiotherapy Rehabilitation Approach for Split Skin Grafting Post-burn in a Pediatric Patient. *Cureus*. 2023;15(8):e44083. doi:10.7759/cureus.44083

1803. Rajan JN, Ireland K, Johnson R, Stepien KM. Review of Mechanisms, Pharmacological Management, Psychosocial Implications, and Holistic Treatment of Pain in Fabry Disease. *J Clin Med*. 2021;10(18). doi:10.3390/jcm10184168

1804. Rajkumar S, Clark ML, Young BN, et al. Exposure to household air pollution from biomass-burning cookstoves and HbA1c and diabetic status among Honduran women. *Indoor Air*. 2018;28(5):768-776. doi:10.1111/ina.12484

1805. Rajkumar S, Young BN, Clark ML, et al. Household air pollution from biomass-burning cookstoves and metabolic syndrome, blood lipid concentrations, and waist circumference in Honduran women: A cross-sectional study. *Environ Res*. 2019;170:46-55. doi:10.1016/j.envres.2018.12.010

1806. Rajput AB, Burns B, Gerridzen R, van der Jagt R. Coexisting mantle cell lymphoma and prostate adenocarcinoma. *Case Rep Med*. 2014;2014:247286. doi:10.1155/2014/247286

1807. Ramelet AA. Exercise-induced vasculitis. *J Eur Acad Dermatol Venereol*. 2006;20(4):423-427. doi:10.1111/j.1468-3083.2006.01504.x

1808. Ramelet AA. Exercise-induced purpura. *Dermatology*. 2004;208(4):293-296. doi:10.1159/000077837

1809. Ramirez CC, Kirsner RS. A refractory case of erythromelalgia involving the ears. *Am J Otolaryngol*. 2004;25(4):251-254. doi:10.1016/j.amjoto.2004.02.008

1810. Rangatchew F, Schoelzer L, Drzewiecki KT, Holmgaard R. EMLA cream in burns: A systematic review of safety, analgesic efficacy, and effects on burn pathophysiology. *J Plast Reconstr Aesthet Surg*. 2024;95:386-401. doi:10.1016/j.bjps.2024.04.001

1811. Rangraz Jeddi F, Nabovati E, Mobayen M, et al. Health care needs, eHealth literacy, use of mobile phone functionalities, and intention to use it for self-management purposes by informal caregivers of children with burns: a survey study. *BMC Med Inform Decis Mak*. 2023;23(1):236. doi:10.1186/s12911-023-02334-w

1812. Ranieri M, Sciuscio M, Cortese AM, et al. The use of alpha-lipoic acid (ALA), gamma linolenic acid (GLA) and rehabilitation in the treatment of back pain: effect on health-related quality of life. *Int J Immunopathol Pharmacol*. 2009;22(3 Suppl CC-Back and Neck CC-Complementary Medicine):45‐50. https://www.cochranelibrary.com/central/doi/10.1002/central/CN-00758433/full

1813. Rao NN, Burns K, Manolikos C, Hodge S. Late-onset multiple acyl-CoA dehydrogenase deficiency: an insidious presentation. *BMJ Case Rep*. 2023;16(5). doi:10.1136/bcr-2022-252668

1814. Rappaport P, Dimnik G, Burns R, Bowie J. Performance indicators for information technology services at four community hospitals. *Healthc Q*. 2006;9(3):80-84. https://search.ebscohost.com/login.aspx?direct=true&AuthType=cookie,ip,shib,uid&db=cul&AN=106243571&site=ehost-live&scope=site

1815. Rasiah J, Prorok JC, Adekpedjou R, et al. Enabling Healthy Aging to AVOID Frailty in Community Dwelling Older Canadians. *Can Geriatr J*. 2022;25(2):202-211. doi:10.5770/cgj.25.536

1816. Rask MR. Medial plantar neurapraxia (jogger’s foot): report of 3 cases. *Clin Orthop Relat Res*. 1978;(134):193-195.

1817. Rasmussen BB, Tipton KD, Miller SL, Wolf SE, Wolfe RR. An oral essential amino acid-carbohydrate supplement enhances muscle protein anabolism after resistance exercise. *J Appl Physiol*. 2000;88(2):386-392. doi:10.1152/jappl.2000.88.2.386

1818. Rasmussen BB, Wolfe RR. Regulation of fatty acid oxidation in skeletal muscle. *Annu Rev Nutr*. 1999;19:463-484. doi:10.1146/annurev.nutr.19.1.463

1819. Ratcliff SL, Brown A, Rosenberg L, et al. The effectiveness of a pain and anxiety protocol to treat the acute pediatric burn patient. *Burns*. 2006;32(5):554-562. doi:10.1016/j.burns.2005.12.006

1820. Ray JJ, Alvarez AD, Ulbrich SL, et al. Shake It Off: a Randomized Pilot Study of the Effect of Whole Body Vibration on Pain in Healing Burn Wounds. *J Burn care Res*. 2017;38 CC-(4):e756‐e764. doi:10.1097/BCR.0000000000000481

1821. Read D, Ashford B. Surgical aspects of Operation Bali Assist: initial wound surgery on the tarmac and in flight. *ANZ J Surg*. 2004;74(11):986-991. doi:10.1111/j.1445-1433.2004.03246.x

1822. Rector JL, Dowd JB, Loerbroks A, et al. Consistent associations between measures of psychological stress and CMV antibody levels in a large occupational sample. *Brain Behav Immun*. 2014;38:133-141. doi:10.1016/j.bbi.2014.01.012
[truncated: 137,603 more chars]
