# Supplementary material for: Effectiveness of physiotherapist-led exercise interventions for burn rehabilitation: A systematic review and meta-analysis
Source: PLoS One. 2024 Dec 31;19(12):e0316658. doi: 10.1371/journal.pone.0316658 (PMC11687864; doi:10.1371/journal.pone.0316658)
Supplement: S3 File — (DOCX) [file pone.0316658.s005.docx]

**Excluded studies (82 studies) with reasons**

**systematic reviews (5 studies)**

1. Disseldorp LM, Nieuwenhuis MK, Baar ME Van, Mouton LJ. Physical Fitness in People After Burn Injury : A Systematic Review. *YAPMR*. 2011;92(9):1501-1510. doi:10.1016/j.apmr.2011.03.025

2. Flores O, Tyack Z, Stockton K, Ware R, Paratz JD. Exercise training for improving outcomes post-burns : a systematic review and meta-analysis. Published online 2018. doi:10.1177/0269215517751586

3. Gittings PM, Grisbrook TL, Edgar DW, Wood FM, Wand BM, Connell NEO. ScienceDirect Resistance training for rehabilitation after burn injury : A systematic literature review &. *Burns*. 2017;44(4):731-751. doi:10.1016/j.burns.2017.08.009

4. Mudawarima T, Chiwaridzo M, Jelsma J, Grimmer K, Muchemwa FC. A systematic review protocol on the effectiveness of therapeutic exercises utilised by physiotherapists to improve function in patients with burns. *Syst Rev*. 2017;6(1):207. doi:10.1186/s13643-017-0592-6

5. Otaghvar HA. A systematic review of the exercise effects on burn wound healing. 2024;(October 2023):1-11. doi:10.1111/iwj.14482

**Led by other health professionals (13 studies)**

1 Abazarnejad E, Froutan R, Ahmadabadi A, Mazlom SR. Improving respiratory muscle strength and health status in burn patients: a randomized controlled trial. *Qual life Res*. 2022;31(3):769‐776. doi:10.1007/s11136-021-02996-x

2. Abdelbasset WK, Abdelhalim NM. Assessing the effects of 6 weeks of intermittent aerobic exercise on aerobic capacity, muscle fatigability, and quality of life in diabetic burned patients: Randomized control study. *Burn*. 2020;46(5):1193-1200. doi:10.1016/j.burns.2019.12.013

3. Ali ZM ibrahiM, El-refay B h., Ali RR. Aerobic exercise training in modulation of aerobic physical fitness and balance of burned patients. *J Phys Ther Sci*. 2015;27:585–589.

4. Al-Mousawi AM, Williams FN, Mlcak RP, Jeschke MG, Herndon DN, Suman OE. Effects of exercise training on resting energy expenditure and lean mass during pediatric burn rehabilitation. *J Burn care Res*. 2010;31(3):400‐408. doi:10.1097/BCR.0b013e3181db5317

5. Celis MM, Suman OE, Huang TT, Yen P, Herndon DN. Effect of a supervised exercise and physiotherapy program on surgical interventions in children with thermal injury. *J Burn Care Rehabil*. 2003;24(1):57‐61; discussion 56. doi:10.1097/00004630-200301000-00014

6. Chao T, Parry I, Palackic A, et al. The effects of short bouts of ergometric exercise for severely burned children in intensive care: A randomized controlled trial. *Clin Rehabil*. 2022;36(8):1052-1061. doi:10.1177/02692155221095643

7. Cucuzzo NA, Ferrando A, Herndon DN. The Effects of Exercise Programming vs Traditional Burned Children. *J Burn Care Rehabil*. 2001;22(3):214-220.

8. Neugebauer CT, Serghiou M, Herndon DN, Suman OE. Effects of a 12-week rehabilitation program with music & exercise groups on range of motion in young children with severe burns. *J Burn Care Res*. 2008;29(6):939-948. doi:10.1097/BCR.0b013e31818b9e0e

9. Omar MT, Hegazy FA, Mokashi SP. Influences of purposeful activity versus rote exercise on improving pain and hand function in pediatric burn. *Burns*. 2012;38(2):261‐268. doi:10.1016/j.burns.2011.08.004

10. Paratz J, Stockton K, Plaza A, Muller M, RJ B. Intensive exercise after thermal injury improves physical, functional, and psychological outcomes. *J Trauma Acute Care Surg*. 2012;73(1):186-194. doi:10.1097/ta.0b013e31824baa52

11. Parry I, Painting L, Bagley A, et al. A Pilot Prospective Randomized Control Trial Comparing Exercises Using Videogame Therapy to Standard Physical Therapy: 6 Months Follow-Up. *J Burn Care Res*. 2015;36(5):534-544. doi:10.1097/BCR.0000000000000165

12. Peña R, Ramirez LL, Crandall CG. Effects of Community-Based Exercise in Children with Severe Burns: A Randomized Trial. *Burns*. 2016;42(1):41-47. doi:10.1016/j.burns.2015.07.007.Effects

13. Przkora R, Herndon DN, Suma OE. The Effects of Oxandrolone and Exercise on Muscle Mass and Function in Children With Severe Burns. *Pediatrics*. 2008;119(1):1-19.

**Duplicates (6 studies)**

1. Flores O, Tyack Z, Stockton K, Ware R, Paratz JD. Exercise training for improving outcomes post-burns: a systematic review and meta-analysis. *Clin Rehabil*. 2018;32(6):734-746. doi:10.1177/0269215517751586

2. Ebid AA, Omar MTA, Abd AM, Baky E. Effect of 12-week isokinetic training on muscle strength in adult with healed thermal burn. *Burns*. 2012;38(1):61-68. doi:10.1016/j.burns.2011.05.007

3. Badawy MM, Allam NM. Impact of Adding Protein Supplementation to Exercise Training on Lean Body Mass and Muscle Strength in Burn Patients. *J Burn care Res*. 2021;42(5):968-974. doi:10.1093/jbcr/irab007

4. Suman OE, Mlcak RP, Herndon DN. Effect of Exercise Training on Pulmonary Function in Children With Thermal Injury. *J ofBurn Care Rehabil*. 2002;(February):288-293. doi:10.1097/01.BCR.0000020443.04389.6B

5. Ebid AA, El-Shamy SM, Draz AH. Effect of isokinetic training on muscle strength , size and gait after healed pediatric burn : A randomized controlled study. *Burns*. 2014;40(1):97-105. doi:10.1016/j.burns.2013.05.022

6. Gittings PM, Wand BM, Hince DA, Grisbrook TL, Wood FM, Edgar DW. The efficacy of resistance training in addition to usual care for adults with acute burn injury: A randomised controlled trial. *BURNS*. 2021;47(1):84-100. doi:10.1016/j.burns.2020.03.015

**Cross sectional and other designs [52 studies]**

1. Al-Ghabeesh SH, Mahmoud MM. Mindfulness and its Positive Effect on Quality of Life among Chronic Burn Survivors: A descriptive Correlational Study. *Burns*. 2022;48(5):1130-1138. doi:10.1016/j.burns.2021.09.022

2. Allahham A, Cooper MN, Fear MW, Martin L, Wood FM. Quality of life in paediatric burn patients with non-severe burns. *Burns*. 2023;49(1):220-232. doi:10.1016/j.burns.2022.03.012

3. Diego AM, Serghiou M, Padmanabha A, Porro LJ, Herndon DN, Suman OE. Exercise training after burn injury: a survey of practice. *J Burn care Res Off Publ Am Burn Assoc*. 2013;34(6):e311-7. doi:10.1097/BCR.0b013e3182839ae9

4. Duke JM, Randall SM, Fear MW, Boyd JH, Rea S, Wood FM. Understanding the long-term impacts of burn on the cardiovascular system. *Burns*. 2016;42(2):366-374. doi:10.1016/j.burns.2015.08.020

5. Elnaggar RK, Osailan AM, Mahmoud WS, Alqahtani BA, Azab AR. Beyond the Acute Phase: Understanding Relationships Among Cardiorespiratory Response to Exercises, Physical Activity Levels, and Quality of Life in Children After Burn Injuries. *J Burn CARE Res*. 2022;43(4):827-833. doi:10.1093/jbcr/irab203

6. Falder S, Browne A, Edgar D, et al. Core outcomes for adult burn survivors: A clinical overview. *Burns*. 2009;35(5):618-641. doi:10.1016/j.burns.2008.09.002

7. Ferguson A, Wright S. Innovative chest physiotherapy techniques (the MetaNeb® System) in the intubated child with extensive burns. *Respir Med Case Reports*. 2017;22:232-234. doi:10.1016/j.rmcr.2017.08.020

8. Flores O, Tyack Z, Stockton K, Paratz JD. The use of exercise in burns rehabilitation: A worldwide survey of practice. *Burns*. 2020;46(2):322-332. doi:10.1016/j.burns.2019.02.016

9. Grisbrook TL, Reid SL, Edgar DW, Wallman KE, Wood FM, Elliott CM. Exercise training to improve health related quality of life in long term survivors of major burn injury: a matched controlled study. *Burns*. 2012;38(8):1165-1173. doi:10.1016/j.burns.2012.03.007

10. Hardwicke J. The influence of outcomes on the provision and practice of burn care. *Burns*. 2016;42(2):307-315. doi:10.1016/j.burns.2015.07.002

11. Heng JS, Clancy O, Atkins J, et al. Revised Baux Score and updated Charlson comorbidity index are independently associated with mortality in burns intensive care patients. *Burns*. 2015;41(7):1420-1427. doi:10.1016/j.burns.2015.06.009

12. Hocking P, Broadhurst M, Nixon RDV, Gannoni A. Validation of the Psychosocial Assessment Tool 2.0 for paediatric burn patients. *Burns*. 2023;49(7):1632-1642. doi:10.1016/j.burns.2023.05.002

13. Angelou IK. Factors that influence the recovery of physical function of adult patients with major burn injuries. *WiredspaceWitsAcZa*. Published online 2020. https://wiredspace.wits.ac.za/server/api/core/bitstreams/3c6c663a-d2dd-48a4-b43d-9052ae7730fd/content

14. Jagnoor J, Lukaszyk C, Fraser S, et al. Rehabilitation practices for burn survivors in low and middle income countries: A literature review. *Burns*. 2018;44(5):1052-1064. doi:10.1016/j.burns.2017.10.007

15. Kondo T, Tsuboi H, Nishiyama K, Takahashi G, Nishimura Y. Effects of rehabilitation treatments jointly considered by physiatrists and rehabilitation therapists in patients with severe burn injury. *Burns*. 2024;50(6):1621-1631. doi:10.1016/j.burns.2024.03.034

16. Leblebici B, Adam M, Baǧiş S, et al. Quality of life after burn injury: The impact of joint contracture. *J Burn Care Res*. 2006;27(6):864-868. doi:10.1097/01.BCR.0000245652.26648.36

17. Martin L, Rea S, Wood F. A quantitative analysis of the relationship between posttraumatic growth, depression and coping styles after burn. *Burns*. 2021;47(8):1748-1755. doi:10.1016/j.burns.2021.05.019

18. Mc Kittrick A, Gustafsson L, Hodson T, Di Tommaso A. Exploration of individuals perspectives of recovery following severe hand burn injuries. *Burns*. 2023;49(2):467-475. doi:10.1016/j.burns.2022.04.026

19. McHargue C, Aden J, Pham TN, Salinas J, Rizzo JA. Precursors to oliguria during major burn resuscitation: An analysis of a prospective observational trial at 5 major US burn centers. *Burns*. 2024;50(6):1513-1518. doi:10.1016/j.burns.2024.03.007

20., et al. Role of exercise in nursing care for burn wound patients: A narrative review from a nursing perspective. *J Nurs Reports Clin Pract*. 2024;0(0):0-0. doi:10.32598/jnrcp.23.101

21. Nitzschke S, Offodile AC, Cauley RP, et al. Long term mortality in critically ill burn survivors. *Burns*. 2017;43(6):1155-1162. doi:10.1016/j.burns.2017.05.010

22. Omar MT, Ibrahim ZM, Salama AB. Patterns and predictors of hand functional recovery following pediatric burn injuries: Prospective cohort study. *Burns*. 2022;48(8):1863-1873. doi:10.1016/j.burns.2021.11.021

23. Öner M, Kalanlar B, Demir S, Özyurt N, Erul A, Şenel E. Challenges, expectations, and cultural care experiences of nurses regarding migrant children receiving burn treatment and their caregivers: A qualitative study. *Burns*. 2023;49(7):1706-1713. doi:10.1016/j.burns.2023.02.004

24. Benjamin NC, Andersen CR, Herndon DN, Suman OE. The effect of lower body burns on physical function. *Burns*. 2015;41(8):1653-1659. doi:10.1016/j.burns.2015.05.020

25. Özkal Ö, Seyyah M, Topuz S, Konan A. Lower limb functional status and its determinants in moderate/major burns 3–6 months following injury: A two-center observational study. *Burns*. 2021;47(3):676-683. doi:10.1016/j.burns.2020.07.025

26. Palackic A, Suman OE, Porter C, Murton AJ, Crandall CG, Rivas E. Rehabilitative Exercise Training for Burn Injury. *Sports Med*. 2021;51(12):2469-2482. doi:10.1007/s40279-021-01528-4

27. Pereira C, Murphy K, Herndon D. Outcome measures in burn care is mortality dead? *Burn*. 2004;30(8):761-771. doi:10.1016/j.burns.2004.05.012

28. Ren Z, Chang WC, Zhou Q, Wang Y, Wang H, Hu D. Recovery of lost face of burn patients, perceived changes, and coping strategies in the rehabilitation stage. *Burns*. 2015;41(8):1855-1861. doi:10.1016/j.burns.2015.08.033

29. Shields BA, Carpenter JN, Bustillos BD, et al. The Interplay of Nutrition, Physical Activity, Severity of Illness, and Mortality in Critically Ill Burn Patients: Is There a Connection? *J Burn Care Res*. 2019;40(6):936-942. doi:10.1093/jbcr/irz126

30. Simon MH, Ujjal MUR, Botman M, van Hövell tot Westerflier C, Ahmed MS, Vries AM de. Burn injuries and acute burn management in the rural areas in northern Bangladesh – A household survey. *Burns*. 2024;50(6):1480-1486. doi:10.1016/j.burns.2024.03.030

31. Sjoberg F, Elmasry M, Abdelrahman I, et al. The impact and validity of the Berlin criteria on burn-induced ARDS: Examining mortality rates, and inhalation injury influences. A single center observational cohort study. *Burns*. 2024;50(6):1528-1535. doi:10.1016/j.burns.2024.05.005

32. Sliwa JA, Heinemann A, Semik P. Inpatient rehabilitation following burn injury: Patient demographics and functional outcomes. *Arch Phys Med Rehabil*. 2005;86(10):1920-1923. doi:10.1016/j.apmr.2005.04.024

33. Smailes S, Spoors C, da Costa FM, Martin N, Barnes D. Early tracheostomy and active exercise programmes in adult intensive care patients with severe burns. *BURNS*. 2022;48(7):1599-1605. doi:10.1016/j.burns.2021.10.005

34. Smailes ST, Eagan JH, Matanle M, Barnes D. The predictive validity of the Functional Assessment for Burns — Critical Care (FAB-CC) score for discharge outcomes in major burns. *Burns*. 2021;47(7):1639-1646. doi:10.1016/j.burns.2021.02.011

35. Chen W hao, Ye H feng, Wu Y xuan, et al. Association of creatinine-albumin ratio with 28-day mortality in major burned patients: A retrospective cohort study. *Burns*. 2023;49(7):1614-1620. doi:10.1016/j.burns.2023.04.002

36. Smailes ST, Engelsman K, Dziewulski P. Physical functional outcome assessment of patients with major burns admitted to a UK Burn Intensive Care Unit. *Burns*. 2013;39(1):37-43. doi:10.1016/j.burns.2012.05.007

37. Tang D, Li-Tsang CWP, Au RKC, et al. Predictors of functional independence, quality of life, and return to work in patients with burn injuries in mainland China. *Burn Trauma*. 2016;4(1):1-11. doi:10.1186/s41038-016-0058-4

38. Wearn C, Hardwicke J, Kitsios A, Siddons V, Nightingale P, Moiemen N. Outcomes of burns in the elderly: Revised estimates from the Birmingham Burn Centre. *Burns*. 2015;41(6):1161-1168. doi:10.1016/j.burns.2015.04.008

39. Wendler CB, Irakoze V. Utility of palm and hand surface area in approximating burn extent in Burundian adults and children. *Burns*. 2022;48(2):456-458. doi:10.1016/j.burns.2021.02.003

40. Willis CE, Grisbrook TL, Elliott CM, Wood FM, Wallman KE, Reid SL. Pulmonary function, exercise capacity and physical activity participation in adults following burn. *Burns*. 2011;37(8):1326-1333. doi:10.1016/j.burns.2011.03.016

41. Zhang A, Vazquez S, Das A, et al. High area deprivation index is associated with increased injury severity in pediatric burn patients. *Burns*. 2023;49(7):1670-1675. doi:10.1016/j.burns.2023.05.018

42. Zhu Z, Kong W, Lu Y, et al. Epidemiological and clinical features of paediatric inpatients for scars: A retrospective study. *Burns*. 2023;49(7):1719-1728. doi:10.1016/j.burns.2023.02.008

43. Chouinard NH, Beaudoin Cloutier C, Chang SL, et al. The economic burden of burned patients for hospitalization in Canada. *Burns*. 2024;50(6):1494-1503. doi:10.1016/j.burns.2024.03.036

44. Cornet PA, Niemeijer AS, Figaroa GD, et al. Clinical outcome of patients with self-inflicted burns. *Burns*. 2017;43(4):789-795. doi:10.1016/j.burns.2016.11.005

45. da Silva MMM, Travensolo C de F, Probst VS, Felcar JM. Quantification of changes in functional capacity and muscle strength in patients: a burn intensive care unit cohort study. *Burns*. 2022;48(4):833-840. doi:10.1016/j.burns.2022.01.010

46. de Figueiredo TB, Utsunomiya KF, de Oliveira AMRR, Pires-Neto RC, Tanaka C. Mobilization practices for patients with burn injury in critical care. *Burns*. 2020;46(2):314-321. doi:10.1016/j.burns.2019.07.037

47. Deng H, Chen J, Li F, et al. Effects of mobility training on severe burn patients in the BICU: A retrospective cohort study. *Burns*. 2016;42(7):1404-1412. doi:10.1016/j.burns.2016.07.029

48. Baldwin J, Li F. Exercise behaviors and barriers to exercise in adult burn survivors: A questionnaire survey. *Burn trauma*. 2013;1(3):134-139. doi:10.4103/2321-3868.123075

49. Atkins WC, Romero SA, Moralez G, et al. Attrition of Well-Healed Burn Survivors to a 6-Month Community-Based Exercise Program: A Retrospective Evaluation. *J Burn CARE Res*. 2023;44(6):1478-1484. doi:10.1093/jbcr/irad063

50. Disseldorp LM, Mouton LJ, Takken T, et al. Design of a cross-sectional study on physical fitness and physical activity in children and adolescents after burn injury. *BMC Pediatr*. 2012;12(1):195. doi:10.1186/1471-2431-12-195

51. Foncerrada G, Capek KD, Wurzer P, et al. Functional Exercise Capacity in Children With Electrical Burns. *J Burn Care Res*. 2017;38(3):e647-e652. doi:10.1097/BCR.0000000000000443

52. Rivas E, Herndon DN, Beck KC, Suman OE. Children with burn injury have impaired cardiac output during submaximal exercise. *Med Sci Sports Exerc*. 2017;49(10):1993-2000. doi:10.1249/MSS.0000000000001329

**Studies with no control (6 studies)**

1. Akkerman M, Mouton LJ, de Groot S, et al. Predictability of exercise capacity following pediatric burns: a preliminary investigation. *Disabil Rehabil*. 2021;43(5):703-712. doi:10.1080/09638288.2019.1641846

2. Zhang Y, Ma X, Pang J. Identification and quantification of physical activity in critically ill burn patients: a feasibility study. *Res Int Bus Financ*. Published online 2023:101890. doi:10.1016/j.vetpar.2024.110145

3. Morris LD, Louw QA, Crous LC. Feasibility and potential effect of a low-cost virtual reality system on reducing pain and anxiety in adult burn injury patients during physiotherapy in a developing country. *Burns*. 2010;36(5):659-664. doi:10.1016/j.burns.2009.09.005

4. Pham TN, Wong JN, Terken T, Gibran NS, Carrougher GJ, Bunnell A. Feasibility of a Kinect ® -based rehabilitation strategy after burn injury. *Burns*. 2018;44(8):2080-2086. doi:10.1016/j.burns.2018.08.032

5. Sizoo SJM, Akkerman M, Trommel N, et al. Feasibility and acceptability of aquatic exercise therapy in burn patients – A pilot study. *Burn Open*. 2021;5(1):10-20. doi:10.1016/j.burnso.2020.10.001

6. Tapking C, Armenta AM, Popp D, et al. Relationship between lean body mass and isokinetic peak torque of knee extensors and flexors in severely burned children. *Burns*. 2019;45(1):114-119. doi:10.1016/j.burns.2018.09.007
