# Supplementary material for: Effectiveness of physiotherapist-led exercise interventions for burn rehabilitation: A systematic review and meta-analysis
Source: PLoS One. 2024 Dec 31;19(12):e0316658. doi: 10.1371/journal.pone.0316658 (PMC11687864; doi:10.1371/journal.pone.0316658)
Supplement: S1 Table — (DOCX) [file pone.0316658.s006.docx]

**S1 Table.** Quality of evidence using the GRADE approach

| **Quality assessment** | | | | | | **Number of patients** | | **Effects size** | **Quality** | **Importance** |
| --- | --- | --- | --- | --- | --- | --- | --- | --- | --- | --- |
| Number of studies (sample) | Risk of bias | Inconsistency | Indirectness | Imprecision | Publication bias | Intervention | Control | Hedge’s (95% CI) |  |  |
| **Lean body mass (kg) (follow up: mean 12weeks; assessed with Dual energy X-ray absorptiometry)** | | | | | | | | | | |
| 1 RCT (adults) | Serious^a^ | Serious^b^ | Not serious | Serious^d^ | - | 15 | 15 | - | ⨁◯◯◯  Low | Critical |
| 1 RCT (children) | Serious^a^ | Serious^b^ | Not serious | Serious^d^ | - | 24 | 23 | - | ⨁◯◯◯  Low | Critical |
| **Quality of life (BSHS-B) (follow up: range 6 to 12 weeks; assessed with: paediatric quality of life inventory and Burns-Specific Health Scale-Brief (BSHS-B)** | | | | | | | | | | |
| 2 RCTs (adults) | Serious^a^ | Serious^a^ | Not serious | Serious^d^ | - | 51 | 48 | - | ⨁◯◯◯  Low | Critical |
| 1 RCT (Children) | Serious^a^ | Serious^a^ | Not serious | Serious^d^ | - | 18 | 18 | - | ⨁◯◯◯  Low | Critical |
|  |  |  |  |  |  |  |  |  |  |  |
| **Pulmonary function (L/min) (follow up: mean 12 weeks; assessed with: spirometry)** | | | | | | | | | | |
| 1 RCT (adults) | Serious^a^ | Serious^b^ | Not serious | Serious^d^ | - | 54 | 56 | - | ⨁◯◯◯  Low | Critical |
| **Muscle strength (N/m) (follow up: range 6 to 12 weeks; assessed with handheld dynamometer, muscular ultrasound, MRC scale, Biodex system dynamometer)** | | | | | | | | | | |
| 4 RCTs (adults) | Serious^a^ | Not Serious | Not serious | Serious^d^ | - | 86 | 89 | 2.27 [0.42; 4.13] | ⨁⨁◯◯  Moderate | Critical |
| **Aerobic capacity (ml/kg/min) (follow up: range 6 to 12 weeks; assessed with: 6-minute walk test, McMaster cycling protocol and a modified Bruce treadmill protocol)** | | | | | | | | | | |
| 2 RCTs (Children) | Serious^a^ | Not serious | Not serious | Serious | - | 118 | 100 | 1.13 [0.44; 1.83] | ⨁⨁◯◯  Moderate | Critical |

a=some included studies have a high risk of bias, b=there appears to be some inconsistency reported by the included studies, d= small number of included studies (<5) and sample size (<100) BSHS-B=Burns-Specific Health Scale Brief, CI=Confidence interval, L/min=litre per minute, kg=kilograms, ml/kg/min=millilitres per kilogram per minute, MRC=Medical Research Council, N/m=Newton metre, RCTs=Randomised Controlled Trial, VO_2peak_= maximal oxygen uptake
